# The complete genome of *Blastobotrys (Arxula) adeninivorans* LS3 - a yeast of biotechnological interest

**DOI:** 10.1186/1754-6834-7-66

**Published:** 2014-04-24

**Authors:** Gotthard Kunze, Claude Gaillardin, Małgorzata Czernicka, Pascal Durrens, Tiphaine Martin, Erik Böer, Toni Gabaldón, Jose A Cruz, Emmanuel Talla, Christian Marck, André Goffeau, Valérie Barbe, Philippe Baret, Keith Baronian, Sebastian Beier, Claudine Bleykasten, Rüdiger Bode, Serge Casaregola, Laurence Despons, Cécile Fairhead, Martin Giersberg, Przemysław Piotr Gierski, Urs Hähnel, Anja Hartmann, Dagmara Jankowska, Claire Jubin, Paul Jung, Ingrid Lafontaine, Véronique Leh-Louis, Marc Lemaire, Marina Marcet-Houben, Martin Mascher, Guillaume Morel, Guy-Franck Richard, Jan Riechen, Christine Sacerdot, Anasua Sarkar, Guilhem Savel, Joseph Schacherer, David J Sherman, Nils Stein, Marie-Laure Straub, Agnès Thierry, Anke Trautwein-Schult, Benoit Vacherie, Eric Westhof, Sebastian Worch, Bernard Dujon, Jean-Luc Souciet, Patrick Wincker, Uwe Scholz, Cécile Neuvéglise

**Affiliations:** 1 Leibniz Institute of Plant Genetics and Crop Plant Research (IPK), Corrensstr. 3, Gatersleben D-06466, Germany; 2 AgroParisTech, Micalis UMR 1319, CBAI, Thiverval-Grignon, F-78850, France; 3 INRA French National Institute for Agricultural Research, Micalis UMR 1319, CBAI, Thiverval-Grignon F-78850, France; 4 Institute of Plant Biology and Biotechnology, University of Agriculture in Krakow, Al. 29 Listopada 54, Krakow 31-425, Poland; 5 LaBRI (UMR 5800 CNRS) and project-team Magnome INRIA Bordeaux Sud-Ouest, Talence F-33405, France; 6 Bioinformatics and Genomics Programme, Centre for Genomic Regulation (CRG), Dr. Aiguader 88, Barcelona 08003, Spain; 7 Universitat Pompeu Fabra (UPF), Barcelona 08003, Spain; 8 Université de Strasbourg, Architecture et Réactivité de l’ARN, Institut de Biologie Moléculaire et Cellulaire du CNRS, F-67084 Strasbourg, France; 9 Aix-Marseille Université, CNRS UMR 7283, Laboratoire de Chimie Bactérienne, F-13402 Marseille, Cedex 20, France; 10 CEA, Saclay Biology and Technologies Institute (iBiTec-S), Gif-sur-Yvette F-91191, France; 11 Université catholique de Louvain, Institut des Sciences de la Vie, Croix du Sud 5/15, Louvain-la-Neuve 1349, Belgium; 12 CEA, Institut de Génomique, Genoscope, 2 Rue Gaston Crémieux, Évry F-91000, France; 13 Université Catholique de Louvain, Earth and Life Institute (ELI), Louvain-la-Neuve 1348, Belgium; 14 School of Biological Sciences, University of Canterbury, Private Bag 4800, Christchurch, New Zealand; 15 Université de Strasbourg, CNRS UMR7156, Strasbourg F-67000, France; 16 Institute of Biochemistry, University of Greifswald, Felix-Hausdorffstraße 4, Greifswald D-17487, Germany; 17 Institut de Génétique et Microbiologie, Université Paris-Sud, UMR CNRS 8621, F- Orsay CEDEX 91405, France; 18 Laboratory of Bioinformatics and Protein Engineering, International Institute of Molecular and Cell Biology in Warsaw, ul. Ks. Trojdena 4, Warsaw 02-109, Poland; 19 CNRS UMR 8030, 2 Rue Gaston Crémieux, Évry F-91000, France; 20 Université d’Evry, Bd François Mitterand, Evry F-91025, France; 21 Institut Pasteur, Université Pierre et Marie Curie UFR927, CNRS UMR 3525, F-75724 Paris-CEDEX 15, France; 22 Université Lyon 1, CNRS UMR 5240, Villeurbanne F-69621, France; 23 Present address: École Normale Supérieure, Institut de Biologie de l’ENS (IBENS), 46 rue d’Ulm, Paris F-75005, France; 24 INRA Institut Micalis UMR 1319, AgroParisTech, BIMLip, Avenue de Bretignières, Bât. CBAI, Thiverval-Grignon 78850, France; 25 Yeast Genetics, Leibniz Institute of Plant Research (IPK), Corrensstrasse 3, Gatersleben 06466, Germany

**Keywords:** Yeast, Genome, Biotechnology, Tannic acid, n-butanol, Metabolism

## Abstract

**Background:**

The industrially important yeast *Blastobotrys (Arxula*) *adeninivorans* is an asexual hemiascomycete phylogenetically very distant from *Saccharomyces cerevisiae*. Its unusual metabolic flexibility allows it to use a wide range of carbon and nitrogen sources, while being thermotolerant, xerotolerant and osmotolerant.

**Results:**

The sequencing of strain LS3 revealed that the nuclear genome of *A. adeninivorans* is 11.8 Mb long and consists of four chromosomes with regional centromeres. Its closest sequenced relative is *Yarrowia lipolytica*, although mean conservation of orthologs is low. With 914 introns within 6116 genes, *A. adeninivorans* is one of the most intron-rich hemiascomycetes sequenced to date. Several large species-specific families appear to result from multiple rounds of segmental duplications of tandem gene arrays, a novel mechanism not yet described in yeasts. An analysis of the genome and its transcriptome revealed enzymes with biotechnological potential, such as two extracellular tannases (Atan1p and Atan2p) of the tannic-acid catabolic route, and a new pathway for the assimilation of n-butanol via butyric aldehyde and butyric acid.

**Conclusions:**

The high-quality genome of this species that diverged early in *Saccharomycotina* will allow further fundamental studies on comparative genomics, evolution and phylogenetics. Protein components of different pathways for carbon and nitrogen source utilization were identified, which so far has remained unexplored in yeast, offering clues for further biotechnological developments. In the course of identifying alternative microorganisms for biotechnological interest, *A. adeninivorans* has already proved its strengthened competitiveness as a promising cell factory for many more applications.

## Background

This paper discusses the sequencing of the genome of *Arxula adeninivorans,* a yeast of biotechnological interest. This species is currently exploited as biocatalyst for the synthesis of various biotechnological products such as tannases [1], 1-(S)-phenylethanol [2] or β-D-galactopyranoside [3], for the production of food with low purine content [4], and for the detection of estrogenic activity in various aqueous media [5,6]. It is also used as a host for the production of recombinant proteins, and as a donor for genes encoding valuable products [7,8]. Also developed as a microbial fuel cell, *A. adeninivorans* is shown to have a higher power output than *Saccharomyces cerevisiae* due to the production of an extracellular redox molecule [9].

This species was first described by Middelhoven *et al*. [10] who isolated a yeast strain from soil and designated it as *Trichosporon adeninovorans* CBS 8244^T^. This strain was found to exhibit unusual biochemical activities, including the ability to assimilate a wide range of amines, adenine and several other purine compounds as a sole energy and carbon source. A second wild-type isolate (strain LS3 (PAR-4)) with characteristics similar to CBS 8244^T^ was selected from wood hydrolysates in Siberia, and additional strains were later isolated from chopped maize silage or humus-rich soil. A new genus name *Arxula* Van der Walt, Smith & Yamada (c*andidaceae*) was proposed for all of these strains [11,12]. No sexual reproduction has been observed in any of these strains, showing that they are all anamorphic ascomycetes. They also share common properties, such as nitrate assimilation and xerotolerance [13].

Kurtzmann and Robnett [14] revisited the phylogeny of yeasts and deduced that *Arxula* is a member of the *Blastobotrys* genus that contains both anamorphic and ascosporic species. Recent classifications consider this taxon as basal to the hemiascomycete tree in a region where genomic data are available for few other species [15]. This sequencing bias remains despite the number of recent publications of yeast genome sequences. For instance, *Ogataea angusta* (*Hansenula polymorpha*), *Komagataella (Pichia) pastoris, Dekkera bruxellensis* or more recently *Kuraichia capsulata*, use the basal yeast species *Yarrowia lipolytica,* which is the closest one of *A. adeninivorans*, as a single outgroup [16-19].

Thus, sequencing of the *Blastobotrys (Arxula) adeninivorans* genome was of interest in order to generate an additional landmark in the basal portion of the hemiascomycete tree and possibly resolve phylogenetic relationships among basal species. In addition, the sequence provides biotechnologists with complete information on the gene content of this species for which only 40 different protein entries are currently recorded in databases.

## Results

### Genome architecture and main non-coding genetic elements

The *A. adeninivorans* strain LS3 was selected because of its established biotechnological use [20]. Both mitochondrial and nuclear genomes were sequenced using the Sanger and 454 pyrosequencing approaches with different shotgun, plasmid and BAC libraries (Additional file 1). The circular mapping mitochondrial genome has a final size of 31,662 bp. It encodes 24 tRNA genes, 15 protein-coding genes including the seven NADH: ubiquinone dehydrogenase subunits of complex I, the genes encoding the RNA component of RNase P and the two subunits of the mitochondrial ribosomal RNA, as expected from the phylogenetic position of this species. All of these genes are transcribed from the same DNA strand except for the tRNA-Cys gene (Additional file 2).

**Figure 1:**
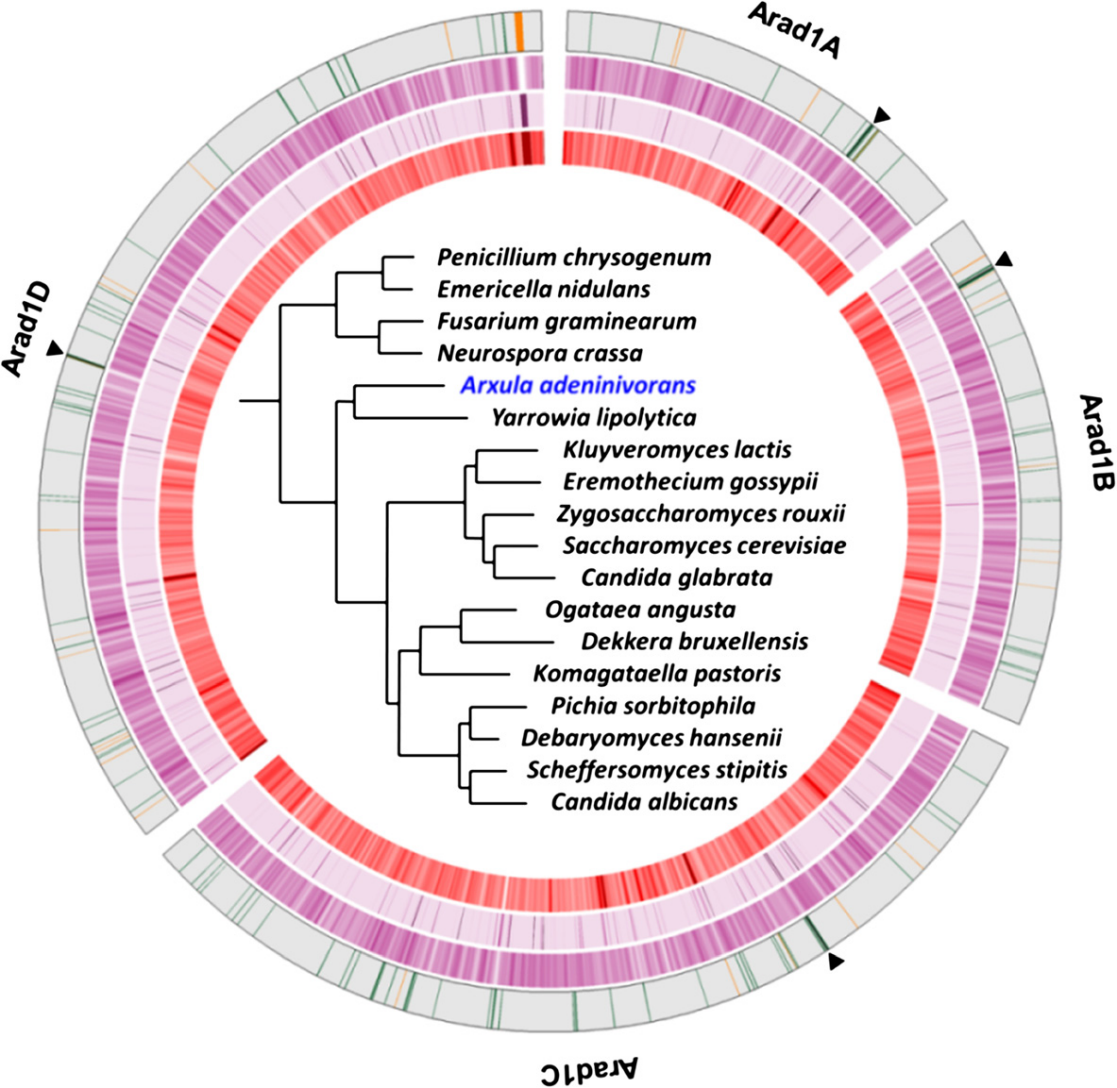
**Circos map of the complete nuclear genome of *A. adeninivorans *LS3.** Chromosome structure (the outermost circle - circle 1): presumed centromeric positions are indicated by black bands and black triangles outside the circle, and tRNA and rRNA genes by green and orange bands, respectively. Genes (circle 2): density of genes in the filtered gene set across the genome, from a gene count per 15 kb sliding window at 5 kb intervals. Repeat content (circle 3): for creating k-mer density ring, k-mers with length = 20 in whole genome using jellyfish program v. 1.1.1 (http://www.cbcb.umd.edu) were counted, a position map of k-mer count was created, k-mers counted in blocks of 3 kb were divided by 3,000 and the data was plotted using Circos’s heatmap. 454 reads mapped to chromosomes (circle 4): density of 454 reads mapped to chromosomes, from a 454 read count per 9 kb sliding window at 3 kb intervals. Underlined blocks indicate alignment in the reverse strand. In the centre of the Circos map the phylogenetic relationship of *A. adeninivorans* is presented as inferred by gene tree parsimony analysis of the complete *A. adeninivorans* phylome. k-mer, tuple of length k.

After directed finishing phases, the 11.8 Mb final assembly of the nuclear genome resulted in four contigs corresponding to the four chromosomes *Arad1A*, *Arad1B*, *Arad1C* and *Arad1D*, of 1,659,397, 2,016,785, 3,827,910, and 4,300,524 nt, respectively, as predicted from previous pulsed-field gel electrophoresis analyses [21] (Figure 1). *K. pastoris* and *Y. lipolytica* have four and six chromosomes respectively, while an average of eight and sixteen chromosomes is observed in protoploid and post-whole genome duplication species [22]. This may suggest a whole genome duplication event during early hemiascomycete evolution although there is presently no other evidence to support this hypothesis [23]. The four contigs contain no internal gaps and lack only terminal repeats at the telomeres. There is a single rDNA cluster, located approximately 75 kb upstream of the chromosome D right subtelomere. Based on 454 read counts, there are about 35 to 40 tandem repeats at this locus of the 18S, 5.8S and 26S rRNA genes, the latter housing a 411-bp group-IC, self-splicing intron [24]. We have left two copies of the rDNA units in *Arad1D* flanked by two artificial gaps of 874 “N”. As in *Y. lipolytica*, the 5S rRNA genes are not included in the rDNA repeat, but occur as 30 copies dispersed throughout the genome (Table 1). A set of 46 tDNAs encoded by 147 tRNA genes was identified and confirmed that *A. adeninivorans* follows the regular eukaryotic-type sparing rules to read CTY Leu and CGA Arg codons [25]. Forty seven genes encoding snRNAs or snoRNAs were identified: the small nuclear RNAs (U1, U2, U4, U5 and U6), the RNA components of the RNase P, and of the signal recognition particle, as well as 14 H/ACA and 33 C/D snoRNAs (Additional file 3)*.* Additionally, three thiamine pyrophosphate (TPP) riboswitch sequence candidates were found in the 5′ region from homologs of *S. cerevisiae THI4* (YGR144W)*, UGA4* (YDL210W) and *DUR3* (YHL016C), namely ARAD1R43560g, ARAD1D08074g and ARAD1B12386g; they show a remarkable conservation of known structural domains and sequence motifs [26]. A single transposable element was identified on chromosome B (Taa3, ARAD1B13860t) that belongs to the *Gypsy* superfamily of Long Terminal Repeat (LTR) retrotransposons with the two gag and pol open reading frames separated by a minus 1 frameshift as seen in the homologous element Tyl6 of *Y. lipolytica*[27]. The single copy of Taa3 was found 13 bp upstream of a tRNA gene, suggesting a possible specificity of insertion [28]. Only three relics of solo LTRs were identified in the genome, which implies that Taa3 has low activity.

**Table 1:** **General features of *A. adeninivorans *** **LS3 nuclear genome**

	**Chromosome**			**CDS**			**Pseudo-genes**	**i-genes**	**Introns**	**tRNA**	**5S rRNA**	**ncRNA**
	**Size**	**G + C %**	**Coding %**	**#**	**G + C %**	**Mean Size (nt)**						
*Arad1A*	1659397	48.2	73.2	871	49.6	1395	3	84	106	13	4	9
*Arad1B*	2016785	48.4	72.6	1051	49.8	1394	5	109	135	31	8	5
*Arad1C*	3827910	48.0	75.8	1991	49.2	1457	11	260	343	54	6	16
*Arad1D*	4300524	48.1	73.6	2203	49.3	1437	14	250	330	49	12	15
**Total**	**11804616**	**48.1**	**74.1**	**6116**	**49.4**	**1430**	**33**	**703**	**914**	**147**	**30**	**45**

CDS, coding DNA sequence; G, guanine, C, cytosine; ncRNA, non coding RNA.

Putative centromeres were identified within one region per chromosome with a conspicuous G + C (Guanine + cytosine) bias defining approximately 6 kb G + C troughs, with a G + C content of 31 to 33% as compared to 48% for the whole genome. Like those of *Y. lipolytica*, they share features of both regional centromeres found in yeasts of the CTG group, and of point centromeres characteristic of *Saccharomycetaceae*[29] (Additional file 4).

### Protein-coding genes, pseudogenes, introns

A total of 6,116 protein-coding genes and 33 pseudogenes were identified. This is slightly less than reported for *Y. lipolytica* or *Debaryomyces hansenii*, but significantly more than for the *Saccharomycetaceae* species (Table 2). The frequency of pseudogenes is one of the lowest reported in hemiascomycetes, while gene density is one of the highest.

**Table 2:** **Annotated features of *A. adeninivorans *** **when compared to other representative Hemiascomycetes**

**Species**	***S. cerevisiae***	***L. thermotolerans***	***D. hansenii***	***Y. lipolytica***	***A. adeninivorans***
**Strain**	**S288c**	**CBS 6340**	**CBS 767**	**E150**	**LS3**
Chromosome number	16	8	7	6	4
Genome					
Ploidy	n	2n	n	n	n
Size	12.1	10.4	12.2	20.5	11.8
Average G + C content (%)	38.3	47.3	36.3	49.0	48.1
Genome coding coverage (%)	70.0	72.3	74.2	46	74.1
CDS					
Total CDS (pseudo)	5769	5094 (46)	6272 (129)	6449 (137)	6116 (33)
Average G + C (%)	39.6	49.2	38.0	52.9	49.4
Average size (aa)	485	492	479	476	477
i-genes	287	278	420	984	703
Introns	296	285	467	1119	914
Total tRNA genes	274	229	205	510	147
Total snRNA	6	5	5	6	5
Total snoRNA	77	43	ND	ND	37
rDNA clusters	1 (internal)	1 (internal)	3 (internal)	6 (subtelomeric)	1 (internal)
Total dispersed 5S rRNA genes	0	0	0	116	30

snRNA, small nuclear ribonucleic acid; snoRNA, small nucleolar ribonucleic acid; CDS, coding DNA sequence; G + C, guanine and cytosine; aa, amino acids; i-genes, intron-containing genes; ND, not-determined. Data from *S. cerevisiae, Lachancea thermotolerans, D. hansenii* and *Y. lipolytica* were taken from [30]; data on intron-containing genes from [31].

A total of 4,815 (78.7%) genes were assigned to gene ontology (GO) terms: 3,853 genes to molecular functions, 2,626 to cellular components and 3,308 to biological processes. In the biological processes, the largest fraction of genes, 1,351 (22.1%), was assigned to metabolism, while in the molecular functions the largest category was represented by genes encoding catalytic activities. The GO slim categories of *A. adeninivorans* are presented in Additional file 5. InterPro domains were detected in 5,147 (84.2%) proteins corresponding to 459 distinct Pfam domains. A secretion signal peptide of type I or type II was predicted in 957 (15.6%) gene products, including *Atan1p* and *Alip1p* that were previously characterized experimentally by N-terminal sequencing and mass spectrometry (MS) analysis [32,33]. Transmembrane helices were found in 1,271 (20.8%) proteins. An Enzyme Commission (EC) number was assigned to 676 (11.1%) genes. We assigned 884 (14.4%) genes to 98 metabolic pathways present at the Kyoto Encyclopedia of Genes and Genomes (KEGG) with the highest number of genes related to purine metabolism. Blast2GO BLASTx alignments using the NCBI NRPEP database confirmed that the closest matches to *A. adeninivorans* genes were very often found in *Y. lipolytica* (Additional file 5).

Spliceosomal introns are more frequent than in *Saccharomycetaceae* or in *Debaryomycetaceae*, but in the same range as reported for *Y. lipolytica* (914 versus 1119, Table 2). In this latter species, introns are characterized by a very short distance between the 3′ splicing site and the branch point, but have in contrast retained the ancestral consensus hemiascomycete 5′ splicing site (GTAAGT). Finally, multi-intronic genes tend to be more frequent in *A. adeninivorans* than in *Y. lipolytica* (21.5% *vs*. 11.5%). For additional information, see Additional file 6 and Genosplicing [31].

### Phylogeny and synteny conservation

A phylogenetic tree was reconstructed for each *A. adeninivorans* protein-coding gene, the so-called phylome, and used to identify orthology and paralogy relationships among related species [34]. This comprehensive collection of evolutionary histories is publicly available at PhylomeDB [35]. Species phylogenies were computed on a set of concatenated orthologs and using a super tree approach combining all individual gene phylogenies. The two methods gave the same topology, in which *A. adeninivorans* groups with *Y. lipolytica* (Additional file 7), although the two species have greatly diverged. For instance, our analyses identified 2,520 *A. adeninivorans* proteins that lack an ortholog in *Y. lipolytica,* 591 of which do not even have a homolog in that species. For 121 proteins we could only detect homologs in *Pezizomycotina* genomes (Additional file 8). Horizontal gene transfer between prokaryotes and fungi was detected using a published pipeline [36], which pinpointed six candidates with putative enzymatic function that are likely to have been transferred from prokaryotes to *Arxula* (Additional file 8). Few genes of bacterial origin have been reported in *Saccharomycotina* so far, but most of them encode metabolic enzymes with important physiological roles that may facilitate host adaptation to biotope variations (see [36,37] for large-scale trans-kingdom transfer in fungi).

The number of conserved gene blocks between *A. adeninivorans* and other genomes ranged from 300 with *S. cerevisiae* to >800 with *Y. lipolytica*, and was roughly proportional to the mean percentage of protein similarity, as is expected when species have greatly diverged. Indeed, in the comparison between *Y. lipolytica* and *A. adeninivorans*, 92% of the blocks contained less than four genes, showing that there is no large-scale conservation of synteny (Additional file 7).

### Gene families: expansion and contraction

**Figure 2:**
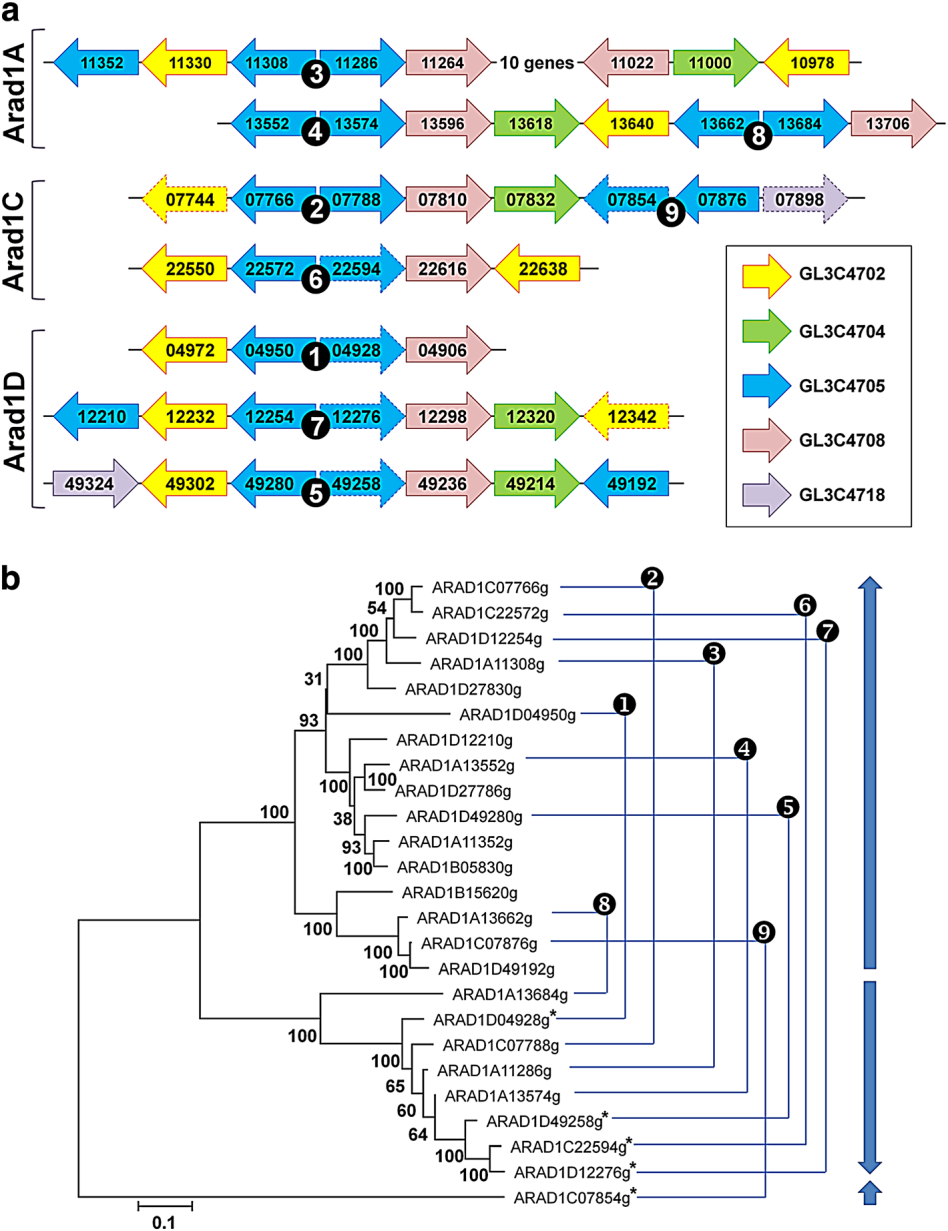
**Tandem gene arrays in *A. adeninivorans. *(a)** Intermingled families. *A. adeninivorans* chromosomes are indicated on the left. Gene members of TGAs are depicted by boxed arrows colored according to their family. Family numbers refer to the Génolevures classification as shown in the legend in the box on the right. Pseudogenes are indicated by dotted lines. The *GL3C4705* family is the largest one. Most of its members are tail-to-tail inverted tandem repeats, numbered from one to nine in black disks. **(b)** Neighbor-joining tree based on the muscle [39] alignment of positions one and two of the codons. Robustness of the tree is indicated by 100 bootstrap values calculated with a maximum composite likelihood model with uniform rates. Thin blue lines indicate pairs in inverted repeats of *GL3C4705* family; heavy blue lines indicate relative orientation of genes in inverted repeats (see Additional file 11 for additional information).

The gene trees in the phylome were scanned to detect and date duplication events [38]. With an average of 0.253 duplications per gene in the specific lineage leading to *Arxula,* this genome does not seem to contain a large amount of duplications. This is nevertheless greater than the 0.015 value found in the common ancestor of *Y. lipolytica* and *A. adeninivorans* (Additional file 9). Most *Arxula*-specific expansions are not very large (between three and nine sequences) and correspond to peptidases, transporters, dehydrogenases and some proteins related to nitrogen metabolism (Additional file 9). One expansion, however, contains over 100 members of unknown function and no homologs in any database, which is to our knowledge the largest gene family described in yeast (Figure 2 and Additional file 9).

There are fewer transporters in *A. adeninivorans* than in for example* D. hansenii* or *Y. lipolytica*, but some have undergone strong amplification. Remarkably, sugar transporters appear overrepresented in this species: there are 60 members of the Sugar Porter family, which is three times as many as in *Kluyveromyces lactis* or *K. pastoris*, and 1.8 times more than in *S. cerevisiae* (Additional file 10). These include 15 glycerol: H^+^ symporters, paralogs of the *S. cerevisiae* singleton *STL1*, compared to eight in the osmotolerant yeast *D. hansenii*, which may reflect the salt tolerance of *A. adeninivorans*. The ability to use various carbon sources is highlighted by the abundance of high affinity glucose: H^+^ symporters (10 members), maltose: H^+^ symporters (10 members), lactose permeases (four members versus one in *K. lactis* and *D. hansenii*), allantoate permeases (six members), and of facilitators for the uptake of xylose (six members), quinate (four members), fructose (four members) and myo-inositol (three members). Surprisingly, there are few glucose uniporters (two members, versus eighteen and four in *S. cerevisiae* and *D. hansenii*, respectively) and few sugar sensors. High-affinity nicotinic acid transporters (six members), polyamine transporters (15 members) and nitrate/nitrite permeases (three members) are also amplified (Additional file 10).

About 10% of the duplicated genes (213/2285) are organized in tandem gene arrays (TGAs), mostly as arrays of two genes. These arrays are sometimes entirely duplicated on the same or on different chromosome(s), a situation that so far remains unusual. The mechanism involved has given rise to the largest protein family in yeasts as mentioned above. BLASTn searches indicated that coding and intergenic regions of duplicated TGAs are highly conserved at the nucleotide level, suggesting propagation of ancestral tandems by segmental duplication at ectopic positions (Figure 2 and Additional file 11).

### Mating genes

**Figure 3:**
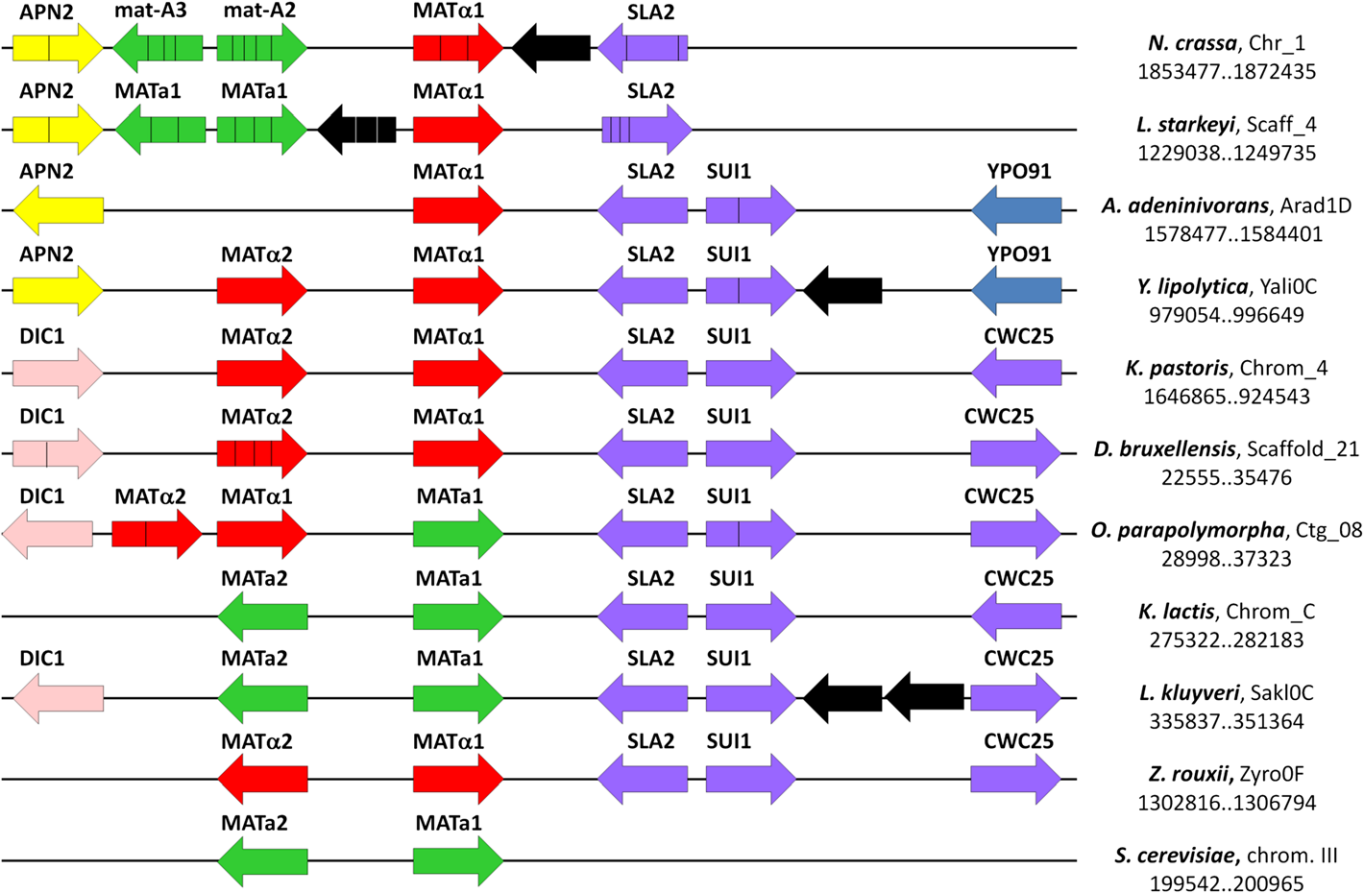
***MAT *locus of *A. adeninivorans *in comparison to other ascomycetes.** Conserved genes are depicted by boxed arrows with the same colour, MATα genes are in red and MATa genes in green. Genes without any homologs at the locus are represented by black boxed arrows. Thin lines (black or white) in genes correspond to the relative position of introns, assuming that the scheme is not to scale. Mating-type locus of CTG species are strongly rearranged, thus not represented here (see [40] for additional information on this clade).

*A. adeninivorans* LS3 is only known to reproduce asexually [20], yet a *MAT* locus was identified on chromosome D as is the case in many asexual species [40]. The region around the mating type locus is conserved between *Y. lipolytica* and *A. adeninivorans*, while it is rearranged in basal species such as *Lipomyces starkeyi,* filamentous fungi, and in species that emerged later, such as *K. pastoris* or *K. lactis* (Figure 3). The *MAT* locus encodes a homolog of the transcriptional factor Matα1 present in other yeast species (ARAD1D19294g, *MTAL1*), with a canonical DNA binding domain and a C-terminal extension partially conserved in *Y. lipolytica*, but absent from other species (Additional file 12). There is no Matα2 coding sequence *(MTAL2*) contrary to the situation reported in other heterothallic yeast species such as *S. cerevisiae* and *Y. lipolytica*. The presence of only *MTAL1* at the *MATalpha* locus is, however, found in several sexually competent filamentous fungi and yeasts such as *Aspergillus nidulans*, *Clavispora lusitaniae, Meyerozyma guillermondii, Scheffersomyces stipitis* or *D. hansenii*[40]. Whether *A. adeninivorans* is asexual or not is still an open question. Either *A. adeninivorans* is truly asexual and the loss of *MTAL2* may be the cause, or alternatively, *A. adeninivorans* is sexual but strains of the opposite mating type have not yet been identified, thus preventing successful mating.

A search for genes involved in mating, meiosis and sporulation in *S. cerevisiae* identified the presence of most genes conserved in the sexual species *D. hansenii, K. pastoris* and *Y. lipolytica* (Additional file 13). For example, out of 368 genes tested, 292 were conserved in *Y. lipolytica* and 288 in *A. adeninivorans*. Candidates for the mating pheromones MFa and MFα and of their cognate receptors as well as for the signaling cascade were identified, confirming that *A. adeninivorans* is either still sexually active or has lost this ability only recently (Additional file 14).

### Metabolic pathways

*A. adeninivorans* is described as having a wide substrate spectrum that includes the assimilation of many nitrogenous and aromatic compounds such as nitrate and nitrite, purines, tannins and benzoic acid derivatives [13,41,42]. The ability to degrade purine compounds is reported in all kingdoms and can occur either aerobically or anaerobically in separate pathways. In the aerobic pathway, the critical step in the degradation of purine bases is the oxidation of hypoxanthine and xanthine to uric acid, catalyzed by xanthine oxidase and/or dehydrogenase. The various purine-degradative pathways are unique and differ from other metabolic pathways because they may serve quite different purposes, depending on the organism or tissue. While some organisms degrade the naturally occurring purines to CO_2_ and ammonia, others contain only some of the steps of the purine degradation pathways, resulting in partial degradation of purines or certain intermediary catabolites [43].

**Figure 4:**
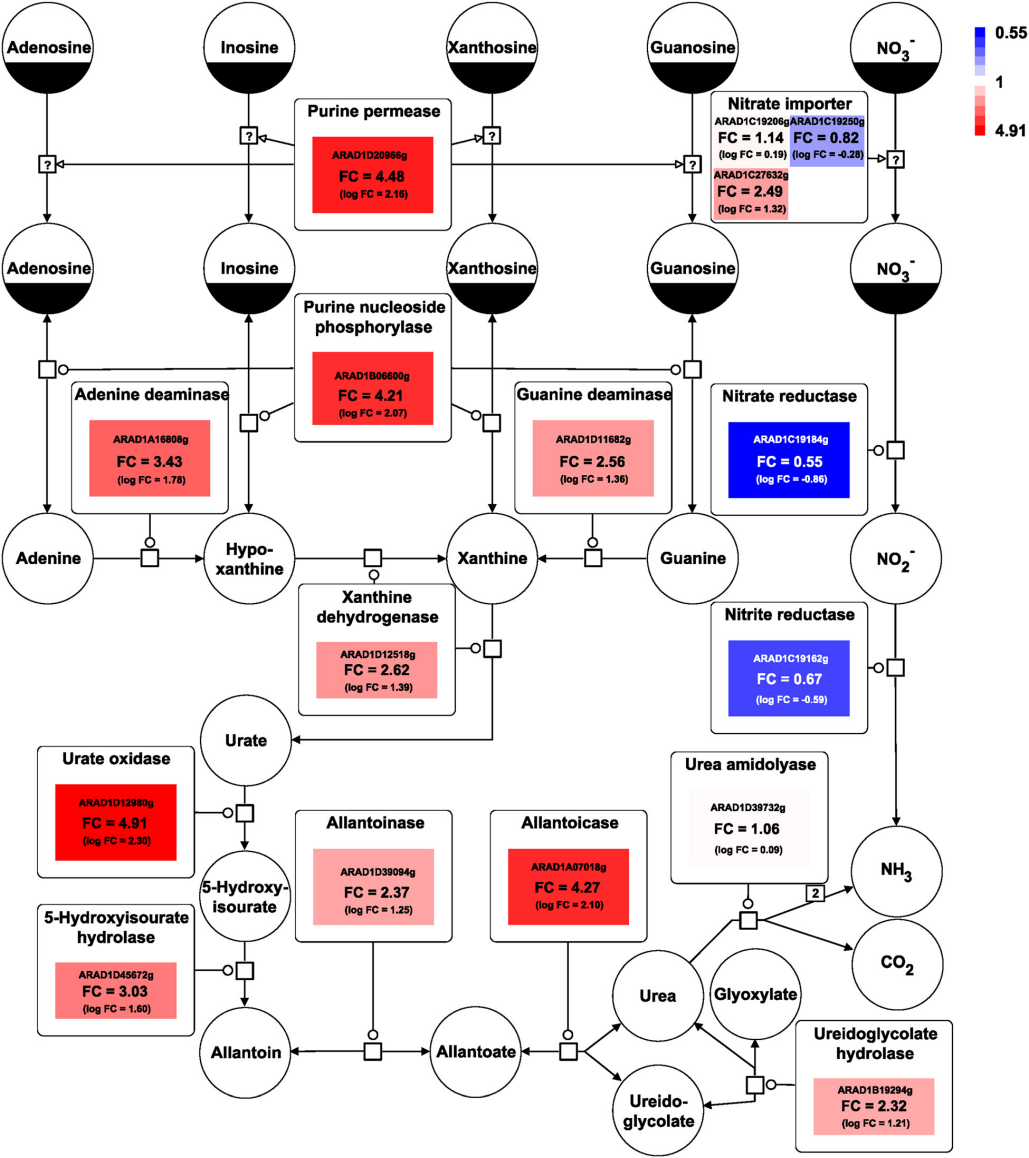
**Scheme of the key components of the purine degradation pathway.** The image shows the reversible (double headed arrow) and irreversible (single headed arrow) reactions catalyzed by the corresponding enzymes (rectangular square) for purine degradation. The colors represent up regulation (red) and down regulation (blue) of genes in cells shifted to medium containing adenine as the sole nitrogen source compared to cells grown with nitrate. Black marked symbols indicate intermediates occurring several times in the pathway. Fold change (FC) values of gene expression are given within the colored boxes.

Purine catabolism is a characteristic feature of *A. adeninivorans*[13]*.* The purine nucleosides (adenosine, inosine, xanthosine and guanosine) are transported across the membrane and into the cytoplasm by a purine permease. They are then converted to adenine, hypoxanthine, xanthine and guanine, further degraded to uric acid and, after transport into the peroxisomes, to urea. All corresponding genes of this pathway are localized on different chromosomes and are induced by adenine and other pathway intermediates [4,44]. Interestingly, an adenosine deaminase, needed to transform adenosine to inosine in animals and human, is absent (Figure 4). This pathway allows *A. adeninivorans* to use all of these purine derivatives as nitrogen and carbon sources [4,44].

As in *Ogatea (Hansenula) polymorpha*[45] and in *Kuraishia capsulata*[18], a cluster of genes encoding a nitrate transporter, a nitrate reductase and a nitrite reductase has been previously identified in *A. adeninivorans*[46]. Genome data indicate that nitrate transporter encoding genes form a three member family, two of which are part of the nitrate cluster.

Microarrays were designed based on the complete genome data of *A. adeninivorans* to analyze gene expression changes before and after a shift from yeast minimal medium (YMM) + 2% glucose with NaNO_3_ to YMM medium with adenine as the nitrogen source. A significant down regulation of the genes involved in nitrate metabolism was observed two hours after the shift. Key components of the purine degradation pathway on the other hand, clearly showed an increased activity (Figure 4). This provides further insight into the regulation of purine degradation by *A. adeninivorans* and emphasizes the possibility of using transcriptomic approaches to identify candidate genes for new biotechnological applications. *Arxula* specificities of the purine degradation pathway include the regulation of the respective genes. Activity tests, qRT-PCR experiments and microarray assays with xanthine dehydrogenase inducers demonstrated strong gene inducibility when cells were cultured on hypoxanthine and adenine and a lower level of induction with uric acid as the sole nitrogen source. However, enzyme induction by purines stops after supplementing the medium with NH_4_^+^ or NO_3_^-^ as nitrogen sources, which is in contrast to the situation in *N. crassa* where the enzyme is induced in the presence of NO_3_^-^, but not with NH_4_^+^. It is known that in *A. nidulans,* NH_4_^+^ inactivates the GATA factor AreA, which is responsible for expression of the urate-xanthine transporter [47]. It is not clear which mechanism triggers the repression with NH_4_^+^ and NO_3_^-^ in *A. adeninivorans*[4].

Tannin, a plant polyphenol molecule, is widely distributed in the plant kingdom where it protects plants against attack by parasites and herbivores. It inhibits the activity of enzymes by binding and precipitation and is to a greater or lesser extent recalcitrant to biodegradation [48]. While tannins are growth inhibitors for most microorganisms, a few bacteria, fungi and yeast such as *D. hansenii, Mycotorula japonica* or *Candida* sp. are capable of exploiting tannins as a carbon and/or energy source for growth [49-51]. *A. adeninivorans* is one of these yeasts that use tannic acid and gallic acid as carbon sources [52]. Genes encoding tannases (*ATAN1 -* ARAD1A06094g, *ATAN2 -* ARAD1A19822g)*,* gallate decarboxylase (*AGDC -* ARAD1C45804g) and catechol 1,2 dioxygenase (*ACDO -* ARAD1D18458g) have been identified and His-tagged recombinant enzymes and corresponding gene mutants were used to confirm the activity of these enzymes (data not shown). This demonstrated that the tannic acid catabolism pathway enables this yeast to assimilate tannic acid and other hydroxylated derivatives of benzoic acid by non-oxidative decarboxylation. All suitable derivatives require an hydroxide group at the *m* or *p* position of the carboxylic acid (Additional file 15). Interestingly, *A. adeninivorans* is thus the first eukaryote known to synthesize two tannases, one extracellular (Atan1p) [32] and one cell-wall localized (Atan2p - data not shown) which permits effective degradation of extracellular tannic acid. Both enzymes are able to remove gallic acid from both condensed and hydrolysable tannins. Substrate specificity, biochemical parameters (temperature optimum 35 to 40°C, pH optimum at ca. 6.0) and nearly complete extracellular localization (≥97%) distinguish Atan1p as an important industrial enzyme. First, transgenic tannase producer strains were constructed with a constitutively expressed *ATAN1* module integrated into a chromosome. In fed-batch fermentation experiments, the transgenic strain produced 51,900 U/L of tannase activity after 42 h with a dry cell weight of 162 g/L [1].

**Figure 5:**
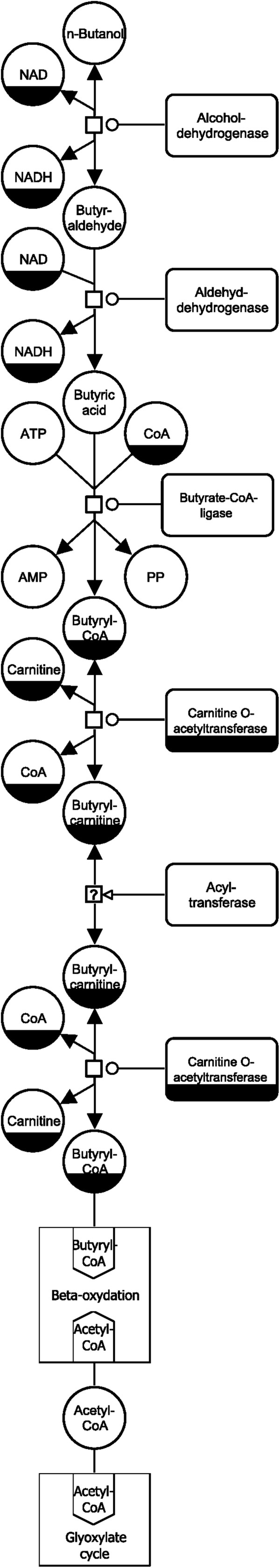
**Schematic overview of the n-butanol degradation pathway in *A. adeninivorans*.***Arxula* is able to use n-butanol as the sole carbon and energy source, by converting it into the central metabolite acetyl-CoA by ß-oxidation, to finally generate succinate in the peroxisomes. A genome-mining approach led to the proposal of the pathway shown here. The figure shows the reversible (double headed arrow) and irreversible (single headed arrow) reactions catalyzed by the corresponding enzymes (rectangular square) and the cofactors (ATP/AMP, NAD+/NADH) necessary for n-butanol degradation. Black marked symbols indicate intermediates occurring several times in the pathway. AMP, Adenosine monophosphate; ATP, Adenosine triphosphate; CoA, coenzyme A; NAD, Nicotinamide adenine dinucleotide; PP, phosphate.

Another uncommon substrate used by this yeast is n-butanol. The n-butanol degradation pathway has not previously been reported to exist in eukaryotes. Genome mining suggests that n-butanol is oxidized to butyraldehyde by an alcohol dehydrogenase (Aadh1p, *AADH1 -* ARAD1B16786g) that has a high substrate specificity, and then to butyric acid by two aldehyde dehydrogenases (Aald2p, *AALD2 -* ARAD1B17094g; Aald5p, *AALD5 -* ARAD1C17776g). The last steps involve an acyl-CoA ligase, a cytoplasmic acyl-CoA carnitine acyltransferase and a peroxisomal acyl-CoA carnitine acyltransferase for butyryl-carnitine synthesis via a butyryl-CoA intermediate that is transported from the cytoplasm to peroxisomes or mitochondria for ß-oxidation. A special feature of this pathway is that the synthesis of butyryl-CoA from butyric aldehyde is a one-way reaction since the aldehyde dehydrogenase and acyl-CoA ligase steps are not reversible (Figure 5).

## Conclusion

The complete sequence of *A. adeninivorans* nuclear and mitochondrial genomes has been provided. High-quality genomes in early-diverging *Saccharomycotina* are scarce and that sequence will allow further fundamental studies on comparative genomics, evolution and phylogenetics. It will also allow the deciphering of a new mechanism of genome modeling through TGA duplication. *Arxula* is able to assimilate a wide spectrum of C and N-sources, which includes not only conventional substrates such as glucose, xylose, and starch but also rarely metabolized substances as n-butanol, tannic acid and protocatechuate. Sequencing its genome revealed protein components of these pathways, which had previously remained unexplored in yeast, offering clues for further biotechnological developments. In the course of identifying alternative microorganisms for biotechnological interest, *A. adeninivorans* has already proved its competitiveness in white biotechnology, and is further strengthened as a promising cell factory for many more applications.

## Materials and methods

### Genome sequencing and assembly

The genome of *A. adeninivorans* LS3 was sequenced independently by the Genoscope (Evry, France) using the capillary Sanger technology and by IPK (Gatersleben, Germany) using the 454 Roche methodology (GS-FLX Titanium version).

For the Sanger technology, a shotgun sequencing strategy using three different clone libraries and capillary Sanger sequencing was used to obtain a 12× coverage of the complete genome. For two of three libraries, genomic DNA was fragmented by mechanical shearing and 3 kb (A) or 10 kb (B) inserts were respectively cloned into pcdna2.1 (Invitrogen, Saint Aubin, France) and pCNS (pSU18 derived) plasmid vectors. In addition, a large insert (25 kb) BAC library (C) was constructed from *Sau*3A partial digest and cloned into pBeloBAC11. Vector DNAs were purified and end-sequenced (124,032 reads (A), 61,440 reads (B), 5,376 reads (C)) using dye-terminator chemistry on ABI3730 sequencers. The reads were assembled using the whole genome shotgun assembler ARACHNE and the chromosome sequences were individually reassembled using the Phred/Phrap/Consed software package. For the finishing step, we used primer walking of clones, PCR amplifications and *in vitro* transposition technology [Template Generation System™ II Kit (Fisher Scientific, Illkirsh, France) or Hypermu < Kan-1 > (Tebu-Bio, Le Perray-en-Yvelines, France), corresponding to 814, 33 and 17,975 reads, respectively. The final assembly consisted of four scaffolds larger than 1 Mb, hereafter referred to as chromosomes, and nine shorter contigs of a size ranging from 4 to 120 kb, including four mitochondrial scaffolds. Four of the remaining contigs were later incorporated at chromosome ends in the final assembly using data obtained from the 454 assembly. The mitochondrial genome sequence was assembled as a circular map using Sanger and 454 contigs and manually validated using single reads obtained with the Sanger technology. *A. adeninivorans* genome sequence data have been deposited at EMBL under the accession number PRJEB4557 [EMBL:PRJEB4557].

The shotgun library of *A. adeninivorans* for sequencing on Roche 454 GS FLX Titanium sequencer was prepared using 5 μg of genomic DNA. Based on random cleavage of the genomic DNA with subsequent removal of small fragments with Agentcourt AMPure SPRI beads (Beckman Coulter, Krefeld, Germany), the resulting single stranded DNA (ssDNA) library showed a fragment distribution between 300 and 1000 bp. The optimal amount of ssDNA library input for the emulsion (emPCR) was determined empirically through 4 small-scale titrations with one, two, four and eight copies per bead (cpb). Finally, one cpb was used for the large-scale experiment. One individual emulsion PCR (two cups, one full emPCR-Kit LV (Roche Applied Science, Mannheim, Germany) was carried out to generate 5.7 million DNA-carrying beads for two-region sized 70 × 75 PicoTiterPlates (Roche Applied Science, Mannheim, Germany) and each region was loaded with 2 million DNA-carrying beads. Two read sets were thus generated totaling 1,074,025 reads. This resulted in 542.3 Mb of sequence data (45-fold genome sequence coverage) with an average read length of 505 bp. Assembly was performed using the Newbler software (v2.3) within the Roche 454 suite package, MIRA multi-pass DNA sequence data assembler/mapper (v3.0.2) and CLC Bio assembler. To allow comparisons between the assemblies of different assembly programs, singletons and contigs shorter than 100 bp were discarded before subsequent analysis. Standard metrics describing the assembly, such as the total bases used in a assembled contigs, the amount of contigs longer than 300 bp, 500 bp, 1 kb, 2 kb and 5 kb, number of base pairs in the largest contig and N50 contig length (the smallest contig size in which half the assembly is represented) were used to compare the assembly programs. The highest number of contigs was produced by MIRA but only 161 contigs were longer than one kilobase pair. While Newbler and CLC Bio assemblers constructed longer contigs, however the longest contig was generated by MIRA (Additional file 1).

### Mapping of GS FLX shotgun reads and contigs to assembled chromosomes

To assess the quality of the final assembled genome, the 454 reads were mapped onto the chromosomes using the Burrows-Wheeler Alignment tool BWA [53]. Two statistics were extracted from the mappings using Samtools [54]: the percentage of reads that mapped on the assembly and the percentage of reads that mapped to each chromosomes. The quality of the final assembled genome was estimated using the dot-plot analysis which was performed using Nucmer software (NUCleotide MUMmer v3.1 [55]). The dot-plot alignment was generated by comparison of all assembled chromosomes and contigs.

Mapping of 454 reads was used to estimate the gene copy number by computing the number of tags mapping to unique regions of the genome. For this purpose, sequences of 21 *A. adeninivorans* genes, deposited in GenBank (NCBI), were used in BLASTn searches together with the set of all 454 reads using three BLAST e-value cutoff = e-10, e-50 and e-100 to improve search specificity. The analysis of the gene copy number was performed using the formula: GeneCopyNumber = (Number of BLASTn hits * Average read length)/(Gene length * 454 sequence coverage).

### Genome annotation

Non-protein coding and protein-coding gene models were predicted according to Louis *et al.*[56]. All translations of models longer than 80 codons were compared against the proteomes of *Y. lipolytica* and *S. cerevisiae* as well as Uniprot-Fungi using BLASTp. In addition, the gene models were compared to position-specific scoring matrix (PSSM) representative of Genolevures protein families [57] with PSI-tBLASTn (Position-Specific Iterated BLAST). Pre-annotated gene models were then examined for validation in the framework of the Génolevures proprietary Magus annotation system by a community of curators, in three phases: (i) curation of models with PSI-tBlastn hits, as possible new members of protein families, for homogeneity of annotation, (ii) curation of other models, (iii) final finishing through contig walk by a single curator in charge. At any phase, curators could add or modify gene models.

Circos [58] was used for illustration of nuclear genome data such as: chromosome structure (position of centromeres, tRNA and rRNA genes), density of genes across the genome, content of repeat DNA, 454 reads mapped to chromosomes, syntenic blocks between *A. adeninivorans* and genomes of *Y. lipolytica*, *K. pastoris* and *S. cerevisiae*.

Functional annotation of genes according to the GO terms, EC numbers and the KEGG pathway were performed for each *A. adeninivorans* CDS using the Blast2GO software suite. Protein domains were detected by InterProScan with various databases (BlastProDom, FPrintScan, HMM-PIR, HMM-Pfam, HMM-Smart, HMM-Tigr, PatternScan, SuperFamily, HMM-Panther and Gene3D) through the European Bioinformatics Institute Web Services. Signal peptide and transmembrane helices were predicted by SignalP v.3.0′s neural network and hidden Markov model tools [59] and TMHMM, respectively.

### Protein families

The classification of *A. adeninivorans* protein sequences into protein families was performed along two procedures. First, protein sequences were tentatively incorporated into protein families defined in the previous round of Génolevures genome annotation using PSI-BLAST with relaxation factors based on family dispersion [30]. Second, the sequences rejected by the procedure were pooled with the sequences of the nine species already present in the Génolevures database which are not members of any protein family and a clustering with OrthoMCL [60] was applied to define new families.

### Phylome reconstruction

The phylome, a complete collection of phylogenetic trees for each gene in *A. adeninivorans,* was reconstructed. Seventeen additional species were included in the phylome: three Pezizomycotina species and fourteen Saccharomycotina species. The phylome was reconstructed using a previously described pipeline [35]. Briefly, for each gene encoded in *A. adeninivorans*, a BLAST search was performed against the proteome database containing the 18 proteomes. Results were filtered according to e-values < 1e-05 and minimal overlaps with hit sequences at 50% of the query length. A maximum of 150 matches were accepted for each *A. adeninivorans* protein. Multiple-sequence alignments were performed in forward and reverse orders [61], using three programs: Muscle [39] v3.8.31, MAFFT v6.814b [62] and DIALIGN-TX [63]. The six resulting alignments were then combined using M-COFFEE (T-Coffee v8.80) [64] and trimmed using trimAl [65] v1.3 (consistency cutoff: 0.1667; gap score cutoff: 0.1). Model selection for phylogenetic tree reconstruction was performed by reconstructing neighbour joining trees using BioNJ [66] with different models (JTT, WAG, MtREV, VT, LG, Blosum62, CpREV and DCMut) and then the two best models according to the AIC criterion [67] were chosen. The selected models were used to reconstruct maximum likelihood trees using phyML [68]. In all cases, a discrete gamma-distribution model with four rate categories plus invariant positions was used, the gamma parameter and the fraction of invariant positions were estimated from the data. A total of 4,992 trees were reconstructed. Trees and alignments are stored in the database phylomeDB with the PhyID code 178.

### Phylome analysis

The trees reconstructed in the phylome were analyzed using ETE [69] v2.0. Orthology and paralogy relationships between the sequences were established using the species overlap algorithm from ETE v2.0. The algorithm scans the trees from seed to the root and at each node it establishes a duplication or a speciation node depending on the overlap between the species located at each side of the node. If there are common species, the node is assumed to be a duplication node, otherwise it is considered a speciation node. Once duplications were detected, they were mapped onto the species tree. It was assumed that the duplication occurred at the common ancestor of the species derived from the duplication node. The duplication rate at each node was calculated by dividing the duplications mapped at a given node by the number of trees that have an outgroup to the node. Species-specific expansions were also detected by selecting those duplication nodes that only contained sequences from *A. adeninivorans*. Groups of expanded proteins that overlapped in more than 20% of their sequences were fused into a single-gene expansion.

### Detection of horizontally acquired genes

Gene transfers from prokaryotes to *A. adeninivorans* were detected using a previously published pipeline [36]. Briefly, a BLAST search was performed for each protein encoded in *A. adeninivorans* against a database that contained 102 completely-sequenced fungi (downloaded from their respective databases), 95 other eukaryotes and 1,395 prokaryotes (downloaded from KEGG as of June 2011). Only genes present in more than 30 prokaryotes, less than 10 fungi and no other eukaryotes were considered to be putative transfers.

### Species phylogeny

The species tree was reconstructed by concatenating 253 genes that were found in all the genomes included in the phylome database and that were exclusively one-to-one orthologs. The genes were concatenated and the tree was reconstructed using RaxML [70]. A second tree was reconstructed using a super-tree approach as implemented in Duptree [71], this algorithm tries to find the species tree that minimizes the number of duplication events that occurred in a set of gene trees. In this case the 4,992 trees reconstructed in the phylome were used.

### Microarray design and hybridization for gene expression analyses

Based on 6,025 annotated chromosomal sequences and 36 putative mitochondrial genes oligos were designed using Agilent Technologies eArray software (design number 035454). Depending on the sequence length of the genes up to ten 60-mers per gene were created resulting in a total of 56,312 *A. adeninivorans* specific oligos. The microarray was produced by Agilent Technologies (Böblingen, Germany) in 8×60k format.

Overnight cultures of *A. adeninivorans* LS3 in YMM with nitrate were shifted to YMM containing 4 mM adenine as the sole nitrogen source and YMM with nitrate as a control, respectively. After 2 h of shaking at 30°C and 180 rpm cells were harvested and total RNA was isolated. Probe labeling and microarray hybridization (duplicates) were executed according to the manufacturer’s instructions (Agilent Technologies “One-Color Microarray-Based Gene Expression Analysis”; v6.5; Böblingen, Germany).

Analysis of microarray data was performed with the R package limma [72]. Raw expression values were background corrected (method “normexp”) and normalized between arrays (method “quantile”). Differentially expressed genes were detected by fitting a linear model to log2-transformed data by an empirical Bayes method [73]. The Bonferroni method was used to correct for multiple testing.

### Accession numbers

*A. adeninivorans* genome sequence data have been deposited at EMBL under the accession number PRJEB4557 [EMBL:PRJEB4557]. The raw data of 454 reads have been deposited at EMBL/ENA database under the accession number ERP001774 [EMBL: ERP001774].

## Abbreviations

BLAST: Basic Local Alignment Search Tool; bp: base pair; CDS: Coding DNA sequence; CoA: Coenzyme A; EC: Enzyme commission number; GO: Gene ontology; kb: kilobase; KEGG: Kyoto encyclopedia of genes and genomes; PCR: Polymerase Chain reaction; PSSM: position-specific scoring matrix; qRT-PCR: Quantitative reverse transcriptase PCR; snRNA: small nuclear ribonucleic acid; snoRNA: Small nucleolar ribonucleic acid; TGA: Tandem gene array; YMM: Yeast minimal medium.

## Competing interests

The authors declare that they have no competing interests.

## Authors’ contributions

All authors contributed substantially to the conception or design of the different parts of the work, within the Génolevures consortium or at the IPK. More, their practical contributions were the following: VB, BV, CJ and PW carried out the genome sequencing at CNS-Genoscope, NS designed and supervised the 454 project at the IPK, MC assembled the 454 data and participated to the gap closure, SB mapped the different genome versions, PPG assisted in the visualization of the nuclear genome, PD, TM, DJS, AS, GS developed the annotation tools, J-LS coordinated the genome annotation by the Génolevures consortium, JAC and EW identified the ncRNA, CM analyzed the tRNA content, EB analyzed the rDNA copy number, PB, CB, SC, LD, CF, PJ, IL, VLL, ML, GM, GFR, CS, JS, M-LS, AT, J-LS, BD, CG, CM and CN curated manually the genome annotation, CN and CG annotated and analyzed the mitochondrial genome, TG and MM-H carried out the phylogenomics studies, TG, MM-H, CG and ET analyzed the protein families, AG and CG analyzed the transporter families, MC and US performed the blast2GO studies, EB and MG analyzed the tannic acid degradation pathway, UH and JR analyzed the n-butanol degradation pathway, AT-S, KB and DJ analyzed the purine degradation pathway, AH mapped the data onto metabolic pathways and created the metabolism figures, MM and SW analyzed the microarray data, RB supervised the biochemical work of the IPK group, US supervised the bioinformatics work of the IPK group, GK designed and supervised the *Arxula* work of the IPK group, CN, MC, CG, US and GK finalized the manuscript from drafts of all co-authors. All authors read and approved the final manuscript.

## Supplementary Material

Additional file 1 Assemblies of 454/Roche data. Click here for file

Additional file 2 **Mitochondrial features of *A. adeninivorans.***
Click here for file

Additional file 3 Non coding RNAs. Click here for file

Additional file 4 Centromeres. Click here for file

Additional file 5 Functional annotation. Click here for file

Additional file 6 Spliceosomal introns. Click here for file

Additional file 7 **Phylogeny and synteny between *A. adeninivorans *** **and other yeasts.** Click here for file

Additional file 8 Proteins having homologs only in Pezizomycotina or bacteria. Click here for file

Additional file 9 **Gene families amplified in *A. adeninivorans. ***
Click here for file

Additional file 10 Transporter distribution in hemiascomycete species. Click here for file

Additional file 11 **Tandem gene arrays in *A. adeninivorans.***
Click here for file

Additional file 12 Mating type locus. Click here for file

Additional file 13 Homologues of genes involved in mating, meiosis and sporulation. Click here for file

Additional file 14 Mating pheromones. Click here for file

Additional file 15 **Tannic acid degradation pathways in *A. adeninivorans.***
Click here for file
