# Supplementary material for: The complete genome of Blastobotrys (Arxula) adeninivorans LS3 - a yeast of biotechnological interest
Source: Biotechnol Biofuels. 2014 Apr 24;7:66. doi: 10.1186/1754-6834-7-66 (PMC4022394; doi:10.1186/1754-6834-7-66)

Additional File 1. Assemblies of 454/Roche data

Assembly of 454 reads

Table S1A Basic assembly metrics of 454 reads.

Figure S1B Cumulative contig numbers for different assemblies of 454 reads.

Figure S1C Coverage rate of total assembled bases by different size contigs.

Mapping of 454 reads and contigs to assembled chromosomes derived from Sanger sequences

Table S1D Alignment between 454 reads and assembled chromosomes.

Figure S1E Coverage ratio of 454 reads and assembled chromosomes.

Assembly of 454 reads

Table S1A Basic assembly metrics of 454 reads

	MIRA	Newbler	CLC Bio
Number of contigs*	1,547	223	1,050
Total bases	12,533,674	11,775,502	13,210,769
Number of contigs >= 1 kbp	161	143	864
Total bases in contigs >= 1 kbp	11,830,569	11,742,937	13,131,111
Max contig length	425,767	390,644	107,436
Number of reads in the longest contig	37,081	34,325	9,314
Mean contig length	8,150	52,805	12,582
N50	142,195	154,801	26,719

*Only contigs > 100 bp

Figure S1B Cumulative contig numbers for different assemblies of 454 reads

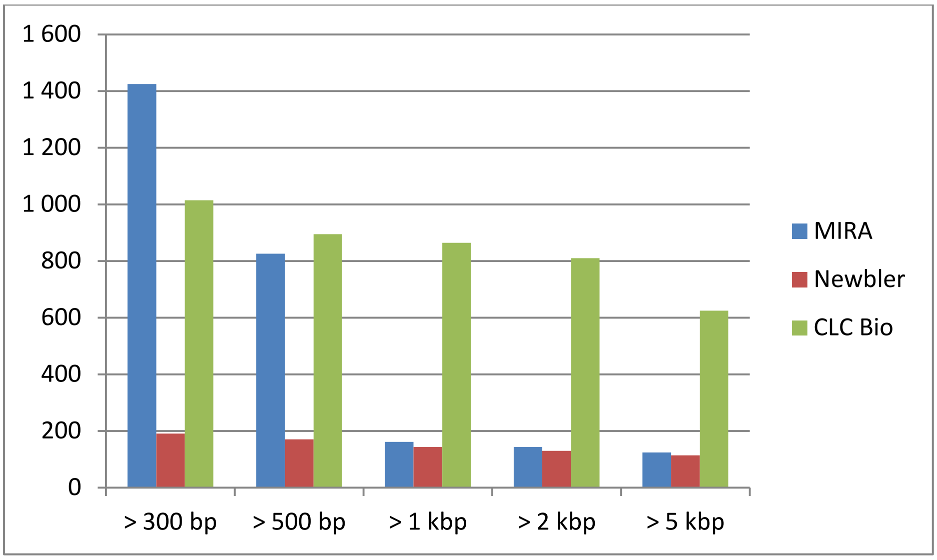

Figure S1C Coverage rate of total assembled bases by different size contigs

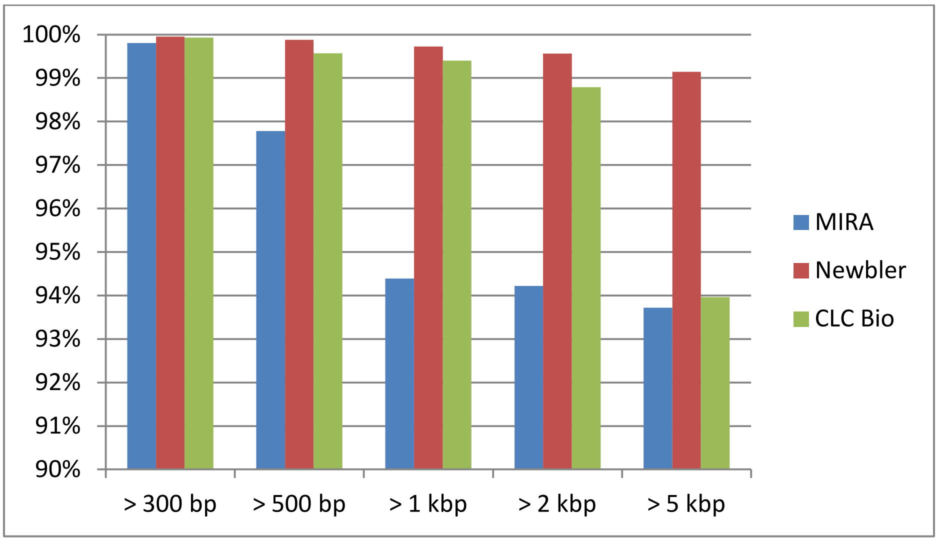

Mapping of 454 reads and contigs to assembled chromosomes

Table S1D Alignment between 454 reads and assembled chromosomes

454 reads	Number	Average lenght [bp]	Total bases	[%]
Aligned to chromosomes	1,056,280	503	531,741,216	98,3%
Not aligned to chromosomes	17745	601	10,657,239	1,7%
Total	1,074,025	505	542,398,455	100%

Figure S1E Coverage ratio of 454 reads and assembled chromosomes

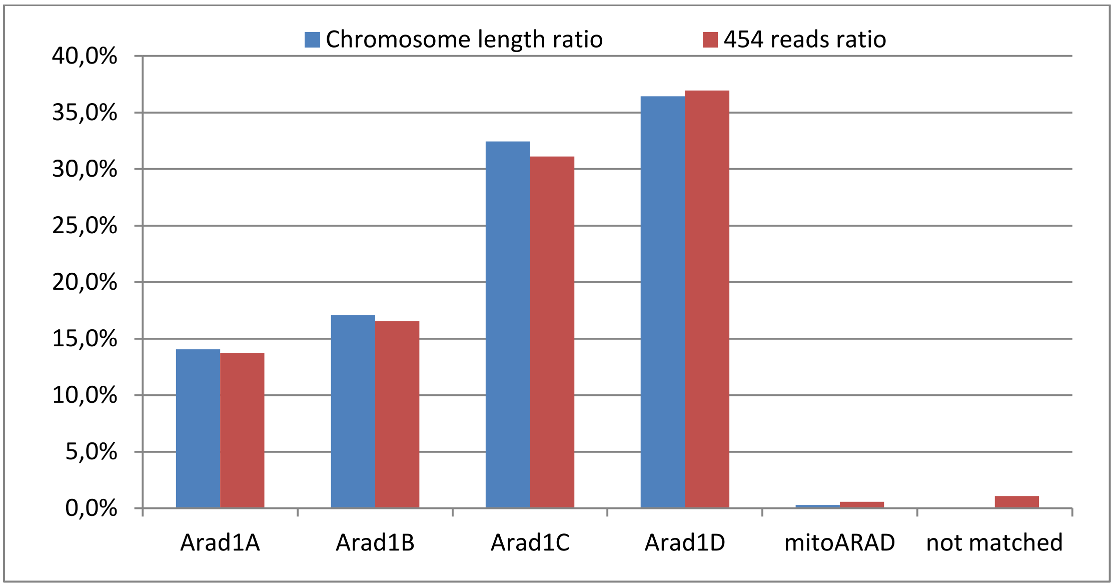

Supplement: Additional file 1 — Assemblies of 454/Roche data. [file 1754-6834-7-66-S1.pdf]
