# Supplementary material for: The complete genome of Blastobotrys (Arxula) adeninivorans LS3 - a yeast of biotechnological interest
Source: Biotechnol Biofuels. 2014 Apr 24;7:66. doi: 10.1186/1754-6834-7-66 (PMC4022394; doi:10.1186/1754-6834-7-66)

Additional File 2. Mitochondrial features of *A. adeninivorans*

Figure S2A Circular map of *A. adeninivorans* mitDNA.

Gene annotation relied on similarity searches with different mitochondrial sequences of hemiascomycetous yeasts, using the mold mitochondrial genetic code for protein-coding genes (UGA for Trp). Genes encoding tRNA-molecules were predicted using tRNAscan-SE [1]. Protein coding genes are indicated by light blue arrows, intronic ORFs by dark green arrows, introns by light grey boxes, rDNA by dark blue arrows, tRNAs by light green arrows with charged amino acid indicated in one letter code. Putative terminators are depicted by purple bars.

Table S2B Codon usage in mitochondrial CDS.

For each amino acid (Aa) encoded by a given codon (Codon), the number of occurrences of this codons is shown (Nb) as well as the cognate tRNA designated by its anticodon (AC).

Figure S2C Intron structure.

Proposed structure of the two group 1D introns present in *cob*; loops encoding the intronic ORFs are omitted.

Figure S2A

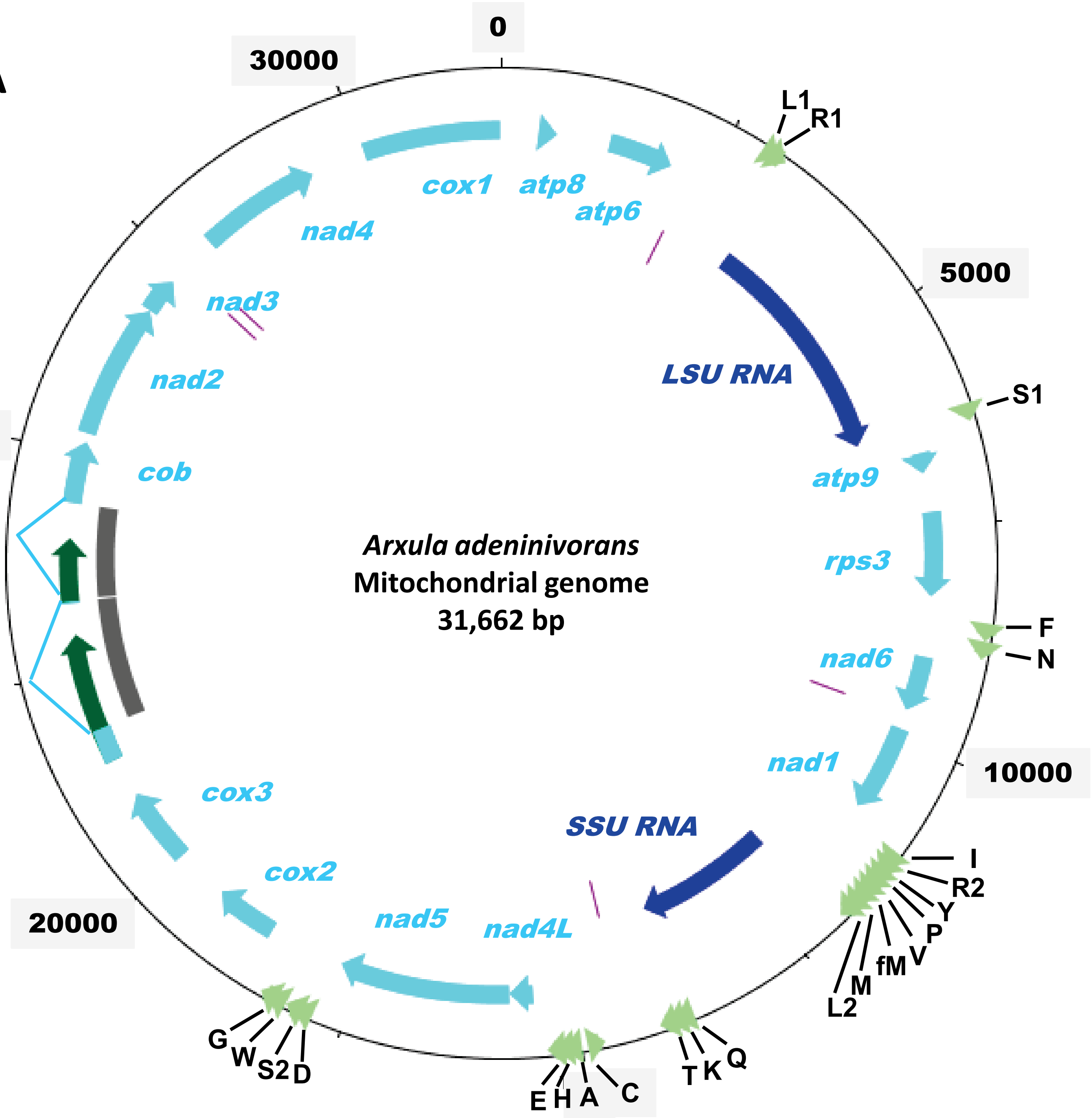
0
5000
10000
20000
25000
30000
L1
R1
S1
F
N
I
R2
Y
P
V
fM
M
L2
Q
K
T
C
A
H
E
D
S2
W
G
cox1
atp8
atp6
LSU RNA
atp9
rps3
nad6
nad1
SSU RNA
nad4L
nad5
cox2
cox3
cob
nad2
nad3
nad4
Arxula adeninivorans
Mitochondrial genome
31,662 bp

Table S2B Codon usage in mitochondrial CDS

For each amino acid (Aa) encoded by a given codon (Codon), the number of occurence of this codons is shown (Nb) as well as the cognate tRNA designated by its anticodon (AC).

Aa	Nb	Codon	AC	Aa	Nb	Codon	AC	Aa	Nb	Codon	AC	Aa	Nb	Codon	AC
Phe	226	ttt	---	Ser	64	tct	---	Tyr	393	tat	---	Cys	47	tgt	---
Phe	98	ttc	gaa	Ser	0	tcc	---	Tyr	9	tac	gta	Cys	0	tgc	gca
Leu	751	tta	taa	Ser	161	tca	tga	-	15	taa		Trp	82	tga	tca
Leu	4	ttg	---	Ser	0	tcg	---	-	0	tag		Trp	0	tgg	---
Leu	6	ctt	---	Pro	77	cct	---	His	104	cat	---	Arg	39	cgt	acg
Leu	0	ctc	---	Pro	2	ccc	---	His	2	cac	gtg	Arg	0	cgc	---
Leu	6	cta	tag	Pro	86	cca	tgg	Gln	86	caa	ttg	Arg	0	cga	---
Leu	1	ctg	---	Pro	0	ccg	---	Gln	0	cag	---	Arg	0	cgg	---
Ile	208	att	---	Thr	119	act	---	Asn	430	aat	---	Ser	194	agt	---
Ile	11	atc	gat	Thr	0	acc	---	Asn	10	aac	gtt	Ser	0	agc	gct
Ile	573	ata	---	Thr	174	aca	tgt	Lys	190	aaa	ttt	Arg	85	aga	tct
Met	124	atg	cat	Thr	0	acg	---	Lys	6	aag	---	Arg	2	agg	---
iMet	14	atg	cat												
Val	124	gtt	---	Ala	164	gct	---	Asp	128	gat	---	Gly	214	ggt	---
Val	0	gtc	---	Ala	3	gcc	---	Asp	1	gac	gtc	Gly	2	ggc	---
Val	180	gta	tac	Ala	85	gca	tgc	Glu	126	gaa	ttc	Gly	101	gga	tcc
Val	1	gtg	---	Ala	1	gcg	---	Glu	7	gag	---	Gly	5	ggg	---

Figure S2C

cob-i1

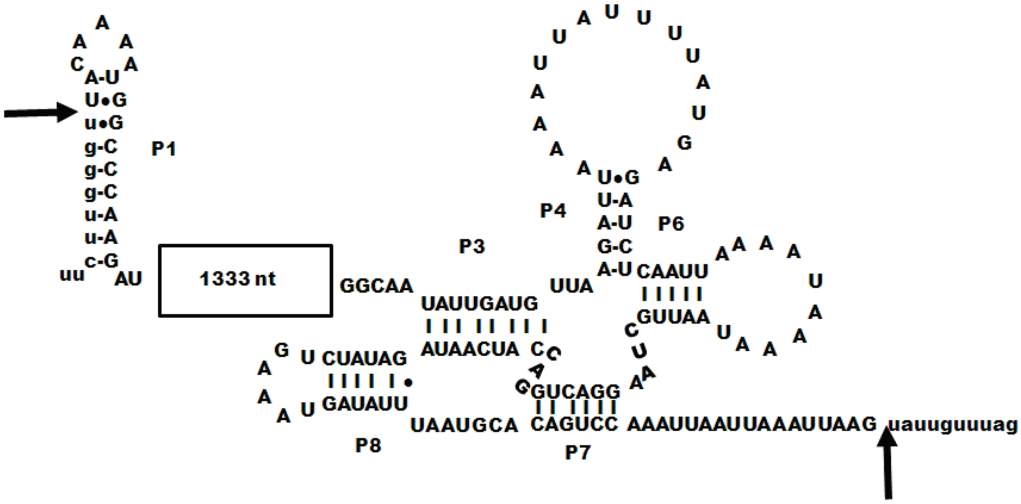

cob-i2

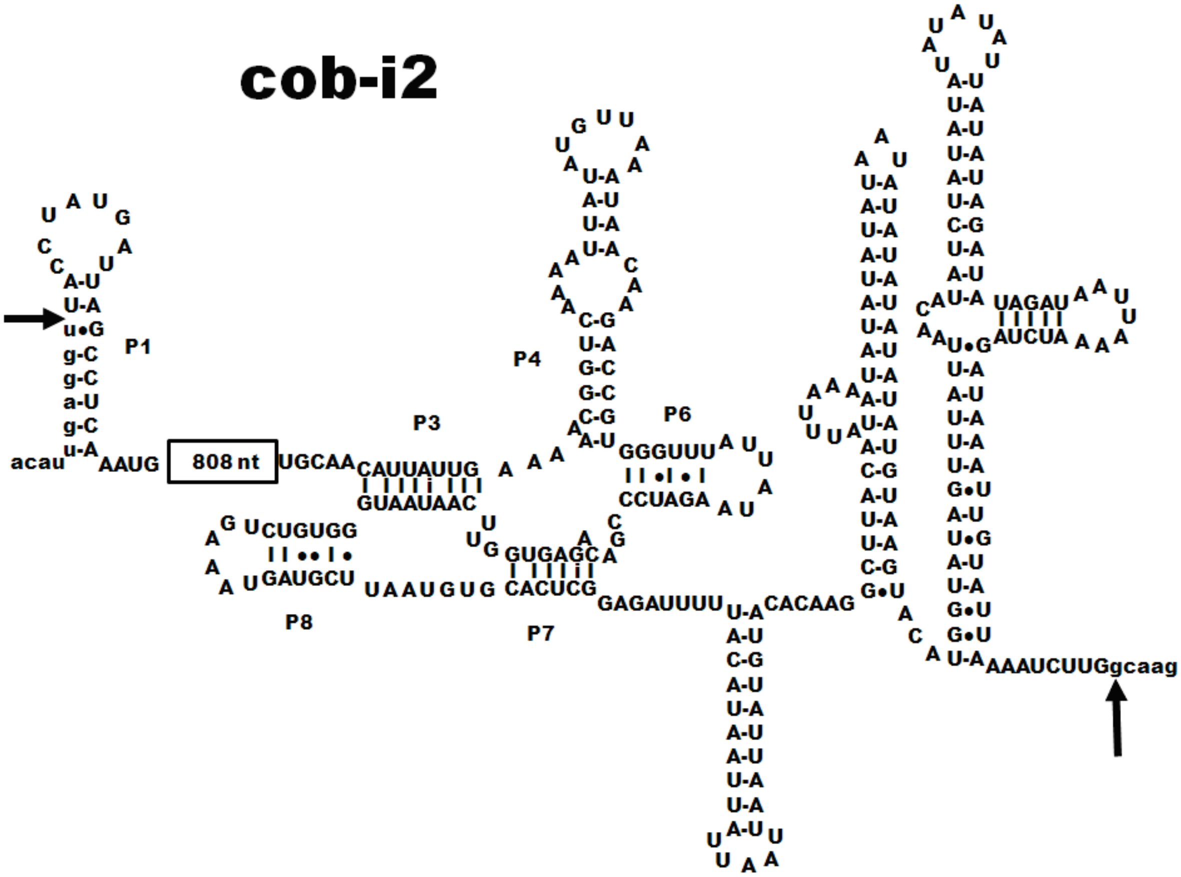

Supplement: Additional file 2 — Mitochondrial features of A. adeninivorans. [file 1754-6834-7-66-S2.pdf]
