# Supplementary material for: The complete genome of Blastobotrys (Arxula) adeninivorans LS3 - a yeast of biotechnological interest
Source: Biotechnol Biofuels. 2014 Apr 24;7:66. doi: 10.1186/1754-6834-7-66 (PMC4022394; doi:10.1186/1754-6834-7-66)
Supplement: Additional file 3 — Non coding RNAs. [file 1754-6834-7-66-S3.pdf]

Additional File 3. Non coding RNAs

Table S3A Codon and tRNA gene usage in *A.adeninivorans* and *Y. lipolytica*.

Genes encoding tRNAs were searched with tRNAScan-SE [1] and sequences were analyzed for possible exceptions to the eukaryotic cloverleaf model, as described in [2]. Initiator Met tRNA genes were distinguished from the elongator sequences thanks to the "GGG" sequence in positions 29 -31.

Table S3B Genes encoding snRNAs and snoRNAs.

Blast [3] and Infernal [4] searches were performed on *A. adeninivorans* genome for snRNAs and snoRNAs detection. For the Blast search, ncRNA sequences from the Genolevures database [5] were used as queries. All hits with an e-value lower than 0.1 were retained for validation. For the Infernal search, covariance models found in the RFam [6] database were used. All hits with an e-value lower than 0.5 were retained for validation. All retained hits were aligned and manually checked. Hits were accepted as candidates if: i) the sequence agrees with known structural features, guiding sequences (for snoRNAs) and conserved sequence motifs for homologous molecules and ii) known synteny was verified.

Table S3A Codon and tRNA gene Usages in *A. adeninivorans* and *Y. lipolytica*

AA		C	AC	ARAD		YALI	
F	Phe	TTT	---	2.14	-	1.58	-
F	Phe	TTC	(GAA)	1.61	4+	2.30	17+
L	Leu	TTA	(TAA)	0.69	1	0.18	1
L	Leu	TTG	(CAA)	1.93	2+	1.04	3+
L	Leu	CTT	(AAG)	1.56	4	1.33	21+
L	Leu	CTC	(GAG)	1.37	-	2.27	-
L	Leu	CTA	(TAG)	0.96	1	0.53	2
L	Leu	CTG	(CAG)	2.41	4	3.36	13+
I	Ile	ATT	(AAT)	3.05	7+	2.26	26+
I	Ile	ATC	(GAT)	1.61	-	2.45	-
I	Ile	ATA	(TAT)	0.42	1+	0.21	1+
M	Met	ATG	(CAT)	1.99	3+	0.21	9
m	iMet	ATG	(CAT)	0.21	3	2.06	9
V	Val	GTT	(AAC)	1.72	5	1.59	24
V	Val	GTC	---	1.46	-	2.16	-
V	Val	GTA	(TAC)	1.28	2	0.40	2
V	Val	GTG	(CAC)	2.23	3+	2.57	8+
S	Ser	TCT	(AGA)	2.35	5	2.19	21
S	Ser	TCC	---	1.57	-	2.07	-
S	Ser	TCA	(TGA)	1.31	1+	0.78	2+
S	Ser	TCG	(CGA)	1.37	2+	1.54	4+
P	Pro	CCT	(AGG)	2.12	5+	1.74	21+
P	Pro	CCC	---	1.34	-	2.34	-
P	Pro	CCA	(TGG)	1.40	2+	0.68	3
P	Pro	CCG	(CGG)	0.56	1+	0.67	2+
T	Thr	ACT	(AGT)	2.19	5	1.64	22
T	Thr	ACC	---	1.77	-	2.58	-
T	Thr	ACA	(TGT)	0.95	1+	1.05	3+
T	Thr	ACG	(CGT)	0.65	1	0.85	2+
A	Ala	GCT	(AGC)	2.68	7	2.55	30
A	Ala	GCC	---	2.32	-	3.28	-
A	Ala	GCA	(TGC)	1.82	2	1.11	4
A	Ala	GCG	(CGC)	0.87	1	0.89	2
Y	Tyr	TAT	---	1.33	-	0.68	-
Y	Tyr	TAC	(GTA)	1.80	5+	2.32	14+
*	Och	TAA	---		-		-
*	Amb	TAG	---		-		-
H	His	CAT	---	1.18	-	0.95	-
H	His	CAC	(GTG)	1.09	4	1.45	12+
Q	Gln	CAA	(TTG)	1.60	1	0.97	3
Q	Gln	CAG	(CTG)	2.71	4	3.22	15+
N	Asn	AAT	---	1.86	-	0.89	-
N	Asn	AAC	(GTT)	2.19	5	3.14	16+
K	Lys	AAA	(TTT)	1.46	2+	1.24	4+
K	Lys	AAG	(CTT)	4.07	8+	4.66	34+
D	Asp	GAT	---	2.91	-	2.16	-
D	Asp	GAC	(GTC)	3.11	6+	3.84	28+
E	Glu	GAA	(TTC)	2.80	2+	1.87	6
E	Glu	GAG	(CTC)	3.52	6	4.65	27+
C	Cys	TGT	---	0.54	-	0.60	-
C	Cys	TGC	(GCA)	0.61	2	0.61	8
*	Opa	TGA	---		-		-
W	Trp	TGG	(CCA)	1.19	3+	1.20	13+
R	Arg	CGT	(ACG)	0.86	4+	0.60	1
R	Arg	CGC	---	0.84	-	0.44	-
R	Arg	CGA	(TCG)	1.74	4+	2.19	25
R	Arg	CGG	(CCG)	0.69	1	0.77	-
S	Ser	AGT	---	0.97	-	0.68	-
S	Ser	AGC	(GCT)	1.06	2+	0.99	6+
R	Arg	AGA	(TCT)	0.91	2	0.83	4
R	Arg	AGG	(CCT)	0.54	1	0.24	1
G	Gly	GGT	---	1.73	-	1.68	-
G	Gly	GGC	(GCC)	1.52	6	2.18	30
G	Gly	GGA	(TCC)	2.47	5	2.09	11+
G	Gly	GGG	(CCC)	0.78	1	0.43	-

	ARAD	YALI
Number of tDNA	147	510
Nr of anticodons	46	44

The first column (AA) indicates the charged amino acid (in one- and three-letter codes), the second one (C) the codon and the third one (AC) the anticodon (between brackets). For each genome, the first value is the codon usage (in % of the 61 sense codons) and the second one the number of tRNA genes ("-" no tRNA gene). The "+" signs denote genes with intron. Data for *Yarrowia lipolytica* are taken from (Dujon et al., 2004).

Contrary to other hemiascomycetes belonging to the *Saccharomyces*, protoploids and CTG clades (Dujon, 2010), *A. adeninivorans* follows the regular eukaryal-type sparing rules to read CTY Leu and CGA Arg codons. *A. adeninivorans* displays the complete eukaryotic set of 46 tDNAs (Marck et al., 2006).

Three sequence exceptions are found: 1- in the seven copies of tDNA-Ala (AGC), 5 GU (or mismatched) pairs are found (usually no more than 4 (Marck and Grosjean, 2002)); 2- 3 GU pairs in a row occur in the two copies of tDNA-Cys (GCA); 3- A_{18} instead of the conserved G_{18} is present in the two copies of tDNA-Pro (TGG) (as in *Y. lipolytica* (Marck et al., 2006)).

The genome of *A. adeninivorans* comprises only one type of tandem tDNA, **tDNA-Ile (AAT)-tDNA-His (GTG)**, present in two copies. In both cases the distance separating the two genes (number of nucleotides between base 73 of the first tRNA gene and base 1 of the second gene) is one nucleotide.

///

Table S3B Genes encoding snRNAs and snoRNAs

Locus_tag	Coordinates	Str.	Class	Gene	Annotation
**Pol III transcripts**					
ARAD1D16500r	1348010..1348345	c	RNase_MRP_RNA	RPM1	RNA component of mitochondrial RNase P
ARAD1C16698r	1375869..1376141	c	RNaseP_RNA	RPR1	RNA component of nuclear RNase P
ARAD1D43428r	3605025..3605287		SRP_RNA	SCR1	RNA subunit of the Signal Recognition Particle (SRP)
ARAD1C04598r	366612..366694		snoRNA	SNR52-B	C/D box small nucleolar RNA, snoU83
ARAD1B19976r	1648499..1648609	c	snRNA	SNR6	U6 RNA, U6 spliceosomal RNA
**Pol II transcripts**					
ARAD1D11726r	946178..946328	c	snRNA	SNR19	U1 RNA, U1 spliceosomal RNA
ARAD1B05148r	426576..426766	c	snRNA	SNR20	U2 RNA, U2 spliceosomal RNA
ARAD1C31152r	2599336..2599489	c	snRNA	SNR14	U4 RNA, U4 spliceosomal RNA
ARAD1C00506r	33699..33819	c	snRNA	SNR7	U5 RNA, U5 spliceosomal RNA
ARAD1A07095r	582975..583075		snoRNA	SNR3	H/ACA box small nucleolar RNA snR3
ARAD1C38522r	3204443..3204601		snoRNA	SNR5	H/ACA box small nucleolar RNA
ARAD1A00858r	60266..60413	c	snoRNA	SNR8	H/ACA box small nucleolar RNA
ARAD1B05082r	421701..421905		snoRNA	SNR10	H/ACA box small nucleolar RNA
ARAD1A17358r	1453882..1453978	c	snoRNA	SNR13	C/D box small nucleolar RNA
ARAD1C25300r	2121301..2121664		snoRNA	SNR17	C/D box small nucleolar RNA, U3a/b RNA
ARAD1D28974r	2399197..2399296		snoRNA	SNR18	C/D box small nucleolar RNA, U18 RNA
ARAD1C26488r	2210588..2210668	c	snoRNA	SNR24	C/D box small nucleolar RNA, U24 RNA
ARAD1B04961r	412539..412651		snoRNA	SNR30	H/ACA box small nucleolar RNA snR30
ARAD1D11792r	951021..951172	c	snoRNA	SNR36	H/ACA box small nucleolar RNA
ARAD1B15411r	1267213..1267452		snoRNA	SNR37	H/ACA box small nucleolar RNA snR37
ARAD1D45298r	3769949..3770032	c	snoRNA	SNR38	C/D box small nucleolar RNA
ARAD1C40777r	3402539..3402587	c	snoRNA	snR40	C/D box small nucleolar RNA snR40
ARAD1D19382r	1589941..1590025		snoRNA	SNR41	C/D box small nucleolar RNA
ARAD1C04147r	338534..338592		snoRNA	snR42	H/ACA box small nucleolar RNA snR42
ARAD1C34232r	2848612..2848791	c	snoRNA	SNR43	H/ACA box small nucleolar RNA
ARAD1C02321r	191313..191457	c	snoRNA	SNR44	H/ACA box small nucleolar RNA snR44
ARAD1D38907r	3196551..3196717	c	snoRNA	SNR45	C/D box small nucleolar RNA snR45
ARAD1B16258r	1328173..1328231		snoRNA	SNR47	C/D box small nucleolar RN
ARAD1D45254r	3768559..3768648	c	snoRNA	SNR48	C/D box small nucleolar RNA
ARAD1D28270r	2339943..2340077		snoRNA	SNR49	H/ACA box small nucleolar RNA
ARAD1D19426r	1590418..1590507		snoRNA	SNR51	C/D box small nucleolar RNA
ARAD1C31790r	2649559..2649627	c	snoRNA	SNR53	C/D box small nucleolar RNA
ARAD1B08470r	694966..695048		snoRNA	SNR54	C/D box small nucleolar RNA
ARAD1C36322r	2993595..2993668	c	snoRNA	SNR55	C/D box small nucleolar RNA
ARAD1D26774r	2217444..2217513	c	snoRNA	SNR56	C/D box small nucleolar RNA
ARAD1C36344r	2993695..2993778	c	snoRNA	SNR57	C/D box small nucleolar RNA
ARAD1D33726r	2790591..2790685	c	snoRNA	SNR60	C/D box small nucleolar RNA
ARAD1C36300r	2993448..2993532	c	snoRNA	SNR61	C/D box small nucleolar RNA
ARAD1D16478r	1347839..1347910	c	snoRNA	SNR66	C/D box small nucleolar RNA
ARAD1C31812r	2649696..2649775	c	snoRNA	SNR67	C/D box small nucleolar RNA
ARAD1D31922r	2634141..2634227	c	snoRNA	SNR69	C/D box small nucleolar RNA
ARAD1D19404r	1590066..1590134		snoRNA	SNR70	C/D box small nucleolar RNA
ARAD1A16984r	1428737..1428818		snoRNA	SNR71	C/D box small nucleolar RNA
ARAD1A04884r	398241..398337		snoRNA	SNR73	C/D box small nucleolar RNA
ARAD1A04862r	398151..398224		snoRNA	SNR74	C/D box small nucleolar RNA
ARAD1A04840r	397984..398070		snoRNA	SNR75	C/D box small nucleolar RNA
ARAD1A04818r	397800..397887		snoRNA	SNR76	C/D box small nucleolar RNA
ARAD1A04811r	397642..397730		snoRNA	snR77	C/D box small nucleolar RNA snR77
ARAD1A04796r	397500..397574		snoRNA	SNR78	C/D box small nucleolar RNA
ARAD1C19844r	1635627..1635649		snoRNA	SNR79	C/D box small nucleolar RNA
ARAD1C04510r	363203..363336		snoRNA	SNR80	H/ACA box small nucleolar RNA
ARAD1B02959r	243466..243577		snoRNA	snR87	C/D box small nucleolar RNA snR87
ARAD1A03300r	267696..267803	c	snoRNA	SNR128	C/D box small nucleolar RNA
ARAD1D38951r	3199765..3199908	c	snoRNA	snR161	H/ACA box small nucleolar RNA snR161
ARAD1C08866r	711755..711935	c	snoRNA	SNR191	H/ACA box small nucleolar RNA
