# Supplementary material for: The complete genome of Blastobotrys (Arxula) adeninivorans LS3 - a yeast of biotechnological interest
Source: Biotechnol Biofuels. 2014 Apr 24;7:66. doi: 10.1186/1754-6834-7-66 (PMC4022394; doi:10.1186/1754-6834-7-66)
Supplement: Additional file 4 — Centromeres. [file 1754-6834-7-66-S4.pdf]

Additional File 4. Centromeres

In *A. adeninivorans*, a conspicuous GC bias is observed once per chromosome defining ~6 kb GC-troughs, with a GC-content of 31-33% vs. 48% for the whole genome (Fig. S4A). These troughs contain large stretches of polyA (~50 residues or more), are gene-free but not associated to inverted repeats or transposons. They resemble those found in *Y. lipolytica* CEN regions (Fig. S4B), even so gene-neighborhood around these regions is not conserved between the two species. A search on the 6 kb putative CEN regions identified a degenerate motif (N**TTCC**N**ATT**NN**GG**NA) present once or twice in each putative CEN region and distantly related to the *Y. lipolytica* CEN consensus (T**TTCC**NA**ATT**N**GG**A**A**A), see Fig. S4C. Although conserved motifs were thus predicted in these putative CENs, contrary to regional CENs, no match was found in the *A. adeninivorans* genome for the 11 proteins that were defined as specific of species having point centromeres (Table S4). Matches were however predicted for two genes, which are absent from the genomes of yeasts species having point centromeres: *SIM4* of *S. pombe* (ARAD1A14256g) and *FTA3* of *A. nidulans* (ARAD1D46310g). Taken together, these results confirm that regional centromeres are ancestral in hemiascomycetes and suggest in addition that they evolved following different paths in pre-CTG, CTG and post-CTG clades.

Figure S4A Putative CEN regions in *A. adeninivorans*.

The mean GC content (left axis) along each chromosome (window size: 2,000 nt) is depicted: conspicuous GC troughs are proposed as centromeric regions.

Figure S4B Centromeric regions are gene-free in *Y. lipolytica* and *A. adeninivorans*.

Chromosomal regions predicted (*A. adeninivorans*, left panels) or known (*Y. lipolytica*, right panels) are compared for CDS distribution (blue arrows), presence of repeats (dark blue arrows) and variation in GC content (wawy line on top of each panel, GC content with a 2.5 SD cutoff; Window size: 2000 nt). The position of the *Y. lipolytica* CEN sequences was defined by a functional test [7,8] and is indicated by green arrows as well as the position of the 16 bp consensus.

Figure S4C Conserved motifs in the centromeric regions in *Y. lipolytica* and *A. adeninivorans.*

The *Y. lipolytica* consensus (right) was derived from the 10 bp consensus defined by [9] on 5 chromosomes and extended to the 6 chromosomes using MEME[10]. The *A. adeninivorans* CEN consensus was identified through MAST searches[11] on the four chromosomes using the MEME derived PSSM from the *Y. lipolytica* consensus. All sequences occur only once in each species.

Figure S4A

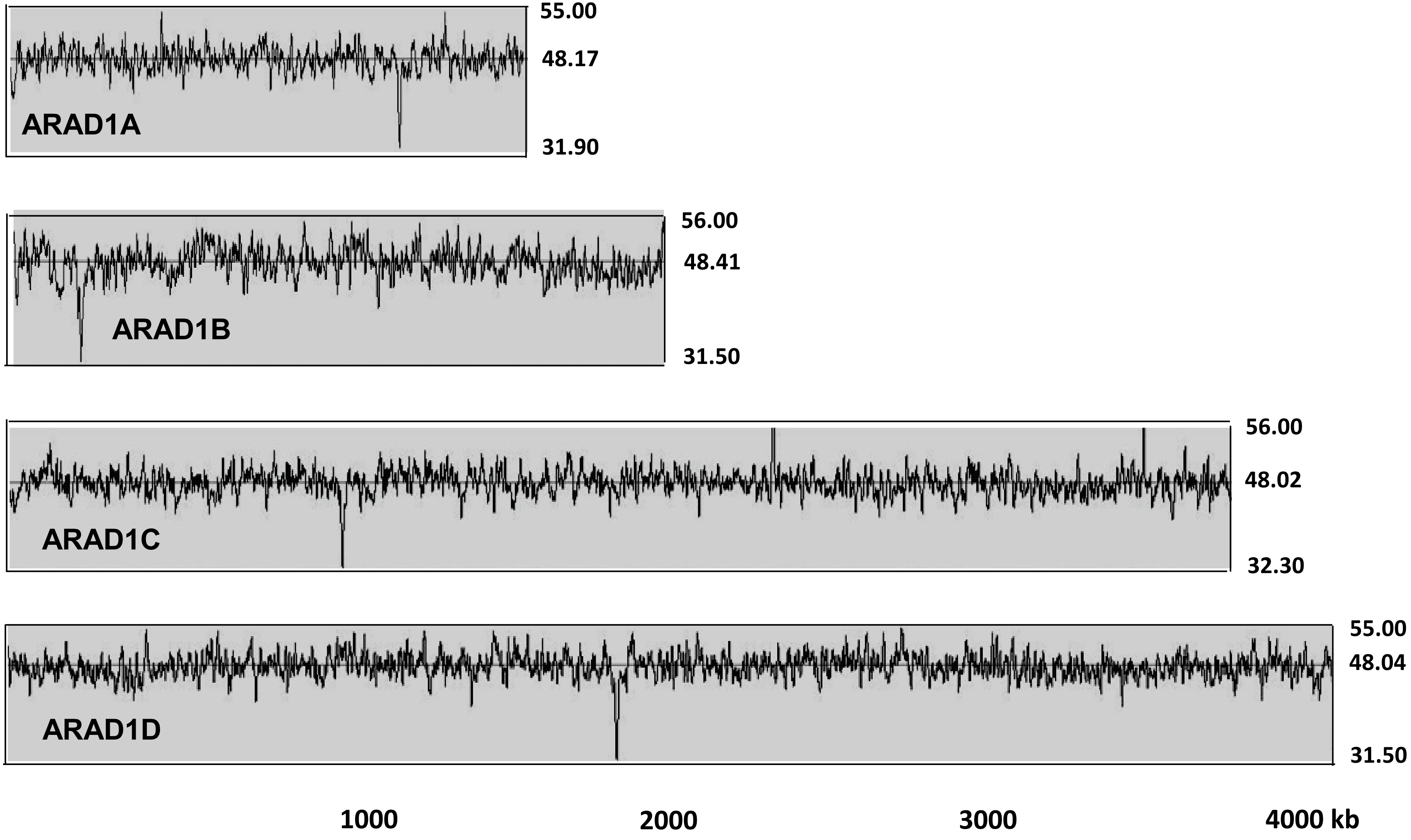
55.00
48.17
ARAD1A
31.90
56.00
48.41
ARAD1B
31.50
56.00
48.02
ARAD1C
32.30
55.00
48.04
ARAD1D
31.50
1000
2000
3000
4000 kb

Figure S4B

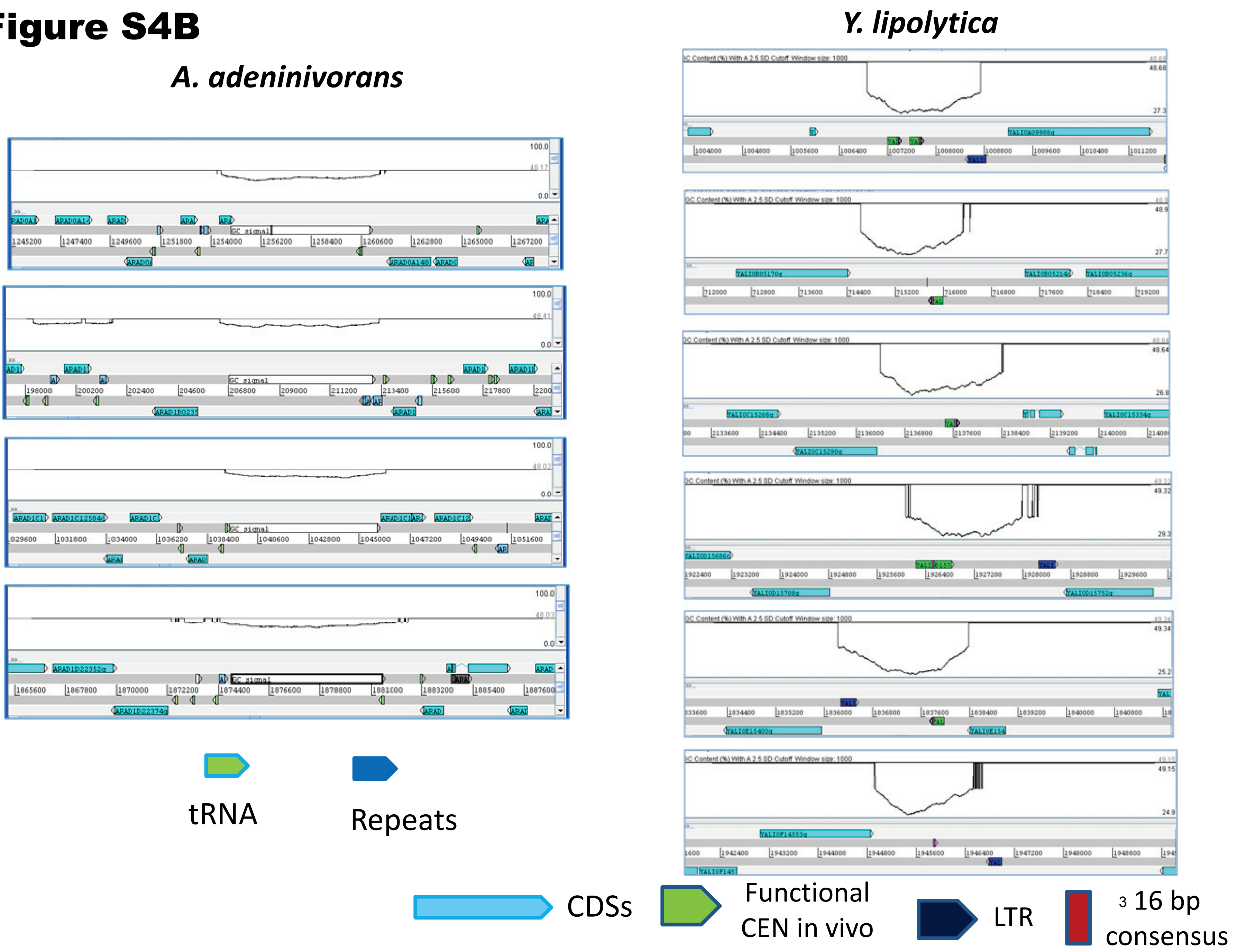
Y. lipolytica
A. adeninivorans
tRNA
Repeats
CDSs
Functional CEN in vivo
LTR
3 16 bp consensus

Figure S4C

A. adeninivorans

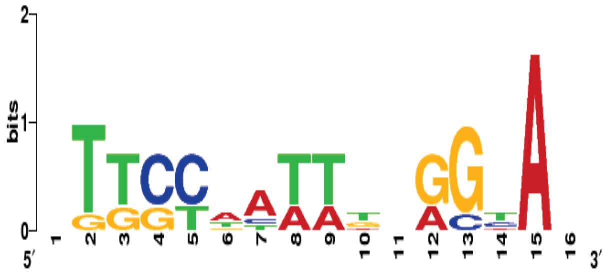

Arad1A	TTTGCAATTAAGGTAT	1255073
	ATTCCTCTTTGGGCAG	1256851
Arad1B	CTTGCTTATTGAGTAA	207561
	CTGCTAATAGTGCAAA	207962
Arad1C	CTGCTAAATTAAGTAG	1041801
Arad1D	GGTCCGATAGTGGCAC	1925290

Y. lipolytica

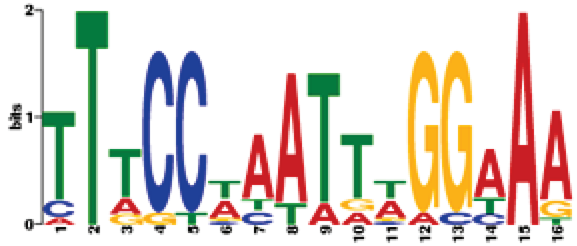

CEN1-1	CTTCCTAATTTGGAAA	1007727
CEN1-2	TTACCAAATTAGGTAA	1077356
CEN3-1	TTTCCTAAATAGGAAG	2137323
CEN6	TTTCCTAAATTGGTAA	1945877
CEN5-1	TTGCCCTATTCGGAAA	1837793
CEN2-2	ATGCCAAATATGGAAA	715804
CEN4-1	TTTGTTCATGTGGAAA	1926501
