# Supplementary material for: The complete genome of Blastobotrys (Arxula) adeninivorans LS3 - a yeast of biotechnological interest
Source: Biotechnol Biofuels. 2014 Apr 24;7:66. doi: 10.1186/1754-6834-7-66 (PMC4022394; doi:10.1186/1754-6834-7-66)

Additional File 5. Functional annotation

Figure S5A Functional classification of *A. adeninivorans* ORFs by biological process category.

Assessments were made through Blat2GO.

Figure S5B Best BLAST hits of *A. adeninivorans* CDS in other genomes.

BLASTx alignments of predicted proteins against the NCBI NRPEP database confirm the close relationship of *A. adeninivorans* and *Y. lipolytica*.

Figure S5A Process categories

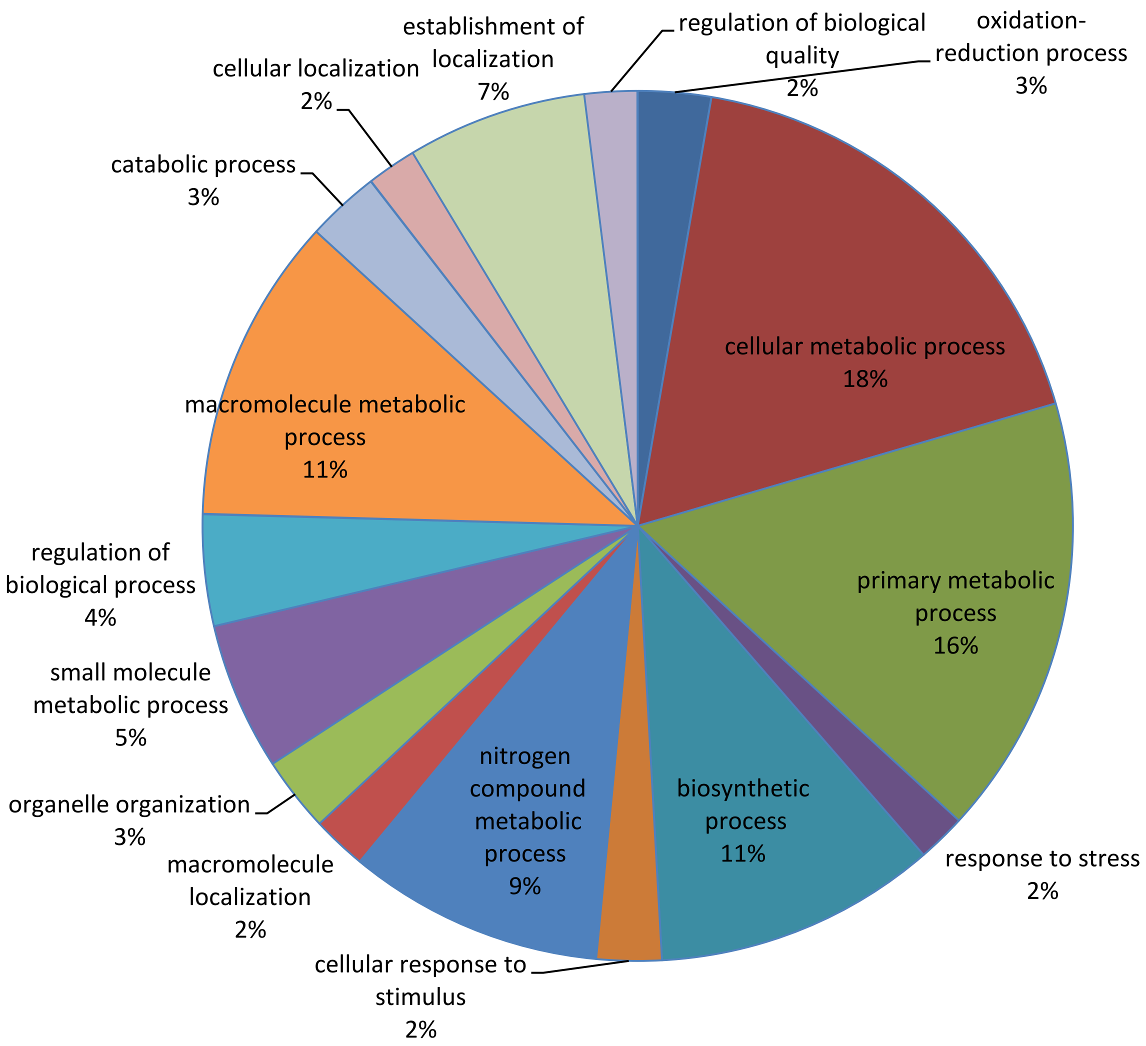

Figure S5B Best BLASTx hits of *A. adeninivorans* CDS in other genomes

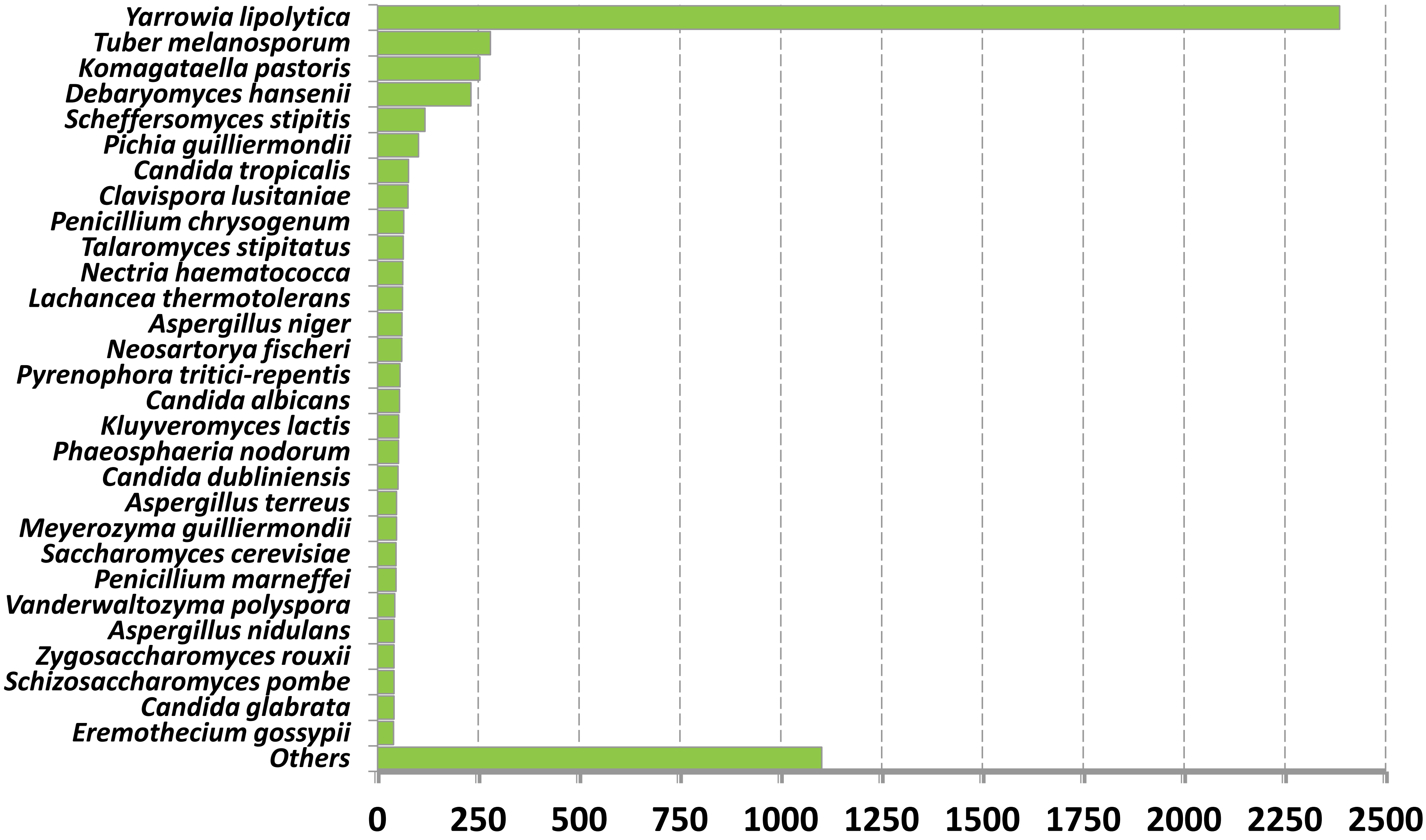

Supplement: Additional file 5 — Functional annotation. [file 1754-6834-7-66-S5.pdf]
