# Supplementary material for: The complete genome of Blastobotrys (Arxula) adeninivorans LS3 - a yeast of biotechnological interest
Source: Biotechnol Biofuels. 2014 Apr 24;7:66. doi: 10.1186/1754-6834-7-66 (PMC4022394; doi:10.1186/1754-6834-7-66)
Supplement: Additional file 6 — Spliceosomal introns. [file 1754-6834-7-66-S6.pdf]

Additional File 6. Spliceosomal introns

Table S6A Number of introns per gene.

Number of genes containing 1 to 7 introns are listed per chromosome. The comparison with *Y. lipolytica* (YALI) is shown on the right.

Figure S6B Size distribution.

Intron sizes are indicated on the x-axis (20bp intervals). The number of introns per size class is on the y-axis. Data for *A. adeninivorans* (ARAD) are in green, and those for *Y. lipolytica* (YALI) in red. Introns are smaller in ARAD than in YALI, with 82% introns shorter than 80 nucleotides.

Table S6A Number of introns per gene

	number of introns per gene					
	1	2	3	4	5	7
Arad1A	68	11	4	1	0	0
Arad1B	84	24	1	0	0	0
Arad1C	200	44	11	3	2	0
Arad1D	200	31	11	7	0	1
**Total**	**552**	**110**	**27**	**11**	**2**	**1**

Intron-containing genes
703/6116=11.5% in ARAD
984/6449=15.3% in YALI

Multi-intronic genes
21.5% in ARAD (151/703)
11.5% in YALI (109/951)

Figure S6B Size distribution

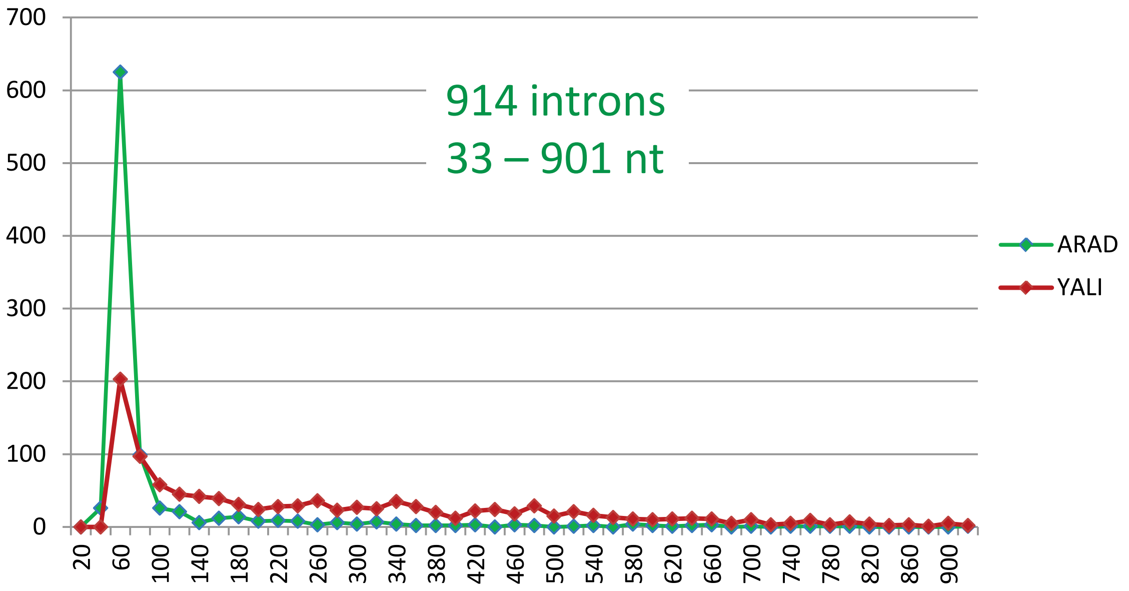

Introns per I-gene
1.30 in ARAD
1.14 in YALI

82% of introns ≤ 80 nt
27% in YALI
