# Supplementary material for: The complete genome of Blastobotrys (Arxula) adeninivorans LS3 - a yeast of biotechnological interest
Source: Biotechnol Biofuels. 2014 Apr 24;7:66. doi: 10.1186/1754-6834-7-66 (PMC4022394; doi:10.1186/1754-6834-7-66)

Additional File 7. Phylogeny and synteny of *A. adeninivorans* and other yeasts

Figure S7A Species trees.

Species trees were computed from a set of concatenated orthologs and by a super tree approach combining all individual gene phylogenies (see online Methods). Right panel: concatenated orthologs. Left panel: super-tree.

Figure S7B Circos map of the whole nuclear genome of *A. adeninivorans* strain LS3 nuclear genome and syntenic blocks with *Y. lipolytica, K. pastoris* and *S. cerevisiae*.

Concentric circles illustrate aspects of the genome. Chromosome structure (the outermost circle - circle 1): presumed centromeric positions were indicated by black bands as well as black triangles outside the circle and tRNA and rRNA genes as green bands and orange bands, respectively. Genes (circle 2): density of genes in the filtered gene set across the genome, from a gene count per 15-kb sliding window at 5-kb intervals. Repeat content (circle 3): for creating k-mer density ring, k-mers with length = 20 in whole genome using jellyfish program v. 1.1.1 (http://www.cbcb.umd.edu) were counted, a position map of k-mer count was created, k-mers counts in blocks of 3kb were divided by 3,000 and the data was plotted using circos's heatmap. 454 reads mapped to chromosomes (circle 4): Density of 454 reads mapped to chromosomes, from a 454 read count per 9-kb sliding window at 3-kb intervals. *Y. lipolytica* synteny (circle 5), *K. pastoris* synteny (circle 6) and *S. cerevisiae* synteny (circle 7). Syntenic blocks between *A. adeninivorans* and related yeast on the basis of tblastx search (e-value = 1e-10) between *A. adeninivorans* chromosomes and CDSs of related yeast. Underlined blocks indicate alignment in the reverse strand. The list of colors used for the chromosomes of *Y. lipolytica*, *K. pastoris* and *S. cerevisiae* for circles 5-7 is given below the circos map.

Figure S7C Synteny between *A. adeninivorans* and *S. cerevisiae, K. lactis, D. hansenii, K. pastoris* and *Y. lipolytica*

A search of bidirectional best Blastp hits (BDBH) was conducted between *A. adeninivorans* proteome and those of above listed species. Gene blocks were defined as continuous series of genes in genome pairs, allowing for one insertion/deletion between two homologs. The number of conserved blocks between *A. adeninivorans* and other genomes ranged from 300 with *S. cerevisiae* to 800 with *Y. lipolytica* and was roughly proportional to the mean percentage of protein similarity, with *D. hansenii* and *K. pastoris* showing intermediate values (roughly 350 and 500 blocks, respectively).

Top: Dot plots between homologues defined by BDBH between the proteins in the different species.

Bottom: Mean protein similarity vs. Number of syntenic blocks.

Suppl. data S7D Synteny between *A. adeninivorans* and *Y. lipolytica.*

Table S7D Syntenic blocks of genes with up to one insertion/deletion.

Blocks were defined by Blastp searches with a cutoff at e-10-10 between all CDS of *A. adeninivorans* and *Y. lipolytica* including pseudogenes. More distant homologs between *Y. lipolytica* and *A. adeninivorans* were screened by Blastp searches with a threshold of 10E-05 and manually curated in order to extend the blocks. A total of 1316 blocks of 2 to 15 genes were then found conserved between *Y. lipolytica* and *A. adeninivorans* accounting for over 58% of the gene content of this species (Supplementary S7B)

Figure S7D Examples of syntenic blocks of genes with two insertions/deletions.

Figure S7A species trees

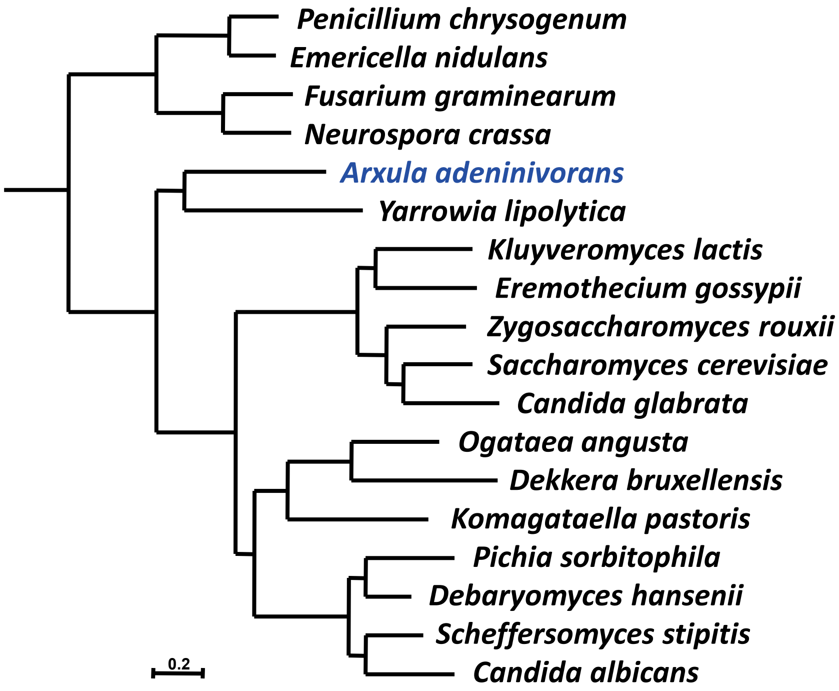

Concatenated species tree

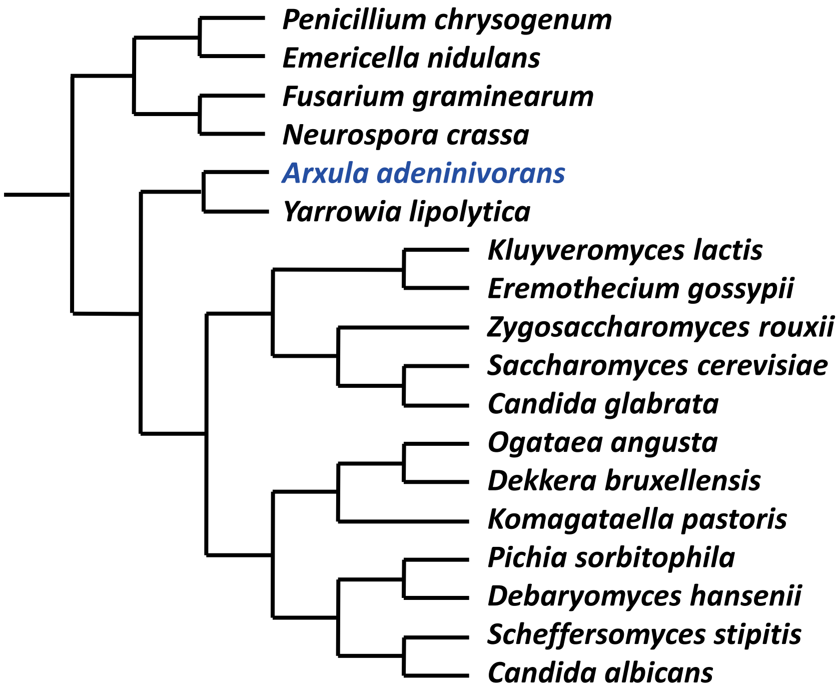

Duptree species tree

Figure S7B   Circos map of *A. adeninivorans* genome showing syntenic blocks with *Y. lipolytica*, *K. pastoris* and *S. cerevisiae*

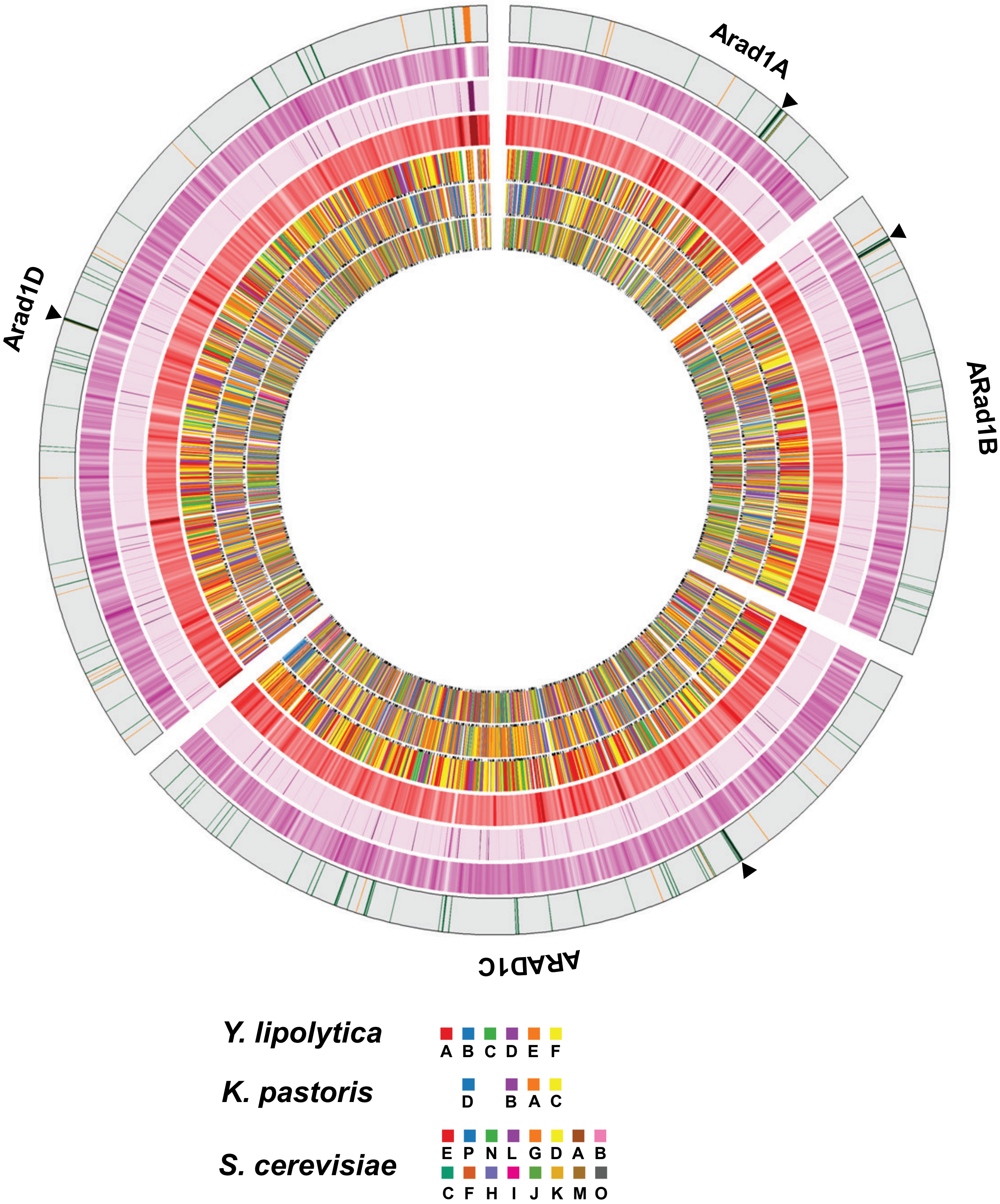

Figure S7C Synteny between *A. adeninivorans, S. cerevisiae, K. pastoris, D. hansenii* and *Y. lipolytica*

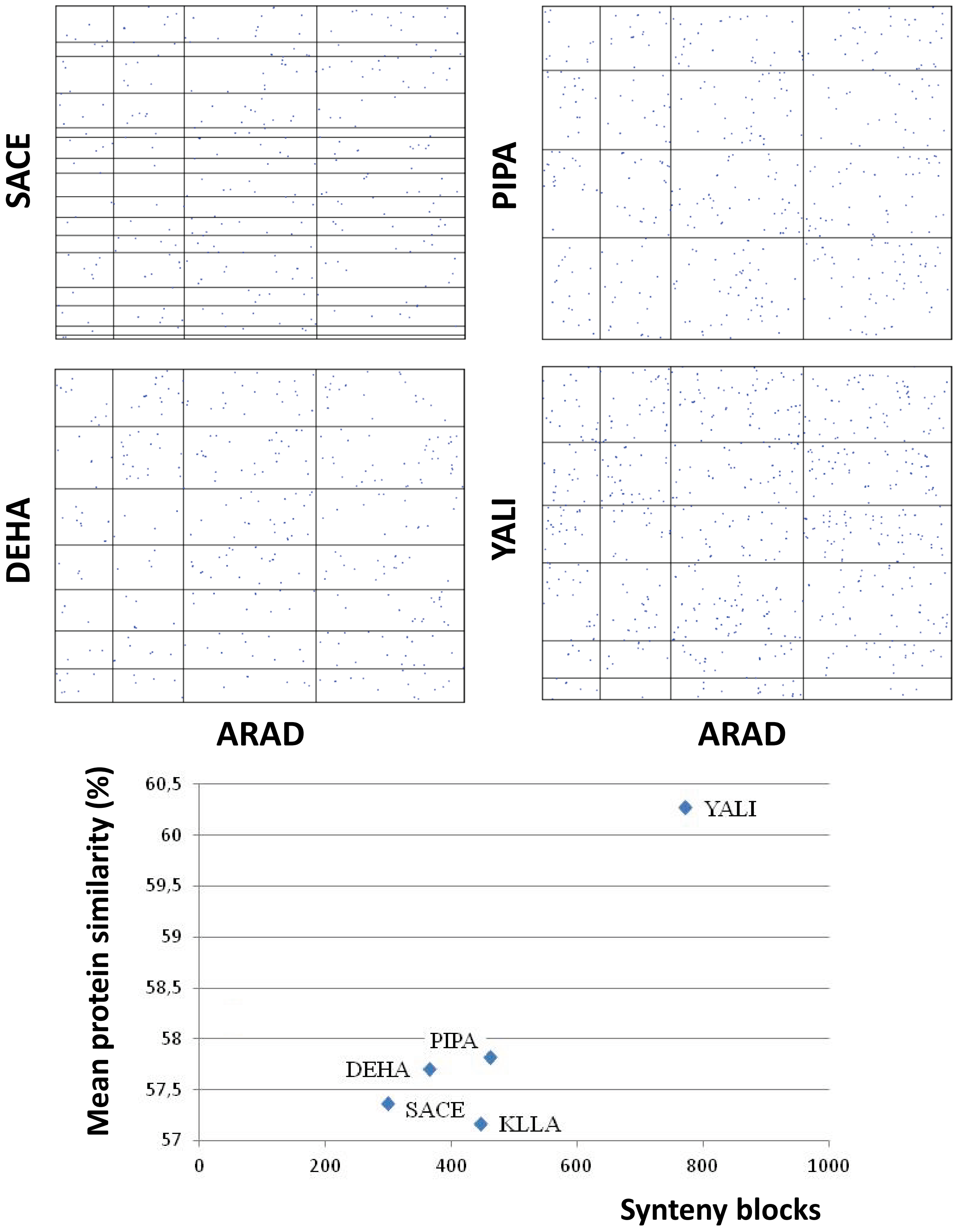

Suppl. data S7D Syntenic blocks with *Y. lipolytica*

Table S7D Syntenic blocks of genes with up to one insertion/deletion

(Size of block refers to number of genes in this block)

Block size	Block nb.	Genes in blocks
2	900	1800
3	342	1026
4	158	632
5	86	430
6	36	216
7	20	140
8	9	72
9	5	45
10	2	20
11	1	11
12	1	12
13	1	13
**Total nb of genes in blocks**		**4417**

Figure S7D Syntenic blocks of genes with two insertions/deletions

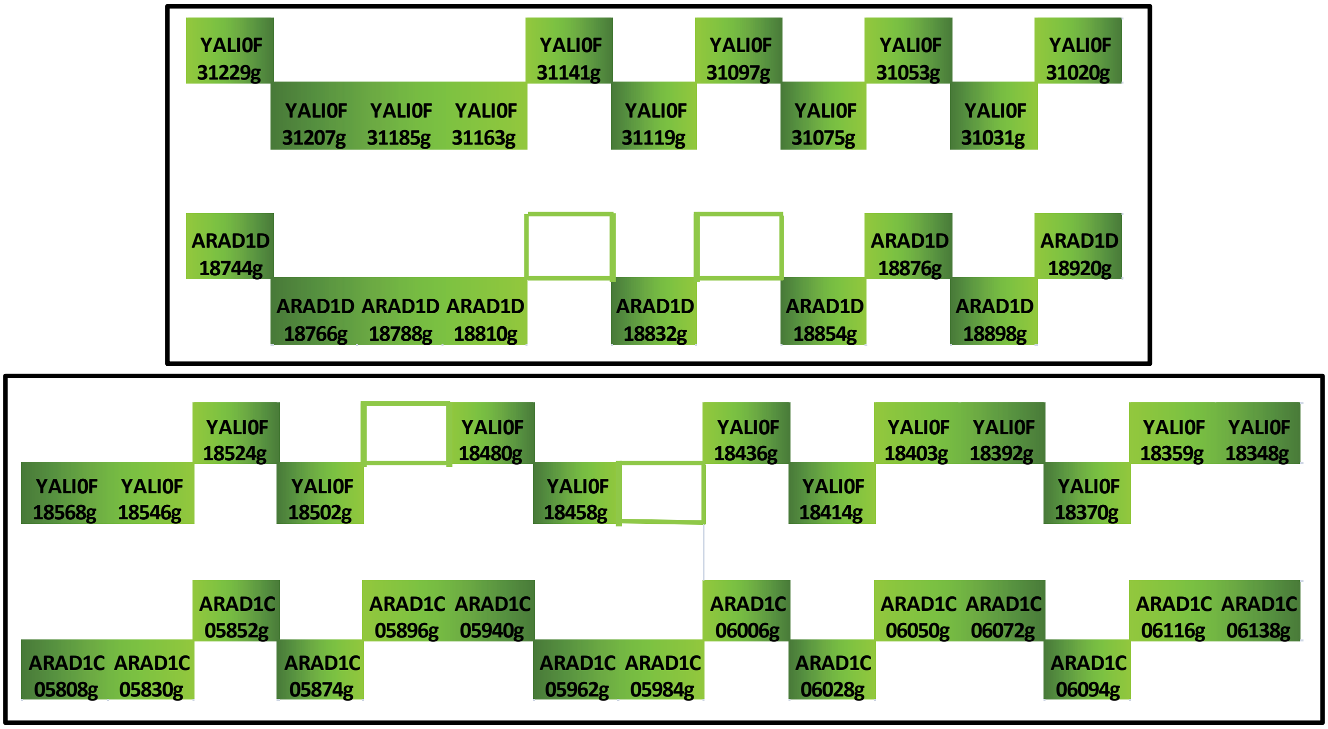

Supplement: Additional file 7 — Phylogeny and synteny between A. adeninivorans and other yeasts. [file 1754-6834-7-66-S7.pdf]
