# Supplementary material for: The complete genome of Blastobotrys (Arxula) adeninivorans LS3 - a yeast of biotechnological interest
Source: Biotechnol Biofuels. 2014 Apr 24;7:66. doi: 10.1186/1754-6834-7-66 (PMC4022394; doi:10.1186/1754-6834-7-66)
Supplement: Additional file 8 — Proteins having homologs only in Pezizomycotina or bacteria. [file 1754-6834-7-66-S8.pdf]

Additional File 8. List of proteins having homologs only in Pezizomycotina or bacteria

Table S8A List of proteins that only have pezizomycotina species as homologs.

Abbreviations used are: EMENI= *Emericella nidulans*, GIBZE= *Giberella zeae*, PENCH= *Penicillium chrysogenum*, NEUCR= *Neurospora crassa*.

Table S8B Putative horizontally transferred genes.

Table S8A List of proteins that only have pezizomycotina species as homologs

PhylomeDB ID	ARAD ID	Homologs in species
Phy004C1PR_BLAAD	ARAD1A01540g	EMENI|PENCH
Phy004C1U1_BLAAD	ARAD1A05082g	GIBZE|EMENI|PENCH|NEUCR
Phy004C1UF_BLAAD	ARAD1A05412g	GIBZE|EMENI|PENCH|NEUCR
Phy004C1W7_BLAAD	ARAD1A06842g	GIBZE|NEUCR
Phy004C1ZM_BLAAD	ARAD1A09548g	GIBZE|EMENI|PENCH
Phy004C20M_BLAAD	ARAD1A10373g	GIBZE|EMENI|NEUCR
Phy004C20S_BLAAD	ARAD1A10494g	GIBZE|EMENI|PENCH|NEUCR
Phy004C21M_BLAAD	ARAD1A11154g	GIBZE|EMENI
Phy004C221_BLAAD	ARAD1A11484g	EMENI|PENCH
Phy004C245_BLAAD	ARAD1A13200g	GIBZE|EMENI|PENCH|NEUCR
Phy004C24U_BLAAD	ARAD1A13750g	EMENI
Phy004C254_BLAAD	ARAD1A13970g	GIBZE|NEUCR|PENCH
Phy004C25X_BLAAD	ARAD1A14630g	GIBZE
Phy004C26F_BLAAD	ARAD1A15312g	EMENI|PENCH
Phy004C28D_BLAAD	ARAD1A16896g	GIBZE|EMENI|PENCH|NEUCR
Phy004C28Y_BLAAD	ARAD1A17402g	GIBZE|EMENI|PENCH|NEUCR
Phy004C28Z_BLAAD	ARAD1A17424g	GIBZE|EMENI|PENCH|NEUCR
Phy004C2A7_BLAAD	ARAD1A18392g	GIBZE|PENCH
Phy004C2C7_BLAAD	ARAD1B00110g	EMENI
Phy004C2J4_BLAAD	ARAD1B06182g	GIBZE|EMENI|PENCH|NEUCR
Phy004C2JY_BLAAD	ARAD1B06842g	GIBZE|EMENI|NEUCR|PENCH
Phy004C2JZ_BLAAD	ARAD1B06864g	GIBZE|EMENI|PENCH|NEUCR
Phy004C2NE_BLAAD	ARAD1B09636g	GIBZE|EMENI|PENCH|NEUCR
Phy004C2O0_BLAAD	ARAD1B10120g	PENCH
Phy004C2OG_BLAAD	ARAD1B10494g	GIBZE|EMENI
Phy004C2RQ_BLAAD	ARAD1B13244g	EMENI|PENCH
Phy004C2SN_BLAAD	ARAD1B14102g	GIBZE|EMENI|NEUCR
Phy004C2T0_BLAAD	ARAD1B14388g	GIBZE|EMENI|PENCH|NEUCR
Phy004C2W1_BLAAD	ARAD1B16852g	GIBZE|PENCH
Phy004C2WP_BLAAD	ARAD1B17402g	EMENI
Phy004C2X5_BLAAD	ARAD1B17754g	GIBZE|EMENI|PENCH|NEUCR
Phy004C2XK_BLAAD	ARAD1B18084g	GIBZE|EMENI|PENCH|NEUCR
Phy004C2XS_BLAAD	ARAD1B18260g	EMENI
Phy004C318_BLAAD	ARAD1B21054g	EMENI
Phy004C34D_BLAAD	ARAD1B23672g	GIBZE|EMENI|NEUCR|PENCH
Phy004C351_BLAAD	ARAD1B24178g	GIBZE|EMENI|PENCH|NEUCR
Phy004C35E_BLAAD	ARAD1B24442g	EMENI
Phy004C35N_BLAAD	ARAD1C00286g	GIBZE|EMENI|PENCH
Phy004C35P_BLAAD	ARAD1C00330g	EMENI|PENCH|NEUCR
Phy004C35V_BLAAD	ARAD1C00462g	GIBZE|EMENI|PENCH|NEUCR
Phy004C36R_BLAAD	ARAD1C01232g	GIBZE|EMENI|NEUCR|PENCH
Phy004C36T_BLAAD	ARAD1C01276g	EMENI|PENCH
Phy004C37H_BLAAD	ARAD1C01804g	GIBZE|PENCH
Phy004C38B_BLAAD	ARAD1C02486g	EMENI|PENCH
Phy004C394_BLAAD	ARAD1C03124g	GIBZE|NEUCR|PENCH
Phy004C39L_BLAAD	ARAD1C03498g	GIBZE|EMENI|PENCH|NEUCR
Phy004C39M_BLAAD	ARAD1C03520g	EMENI|PENCH
Phy004C3AQ_BLAAD	ARAD1C04422g	GIBZE|EMENI|NEUCR|PENCH
Phy004C3BX_BLAAD	ARAD1C05434g	PENCH
Phy004C3EQ_BLAAD	ARAD1C07722g	PENCH
Phy004C3HQ_BLAAD	ARAD1C10164g	GIBZE|EMENI
Phy004C3I2_BLAAD	ARAD1C10406g	PENCH|NEUCR
Phy004C3IK_BLAAD	ARAD1C10802g	GIBZE|EMENI|PENCH|NEUCR
Phy004C3KO_BLAAD	ARAD1C12496g	EMENI|PENCH
Phy004C3KX_BLAAD	ARAD1C12782g	EMENI|PENCH
Phy004C3LD_BLAAD	ARAD1C13266g	GIBZE|EMENI|PENCH
Phy004C3M9_BLAAD	ARAD1C13948g	GIBZE|EMENI|PENCH|NEUCR
Phy004C3NC_BLAAD	ARAD1C14894g	GIBZE|EMENI|PENCH
Phy004C3OH_BLAAD	ARAD1C15818g	GIBZE|PENCH
Phy004C3OZ_BLAAD	ARAD1C16214g	EMENI|PENCH
Phy004C3R0_BLAAD	ARAD1C17996g	GIBZE|EMENI|PENCH|NEUCR
Phy004C3RG_BLAAD	ARAD1C18348g	GIBZE|PENCH
Phy004C3SV_BLAAD	ARAD1C19492g	GIBZE

Phy004C3UM_BLAAD	ARAD1C20900g	GIBZE|EMENI|NEUCR
Phy004C3W3_BLAAD	ARAD1C22088g	GIBZE|NEUCR
Phy004C3W7_BLAAD	ARAD1C22176g	GIBZE|EMENI|PENCH|NEUCR
Phy004C3XD_BLAAD	ARAD1C23166g	GIBZE|EMENI|NEUCR|PENCH
Phy004C40X_BLAAD	ARAD1C26092g	GIBZE|EMENI|PENCH|NEUCR
Phy004C48R_BLAAD	ARAD1C32560g	GIBZE|EMENI|PENCH
Phy004C4BB_BLAAD	ARAD1C34848g	EMENI
Phy004C4DE_BLAAD	ARAD1C36608g	GIBZE|PENCH
Phy004C4GG_BLAAD	ARAD1C39050g	EMENI|NEUCR
Phy004C4GO_BLAAD	ARAD1C39226g	GIBZE|EMENI|PENCH|NEUCR
Phy004C4HV_BLAAD	ARAD1C40172g	EMENI|PENCH
Phy004C4HZ_BLAAD	ARAD1C40260g	GIBZE|EMENI|PENCH|NEUCR
Phy004C4IU_BLAAD	ARAD1C40964g	EMENI|PENCH|NEUCR
Phy004C4JI_BLAAD	ARAD1C41514g	GIBZE|PENCH|NEUCR
Phy004C4JO_BLAAD	ARAD1C41668g	GIBZE|EMENI|PENCH|NEUCR
Phy004C4KM_BLAAD	ARAD1C42438g	EMENI|NEUCR|PENCH
Phy004C4OL_BLAAD	ARAD1C45694g	GIBZE|PENCH
Phy004C4OM_BLAAD	ARAD1C45716g	EMENI|NEUCR
Phy004C4OQ_BLAAD	ARAD1D00132g	EMENI
Phy004C4P4_BLAAD	ARAD1D00462g	GIBZE|EMENI|NEUCR|PENCH
Phy004C4QF_BLAAD	ARAD1D01540g	PENCH|NEUCR
Phy004C4T1_BLAAD	ARAD1D03652g	GIBZE|EMENI|NEUCR
Phy004C4V5_BLAAD	ARAD1D05500g	EMENI
Phy004C4WD_BLAAD	ARAD1D06512g	GIBZE|EMENI|PENCH|NEUCR
Phy004C505_BLAAD	ARAD1D09526g	GIBZE|EMENI|PENCH|NEUCR
Phy004C53M_BLAAD	ARAD1D12474g	GIBZE|EMENI|NEUCR|PENCH
Phy004C54X_BLAAD	ARAD1D13508g	GIBZE|EMENI|NEUCR|PENCH
Phy004C59P_BLAAD	ARAD1D17424g	GIBZE|NEUCR|PENCH
Phy004C59W_BLAAD	ARAD1D17578g	GIBZE|EMENI|NEUCR
Phy004C5AH_BLAAD	ARAD1D18040g	GIBZE|EMENI
Phy004C5AL_BLAAD	ARAD1D18128g	GIBZE|EMENI|PENCH
Phy004C5AP_BLAAD	ARAD1D18216g	GIBZE|EMENI|PENCH
Phy004C5AR_BLAAD	ARAD1D18260g	GIBZE|EMENI|PENCH|NEUCR
Phy004C5AS_BLAAD	ARAD1D18282g	GIBZE|NEUCR
Phy004C5B5_BLAAD	ARAD1D18568g	GIBZE|EMENI|PENCH
Phy004C5B9_BLAAD	ARAD1D18656g	GIBZE|EMENI|PENCH
Phy004C5CN_BLAAD	ARAD1D19866g	GIBZE|NEUCR
Phy004C5D5_BLAAD	ARAD1D20262g	GIBZE|EMENI|NEUCR|PENCH
Phy004C5E5_BLAAD	ARAD1D21076g	GIBZE|EMENI|NEUCR|PENCH
Phy004C5G9_BLAAD	ARAD1D22924g	GIBZE|PENCH
Phy004C5H8_BLAAD	ARAD1D23848g	GIBZE|EMENI|NEUCR|PENCH
Phy004C5LM_BLAAD	ARAD1D27632g	GIBZE|PENCH
Phy004C5LX_BLAAD	ARAD1D27896g	GIBZE|NEUCR|PENCH
Phy004C5O0_BLAAD	ARAD1D29678g	GIBZE|EMENI|PENCH|NEUCR
Phy004C5P2_BLAAD	ARAD1D30514g	GIBZE|EMENI|NEUCR|PENCH
Phy004C5Q9_BLAAD	ARAD1D31460g	GIBZE|EMENI|PENCH|NEUCR
Phy004C5QV_BLAAD	ARAD1D31966g	GIBZE|EMENI|PENCH|NEUCR
Phy004C5RL_BLAAD	ARAD1D32538g	EMENI|NEUCR|PENCH
Phy004C5TC_BLAAD	ARAD1D33968g	GIBZE|EMENI|NEUCR
Phy004C5TP_BLAAD	ARAD1D34254g	GIBZE|EMENI|NEUCR|PENCH
Phy004C5V0_BLAAD	ARAD1D35310g	GIBZE|NEUCR|PENCH
Phy004C5VN_BLAAD	ARAD1D35816g	GIBZE|EMENI|PENCH|NEUCR
Phy004C5YO_BLAAD	ARAD1D38214g	GIBZE|EMENI|PENCH
Phy004C622_BLAAD	ARAD1D40964g	GIBZE|EMENI|PENCH|NEUCR
Phy004C65M_BLAAD	ARAD1D44022g	GIBZE|PENCH
Phy004C69Z_BLAAD	ARAD1D47564g	PENCH|NEUCR
Phy004C6CV_BLAAD	ARAD1D50028g	EMENI|NEUCR|PENCH
Phy004C6CY_BLAAD	ARAD1D50600g	GIBZE|EMENI|PENCH|NEUCR

Table S8B Putative horizontally transferred genes

PhylomeDB ID	*Génolevures ID*	*Closest BLASTp match*	*UniProt annotation*
Phy004C1ZK_BLAAD	ARAD1A09504g	Emtol_2911 [Emticicia oligotrophica DSM 17448]	uniprot| I2EWJ1 Uncharacterized, contains a Haem oxygenase-like InterPro domain
Phy004C266_BLAAD	ARAD1A15026g	O3I_025100 [Nocardia brasiliensis ATCC 700358]	uniprot|K0F6A1 Uncharacterized, similar to Beta-lactamase class C and other penicillin binding proteins
Phy004C2A7_BLAAD	ARAD1A18392g	GCN5 [Spirosoma linguale DSM 74]	uniprot|D2QRU3 GCN5-related N-acetyltransferase
Phy004C3EQ_BLAAD	ARAD1C07722g	Pc06g02220 [Penicillium chrysogenum Wisconsin 54-1255]	uniprot|B6GWC2 Pc06g02220 protein, similar to low molecular weight phosphotyrosine protein phosphatase ArsC*
Phy004C6D8_BLAAD	ARAD1D50842g	peptidase M24 [Ajellomyces dermatitidis ATCC 18188]	uniprot|F2T984 Peptidase M24
Phy004C6DJ_BLAAD	ARAD1D51084g	PFWH6_3129 [Pseudomonas fluorescens WH6]	uniprot|E2XSY4 Uncharacterized, similar to Sugar phosphate permease **

* The same as the one described as transferred in *P. chrysogenum* and some other eurotiomycetes, but not the same found in *Yarrowia lipolytica*, which is absent in *Arxula adeninivorans*
** , Also has hits in *P. chrysogenum, Ajellomyces dermatidis* and *Aspergillus nidulans*
