# Supplementary material for: The complete genome of Blastobotrys (Arxula) adeninivorans LS3 - a yeast of biotechnological interest
Source: Biotechnol Biofuels. 2014 Apr 24;7:66. doi: 10.1186/1754-6834-7-66 (PMC4022394; doi:10.1186/1754-6834-7-66)

Additional File 9. Gene families amplified in *A. adeninivorans.*

Figure S9A Duplication rate per gene per branch of the species tree leading to *A. adeninivorans.*

Species tree showing the evolution of the species included in the phylome based on a Maximum Likelihood analysis of a concatenated set of widespread genes. Numbers at the lineages leading to *A. adeninivorans* represent duplication rates, which indicate the duplication rate per gene per branch found in the phylome. Duplication rates are calculated as number of duplications found at a given node divided by all those trees that are rooted at a deeper node.

Table S9B Gene families specifically amplified in *A. adeninivorans*.

A. adeninivorans specific expansions were obtained from the phylome, when a node contained exclusively 3 or more sequences of *A. adeninivorans*. Overlapping expansions were fused together when more than 20% of their members overlapped. Expansions were annotated using a Blastp search against UniProt.

Figure S9C Genome Redundancy based on Génolevures families.

Genes of *S. cerevisiae* (SACE), *C. glabrata* (CAGL), *Z. rouxii* (ZYRO), *S. kluyveri* (SAKL), *K. thermotolerans* (KLTH), *K. lactis* (KLLA), *E. gossypii* (ERGO), *D. hansenii* (DEHA), *Millerozyma (Pichia) sorbitophila* (PISO), *A. adeninivorans* (ARAD), and *Y. lipolytica* YALI were clustered into 'multigene families' as described in [5]. About 93% of the total proteins (62285/66964) are clustered in 5854 protein families, in which 4745 paralogous gene families (covering more than 85% of the total proteins (57203/66964) and leading to an average of 12 members in each paralogous family). The overall genome redundancy is defined as the number of families containing a set of paralogous gene copies. The degree of redundancy (percentage of proteins in multigene families) represents a more sensitive indicator of the overall genome redundancy. It ranges from 29.2-33.5% (in ERGO, KLLA, KLTH, SAKL, and ZYRO) to 97.3% (in PISO). The degree of redundancy of ARAD (37.3%) is more comparable to those observed for DEHA, YALI, SACE and CAGL (ranging from 37.3 to 40.4%). However, these results contrast with the fact that ARAD has lost a high number of paralogs compared to other hemiascomycetes. Moreover, the degree of redundancy in multigene families is lower than those seen in other eukaryotes such as *Neurospora crassa*, *S. pombe* or *Encephalitozoon cuniculi*. These findings illustrate the importance of gene loss in the ARAD/YALI branch, during the evolution.

The overall genome redundancy of each genome (in square brackets) is defined as the percentage of proteins belonging to protein families over the total number of proteins.

Table S9D Phyletic patterns of paralogous protein families.

The set of Génolevures paralogous families was analyzed according to the phyletic pattern, with a focus on the YALI/ARAD genomes. ARAD and YALI exhibit the highest sets of families missing in one species [*sczltkgdp-y* (missing genes in ARAD) and *sczltkgdpa-* (missing genes in YALI)]. We also

noticed the same observation with the pattern *sczltkgdp--* (families missing in both ARAD and YALI) with 135 gene families.

ARAD and YALI exhibit 230 (from 53 paralogous gene families) and 322 (from 93 paralogous gene families) specific proteins (pattern ------------a- and ------------y, respectively).

Gene contractions/expansions in ARAD and YALI.

The set of analysed families was restricted to strict paralogous families, *i.e.* without species-specific families and families missing in one species, leading to 3571 strict paralogous families (75.2% of the overall paralogous gene families). The set of universal families (pattern *sczltkgdpay*) is represented by 62.8% (2243/3571) of the strict paralogous families. For those families, we make the hypothesis that for each family, the number of gene along the ARAD/YALI ancestor branch was comprised between Xmin and Xmax, where Xmin is: Mean – 1.96*s/3and Xmax is Mean + 1,96*square(s2). Mean is the average of the number of genes present in the 9 others genomes. 3 is from square(9). These parameters take into account the variation of the number of paralogous gene copies in each genome, relative to the size of the family. Genomes which exhibit higher variation compared to the average will therefore be more prone to have been subjected to contraction or expansion during their evolution. Therefore, when the number of genes in ARAD or YALI is lower than Xmin, a contraction event is considered and when the number of genes is higher than Xmax, an expansion event is considered. Contraction or expansion events in both genomes is considered as an event that occurred in the ARAD/YALI ancestor branch. The results show that in main cases (NN: 64.60% = 2307/3571 of the families), no contraction and no expansion event occurred in the ARAD or YALI branches. 11.73% (CC: 419/3571) and 7.0% (EE: 250/3571) of the protein families exhibit contraction and expansion events in the ancestor branch, respectively. A lower proportion of contraction with an expansion in another genome was also observed: 0.53% (CE) in the case of a contraction event in ARAD with an expansion event in YALI, and 0.28% (EC) in the opposite case. In many cases, the expansion or contraction event appears only in one of the branches. Therefore, within the same family, a contraction event is only observed in ARAD or YALI for 2.80% (CN) or 1.54% (NC) of families, respectively; while an expansion event only occurs in 5.79% (EN) or 5.71% (NE) of the paralogous families, respectively. Overall, a contraction event appears independently 2 times (19 + 100 / 10 + 55) more in ARAD compared to YALI, whereas similar number of expansion events (10 + 207 and 19 + 204) was observed for those two genomes.

Figure S9E A 107-member gene family in *A. adeninivorans*.
Alignments were generated with Muscle 3.7[12] and treated with Gblocks[13]. Trees were built with BioNJ[14] and represented with Dendroscope 3[15]. Subfamilies obtained using the Génolevures standards for building gene families (see online Methods) are indicated.

Figure S9A Duplication rate per gene per branch of the species tree

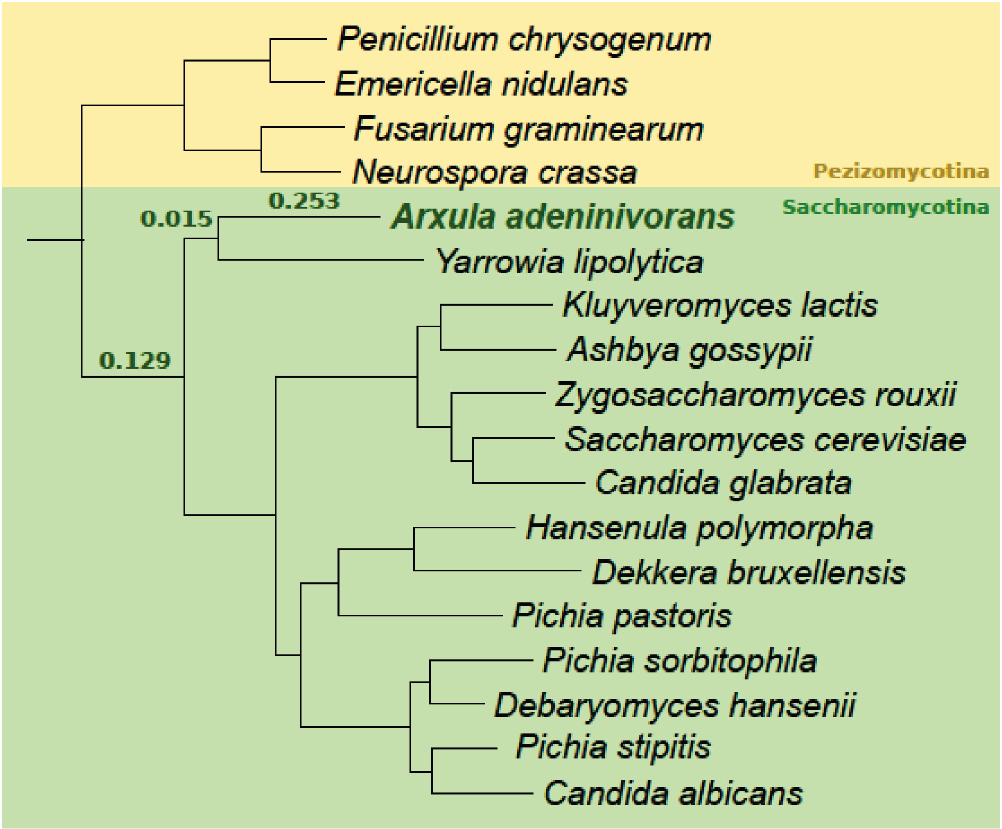

Table S9B Gene families specifically amplified in *A. adeninivorans*

Expansion_0

Phy004C1SJ_BLAAD	ARAD1A03740g	Phy004C3O9_BLAAD	ARAD1C15642g
Phy004C1TO_BLAAD	ARAD1A04686g	Phy004C3OD_BLAAD	ARAD1C15730g
Phy004C20E_BLAAD	ARAD1A10208g	Phy004C3Q6_BLAAD	ARAD1C17248g
Phy004C21E_BLAAD	ARAD1A10978g	Phy004C3Q7_BLAAD	ARAD1C17314g
Phy004C21F_BLAAD	ARAD1A11000g	Phy004C3Q8_BLAAD	ARAD1C17358g
Phy004C21G_BLAAD	ARAD1A11022g	Phy004C3RN_BLAAD	ARAD1C18502g
Phy004C21R_BLAAD	ARAD1A11264g	Phy004C3RO_BLAAD	ARAD1C18524g
Phy004C21S_BLAAD	ARAD1A11286g	Phy004C3T6_BLAAD	ARAD1C19734g
Phy004C21T_BLAAD	ARAD1A11308g	Phy004C3T8_BLAAD	ARAD1C19778g
Phy004C21U_BLAAD	ARAD1A11330g	Phy004C3TD_BLAAD	ARAD1C19910g
Phy004C21V_BLAAD	ARAD1A11352g	Phy004C3U3_BLAAD	ARAD1C20482g
Phy004C237_BLAAD	ARAD1A12430g	Phy004C3UX_BLAAD	ARAD1C21142g
Phy004C238_BLAAD	ARAD1A12452g	Phy004C3UY_BLAAD	ARAD1C21164g
Phy004C24L_BLAAD	ARAD1A13552g	Phy004C3UZ_BLAAD	ARAD1C21186g
Phy004C24M_BLAAD	ARAD1A13574g	Phy004C3WD_BLAAD	ARAD1C22308g
Phy004C24N_BLAAD	ARAD1A13596g	Phy004C3WE_BLAAD	ARAD1C22330g
Phy004C24O_BLAAD	ARAD1A13618g	Phy004C3WO_BLAAD	ARAD1C22550g
Phy004C24P_BLAAD	ARAD1A13640g	Phy004C3WP_BLAAD	ARAD1C22572g
Phy004C24Q_BLAAD	ARAD1A13662g	Phy004C3WQ_BLAAD	ARAD1C22616g
Phy004C24R_BLAAD	ARAD1A13684g	Phy004C3WR_BLAAD	ARAD1C22638g
Phy004C267_BLAAD	ARAD1A15070g	Phy004C40I_BLAAD	ARAD1C25762g
Phy004C2AZ_BLAAD	ARAD1A19008g	Phy004C40J_BLAAD	ARAD1C25784g
Phy004C2F9_BLAAD	ARAD1B03014g	Phy004C4J5_BLAAD	ARAD1C41228g
Phy004C2FA_BLAAD	ARAD1B03036g	Phy004C4OV_BLAAD	ARAD1D00264g
Phy004C2FP_BLAAD	ARAD1B03388g	Phy004C4UG_BLAAD	ARAD1D04906g
Phy004C2FY_BLAAD	ARAD1B03586g	Phy004C4UH_BLAAD	ARAD1D04950g
Phy004C2IP_BLAAD	ARAD1B05830g	Phy004C4UI_BLAAD	ARAD1D04972g
Phy004C2IQ_BLAAD	ARAD1B05852g	Phy004C53C_BLAAD	ARAD1D12210g
Phy004C2JX_BLAAD	ARAD1B06820g	Phy004C53D_BLAAD	ARAD1D12232g
Phy004C2OC_BLAAD	ARAD1B10406g	Phy004C53E_BLAAD	ARAD1D12254g
Phy004C2OO_BLAAD	ARAD1B10736g	Phy004C53F_BLAAD	ARAD1D12298g
Phy004C2OP_BLAAD	ARAD1B10758g	Phy004C53G_BLAAD	ARAD1D12320g
Phy004C2T5_BLAAD	ARAD1B14498g	Phy004C5L9_BLAAD	ARAD1D27280g
Phy004C2UK_BLAAD	ARAD1B15620g	Phy004C5LS_BLAAD	ARAD1D27786g
Phy004C2Y2_BLAAD	ARAD1B18480g	Phy004C5LT_BLAAD	ARAD1D27808g
Phy004C2Y3_BLAAD	ARAD1B18502g	Phy004C5LU_BLAAD	ARAD1D27830g
Phy004C3ER_BLAAD	ARAD1C07766g	Phy004C5NQ_BLAAD	ARAD1D29436g
Phy004C3ES_BLAAD	ARAD1C07788g	Phy004C5RS_BLAAD	ARAD1D32692g
Phy004C3ET_BLAAD	ARAD1C07810g	Phy004C6BX_BLAAD	ARAD1D49192g
Phy004C3EU_BLAAD	ARAD1C07832g	Phy004C6BY_BLAAD	ARAD1D49214g
Phy004C3EV_BLAAD	ARAD1C07876g	Phy004C6BZ_BLAAD	ARAD1D49236g
Phy004C3F2_BLAAD	ARAD1C08030g	Phy004C6C0_BLAAD	ARAD1D49280g
Phy004C3LM_BLAAD	ARAD1C13442g	Phy004C6C1_BLAAD	ARAD1D49302g
Phy004C3LN_BLAAD	ARAD1C13464g		

Expansion_1 Probable aspartic-type endopeptidase OPSB | Aspartic proteinase MKC

Phy004C2UC_BLAAD	ARAD1B15444g	Phy004C3M4_BLAAD	ARAD1C13838g
Phy004C2UV_BLAAD	ARAD1B15884g	Phy004C4VR_BLAAD	ARAD1D06006g
Phy004C3M1_BLAAD	ARAD1C13772g	Phy004C57O_BLAAD	ARAD1D15730g
Phy004C3M2_BLAAD	ARAD1C13794g	Phy004C69B_BLAAD	ARAD1D47014g
Phy004C3M3_BLAAD	ARAD1C13816g		

Expansion_2

Phy004C1YR_BLAAD	ARAD1A08866g	Phy004C5J4_BLAAD	ARAD1D25366g
Phy004C214_BLAAD	ARAD1A10758g	Phy004C5J5_BLAAD	ARAD1D25388g
Phy004C3KY_BLAAD	ARAD1C12804g	Phy004C5J6_BLAAD	ARAD1D25410g
Phy004C3U5_BLAAD	ARAD1C20526g	Phy004C5J7_BLAAD	ARAD1D25432g
Phy004C411_BLAAD	ARAD1C26180g	Phy004C5XP_BLAAD	ARAD1D37444g
Phy004C4JY_BLAAD	ARAD1C41910g		

Expansion_3 Candidadepepsin

Phy004C24D_BLAAD ARAD1A13376g
Phy004C27G_BLAAD ARAD1A16148g
Phy004C2ND_BLAAD ARAD1B09614g
Phy004C32C_BLAAD ARAD1B22000g
Phy004C445_BLAAD ARAD1C28688g
Phy004C4OH_BLAAD ARAD1C45606g
Phy004C4YW_BLAAD ARAD1D08514g
Phy004C4YX_BLAAD ARAD1D08536g
Phy004C55U_BLAAD ARAD1D14234g
Phy004C69J_BLAAD ARAD1D47190g

Expansion_4 NADPH dehydrogenase 1

Phy004C4M1_BLAAD ARAD1C43604g
Phy004C5EV_BLAAD ARAD1D21714g
Phy004C5EW_BLAAD ARAD1D21736g
Phy004C5FL_BLAAD ARAD1D22396g
Phy004C5FM_BLAAD ARAD1D22418g
Phy004C5FN_BLAAD ARAD1D22440g
Phy004C5FO_BLAAD ARAD1D22462g
Phy004C5FP_BLAAD ARAD1D22484g

Expansion_5

Phy004C24S_BLAAD ARAD1A13706g
Phy004C2QM_BLAAD ARAD1B12298g
Phy004C2UL_BLAAD ARAD1B15642g
Phy004C3EP_BLAAD ARAD1C07700g
Phy004C3KZ_BLAAD ARAD1C12870g
Phy004C69X_BLAAD ARAD1D47498g
Phy004C6C2_BLAAD ARAD1D49324g
Phy004C6DP_BLAAD ARAD1D51216g

Expansion_6
Putative aryl-alcohol dehydrogenase C977.14c

Phy004C22T_BLAAD ARAD1A12100g
Phy004C2MW_BLAAD ARAD1B09240g
Phy004C2MX_BLAAD ARAD1B09262g
Phy004C2MY_BLAAD ARAD1B09284g
Phy004C2UD_BLAAD ARAD1B15466g
Phy004C3X3_BLAAD ARAD1C22946g
Phy004C5OZ_BLAAD ARAD1D30448g

Expansion_7 Beta-glucosidase

Phy004C1WZ_BLAAD ARAD1A07458g
Phy004C2ED_BLAAD ARAD1B01980g
Phy004C2EP_BLAAD ARAD1B02354g
Phy004C2R5_BLAAD ARAD1B12782g
Phy004C482_BLAAD ARAD1C31966g
Phy004C6B0_BLAAD ARAD1D48422g
Phy004C6B1_BLAAD ARAD1D48444g

Expansion_8
AB hydrolase superfamily protein C1039.03

Phy004C2KA_BLAAD ARAD1B07106g
Phy004C3NT_BLAAD ARAD1C15268g
Phy004C4C2_BLAAD ARAD1C35442g
Phy004C5AT_BLAAD ARAD1D18304g
Phy004C5AU_BLAAD ARAD1D18326g
Phy004C5EN_BLAAD ARAD1D21538g

Expansion_9

Phy004C2EO_BLAAD ARAD1B02288g
Phy004C2EQ_BLAAD ARAD1B02464g
Phy004C51I_BLAAD ARAD1D10692g
Phy004C5HT_BLAAD ARAD1D24310g
Phy004C642_BLAAD ARAD1D42746g
Phy004C672_BLAAD ARAD1D45166g

Expansion_10

Phy004C1PG_BLAAD ARAD1A01298g
Phy004C1PI_BLAAD ARAD1A01342g
Phy004C1PJ_BLAAD ARAD1A01364g
Phy004C2BN_BLAAD ARAD1A19536g
Phy004C2XG_BLAAD ARAD1B18018g
Phy004C368_BLAAD ARAD1C00770g

Expansion_11
NmrA-like family domain-containing protein 1

Phy004C2DL_BLAAD ARAD1B01276g
Phy004C3FS_BLAAD ARAD1C08602g
Phy004C4VX_BLAAD ARAD1D06160g
Phy004C4ZC_BLAAD ARAD1D08866g
Phy004C5RV_BLAAD ARAD1D32758g
Phy004C626_BLAAD ARAD1D41052g

Expansion_12 MFS-type transporter

Phy004C2BZ_BLAAD ARAD1A19800g
Phy004C2E0_BLAAD ARAD1B01628g
Phy004C3Q9_BLAAD ARAD1C17402g
Phy004C5B3_BLAAD ARAD1D18524g
Phy004C5B4_BLAAD ARAD1D18546g
Phy004C5FC_BLAAD ARAD1D22176g

Expansion_13
Uncharacterized oxidoreductase C736.13

Phy004C3QP_BLAAD ARAD1C17754g
Phy004C3SL_BLAAD ARAD1C19272g
Phy004C4H8_BLAAD ARAD1C39666g
Phy004C4TP_BLAAD ARAD1D04246g

Expansion_14
Cutinase transcription factor 1 alpha

Phy004C263_BLAAD ARAD1A14894g
Phy004C40W_BLAAD ARAD1C26070g
Phy004C5B9_BLAAD ARAD1D18656g
Phy004C5NZ_BLAAD ARAD1D29656g

Expansion_15 High-affinity glucose transporter

Phy004C26D_BLAAD ARAD1A15246g
Phy004C2QT_BLAAD ARAD1B12496g
Phy004C2QU_BLAAD ARAD1B12518g
Phy004C513_BLAAD ARAD1D10362g

Expansion_16 ATP-dependent DNA helicase Q1
Phy004C1O0_BLAAD ARAD1A00110g
Phy004C24U_BLAAD ARAD1A13750g
Phy004C35D_BLAAD ARAD1B24420g
Phy004C35E_BLAAD ARAD1B24442g
Phy004C6BW_BLAAD ARAD1D49170g
Phy004C6DV_BLAAD ARAD1D51348g

Expansion_17 Glycoprotein-N-acetylgalactosamine 3-beta-galactosyltransferase 1-A
Phy004C5C9_BLAAD ARAD1D19558g
Phy004C5DM_BLAAD ARAD1D20636g
Phy004C695_BLAAD ARAD1D46860g
Phy004C696_BLAAD ARAD1D46882g

Expansion_18 NADPH-dependent methylglyoxal reductase GRE2 | Putative uncharacterized oxidoreductase YGL039W | NADPH-dependent aldehyde reductase ARI1
Phy004C21O_BLAAD ARAD1A11198g
Phy004C2F2_BLAAD ARAD1B02860g
Phy004C2GM_BLAAD ARAD1B04136g
Phy004C3T7_BLAAD ARAD1C19756g

Expansion_19 Beta-glucosidase 2
Phy004C26J_BLAAD ARAD1A15422g
Phy004C466_BLAAD ARAD1C30404g
Phy004C46E_BLAAD ARAD1C30558g
Phy004C560_BLAAD ARAD1D14366g

Expansion_20 Sugar transporter STL1
Phy004C1W3_BLAAD ARAD1A06754g
Phy004C25A_BLAAD ARAD1A14102g
Phy004C5B8_BLAAD ARAD1D18634g
Phy004C5NY_BLAAD ARAD1D29634g

Expansion_21
Phy004C29T_BLAAD ARAD1A18084g
Phy004C4BB_BLAAD ARAD1C34848g
Phy004C583_BLAAD ARAD1D16060g
Phy004C5JE_BLAAD ARAD1D25586g

Expansion_22 NADPH-dependent 1-acyldihydroxyacetone phosphate reductase
Phy004C2BB_BLAAD ARAD1A19272g
Phy004C41N_BLAAD ARAD1C26686g
Phy004C41O_BLAAD ARAD1C26708g
Phy004C5ZP_BLAAD ARAD1D39072g

Expansion_23 Probable ferric reductase transmembrane component
Phy004C1O7_BLAAD ARAD1A00264g
Phy004C1O8_BLAAD ARAD1A00286g
Phy004C5K1_BLAAD ARAD1D26136g
Phy004C5YH_BLAAD ARAD1D38060g

Expansion_24 Lipase
Phy004C2MB_BLAAD ARAD1B08712g
Phy004C507_BLAAD ARAD1D09636g
Phy004C56J_BLAAD ARAD1D14784g
Phy004C5NW_BLAAD ARAD1D29590g

Expansion_25 Cytochrome P450 52E1
Phy004C4WS_BLAAD ARAD1D06842g
Phy004C65A_BLAAD ARAD1D43758g
Phy004C65B_BLAAD ARAD1D43780g
Phy004C67L_BLAAD ARAD1D45628g

Expansion_26 UPF0420 protein
Phy004C2IN_BLAAD ARAD1B05786g
Phy004C2TG_BLAAD ARAD1B14740g
Phy004C3US_BLAAD ARAD1C21032g
Phy004C47S_BLAAD ARAD1C31702g

Expansion_27
Phy004C4QH_BLAAD ARAD1D01584g
Phy004C4QL_BLAAD ARAD1D01672g
Phy004C4QM_BLAAD ARAD1D01694g
Phy004C58O_BLAAD ARAD1D16566g

Expansion_28
Heat shock protein 42 | Heat shock protein 16
Phy004C1W2_BLAAD ARAD1A06732g
Phy004C4AZ_BLAAD ARAD1C34584g
Phy004C4O2_BLAAD ARAD1C45276g
Phy004C53A_BLAAD ARAD1D12166g

Expansion_29 Glutathione S-transferase
Phy004C3SV_BLAAD ARAD1C19492g
Phy004C5Z7_BLAAD ARAD1D38676g
Phy004C6BN_BLAAD ARAD1D48972g

Expansion_30 Ammonia transport outward protein 2 | Meiotically up-regulated gene 86 protein | Accumulation of dyads protein 2
Phy004C1PC_BLAAD ARAD1A01188g
Phy004C28S_BLAAD ARAD1A17248g
Phy004C657_BLAAD ARAD1D43692g

Expansion_31 Polyamine transporter 1
Phy004C48O_BLAAD ARAD1C32472g
Phy004C4PW_BLAAD ARAD1D01100g
Phy004C4RY_BLAAD ARAD1D02772g

Expansion_32
Phy004C2HQ_BLAAD ARAD1B05038g
Phy004C2HR_BLAAD ARAD1B05060g
Phy004C37N_BLAAD ARAD1C01936g
Phy004C3GG_BLAAD ARAD1C09152g

Expansion_33
Long-chain-fatty-acid--CoA ligase 2
Phy004C1QA_BLAAD ARAD1A01936g
Phy004C1ZW_BLAAD ARAD1A09768g
Phy004C3MC_BLAAD ARAD1C14014g

Expansion_34
Uncharacterized MFS-type transporter C947.06c
Phy004C3GR_BLAAD ARAD1C09394g
Phy004C4DN_BLAAD ARAD1C36806g
Phy004C4EQ_BLAAD ARAD1C37686g

Expansion_35
Corticosteroid-binding protein | Polyamine oxidase FMS1
Phy004C564_BLAAD ARAD1D14454g
Phy004C5E8_BLAAD ARAD1D21142g
Phy004C6AH_BLAAD ARAD1D47982g

Expansion_36 Oligopeptide transporter 2
Phy004C2DX_BLAAD ARAD1B01540g
Phy004C2T8_BLAAD ARAD1B14564g
Phy004C3IP_BLAAD ARAD1C10934g

Expansion_37
3-oxoacyl-[acyl-carrier-protein] reductase
Phy004C1ZR_BLAAD ARAD1A09658g
Phy004C4IJ_BLAAD ARAD1C40722g
Phy004C4NE_BLAAD ARAD1C44704g

Expansion_38
Uncharacterized MFS-type transporter PB1E7.08c
Phy004C4C6_BLAAD ARAD1C35530g
Phy004C4S5_BLAAD ARAD1D02948g
Phy004C6DB_BLAAD ARAD1D50908

Expansion_39 Sorbose reductase SOU1
Phy004C34K_BLAAD ARAD1B23848g
Phy004C5BM_BLAAD ARAD1D18942g
Phy004C5KT_BLAAD ARAD1D26862g

Expansion_40
Peptidase M20 domain-containing protein 2
Phy004C2QB_BLAAD ARAD1B12056g
Phy004C4AF_BLAAD ARAD1C33968g
Phy004C65K_BLAAD ARAD1D43978g

Expansion_41 Protein SNQ2
Phy004C231_BLAAD ARAD1A12298g
Phy004C316_BLAAD ARAD1B20988g
Phy004C5DQ_BLAAD ARAD1D20724g

Expansion_42 Uncharacterized oxidoreductase yrbE
Phy004C259_BLAAD ARAD1A14080g
Phy004C5B6_BLAAD ARAD1D18590g
Phy004C5NV_BLAAD ARAD1D29568g

Expansion_43 GABA-specific permease
Phy004C30V_BLAAD ARAD1B20746g
Phy004C3JT_BLAAD ARAD1C11814g
Phy004C4TQ_BLAAD ARAD1D04268g

Expansion_44 Lipase 4
Phy004C2IM_BLAAD ARAD1B05764g
Phy004C4AV_BLAAD ARAD1C34496g
Phy004C638_BLAAD ARAD1D41954g

Expansion_45 Quinic acid utilization activator
Phy004C35O_BLAAD ARAD1C00308g
Phy004C4T3_BLAAD ARAD1D03696g
Phy004C5AY_BLAAD ARAD1D18414g

Expansion_46
Phy004C20F_BLAAD ARAD1A10230g
Phy004C2MI_BLAAD ARAD1B08932g
Phy004C68I_BLAAD ARAD1D46354g

Expansion_47 Probable phosphomutase PMU1
Phy004C30F_BLAAD ARAD1B20372g
Phy004C321_BLAAD ARAD1B21736g
Phy004C5W3_BLAAD ARAD1D36168g

Expansion_48
Carboxylic acid transporter protein homolog
Phy004C3F1_BLAAD ARAD1C08008g
Phy004C55R_BLAAD ARAD1D14168g
Phy004C69U_BLAAD ARAD1D47432g

Expansion_49 Probable cutinase 1
Phy004C2JY_BLAAD ARAD1B06842g
Phy004C2JZ_BLAAD ARAD1B06864g
Phy004C3R0_BLAAD ARAD1C17996g

Expansion_50 Maltose permease MAL31
Phy004C35M_BLAAD ARAD1C00264g
Phy004C4OP_BLAAD ARAD1C45782g
Phy004C5AK_BLAAD ARAD1D18106g

Expansion_51
Phy004C392_BLAAD ARAD1C03080g
Phy004C3H9_BLAAD ARAD1C09790g
Phy004C417_BLAAD ARAD1C26312g

Expansion_52
Phy004C2E2_BLAAD ARAD1B01694g
Phy004C2Z9_BLAAD ARAD1B19426g
Phy004C2ZA_BLAAD ARAD1B19448g

Expansion_53 3-phytase B
Phy004C209_BLAAD ARAD1A10054g
Phy004C2I2_BLAAD ARAD1B05324g
Phy004C4VM_BLAAD ARAD1D05896g

Expansion_54 Glycolipid 2-alpha-mannosyltransferase
Phy004C2DZ_BLAAD ARAD1B01606g
Phy004C3SM_BLAAD ARAD1C19294g
Phy004C605_BLAAD ARAD1D39424g

Expansion_55 Zinc-type alcohol dehydrogenase-like protein C1773.06c

Phy004C216_BLAAD ARAD1A10802g
Phy004C3OG_BLAAD ARAD1C15796g
Phy004C5K2_BLAAD ARAD1D26158g

Expansion_56 Aldehyde dehydrogenase

Phy004C2GC_BLAAD ARAD1B03916g
Phy004C2GD_BLAAD ARAD1B03938g
Phy004C6DE_BLAAD ARAD1D50974g

Expansion_57 Sterol uptake control protein 2

Phy004C25X_BLAAD ARAD1A14630g
Phy004C2WP_BLAAD ARAD1B17402g
Phy004C5AM_BLAAD ARAD1D18150g

Expansion_58 Nitrate transporter

Phy004C3SI_BLAAD ARAD1C19206g
Phy004C3SK_BLAAD ARAD1C19250g
Phy004C42U_BLAAD ARAD1C27632g

Expansion_59

Phy004C1O1_BLAAD ARAD1A00132g
Phy004C1O6_BLAAD ARAD1A00242g
Phy004C2CB_BLAAD ARAD1B00198g

Expansion_60 Maltose permease MAL31

Phy004C1ZV_BLAAD ARAD1A09746g
Phy004C508_BLAAD ARAD1D09658g
Phy004C5VJ_BLAAD ARAD1D35728g

Expansion_61

Phy004C2RA_BLAAD ARAD1B12892g
Phy004C2ZO_BLAAD ARAD1B19756g
Phy004C68P_BLAAD ARAD1D46508g

Figure S9C

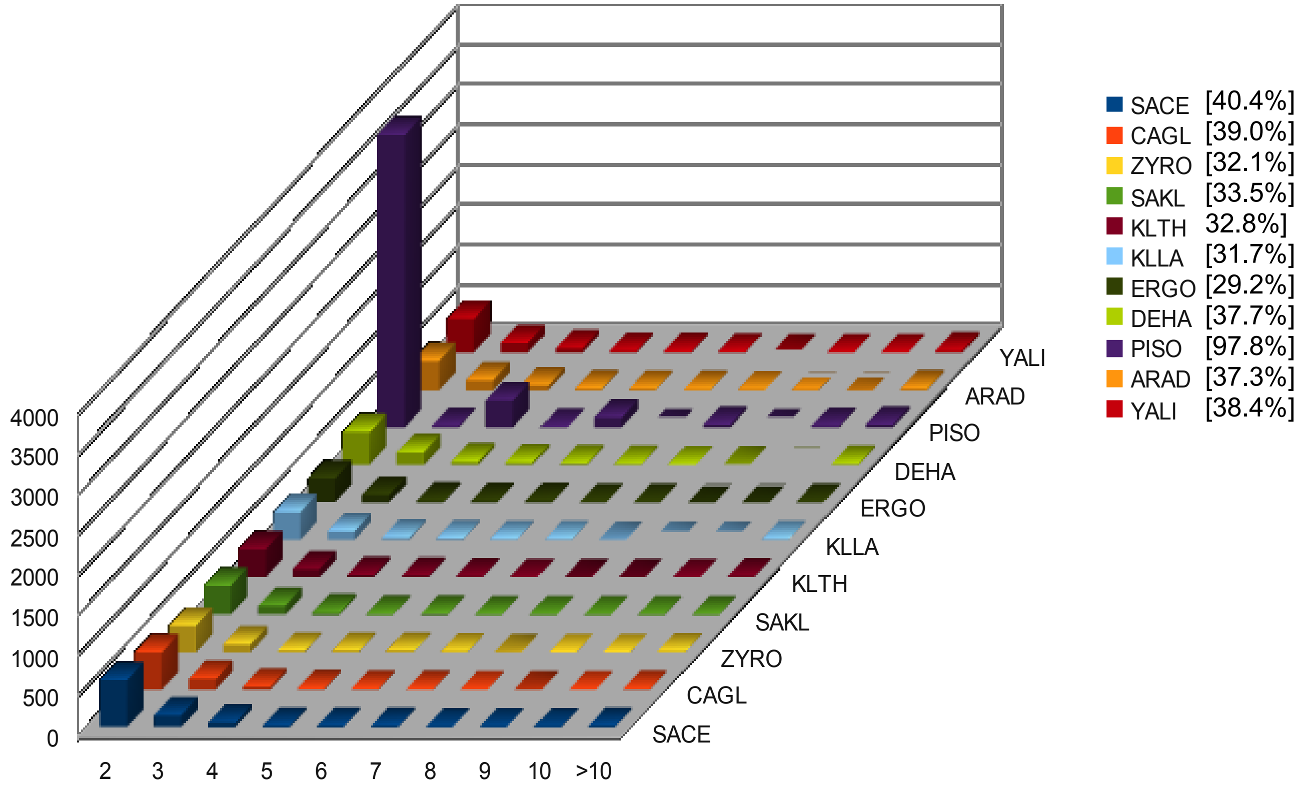
4000
3500
3000
2500
2000
1500
1000
500
0
2
3
4
5
6
7
8
9
10
>10
SACE
CAGL
ZYRO
SAKL
KLTH
KLLA
ERGO
DEHA
PISO
ARAD
YALI
SACE [40.4%]
CAGL [39.0%]
ZYRO [32.1%]
SAKL [33.5%]
KLTH 32.8%]
KLLA [31.7%]
ERGO [29.2%]
DEHA [37.7%]
PISO [97.8%]
ARAD [37.3%]
YALI [38.4%]

Table S9D Phyletic patterns of yeast paralogous protein families

	##Families	## proteins
**Families universal to all**		
sczltkgdpay	2243	39756
**Families missing in one species**		
-czltkgdpay	7	7
s-zltkgdpay	20	21
sc-ltkgdpay	12	12
scz-tkgdpay	8	8
sczl-kgdpay	9	9
sczlt-gdpay	24	24
sczltk-dpay	54	58
sczltkg-pay	28	28
sczltkgd-ay	11	11
sczltkgdp-y	262	263
sczltkgdpa-	101	102
**Families missing in 2 species (a)**		
sczltkgdp--	135	136
sczltkgd--y	15	15
--zltkgdpay	10	10
s-zltk-dpay	9	9
s-zlt-gdpay	9	9
sczltkg-pa-	9	9
**Species-specific families**		
s----------	42	158
-c---------	5	37
--z--------	7	44
---l-------	7	22
----t------	5	13
-----k-----	9	31
------g----	3	7
-------d---	42	181
--------p--	372	760
---------a-	53	230
----------y	93	322
**Families restricted to 2 species(b)**		
-------dp--	358	1109
--------pa-	68	226
---------ay	18	81
-------d-a-	7	33
--------p-y	6	20
**Other families**		
all other combinations patterns	599	4661

(a) only the six best pattern families are reported, (b) only the 5 best pattern families are reported.

Figure S9E

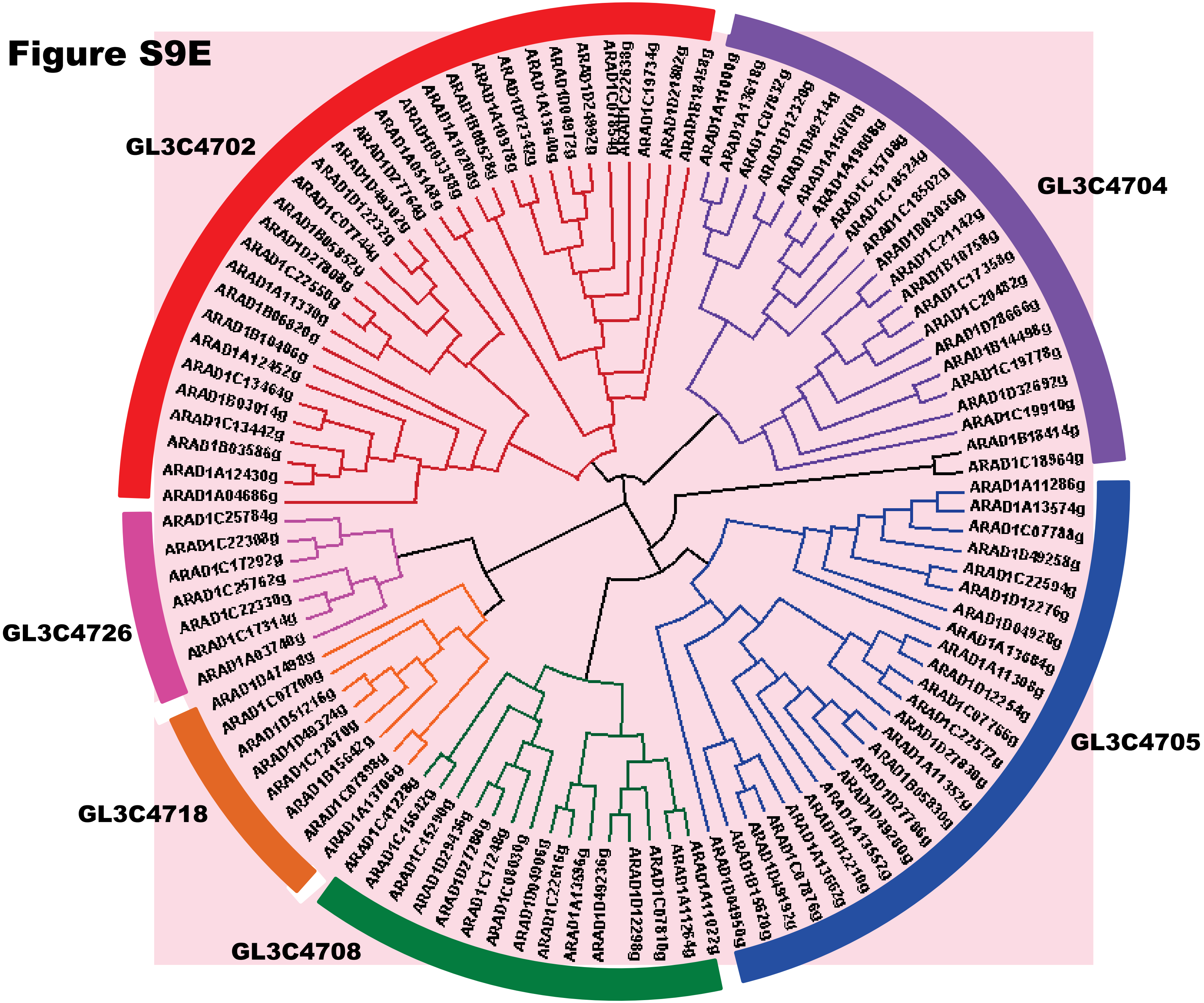

Supplement: Additional file 9 — Gene families amplified in A. adeninivorans. [file 1754-6834-7-66-S9.pdf]
