# Supplementary material for: The complete genome of Blastobotrys (Arxula) adeninivorans LS3 - a yeast of biotechnological interest
Source: Biotechnol Biofuels. 2014 Apr 24;7:66. doi: 10.1186/1754-6834-7-66 (PMC4022394; doi:10.1186/1754-6834-7-66)

# Additional File 10. Transporter distribution in hemiascomycete species

Transporters were identified through a two step process on the proteomes of *S. cerevisiae, C. glabrata, K. lactis, D. hansenii, K. pastoris, Y. lipolytica* and *A. adeninivorans*: 1) identification of protein products having at least three transmembrane domains using THMM (v2.0; http://www.cbs.dtu.dk/services/TMHMM-2.0), 2) identification of transporters by a Blastp search against the TCDB database[16].

**Table S10A Transporter distribution in hemiascomycete species.**

**Table S10B TCDB classification of transporters of *A. adeninivorans*.**

**Table S10C Class of transporters amplified in *A. adeninivorans* compared to two other pre-CTG species.**

**Figure S10D Phylogeny of sugar porters in *A. adeninivorans* and *D. hansenii.***

# Table S10A  Transporter distribution in hemiascomycete species

| Type | Subfamily code TCDB | Species ARAD | YALI | PIPA | DEHA | KLLA | CAGL | SACE | Total | TCDB Family |
|---|---|---|---|---|---|---|---|---|---|---|
| α-Type Channels | 1.A.1.10 | **1** | | | | | | | 1 | The Voltage-gated Ion Channel (VIC) Family |
| | 1.A.1.11 | **1** | 1 | 1 | 1 | 1 | 1 | 1 | 7 | The Voltage-gated Ion Channel (VIC) Family |
| | 1.A.1.5 | **1** | | | | | | | 1 | The Voltage-gated Ion Channel (VIC) Family |
| | 1.A.1.7 | **1** | 2 | | | 1 | 1 | 1 | 6 | The Voltage-gated Ion Channel (VIC) Family |
| | 1.A.11.1 | | 1 | | 1 | 1 | 1 | 1 | 5 | The Ammonia Transporter Channel (Amt) Family |
| | 1.A.11.2 | | 2 | | 2 | | | | 4 | The Ammonia Transporter Channel (Amt) Family |
| | 1.A.11.3 | **2** | | 3 | | | | | 5 | The Ammonia Transporter Channel (Amt) Family |
| | 1.A.15.1 | | 1 | | 1 | 1 | 1 | 1 | 5 | The Non-selective Cation Channel-2 (NSCC2) Family |
| | 1.A.15.2 | | | 1 | | | | | 1 | The Non-selective Cation Channel-2 (NSCC2) Family |
| | 1.A.16.1 | | 1 | | 1 | 1 | 1 | 1 | 5 | The Yeast Stretch-Activated, Cation-Selective, Ca2+ Channel, Mid1 Family |
| | 1.A.17.2 | **1** | | | | | | | 1 | The Calcium-Dependent Chloride Channel (Ca-ClC) Family |
| | 1.A.17.3 | | | 1 | | | | | 1 | The Calcium-Dependent Chloride Channel (Ca-ClC) Family |
| | 1.A.20.5 | | 11 | | 7 | 5 | 1 | 7 | 31 | The BCL2/Adenovirus E1B-interacting Protein 3 (BNip3) Family |
| | 1.A.20.Y1 | | 1 | | | | | | 1 | The BCL2/Adenovirus E1B-interacting Protein 3 (BNip3) Family |
| | 1.A.20.Y2 | | 1 | | 1 | | | | 2 | The BCL2/Adenovirus E1B-interacting Protein 3 (BNip3) Family |
| | 1.A.31.Y1 | | 1 | | | | | | 1 | The Annexin (Annexin) Family |
| | 1.A.33.1 | | 10 | | 9 | 9 | 11 | 14 | 53 | The Cation Channel-forming Heat Shock Protein-70 (Hsp70) Family |
| | 1.A.35.2 | **2** | 3 | | 2 | 2 | 2 | 3 | 14 | The CorA Metal Ion Transporter (MIT) Family |
| | 1.A.35.5 | **1** | 2 | | 2 | 2 | 2 | 2 | 11 | The CorA Metal Ion Transporter (MIT) Family |
| | 1.A.38.1 | **1** | | 1 | | | | | 2 | The Golgi pH Regulator (GPHR) Family |
| | 1.A.4.4 | **3** | 1 | 1 | 1 | 1 | 1 | 2 | 10 | The Transient Receptor Potential Ca2+ Channel (TRP-CC) Family |
| | 1.A.4.Y1 | | 2 | | | | | | 2 | The Transient Receptor Potential Ca2+ Channel (TRP-CC) Family |
| | 1.A.55.4 | **1** | | | | | | | 1 | The Synaptic Vesicle-Associated Ca2+ Channel, Flower Family |
| | 1.A.56.1 | **2** | | | | | | | 2 | The Copper Transporter (Ctr) Family |
| | 1.A.6.Y1 | | 1 | | 1 | | | | 2 | The Epithelial Na+ Channel (ENaC) Family |
| | 1.A.6.Y2 | | 1 | | 1 | | | | 2 | The Epithelial Na+ Channel (ENaC) Family |
| | 1.A.8.5 | | | | | 1 | 2 | 1 | 4 | The Major Intrinsic Protein (MIP) Family |
| | 1.A.8.6 | **1** | 1 | 1 | 1 | 1 | 2 | 3 | 10 | The Major Intrinsic Protein (MIP) Family |
| | 1.A.8.7 | | 2 | 1 | | | | 1 | 4 | The Major Intrinsic Protein (MIP) Family |
| | 1.A.X1.Y1 | | 1 | | 1 | | | | 2 | Unassigned |
| β-Barrel Porins | 1.B.40.1 | **2** | | | | | | | 2 | The Autotransporter-2 (AT-2) Family |
| | 1.B.53.1 | **1** | | | | | | | 1 | The Filamentous Phage gp3 Channel-Forming Protein (FP-gp3) Family |
| | 1.B.8.1 | | 1 | | 1 | 1 | 2 | 2 | 7 | The Mitochondrial and Plastid Porin (MPP) Family |
| | 2.A.1.1 | **60** | 27 | 17 | 48 | 20 | 17 | 34 | 223 | Sugar Porter (SP) Family |
| | 2.A.1.11 | **1** | | 2 | | | | | 3 | Oxalate: Formate Antiporter (OFA) Family |
| | 2.A.1.12 | **4** | 6 | 1 | 3 | 2 | | 1 | 17 | Sialate: H+ Symporter (SHS) Family |
| | 2.A.1.13 | **8** | 5 | 3 | 8 | 12 | 3 | | 39 | Monocarboxylate Porter (MCP) Family (Halestrap, 2011) |
| | 2.A.1.14 | **18** | 39 | 14 | 27 | 13 | 6 | 10 | 127 | Anion: Cation Symporter (ACS) Family |
| | 2.A.1.15 | **1** | | | | | | | 1 | Aromatic Acid: H+ Symporter (AAHS) Family |
| | 2.A.1.16 | **6** | 14 | 3 | 5 | 4 | 1 | 6 | 39 | Siderophore-Iron Transporter (SIT) Family |
| | 2.A.1.19 | **1** | | | | | | | 1 | Organic Cation Transporter (OCT) Family |

| | | | | | | | | | |
|---|---|---|---|---|---|---|---|---|---|
| 2.A.1.2 | **39** | 33 | 10 | 24 | 8 | 10 | 12 | 136 | Drug: H+ Antiporter-1 (12 Spanner) (DHA1) Family |
| 2.A.1.22 | **2** | | | | | | | 2 | Vesicular Neurotransmitter Transporter (VNT) Family |
| 2.A.1.24 | **2** | 1 | 1 | | 1 | 1 | 1 | 7 | Unknown Major Facilitator-1 (UMF1) Family |
| 2.A.1.25 | **1** | 1 | 1 | 1 | 1 | 1 | 1 | 7 | Peptide-Acetyl-Coenzyme A Transporter (PAT) Family |
| 2.A.1.28 | **1** | 1 | | | | | | 2 | Feline Leukemia Virus Subgroup C Receptor (FLVCR) Family |
| 2.A.1.3 | **9** | 7 | 2 | 7 | 6 | 5 | 10 | 46 | Drug: H+ Antiporter-2 (14 Spanner) (DHA2) Family |
| 2.A.1.43 | | | 1 | | | | | 1 | The Putative Magnetosome Permease (PMP) Family |
| 2.A.1.44 | **1** | | 2 | | | | | 3 | L-Amino Acid Transporter-3 (LAT3) Family (also called the SLC43 family) |
| 2.A.1.48 | **1** | | 3 | | | | | 4 | Vacuolar Basic Amino Acid Transporter (V-BAAT) Family |
| 2.A.1.58 | **3** | | 1 | | | | | 4 | N-Acetylglucosamine Transporter (NAG-T) Family |
| 2.A.1.63 | **1** | | 1 | | | | | 2 | Unidentified Major Facilitator-12 (UMF12) Family |
| 2.A.1.7 | **2** | | | | | | | 2 | Fucose: H+ Symporter (FHS) Family |
| 2.A.1.8 | **3** | | | | | | | 3 | Nitrate/Nitrite Porter (NNP) family |
| 2.A.1.9 | **3** | 5 | 2 | 3 | 3 | 2 | 2 | 20 | Phosphate: H+ Symporter (PHS) Family |
| 2.A.1.Y1 | | 5 | | 1 | 4 | 2 | 4 | 16 | Unassigned |
| 2.A.1.Y2 | | 1 | | 1 | | | 1 | 3 | Unassigned |
| 2.A.1.Y3 | | 3 | | 1 | | | | 4 | Unassigned |
| 2.A.16.3 | | | 1 | | | | | 1 | The Tellurite-resistance/Dicarboxylate Transporter (TDT) Family |
| 2.A.16.4 | **1** | | | | | | | 1 | The Telurite-resistance/Dicarboxylate Transporter (TDT) Family |
| 2.A.16.Y1 | | 1 | | 3 | 1 | 1 | 1 | 7 | The Telurite-resistance/Dicarboxylate Transporter (TDT) Family |
| 2.A.17.2 | **2** | 7 | | 3 | 1 | 1 | 1 | 15 | The Proton-dependent Oligopeptide Transporter (POT) Family |
| 2.A.18.4 | **4** | 1 | | 3 | 2 | | | 10 | The Amino Acid/Auxin Permease (AAAP) Family |
| 2.A.18.5 | **1** | 1 | 1 | 2 | 1 | 1 | 1 | 8 | The Amino Acid/Auxin Permease (AAAP) Family |
| 2.A.18.6 | **2** | 2 | 3 | 3 | 3 | 3 | 4 | 20 | The Amino Acid/Auxin Permease (AAAP) Family |
| 2.A.18.7 | **1** | 1 | 2 | 2 | 2 | 3 | 2 | 13 | The Amino Acid/Auxin Permease (AAAP) Family |
| 2.A.18.Y1 | | 3 | | 1 | | | | 4 | The Amino Acid/Auxin Permease (AAAP) Family |
| 2.A.19.2 | **1** | 2 | 1 | 1 | 2 | 2 | 1 | 10 | The Ca2+:Cation Antiporter (CaCA) Family |
| 2.A.19.4 | **1** | | 2 | | | | | 3 | The Ca2+:Cation Antiporter (CaCA) Family |
| 2.A.19.7 | **1** | | 1 | | | | | 2 | The Ca2+:Cation Antiporter (CaCA) Family |
| 2.A.19.Y1 | | | | 1 | 1 | | 1 | 3 | The Ca2+:Cation Antiporter (CaCA) Family |
| 2.A.2.6 | **2** | 1 | 1 | | | | | 4 | The Glycoside-Pentoside-Hexuronide (GPH):Cation Symporter Family |
| 2.A.20.2 | **1** | 1 | 1 | 1 | 1 | | 1 | 6 | The Inorganic Phosphate Transporter (PiT) Family |
| 2.A.21.6 | **2** | 4 | 5 | 3 | 3 | 1 | 1 | 19 | The Solute:Sodium Symporter (SSS) Family |
| 2.A.21.9 | **2** | | | | | | | 2 | The Solute:Sodium Symporter (SSS) Family |
| 2.A.29.1 | **1** | 3 | | 1 | 1 | 1 | 3 | 10 | The Mitochondrial Carrier (MC) Family |
| 2.A.29.10 | **1** | | 1 | 1 | 1 | 1 | 1 | 6 | The Mitochondrial Carrier (MC) Family |
| 2.A.29.12 | **1** | | 1 | | | | | 2 | The Mitochondrial Carrier (MC) Family |
| 2.A.29.13 | | 1 | | 2 | 3 | 3 | 2 | 11 | The Mitochondrial Carrier (MC) Family |
| 2.A.29.14 | | 1 | | | | | 1 | 2 | The Mitochondrial Carrier (MC) Family |
| 2.A.29.15 | | 1 | | 1 | 1 | 1 | 1 | 5 | The Mitochondrial Carrier (MC) Family |
| 2.A.29.17 | **2** | 2 | | 1 | 1 | 1 | 1 | 8 | The Mitochondrial Carrier (MC) Family |
| 2.A.29.18 | **3** | 1 | | 1 | 1 | 1 | 1 | 8 | The Mitochondrial Carrier (MC) Family |
| 2.A.29.2 | **1** | 2 | | 2 | 1 | 2 | 3 | 11 | The Mitochondrial Carrier (MC) Family |
| 2.A.29.27 | **1** | | | | | | | 1 | The Mitochondrial Carrier (MC) Family |
| 2.A.29.4 | **1** | 2 | | 2 | 1 | 1 | 2 | 9 | The Mitochondrial Carrier (MC) Family |

| | | | | | | | | | | |
|---|---|---|---|---|---|---|---|---|---|---|
| Porters (uniporters, symporters, antiporters) | 2.A.29.5 | | 1 | | 1 | 1 | 2 | 2 | 7 | The Mitochondrial Carrier (MC) Family |
| | 2.A.29.6 | **1** | 1 | 1 | 1 | 1 | | | 5 | The Mitochondrial Carrier (MC) Family |
| | 2.A.29.7 | | 3 | | 3 | 2 | 3 | 2 | 13 | The Mitochondrial Carrier (MC) Family |
| | 2.A.29.8 | **1** | 1 | 2 | 1 | 1 | 1 | 1 | 8 | The Mitochondrial Carrier (MC) Family |
| | 2.A.29.9 | | | | 1 | 1 | 1 | 1 | 4 | The Mitochondrial Carrier (MC) Family |
| | 2.A.29.Y1 | | 1 | | 1 | 1 | 1 | 1 | 5 | The Mitochondrial Carrier (MC) Family |
| | 2.A.29.Y10 | | 1 | | 1 | 1 | 1 | 1 | 5 | The Mitochondrial Carrier (MC) Family |
| | 2.A.29.Y11 | | | | | | 1 | 1 | 2 | The Mitochondrial Carrier (MC) Family |
| | 2.A.29.Y12 | | 1 | | 1 | 1 | 1 | 1 | 5 | The Mitochondrial Carrier (MC) Family |
| | 2.A.29.Y13 | | 1 | | 1 | 1 | 1 | 1 | 5 | The Mitochondrial Carrier (MC) Family |
| | 2.A.29.Y14 | | | | 1 | 1 | 1 | | 3 | The Mitochondrial Carrier (MC) Family |
| | 2.A.29.Y15 | | | | | | 1 | | 1 | The Mitochondrial Carrier (MC) Family |
| | 2.A.29.Y16 | | 1 | | | | | | 1 | The Mitochondrial Carrier (MC) Family |
| | 2.A.29.Y17 | | 1 | | 1 | 1 | | | 3 | The Mitochondrial Carrier (MC) Family |
| | 2.A.29.Y18 | | 1 | | | | | | 1 | The Mitochondrial Carrier (MC) Family |
| | 2.A.29.Y19 | | 2 | | | | | | 2 | The Mitochondrial Carrier (MC) Family |
| | 2.A.29.Y2 | | | | 1 | 1 | 1 | 1 | 4 | The Mitochondrial Carrier (MC) Family |
| | 2.A.29.Y20 | | 1 | | 1 | | | | 2 | The Mitochondrial Carrier (MC) Family |
| | 2.A.29.Y21 | | 1 | | 2 | | | | 3 | The Mitochondrial Carrier (MC) Family |
| | 2.A.29.Y22 | | 1 | | | | | | 1 | The Mitochondrial Carrier (MC) Family |
| | 2.A.29.Y23 | | 1 | | | | | | 1 | The Mitochondrial Carrier (MC) Family |
| | 2.A.29.Y3 | | 2 | | 2 | 2 | 2 | 2 | 10 | The Mitochondrial Carrier (MC) Family |
| | 2.A.29.Y4 | | 1 | | 1 | 1 | 1 | 2 | 6 | The Mitochondrial Carrier (MC) Family |
| | 2.A.29.Y5 | | 1 | | 1 | 2 | 1 | 1 | 6 | The Mitochondrial Carrier (MC) Family |
| | 2.A.29.Y6 | | 1 | | 1 | 1 | 1 | 1 | 5 | The Mitochondrial Carrier (MC) Family |
| | 2.A.29.Y7 | | 1 | | 2 | 1 | 1 | 1 | 6 | The Mitochondrial Carrier (MC) Family |
| | 2.A.29.Y8 | | | | | 1 | 1 | 1 | 3 | The Mitochondrial Carrier (MC) Family |
| | 2.A.3.1 | **1** | 2 | 1 | | | | | 4 | The Amino Acid-Polyamine-Organocation (APC) Family |
| | 2.A.3.10 | **9** | 14 | 12 | 24 | 16 | 14 | 18 | 107 | The Amino Acid-Polyamine-Organocation (APC) Family |
| | 2.A.3.4 | **10** | 9 | 2 | 6 | 6 | 2 | 4 | 39 | The Amino Acid-Polyamine-Organocation (APC) Family |
| | 2.A.3.8 | **2** | 5 | 4 | 3 | 3 | 3 | 2 | 22 | The Amino Acid-Polyamine-Organocation (APC) Family |
| | 2.A.30.1 | | | 1 | | | | | 1 | The Cation-Chloride Cotransporter (CCC) Family |
| | 2.A.30.6 | **1** | | | | | | | 1 | The Cation-Chloride Cotransporter (CCC) Family |
| | 2.A.30.7 | | 1 | | 1 | 1 | 1 | 1 | 5 | The Cation-Chloride Cotransporter (CCC) Family |
| | 2.A.31.3 | **1** | 1 | 1 | 2 | 1 | 1 | 1 | 8 | The Anion Exchanger (AE) Family |
| | 2.A.36.2 | **1** | 1 | 1 | 1 | 1 | 1 | 1 | 7 | The Monovalent Cation: Proton Antiporter-1 (CPA1) Family |
| | 2.A.36.4 | **2** | 2 | 1 | 1 | 1 | 1 | 1 | 9 | The Monovalent Cation: Proton Antiporter-1 (CPA1) Family |
| | 2.A.37.4 | **1** | | 1 | 1 | 1 | 1 | 1 | 6 | The Monovalent Cation: Proton Antiporter-2 (CPA2) Family |
| | 2.A.37.Y1 | | | | 2 | | | | 2 | The Monovalent Cation: Proton Antiporter-2 (CPA2) Family |
| | 2.A.38.2 | **2** | 1 | 1 | | 1 | 1 | 2 | 8 | The K+ Transporter (Trk) Family |
| | 2.A.39.2 | **2** | 2 | 2 | 3 | 2 | 4 | 4 | 19 | The Nucleobase: Cation Symporter-1 (NCS1) Family |
| | 2.A.39.3 | **1** | 2 | 2 | 1 | 2 | 2 | 3 | 13 | The Nucleobase: Cation Symporter-1 (NCS1) Family |
| | 2.A.39.4 | **1** | | 1 | 1 | 2 | 3 | 3 | 11 | The Nucleobase: Cation Symporter-1 (NCS1) Family |
| | 2.A.4.1 | **1** | | | | | | | 1 | The Cation Diffusion Facilitator (CDF) Family |
| | 2.A.4.2 | **1** | 1 | 1 | 1 | 1 | 1 | 2 | 8 | The Cation Diffusion Facilitator (CDF) Family |

| | | | | | | | | | |
|---|---|---|---|---|---|---|---|---|---|
| 2.A.4.4 | **2** | 1 | 2 | 1 | 1 | 1 | 1 | 9 | The Cation Diffusion Facilitator (CDF) Family |
| 2.A.4.5 | **2** | | 1 | | | | | 3 | The Cation Diffusion Facilitator (CDF) Family |
| 2.A.4.Y1 | | 1 | | 1 | 1 | 2 | 2 | 7 | The Cation Diffusion Facilitator (CDF) Family |
| 2.A.40.5 | **1** | 2 | 1 | 1 | 1 | | | 6 | The Nucleobase: Cation Symporter-2 (NCS2) Family |
| 2.A.41.2 | **1** | 2 | | 1 | | | | 4 | The Concentrative Nucleoside Transporter (CNT) Family |
| 2.A.43.1 | **1** | | 1 | | | | | 2 | The Lysosomal Cystine Transporter (LCT) Family |
| 2.A.43.2 | | | 1 | | 1 | 1 | 1 | 4 | The Lysosomal Cystine Transporter (LCT) Family |
| 2.A.43.3 | **1** | 1 | | | | | | 2 | The Lysosomal Cystine Transporter (LCT) Family |
| 2.A.43.Y1 | | | | 1 | | | | 1 | The Lysosomal Cystine Transporter (LCT) Family |
| 2.A.44.2 | | 1 | 1 | 1 | 1 | 1 | 1 | 6 | The Formate-Nitrite Transporter (FNT) Family |
| 2.A.47.2 | **1** | 1 | 1 | 2 | 2 | 2 | 3 | 12 | The Divalent Anion: Na+ Symporter (DASS) Family |
| 2.A.49.1 | **1** | | 1 | | | | | 2 | The Chloride Carrier/Channel (ClC) Family |
| 2.A.49.2 | **2** | | 1 | | | | | 3 | The Chloride Carrier/Channel (ClC) Family |
| 2.A.49.3 | | 5 | | 3 | 3 | 3 | 3 | 17 | The Chloride Carrier/Channel (ClC) Family |
| 2.A.5.1 | **4** | 4 | 2 | 3 | 1 | 2 | 2 | 18 | The Zinc (Zn2+)-Iron (Fe2+) Permease (ZIP) Family |
| 2.A.5.2 | | | | | 1 | 1 | 1 | 3 | The Zinc (Zn2+)-Iron (Fe2+) Permease (ZIP) Family |
| 2.A.5.4 | **1** | | 1 | | | | | 2 | The Zinc (Zn2+)-Iron (Fe2+) Permease (ZIP) Family |
| 2.A.5.5 | **1** | | 1 | | | | | 2 | The Zinc (Zn2+)-Iron (Fe2+) Permease (ZIP) Family |
| 2.A.5.7 | | | 1 | | | | | 1 | The Zinc (Zn2+)-Iron (Fe2+) Permease (ZIP) Family |
| 2.A.5.Y1 | | | | | 1 | | 1 | 2 | The Zinc (Zn2+)-Iron (Fe2+) Permease (ZIP) Family |
| 2.A.5.Y2 | | 1 | | 1 | | | | 2 | The Zinc (Zn2+)-Iron (Fe2+) Permease (ZIP) Family |
| 2.A.50.1 | **1** | 1 | 1 | 1 | 1 | 1 | 2 | 8 | The Glycerol Uptake (GUP) Family |
| 2.A.53.1 | **2** | 2 | 2 | 3 | 2 | 2 | 3 | 16 | The Sulfate Permease (SulP) Family |
| 2.A.53.7 | **1** | | 1 | | | | | 2 | The Sulfate Permease (SulP) Family |
| 2.A.53.Y1 | | 1 | | 1 | 1 | 1 | 1 | 5 | The Sulfate Permease (SulP) Family |
| 2.A.54.1 | **1** | 1 | 1 | 1 | 1 | | 1 | 6 | The Mitochondrial Tricarboxylate Carrier (MTC) Family |
| 2.A.55.1 | **2** | 2 | 3 | 3 | 3 | 3 | 3 | 19 | The Metal Ion (Mn2+-iron) Transporter (Nramp) Family |
| 2.A.57.3 | **1** | 1 | 1 | 1 | 1 | 1 | 1 | 7 | The Equilibrative Nucleoside Transporter (ENT) Family |
| 2.A.59.1 | **1** | 1 | 1 | 1 | 2 | | 1 | 7 | The Arsenical Resistance-3 (ACR3) Family |
| 2.A.6.6 | **3** | 2 | 3 | 2 | 2 | 2 | 3 | 17 | The Eukaryotic (Putative) Sterol Transporter (EST) Family |
| 2.A.6.Y1 | | 1 | | | | | | 1 | Unassigned |
| 2.A.66.1 | **2** | 2 | 2 | 6 | 4 | 2 | 2 | 20 | The Multi Antimicrobial Extrusion (MATE) Family |
| 2.A.66.3 | **1** | 1 | 1 | | 1 | 1 | 1 | 6 | The Oligosaccharidyl-lipid Flippase (OLF) Family |
| 2.A.67.1 | **6** | 17 | 2 | 4 | 3 | | 2 | 34 | The Oligopeptide Transporter (OPT) Family |
| 2.A.67.2 | **1** | | 1 | | | | | 2 | The Oligopeptide Transporter (OPT) Family |
| 2.A.67.Y1 | | 1 | | 1 | 1 | 1 | 1 | 5 | The Oligopeptide Transporter (OPT) Family |
| 2.A.69.3 | | | 2 | | | | | 2 | The Auxin Efflux Carrier (AEC) Family |
| 2.A.7.10 | **1** | 1 | 1 | | 2 | | 1 | 6 | The UDP-N-Acetylglucosamine:UMP Antiporter (UAA) Family |
| 2.A.7.11 | **1** | | 1 | | | | | 2 | The UDP-Galactose:UMP Antiporter (UGA) Family |
| 2.A.7.12 | **1** | 1 | 1 | | | | | 3 | The CMP-Sialate:CMP Antiporter (CSA) Family |
| 2.A.7.13 | **1** | 1 | 1 | 1 | 1 | 1 | 3 | 9 | The GDP-Mannose:GMP Antiporter (GMA) Family |
| 2.A.7.24 | **3** | | 3 | | | | | 6 | The Thiamine Pyrophosphate Transporter (TPPT) Family |
| 2.A.7.25 | **2** | | 2 | | | | | 4 | The NIPA Mg2+ Uptake Permease (NIPA) Family |
| 2.A.7.3 | | | 1 | | | | | 1 | The 10 TMS Drug/Metabolite Exporter (DME) Family |
| 2.A.7.9 | **1** | 2 | 1 | 3 | 3 | 3 | 3 | 16 | The Triose-phosphate Transporter (TPT) Family |

| | | | | | | | | | | |
|---|---|---|---|---|---|---|---|---|---|---|
| | 2.A.7.Y1 | | 1 | | 1 | 1 | 1 | 1 | 5 | Unassigned |
| | 2.A.7.Y2 | | 1 | | | | | | 1 | Unassigned |
| | 2.A.72.2 | | 2 | | | | | | 2 | The K+ Uptake Permease (KUP) Family |
| | 2.A.85.3 | **4** | | 1 | | | | | 5 | The Aromatic Acid Exporter (ArAE) Family |
| | 2.A.85.4 | **1** | | | | | | | 1 | The Aromatic Acid Exporter (ArAE) Family |
| | 2.A.89.1 | **1** | | | | | | | 1 | The Vacuolar Iron Transporter (VIT) Family |
| | 2.A.9.1 | **1** | 1 | | 1 | 1 | 1 | 1 | 6 | The Cytochrome Oxidase Biogenesis (Oxa1) Family |
| | 2.A.92.1 | **1** | | 1 | | | | | 2 | The Choline Transporter-like (CTL) Family |
| | 2.A.93.1 | | | 1 | | | | | 1 | The Unknown BART Superfamily-1 (UBS1) Family |
| | 2.A.94.1 | **4** | | 2 | | | | | 6 | The Phosphate Permease (Pho1) Family |
| | 2.A.96.1 | **5** | | 5 | | | | | 10 | The YaaH (YaaH) Family |
| | 2.A.X1.Y1 | | 2 | | | 2 | 1 | | 5 | Unassigned |
| | 2.A.X2.Y1 | | 1 | | 2 | 1 | | | 4 | Unassigned |
| Primary Active Transporters | 3.A.1.201 | **1** | 3 | 1 | | | | | 5 | The Multidrug Resistance Exporter (MDR) Family (ABCB) |
| | 3.A.1.203 | **2** | 2 | | 2 | 2 | 2 | 2 | 12 | The Peroxysomal Fatty Acyl CoA Transporter (P-FAT) Family (ABCD) |
| | 3.A.1.204 | **3** | 2 | 1 | 1 | 1 | 1 | 1 | 10 | The Eye Pigment Precursor Transporter (EPP) Family (ABCG |
| | 3.A.1.205 | **5** | 6 | 6 | 8 | 5 | 6 | 9 | 45 | The Pleiotropic Drug Resistance (PDR) Family (ABCG) |
| | 3.A.1.206 | **1** | 1 | 1 | 1 | 1 | 1 | 1 | 7 | The a-Factor Sex Pheromone Exporter (STE) Family (ABCB) |
| | 3.A.1.208 | **4** | 10 | 5 | 4 | 4 | 5 | 7 | 39 | The Drug Conjugate Transporter (DCT) Family (ABCC) |
| | 3.A.1.210 | **1** | 1 | 1 | 1 | 1 | 1 | 1 | 7 | The Heavy Metal Transporter (HMT) Family |
| | 3.A.1.211 | **1** | 1 | | | | | | 2 | The Cholesterol/Phospholipid/Retinal (CPR) Flippase Family |
| | 3.A.1.212 | **1** | 1 | 1 | 2 | 2 | 2 | 2 | 11 | The Mitochondrial Peptide Exporter (MPE) Family |
| | 3.A.12.1 | **1** | | | | | | | 1 | The Septal DNA Translocator (S-DNA-T) Family |
| | 3.A.16.1 | **2** | | 1 | | | | | 3 | The Endoplasmic Reticular Retrotranslocon (ER-RT) Family |
| | 3.A.18.1 | **3** | | 1 | | | | | 4 | The Nuclear mRNA Exporter (mRNA-E) Family |
| | 3.A.2.1 | | 7 | | 8 | 10 | 8 | 12 | 45 | The H+- or Na+-translocating F-type Family |
| | 3.A.2.2 | **5** | 14 | 5 | 15 | 15 | 15 | 16 | 85 | The H+- or Na+-translocating F-type Family |
| | 3.A.3.1 | **1** | | | | | | | 1 | The Na+, K+ ATPase Family |
| | 3.A.3.10 | **1** | | 1 | | | | | 2 | Functionally Uncharacterized P-type ATPase Family |
| | 3.A.3.14 | | | 1 | | | | | 1 | Functionally Uncharacterized P-type ATPase Family |
| | 3.A.3.15 | **1** | | | | | | | 1 | Functionally Uncharacterized P-type ATPase Family |
| | 3.A.3.2 | **3** | 2 | 2 | 2 | 2 | 3 | 2 | 16 | The Ca2+-ATPase Family |
| | 3.A.3.3 | **2** | 1 | 1 | 1 | 1 | 1 | 2 | 9 | Unassigned |
| | 3.A.3.5 | **2** | 2 | 2 | 2 | 2 | 1 | 2 | 13 | The Cu+-, Ag+-ATPase (efflux) Family |
| | 3.A.3.8 | **5** | 4 | 5 | 4 | 4 | 5 | 5 | 32 | The Phospholipid Translocating P-type ATPase Family |
| | 3.A.3.9 | **1** | 2 | 1 | 2 | 2 | 1 | 3 | 12 | The Monovalent Alkali Cation (Na+ and K+) ATPase (efflux) Family |
| | 3.A.3.Y1 | | 2 | | 3 | 2 | 2 | 2 | 11 | Unassigned |
| | 3.A.5.8 | **4** | 9 | 2 | 9 | 12 | 9 | 11 | 56 | The General Secretory Pathway (Sec) Family |
| | 3.A.5.9 | **1** | | | | | | | 1 | The General Secretory Pathway (Sec) Family |
| | 3.A.8.1 | **1** | 17 | | 13 | 13 | 14 | 14 | 72 | The Mitochondrial Protein Translocase (MPT) Family |
| | 3.A.8.2 | | 7 | | 11 | 12 | 10 | 15 | 55 | The Mitochondrial Protein Translocase (MPT) Family |
| Decarboxylation-driven transporters | 3.B.1.1 | | 2 | | 1 | 1 | 2 | | | The Na+-transporting Carboxylic Acid Decarboxylase (NaT-DC) Family |
| | | | | | | | | | 6 | |

| | | | | | | | | | | |
|---|---|---|---|---|---|---|---|---|---|---|
| Oxidoreduction-driven transporters | 3.D.1.2 | | 8 | | 8 | | | | 16 | The H+ or Na+-translocating NADH Dehydrogenase (NDH) Family |
| | 3.D.1.6 | **1** | | | | | | | 1 | The H+ or Na+-translocating NADH Dehydrogenase (NDH) Family |
| | 3.D.3.3 | | 6 | | 9 | 8 | 8 | 10 | 41 | The Proton-translocating Quinol:Cytochrome c Reductase (QCR) Family |
| | 3.D.4.4 | | | 1 | | | | | 1 | |
| | 3.D.4.8 | **2** | 7 | 1 | 10 | 10 | 7 | 15 | 52 | The Proton-translocating Cytochrome Oxidase (COX) Family |
| Light absorption-driven transporters | 3.E.1.4 | | | | | | 3 | 3 | 6 | The Ion-translocating Microbial Rhodopsin (MR) Family |
| | 3.E.1.5 | **1** | | | | | | | 1 | The Ion-translocating Microbial Rhodopsin (MR) Family |
| Transmembrane Electron Carriers | 5.B.1.4 | | | 1 | | | | | 1 | The Phagocyte (gp91phox) NADPH Oxidase Family |
| | 5.B.1.5 | **8** | | 1 | | | | | 9 | The Phagocyte (gp91phox) NADPH Oxidase Family |
| | 5.B.1.7 | **3** | | 4 | | | | | 7 | The Phagocyte (gp91phox) NADPH Oxidase Family |
| | 5.B.2.1 | **1** | | | | | | | 1 | The Eukaryotic Cytochrome b561 (Cytb561) Family |
| Accessory Factors Involved in Transport | 8.A.13.1 | | 1 | | 1 | 1 | 1 | 1 | 5 | The Tetratricopeptide Repeat (Tpr1) Family |
| | 8.A.15.Y1 | | 1 | | | | 1 | | 2 | The K+ Channel Accessory Protein (KChAP) Family |
| | 8.A.27.1 | **1** | | | | | | | 1 | The Phospholipid Importer β-subunit (PLI-β) Family |
| | 8.A.5.1 | | 2 | | 3 | 1 | | 1 | 7 | The Voltage-gated K+ Channel β-subunit (Kvβ) Family |
| | 8.A.6.1 | | 1 | | 1 | 1 | 1 | 1 | 5 | The Auxiliary Nutrient Transporter (ANT) Family |
| | 8.A.9.1 | | | | 2 | 2 | | | 4 | The rBAT Transport Accessory Protein (rBAT) Family |
| | 8.A.9.Y1 | | | | | | | 7 | 7 | The rBAT Transport Accessory Protein (rBAT) Family |
| Incompletely Characterized Transport Systems: Recognized transporters of unknown biochemical mechanism | 9.A.1.1 | | 1 | | 2 | 2 | 3 | 3 | 11 | The Non ABC Multidrug Exporter (N-MDE) Family |
| | 9.A.10.1 | **3** | 3 | 3 | 4 | 2 | 2 | 2 | 19 | The Oxidase-dependent Fe2+ Transporter (OFeT) Family |
| | 9.A.10.Y1 | | 3 | | 2 | 3 | 3 | 3 | 14 | Unassigned |
| | 9.A.12.1 | | | | | | 1 | 2 | 3 | The Peptidoglycolipid Addressing Protein (GAP) Family |
| | 9.A.12.2 | | | | | 1 | 1 | 1 | 3 | The Peptidoglycolipid Addressing Protein (GAP) Family |
| | 9.A.12.Y1 | | 1 | | | | | | 1 | The Peptidoglycolipid Addressing Protein (GAP) Family |
| | 9.A.12.Y2 | | | | 1 | | | | 1 | The Peptidoglycolipid Addressing Protein (GAP) Family |
| | 9.A.12.Y3 | | | | 1 | | | | 1 | The Peptidoglycolipid Addressing Protein (GAP) Family |
| | 9.A.12.Y4 | | | | 1 | | | | 1 | The Peptidoglycolipid Addressing Protein (GAP) Family |
| | 9.A.12.Y5 | | | | | 1 | | | 1 | The Peptidoglycolipid Addressing Protein (GAP) Family |
| | 9.A.14.1 | **3** | | 1 | | | | | 4 | The G-protein-coupled receptor (GPCR) Family |
| | 9.A.14.Y1 | | 21 | | 23 | 25 | 27 | 29 | 125 | The G-protein-coupled receptor (GPCR) Family |
| | 9.A.14.Y2 | | 1 | | 1 | 2 | 1 | 1 | 6 | The G-protein-coupled receptor (GPCR) Family |
| | 9.A.14.Y3 | | 13 | | 15 | 15 | 17 | 16 | 76 | The G-protein-coupled receptor (GPCR) Family |
| | 9.A.14.Y4 | | 4 | | 4 | 5 | 4 | 5 | 22 | The G-protein-coupled receptor (GPCR) Family |
| | 9.A.14.Y5 | | 6 | | 9 | 7 | 8 | 7 | 37 | The G-protein-coupled receptor (GPCR) Family |
| | 9.A.26.1 | **5** | | 2 | 4 | 1 | 1 | 4 | 17 | The Lipid-translocating Exporter (LTE) Family |
| | 9.A.26.2 | | 2 | | 1 | 1 | 1 | 1 | 6 | The Lipid-translocating Exporter (LTE) Family |
| | 9.A.27.1 | **1** | | 1 | | | | | 2 | The Non-Classical Protein Exporter (NCPE) Family |
| | 9.A.40.3 | **2** | | | | | | | 2 | The HlyC/CorC (HCC) Family |
| | 9.A.45.1 | **1** | | 1 | | | | | 2 | The Magnesium Transporter1 (MagT1) Family |
| | 9.A.46.1 | **2** | | 1 | | | | | 3 | The Putative Low Affinity Ca2+ Channel (LACC) Family |
| | 9.A.5.1 | | 27 | | 7 | 10 | 11 | 10 | 65 | The Putative Arginine Transporter (ArgW) Family |
| | 9.A.6.1 | **3** | | 2 | | | | | 5 | The ATP Exporter (ATP-E) Family |

| | | | | | | | | | | |
|---|---|---|---|---|---|---|---|---|---|---|
| | 9.A.9.1 | | | 2 | | 1 | 1 | 1 | 5 | The Low Affinity Fe2+ Transporter (FeT) Family |
| Incompletely characterized Transport Systems: Putative transport proteins | 9.B.1.1 | **1** | | | | 1 | 1 | 1 | 4 | The Metal Homeostasis Protein (MHP) Family |
| | 9.B.1.Y1 | | 1 | | 1 | | | | 2 | The Metal Homeostasis Protein (MHP) Family |
| | 9.B.12.1 | | | | 1 | | | 1 | 2 | The (Salt or Low Temperature) Stress-induced Hydrophobic Peptide Family |
| | 9.B.12.3 | **1** | 2 | | 3 | 1 | | 3 | 10 | The (Salt or Low Temperature) Stress-induced Hydrophobic Peptide Family |
| | 9.B.17.1 | | 14 | | 3 | 3 | 5 | 5 | 30 | The VAMP-associated protein (VAP) Family |
| | 9.B.17.Y1 | | 1 | | 4 | 2 | 3 | 4 | 14 | The VAMP-associated protein (VAP) Family |
| | 9.B.17.Y2 | | 1 | | 2 | | | | 3 | The VAMP-associated protein (VAP) Family |
| | 9.B.19.1 | **1** | | | | | | | 1 | The Mn2+ Homeostasis Protein (MnHP) Family |
| | 9.B.2.1 | | | | | | 1 | 1 | 2 | The Ca2+ Homeostasis Protein (CHP) Family |
| | 9.B.2.Y1 | | | | 1 | | | | 1 | The Ca2+ Homeostasis Protein (CHP) Family |
| | 9.B.24.3 | **1** | | 1 | | | | | 2 | The Outer Membrane Protein NilB/OmpU (NilB) Family |
| | 9.B.24.Y1 | | | | | 1 | 1 | 1 | 3 | The Outer Membrane Protein NilB/OmpU (NilB) Family |
| | 9.B.24.Y2 | | 1 | | | | | | 1 | The Outer Membrane Protein NilB/OmpU (NilB) Family |
| | 9.B.24.Y3 | | | | 1 | | | | 1 | The Outer Membrane Protein NilB/OmpU (NilB) Family |
| | 9.B.26.3 | **1** | | 1 | | | | | 2 | The PF27 (PF27) Family |
| | 9.B.26.Y1 | | | | 1 | 1 | 1 | 1 | 4 | The PF27 (PF27) Family |
| | 9.B.26.Y2 | | 1 | | | | | | 1 | The PF27 (PF27) Family |
| | 9.B.32.1 | **4** | | 1 | | | | | 5 | The Putative Vectorial Glycosyl Polymerization (VGP) Family |
| | 9.B.33.1 | | 6 | | 7 | 4 | 3 | 3 | 23 | The Sensor Histidine Kinase (SHK) Family |
| | 9.B.37.1 | **2** | | 1 | | | | | 3 | The Huntington-interacting Protein 14 (HIP14) Family |
| | 9.B.37.2 | **3** | | 3 | | | | | 6 | The Huntington-interacting Protein 14 (HIP14) Family |
| | 9.B.37.3 | | | 1 | | | | | 1 | The Huntington-interacting Protein 14 (HIP14) Family |
| | 9.B.4.Y1 | | | | 1 | | | | 1 | The Universal Stress Protein-B (UspB) Family |
| | 9.B.4.Y2 | | 1 | | | 1 | 1 | | 3 | The Universal Stress Protein-B (UspB) Family |
| | 9.B.4.Y3 | | 2 | | | 1 | 1 | | 4 | The Universal Stress Protein-B (UspB) Family |
| | 9.B.7.2 | **1** | | 1 | | | | | 2 | The Putative Sulfate Transporter (CysZ) Family |
| | 9.B.82.1 | **1** | | | | | | | 1 | Putative Heavy Metal Transporter (Rer1) Family |
| | Total | **430** | 597 | 253 | 538 | 439 | 398 | 508 | 3163 | |

# Table S10B TCDB classification of transporters of *A. adeninivorans*

| Gene ARAD1 | Nb TMH | TCDB Best Hit | TCDB class | TCDB Annotation of best hit |
|---|---|---|---|---|
| ARAD1C38192g | 4 | gnl\|TC-DB\|P35499 | 1.A.1.10 | Sodium channel protein type 4 subunit alpha - *Homo sapiens* (Human). |
| ARAD1D08602g | 21 | gnl\|TC-DB\|P50077 | 1.A.1.11 | Calcium-channel protein CCH1 - *Saccharomyces cerevisiae* (Baker's yeast). |
| ARAD1D15510g | 3 | gnl\|TC-DB\|O54899 | 1.A.1.5 | Brain cyclic nucleotide gated 1 - *mus musculus* (mouse). |
| ARAD1D15488g | 10 | gnl\|TC-DB\|P40310 | 1.A.1.7 | Outward-rectifier potassium channel Tok1 - *Saccharomyces cerevisiae* (baker's yeast). |
| ARAD1D14960g | 11 | gnl\|TC-DB\|P40260 | 1.A.11.3 | Ammonium transporter mep1 - *Saccharomyces cerevisiae* (baker's yeast). |
| ARAD1D31020g | 11 | gnl\|TC-DB\|Q59UP8 | 1.A.11.3 | Putative uncharacterized protein MEP2 - *Candida albicans* |
| ARAD1C40876g | 8 | gnl\|TC-DB\|D0NGF4 | 1.A.17.2 | Anoctamin-like protein - *Phytophthora infestans* |
| ARAD1D40766g | 2 | gnl\|TC-DB\|Q08269 | 1.A.35.2 | Aluminum resistance protein 1 - *Saccharomyces cerevisiae* (Baker's yeast). |
| ARAD1D30888g | 3 | gnl\|TC-DB\|P35724 | 1.A.35.2 | Manganese resistance protein - *Saccharomyces cerevisiae* (baker's yeast). |
| ARAD1A11616g | 2 | gnl\|TC-DB\|Q01926 | 1.A.35.5 | Mitochondrial RNA splicing protein Mrs2 precursor - *Saccharomyces cerevisiae* |
| ARAD1D49830g | 8 | gnl\|TC-DB\|B2ZXD5 | 1.A.38.1 | Golgi pH regulator - Cricetulus griseus |
| ARAD1B20526g | 7 | gnl\|TC-DB\|Q12324 | 1.A.4.4 | Calcium channel YVC1 - *Saccharomyces cerevisiae* (Baker's yeast). |
| ARAD1A02750g | 8 | gnl\|TC-DB\|Q12324 | 1.A.4.4 | Calcium channel YVC1 - *Saccharomyces cerevisiae* (Baker's yeast). |
| ARAD1C07194g | 6 | gnl\|TC-DB\|Q12324 | 1.A.4.4 | Calcium channel YVC1 - *Saccharomyces cerevisiae* (Baker's yeast). |
| ARAD1B13992g | 3 | gnl\|TC-DB\|B8N1Q6 | 1.A.55.4 | Clathrin-coated vesicle protein, putative - *Aspergillus flavus* strain ATCC 200026 |
| ARAD1D05984g | 3 | gnl\|TC-DB\|O94722 | 1.A.56.1 | Copper transport protein ctr4 (Copper transporter 4) - *Schizosaccharomyces pombe* |
| ARAD1C44748g | 3 | gnl\|TC-DB\|A9XIK8 | 1.A.56.1 | CTR2 long splice variant- *Colletotrichum gloeosporioides f. sp. aeschynomenes* |
| ARAD1D01562g | 7 | gnl\|TC-DB\|Q9C411 | 1.A.8.6 | Aquaporin 2 - *Saccharomyces chevalieri.* |
| ARAD1C31372g | 2 | gnl\|TC-DB\|B3FNS7 | 1.B.40.1 | Trimeric autotransporter adhesin - *Haemophilus sp.* |
| ARAD1C24640g | 4 | gnl\|TC-DB\|B3FNS7 | 1.B.40.1 | Trimeric autotransporter adhesin - *Haemophilus sp.* 1595 |
| ARAD1B05038g | 3 | gnl\|TC-DB\|P69169 | 1.B.53.1 | Coat protein A - *Enterobacteria* phage f1 |
| ARAD1D16676g | 8 | gnl\|TC-DB\|Q14770 | 2.A.1.1 | Fructose transporter - *Homo sapiens* (human). |
| ARAD1C12694g | 5 | gnl\|TC-DB\|P53048 | 2.A.1.1 | General alpha-glucoside permease - *Saccharomyces cerevisiae* (Baker's yeast). |
| ARAD1D18106g | 10 | gnl\|TC-DB\|P53048 | 2.A.1.1 | General alpha-glucoside permease - *Saccharomyces cerevisiae* (Baker's yeast). |
| ARAD1D18062g | 12 | gnl\|TC-DB\|P53048 | 2.A.1.1 | General alpha-glucoside permease - *Saccharomyces cerevisiae* (Baker's yeast). |
| ARAD1C05104g | 8 | gnl\|TC-DB\|P53048 | 2.A.1.1 | General alpha-glucoside permease - *Saccharomyces cerevisiae* (Baker's yeast). |
| ARAD1D51040g | 12 | gnl\|TC-DB\|P53048 | 2.A.1.1 | General alpha-glucoside permease - *Saccharomyces cerevisiae* (Baker's yeast). |
| ARAD1D42680g | 11 | gnl\|TC-DB\|Q5A8J5 | 2.A.1.1 | Glycerol/H+ symportertransporter STL1 - *Candida albicans* |
| ARAD1A14102g | 12 | gnl\|TC-DB\|Q5A8J5 | 2.A.1.1 | Glycerol/H+ symportertransporter STL1 - *Candida albicans* |
| ARAD1A19954g | 10 | gnl\|TC-DB\|Q5A8J5 | 2.A.1.1 | Glycerol/H+ symportertransporter STL1 - *Candida albicans* |
| ARAD1B17908g | 12 | gnl\|TC-DB\|Q5A8J5 | 2.A.1.1 | Glycerol/H+ symportertransporter STL1 - *Candida albicans* |
| ARAD1D26840g | 10 | gnl\|TC-DB\|Q5A8J5 | 2.A.1.1 | Glycerol/H+ symportertransporter STL1 - *Candida albicans* |
| ARAD1A06754g | 11 | gnl\|TC-DB\|P39932 | 2.A.1.1 | Glycerol/H+ symportertransporter STL1 - *Saccharomyces cerevisiae* (Baker's yeast). |
| ARAD1A08778g | 12 | gnl\|TC-DB\|P39932 | 2.A.1.1 | Glycerol/H+ symportertransporter STL1 - *Saccharomyces cerevisiae* (Baker's yeast). |
| ARAD1B10296g | 10 | gnl\|TC-DB\|P39932 | 2.A.1.1 | Glycerol/H+ symportertransporter STL1 - *Saccharomyces cerevisiae* (Baker's yeast). |

| | | | | |
|---|---|---|---|---|
| ARAD1C18216g | 10 | gnl\|TC-DB\|P39932 | 2.A.1.1 | Glycerol/H+ symportertransporter STL1 - *Saccharomyces cerevisiae* (Baker's yeast). |
| ARAD1D03476g | 9 | gnl\|TC-DB\|P39932 | 2.A.1.1 | Glycerol/H+ symportertransporter STL1 - *Saccharomyces cerevisiae* (Baker's yeast). |
| ARAD1D18084g | 12 | gnl\|TC-DB\|P39932 | 2.A.1.1 | Glycerol/H+ symportertransporter STL1 - *Saccharomyces cerevisiae* (Baker's yeast). |
| ARAD1D18634g | 12 | gnl\|TC-DB\|P39932 | 2.A.1.1 | Glycerol/H+ symportertransporter STL1 - *Saccharomyces cerevisiae* (Baker's yeast). |
| ARAD1D29634g | 10 | gnl\|TC-DB\|P39932 | 2.A.1.1 | Glycerol/H+ symportertransporter STL1 - *Saccharomyces cerevisiae* (Baker's yeast). |
| ARAD1D41998g | 12 | gnl\|TC-DB\|P39932 | 2.A.1.1 | Glycerol/H+ symportertransporter STL1 - *Saccharomyces cerevisiae* (Baker's yeast). |
| ARAD1D50710g | 10 | gnl\|TC-DB\|P39932 | 2.A.1.1 | Glycerol/H+ symportertransporter STL1 - *Saccharomyces cerevisiae* (Baker's yeast). |
| ARAD1B01298g | 12 | gnl\|TC-DB\|Q8NJ22 | 2.A.1.1 | Hexose transporter (Similarity) - *Kluyveromyces lactis* (Yeast). |
| ARAD1B17622g | 10 | gnl\|TC-DB\|Q8NJ22 | 2.A.1.1 | Hexose transporter (Similarity) - *Kluyveromyces lactis* (Yeast). |
| ARAD1D05016g | 10 | gnl\|TC-DB\|Q8NJ22 | 2.A.1.1 | Hexose transporter (Similarity) - *Kluyveromyces lactis* (Yeast). |
| ARAD1B23166g | 11 | gnl\|TC-DB\|P43581 | 2.A.1.1 | Hexose transporter HXT10 - *Saccharomyces cerevisiae* (Baker's yeast). |
| ARAD1B12496g | 12 | gnl\|TC-DB\|P49374 | 2.A.1.1 | High-affinity glucose transporter - *Kluyveromyces lactis* (Yeast) (*Candida sphaerica*). |
| ARAD1B12518g | 11 | gnl\|TC-DB\|P49374 | 2.A.1.1 | High-affinity glucose transporter - *Kluyveromyces lactis* (Yeast) (*Candida sphaerica*). |
| ARAD1D10362g | 10 | gnl\|TC-DB\|P49374 | 2.A.1.1 | High-affinity glucose transporter - *Kluyveromyces lactis* (Yeast) (*Candida sphaerica*). |
| ARAD1B09900g | 8 | gnl\|TC-DB\|P49374 | 2.A.1.1 | High-affinity glucose transporter - *Kluyveromyces lactis* (Yeast) (*Candida sphaerica*). |
| ARAD1A15246g | 10 | gnl\|TC-DB\|P49374 | 2.A.1.1 | High-affinity glucose transporter - *Kluyveromyces lactis* (Yeast) (*Candida sphaerica*). |
| ARAD1D50952g | 8 | gnl\|TC-DB\|P39003 | 2.A.1.1 | High-affinity hexose transporter HXT6 - *Saccharomyces cerevisiae* (Baker's yeast). |
| ARAD1C00242g | 11 | gnl\|TC-DB\|P07921 | 2.A.1.1 | Lactose permease - *Kluyveromyces lactis* (Yeast). |
| ARAD1D12012g | 12 | gnl\|TC-DB\|P07921 | 2.A.1.1 | Lactose permease - *Kluyveromyces lactis* (Yeast). |
| ARAD1A11792g | 11 | gnl\|TC-DB\|P07921 | 2.A.1.1 | Lactose permease - *Kluyveromyces lactis* (Yeast). |
| ARAD1B20438g | 10 | gnl\|TC-DB\|P07921 | 2.A.1.1 | Lactose permease - *Kluyveromyces lactis* (Yeast). |
| ARAD1C00264g | 12 | gnl\|TC-DB\|P15685 | 2.A.1.1 | Maltose permease MAL6T - *Saccharomyces cerevisiae* (Baker's yeast). |
| ARAD1D35728g | 12 | gnl\|TC-DB\|P15685 | 2.A.1.1 | Maltose permease MAL6T - *Saccharomyces cerevisiae* (Baker's yeast). |
| ARAD1A07150g | 11 | gnl\|TC-DB\|P15685 | 2.A.1.1 | Maltose permease MAL6T - *Saccharomyces cerevisiae* (Baker's yeast). |
| ARAD1D09658g | 12 | gnl\|TC-DB\|P15685 | 2.A.1.1 | Maltose permease MAL6T - *Saccharomyces cerevisiae* (Baker's yeast). |
| ARAD1A09746g | 12 | gnl\|TC-DB\|P15685 | 2.A.1.1 | Maltose permease MAL6T - *Saccharomyces cerevisiae* (Baker's yeast). |
| ARAD1C20878g | 10 | gnl\|TC-DB\|Q8J0V1 | 2.A.1.1 | Monosaccharide transporter - *Aspergillus niger.* |
| ARAD1C00396g | 10 | gnl\|TC-DB\|Q8J0V1 | 2.A.1.1 | Monosaccharide transporter - *Aspergillus niger.* |
| ARAD1A00572g | 11 | gnl\|TC-DB\|A0ZXK6 | 2.A.1.1 | Monosaccharide transporter - *Geosiphon pyriformis.* |
| ARAD1D13442g | 12 | gnl\|TC-DB\|P30605 | 2.A.1.1 | Myo-inositol transporter 1 - *Saccharomyces cerevisiae* (Baker's yeast). |
| ARAD1C40392g | 12 | gnl\|TC-DB\|P30605 | 2.A.1.1 | Myo-inositol transporter 1 - *Saccharomyces cerevisiae* (Baker's yeast). |
| ARAD1C40678g | 12 | gnl\|TC-DB\|P30605 | 2.A.1.1 | Myo-inositol transporter 1 - *Saccharomyces cerevisiae* (Baker's yeast). |
| ARAD1C45012g | 12 | gnl\|TC-DB\|P11636 | 2.A.1.1 | Quinate permease (Quinate transporter) - *Neurospora crassa*. |
| ARAD1D50622g | 12 | gnl\|TC-DB\|P11636 | 2.A.1.1 | Quinate permease (Quinate transporter) - *Neurospora crassa*. |
| ARAD1C34342g | 11 | gnl\|TC-DB\|P11636 | 2.A.1.1 | Quinate permease (Quinate transporter) - *Neurospora crassa*. |
| ARAD1C40326g | 12 | gnl\|TC-DB\|P11636 | 2.A.1.1 | Quinate permease (Quinate transporter) - *Neurospora crassa*. |
| ARAD1C05456g | 12 | gnl\|TC-DB\|P95908 | 2.A.1.1 | Sugar transporter (sugar transport related protein) - *Sulfolobus solfataricus*. |
| ARAD1B10384g | 12 | gnl\|TC-DB\|P95908 | 2.A.1.1 | Sugar transporter (sugar transport related protein) - *Sulfolobus solfataricus*. |
| ARAD1C25168g | 11 | gnl\|TC-DB\|A1Z264 | 2.A.1.1 | Sugar/H+ symporter - *Galdieria sulphuraria* GN=SPT1 PE=2 SV=1 |

| | | | | |
|---|---|---|---|---|
| ARAD1C40766g | 11 | gnl\|TC-DB\|A1Z264 | 2.A.1.1 | Sugar/H+ symporter - *Galdieria sulphuraria* GN=SPT1 PE=2 SV=2 |
| ARAD1C00440g | 12 | gnl\|TC-DB\|Q64L87 | 2.A.1.1 | Xylose facilitator Xylhp (Fragment) - *Debaryomyces hansenii* (Yeast) *(Torulaspora hansenii* ). |
| ARAD1C39600g | 11 | gnl\|TC-DB\|Q64L87 | 2.A.1.1 | Xylose facilitator Xylhp (Fragment) - *Debaryomyces hansenii* (Yeast) *(Torulaspora hansenii* ). |
| ARAD1C39094g | 12 | gnl\|TC-DB\|Q64L87 | 2.A.1.1 | Xylose facilitator Xylhp (Fragment) - *Debaryomyces hansenii* (Yeast) *(Torulaspora hansenii* ). |
| ARAD1D18194g | 11 | gnl\|TC-DB\|Q64L87 | 2.A.1.1 | Xylose facilitator Xylhp (Fragment) - *Debaryomyces hansenii* (Yeast) *(Torulaspora hansenii* ). |
| ARAD1C00220g | 12 | gnl\|TC-DB\|Q64L87 | 2.A.1.1 | Xylose facilitator Xylhp (Fragment) - *Debaryomyces hansenii* (Yeast) *(Torulaspora hansenii* ). |
| ARAD1D10120g | 12 | gnl\|TC-DB\|Q64L87 | 2.A.1.1 | Xylose facilitator Xylhp (Fragment) - *Debaryomyces hansenii* (Yeast) *(Torulaspora hansenii* ). |
| ARAD1C29898g | 11 | gnl\|TC-DB\|Q51330 | 2.A.1.11 | Oxalate:formate antiport protein - *Oxalobacter formigenes* . |
| ARAD1D14168g | 11 | gnl\|TC-DB\|P36035 | 2.A.1.12 | Carboxylic acid transporter protein homolog - *Saccharomyces cerevisiae* (Baker's yeast). |
| ARAD1D34804g | 10 | gnl\|TC-DB\|P36035 | 2.A.1.12 | Carboxylic acid transporter protein homolog - *Saccharomyces cerevisiae* (Baker's yeast). |
| ARAD1D47432g | 10 | gnl\|TC-DB\|P36035 | 2.A.1.12 | Carboxylic acid transporter protein homolog - *Saccharomyces cerevisiae* (Baker's yeast). |
| ARAD1C08008g | 11 | gnl\|TC-DB\|P36035 | 2.A.1.12 | Carboxylic acid transporter protein homolog - *Saccharomyces cerevisiae* (Baker's yeast). |
| ARAD1B13464g | 12 | gnl\|TC-DB\|Q08777 | 2.A.1.13 | Putative monocarboxylate permease YOR306c - *Saccharomyces cerevisiae* (Baker's yeast). |
| ARAD1D17842g | 12 | gnl\|TC-DB\|Q08777 | 2.A.1.13 | Putative monocarboxylate permease YOR306c - *Saccharomyces cerevisiae* (Baker's yeast). |
| ARAD1D27170g | 12 | gnl\|TC-DB\|Q08777 | 2.A.1.13 | Putative monocarboxylate permease YOR306c - *Saccharomyces cerevisiae* (Baker's yeast). |
| ARAD1C10824g | 12 | gnl\|TC-DB\|Q08777 | 2.A.1.13 | Putative monocarboxylate permease YOR306c - *Saccharomyces cerevisiae* (Baker's yeast). |
| ARAD1C14718g | 11 | gnl\|TC-DB\|Q08777 | 2.A.1.13 | Putative monocarboxylate permease YOR306c - *Saccharomyces cerevisiae* (Baker's yeast). |
| ARAD1D17292g | 11 | gnl\|TC-DB\|Q08777 | 2.A.1.13 | Putative monocarboxylate permease YOR306c - *Saccharomyces cerevisiae* (Baker's yeast). |
| ARAD1D36124g | 12 | gnl\|TC-DB\|Q08777 | 2.A.1.13 | Putative monocarboxylate permease YOR306c - *Saccharomyces cerevisiae* (Baker's yeast). |
| ARAD1D49390g | 12 | gnl\|TC-DB\|Q08777 | 2.A.1.13 | Putative monocarboxylate permease YOR306c - *Saccharomyces cerevisiae* (Baker's yeast). |
| ARAD1C00176g | 9 | gnl\|TC-DB\|P15365 | 2.A.1.14 | Allantoate permease - *Saccharomyces cerevisiae* (Baker's yeast). |
| ARAD1A13354g | 12 | gnl\|TC-DB\|P15365 | 2.A.1.14 | Allantoate permease - *Saccharomyces cerevisiae* (Baker's yeast). |
| ARAD1A19030g | 12 | gnl\|TC-DB\|P15365 | 2.A.1.14 | Allantoate permease - *Saccharomyces cerevisiae* (Baker's yeast). |
| ARAD1C20218g | 10 | gnl\|TC-DB\|P15365 | 2.A.1.14 | Allantoate permease - *Saccharomyces cerevisiae* (Baker's yeast). |
| ARAD1B22616g | 10 | gnl\|TC-DB\|P15365 | 2.A.1.14 | Allantoate permease - *Saccharomyces cerevisiae* (Baker's yeast). |
| ARAD1C11858g | 12 | gnl\|TC-DB\|P15365 | 2.A.1.14 | Allantoate permease - *Saccharomyces cerevisiae* (Baker's yeast). |
| ARAD1A16742g | 11 | gnl\|TC-DB\|P53322 | 2.A.1.14 | High-affinity nicotinic acid transporter - *Saccharomyces cerevisiae* (Baker's yeast). |
| ARAD1C12562g | 10 | gnl\|TC-DB\|P53322 | 2.A.1.14 | High-affinity nicotinic acid transporter - *Saccharomyces cerevisiae* (Baker's yeast). |
| ARAD1C20174g | 12 | gnl\|TC-DB\|P53322 | 2.A.1.14 | High-affinity nicotinic acid transporter - *Saccharomyces cerevisiae* (Baker's yeast). |
| ARAD1D30976g | 12 | gnl\|TC-DB\|P53322 | 2.A.1.14 | High-affinity nicotinic acid transporter - *Saccharomyces cerevisiae* (Baker's yeast). |
| ARAD1C04268g | 12 | gnl\|TC-DB\|P53322 | 2.A.1.14 | High-affinity nicotinic acid transporter - *Saccharomyces cerevisiae* (Baker's yeast). |
| ARAD1B08778g | 12 | gnl\|TC-DB\|P53322 | 2.A.1.14 | High-affinity nicotinic acid transporter - *Saccharomyces cerevisiae* (Baker's yeast). |
| ARAD1A14652g | 10 | gnl\|TC-DB\|O43000 | 2.A.1.14 | Pantothenate transporter liz1 - *Schizosaccharomyces pombe* (Fission yeast). |
| ARAD1C35992g | 7 | gnl\|TC-DB\|P70786 | 2.A.1.14 | Putative tartrate transporter - *Agrobacterium vitis* . |
| ARAD1B06138g | 8 | gnl\|TC-DB\|P70786 | 2.A.1.14 | Putative tartrate transporter - *Agrobacterium vitis* . |
| ARAD1D08558g | 12 | gnl\|TC-DB\|P70786 | 2.A.1.14 | Putative tartrate transporter - *Agrobacterium vitis* . |
| ARAD1A08228g | 12 | gnl\|TC-DB\|P53241 | 2.A.1.14 | Vitamin H transporter (H+/biotin symporter) - *Saccharomyces cerevisiae* (Baker's yeast). |
| ARAD1C26906g | 11 | gnl\|TC-DB\|O13880 | 2.A.1.14 | Vitamin H transporter 1 - *Schizosaccharomyces pombe* (Fission yeast). |
| ARAD1D50908g | 8 | gnl\|TC-DB\|O24842 | 2.A.1.15 | Hypothetical 47.9 |

| | | | | |
|---|---|---|---|---|
| ARAD1D48356g | 14 | gnl\|TC-DB\|Q08299 | 2.A.1.16 | Ferric enterobactin transporter YOL158c - *Saccharomyces cerevisiae* (baker's yeast). |
| ARAD1C11330g | 13 | gnl\|TC-DB\|P39980 | 2.A.1.16 | Ferrioxamine B transporter YEL065W - *Saccharomyces cerevisiae* |
| ARAD1D51018g | 13 | gnl\|TC-DB\|P39980 | 2.A.1.16 | Ferrioxamine B transporter YEL065W - *Saccharomyces cerevisiae* |
| ARAD1D11704g | 13 | gnl\|TC-DB\|P38731 | 2.A.1.16 | Transporter of the ARN family that specifically recognizes siderophore iron chelates |
| ARAD1A16852g | 14 | gnl\|TC-DB\|P38724 | 2.A.1.16 | Transporter of the ARN family that specifically recognizes siderophore iron chelates |
| ARAD1C34078g | 14 | gnl\|TC-DB\|P38724 | 2.A.1.16 | Transporter of the ARN family that specifically recognizes siderophore iron chelates |
| ARAD1C19514g | 11 | gnl\|TC-DB\|O35956 | 2.A.1.19 | Renal organic anion transport protein 1 - *Rattus norvegicus* (rat). |
| ARAD1D12672g | 11 | gnl\|TC-DB\|P28873 | 2.A.1.2 | Benomyl/methotrexate resistance protein - *Candida albicans* (Yeast). |
| ARAD1D12694g | 11 | gnl\|TC-DB\|P28873 | 2.A.1.2 | Benomyl/methotrexate resistance protein - *Candida albicans* (Yeast). |
| ARAD1D46156g | 12 | gnl\|TC-DB\|P32071 | 2.A.1.2 | Cycloheximide resistance protein - *Candida maltosa* (Yeast). |
| ARAD1D18546g | 12 | gnl\|TC-DB\|P32071 | 2.A.1.2 | Cycloheximide resistance protein - *Candida maltosa* (Yeast). |
| ARAD1D22176g | 12 | gnl\|TC-DB\|P32071 | 2.A.1.2 | Cycloheximide resistance protein - *Candida maltosa* (Yeast). |
| ARAD1D27918g | 10 | gnl\|TC-DB\|P32071 | 2.A.1.2 | Cycloheximide resistance protein - *Candida maltosa* (Yeast). |
| ARAD1C13860g | 12 | gnl\|TC-DB\|Q9KMQ3 | 2.A.1.2 | Multidrug resistance protein [*Vibrio cholerae*] |
| ARAD1C11352g | 12 | gnl\|TC-DB\|Q9KMQ3 | 2.A.1.2 | Multidrug resistance protein [*Vibrio cholerae*] |
| ARAD1A19800g | 12 | gnl\|TC-DB\|Q07824 | 2.A.1.2 | Polyamine transporter YLL028W - *Saccharomyces cerevisiae* (Baker's yeast). |
| ARAD1A03938g | 12 | gnl\|TC-DB\|Q07824 | 2.A.1.2 | Polyamine transporter YLL028W - *Saccharomyces cerevisiae* (Baker's yeast). |
| ARAD1D24970g | 12 | gnl\|TC-DB\|Q07824 | 2.A.1.2 | Polyamine transporter YLL028W - *Saccharomyces cerevisiae* (Baker's yeast). |
| ARAD1D27214g | 10 | gnl\|TC-DB\|Q07824 | 2.A.1.2 | Polyamine transporter YLL028W - *Saccharomyces cerevisiae* (Baker's yeast). |
| ARAD1B15224g | 12 | gnl\|TC-DB\|Q07824 | 2.A.1.2 | Polyamine transporter YLL028W - *Saccharomyces cerevisiae* (Baker's yeast). |
| ARAD1A19448g | 12 | gnl\|TC-DB\|Q07824 | 2.A.1.2 | Polyamine transporter YLL028W - *Saccharomyces cerevisiae* (Baker's yeast). |
| ARAD1B07260g | 10 | gnl\|TC-DB\|Q07824 | 2.A.1.2 | Polyamine transporter YLL028W - *Saccharomyces cerevisiae* (Baker's yeast). |
| ARAD1B15048g | 12 | gnl\|TC-DB\|Q07824 | 2.A.1.2 | Polyamine transporter YLL028W - *Saccharomyces cerevisiae* (Baker's yeast). |
| ARAD1D02772g | 12 | gnl\|TC-DB\|Q07824 | 2.A.1.2 | Polyamine transporter YLL028W - *Saccharomyces cerevisiae* (Baker's yeast). |
| ARAD1A19096g | 12 | gnl\|TC-DB\|Q07824 | 2.A.1.2 | Polyamine transporter YLL028W - *Saccharomyces cerevisiae* (Baker's yeast). |
| ARAD1D01100g | 12 | gnl\|TC-DB\|Q07824 | 2.A.1.2 | Polyamine transporter YLL028W - *Saccharomyces cerevisiae* (Baker's yeast). |
| ARAD1C32472g | 12 | gnl\|TC-DB\|Q07824 | 2.A.1.2 | Polyamine transporter YLL028W - *Saccharomyces cerevisiae* (Baker's yeast). |
| ARAD1C36806g | 11 | gnl\|TC-DB\|Q07824 | 2.A.1.2 | Polyamine transporter YLL028W - *Saccharomyces cerevisiae* (Baker's yeast). |
| ARAD1C37686g | 10 | gnl\|TC-DB\|Q07824 | 2.A.1.2 | Polyamine transporter YLL028W - *Saccharomyces cerevisiae* (Baker's yeast). |
| ARAD1C09394g | 10 | gnl\|TC-DB\|Q07824 | 2.A.1.2 | Polyamine transporter YLL028W - *Saccharomyces cerevisiae* (Baker's yeast). |
| ARAD1B01628g | 12 | gnl\|TC-DB\|P53389 | 2.A.1.2 | Protein HOL1 - *Saccharomyces cerevisiae* (Baker's yeast). |
| ARAD1D28314g | 10 | gnl\|TC-DB\|P53389 | 2.A.1.2 | Protein HOL1 - *Saccharomyces cerevisiae* (Baker's yeast). |
| ARAD1C17402g | 12 | gnl\|TC-DB\|P53389 | 2.A.1.2 | Protein HOL1 - *Saccharomyces cerevisiae* (Baker's yeast). |
| ARAD1D18524g | 9 | gnl\|TC-DB\|P53389 | 2.A.1.2 | Protein HOL1 - *Saccharomyces cerevisiae* (Baker's yeast). |
| ARAD1A15356g | 11 | gnl\|TC-DB\|P53389 | 2.A.1.2 | Protein HOL1 - *Saccharomyces cerevisiae* (Baker's yeast). |
| ARAD1D17336g | 12 | gnl\|TC-DB\|P53389 | 2.A.1.2 | Protein HOL1 - *Saccharomyces cerevisiae* (Baker's yeast). |
| ARAD1C17094g | 10 | gnl\|TC-DB\|P53389 | 2.A.1.2 | Protein HOL1 - *Saccharomyces cerevisiae* (Baker's yeast). |
| ARAD1D06226g | 12 | gnl\|TC-DB\|P53389 | 2.A.1.2 | Protein HOL1 - *Saccharomyces cerevisiae* (Baker's yeast). |
| ARAD1A18348g | 7 | gnl\|TC-DB\|P0A0J7 | 2.A.1.2 | Quinolone resistance protein norA - *Staphylococcus aureus* . |

| | | | | |
|---|---|---|---|---|
| ARAD1D31416g | 11 | gnl\|TC-DB\|Q5JAK9 | 2.A.1.2 | TetA(41) - *Serratia marcescens* GN=tetA(41) PE=4 SV=1 |
| ARAD1A13112g | 9 | gnl\|TC-DB\|B2YGG2 | 2.A.1.2 | Tetracycline resistance protein [*Micrococcus sp*. SMCC G887] |
| ARAD1C21626g | 11 | gnl\|TC-DB\|P02982 | 2.A.1.2 | Tetracycline resistance protein, class A (TETA(A)) - *Escherichia coli.* |
| ARAD1C33044g | 12 | gnl\|TC-DB\|P38227 | 2.A.1.2 | Uncharacterized transporter YBR043C - *Saccharomyces cerevisiae* |
| ARAD1A18260g | 12 | gnl\|TC-DB\|P38125 | 2.A.1.2 | Uncharacterized transporter YBR180W - *Saccharomyces cerevisiae* |
| ARAD1D36146g | 11 | gnl\|TC-DB\|P40474 | 2.A.1.2 | Uncharacterized transporter YIL121W - *Saccharomyces cerevisiae* (Baker's yeast). |
| ARAD1B13662g | 10 | gnl\|TC-DB\|P40474 | 2.A.1.2 | Uncharacterized transporter YIL121W - *Saccharomyces cerevisiae* (Baker's yeast). |
| ARAD1D02948g | 11 | gnl\|TC-DB\|Q02563 | 2.A.1.22 | Synaptic vesicle protein 2 (SV2) - *Rattus norvegicus* (Rat). |
| ARAD1C35530g | 10 | gnl\|TC-DB\|Q02563 | 2.A.1.22 | Synaptic vesicle protein 2 (SV2) - *Rattus norvegicus* (Rat). |
| ARAD1D35860g | 10 | gnl\|TC-DB\|P25568 | 2.A.1.24 | Hypothetical 58.8 |
| ARAD1C23496g | 10 | gnl\|TC-DB\|P25568 | 2.A.1.24 | Hypothetical 58.8 |
| ARAD1D28864g | 8 | gnl\|TC-DB\|O00400 | 2.A.1.25 | Acetyl-coenzyme A transporter - *Homo sapiens* (human). |
| ARAD1B19778g | 12 | gnl\|TC-DB\|Q8CE47 | 2.A.1.28 | Major facilitator superfamily domain-containing protein 7 - *Mus musculus* (Mouse). |
| ARAD1D20108g | 14 | gnl\|TC-DB\|P13090 | 2.A.1.3 | Aminotriazole resistance protein - *Saccharomyces cerevisiae* (Baker's yeast). |
| ARAD1D01320g | 12 | gnl\|TC-DB\|P13090 | 2.A.1.3 | Aminotriazole resistance protein - *Saccharomyces cerevisiae* (Baker's yeast). |
| ARAD1B22836g | 14 | gnl\|TC-DB\|P13090 | 2.A.1.3 | Aminotriazole resistance protein - *Saccharomyces cerevisiae* (Baker's yeast). |
| ARAD1C13750g | 10 | gnl\|TC-DB\|P32369 | 2.A.1.3 | Bile acid transporter - *Eubacterium sp.* (strain VPI 12708). |
| ARAD1B14916g | 14 | gnl\|TC-DB\|Q9ZGB6 | 2.A.1.3 | Drug resistance transporter LanJ [*Streptomyces cyanogenus*] |
| ARAD1B04334g | 14 | gnl\|TC-DB\|Q9ZGB6 | 2.A.1.3 | Drug resistance transporter LanJ [*Streptomyces cyanogenus*] |
| ARAD1D20680g | 14 | gnl\|TC-DB\|P42670 | 2.A.1.3 | Puromycin resistance protein pur8 - *Streptomyces lipmanii* (*Streptomyces alboniger*). |
| ARAD1D14058g | 13 | gnl\|TC-DB\|O69070 | 2.A.1.3 | Tetracycline efflux protein - *Streptomyces rimosus*. |
| ARAD1B22814g | 14 | gnl\|TC-DB\|O32182 | 2.A.1.3 | Uncharacterized MFS-type transporter yusP - *Bacillus subtilis* |
| ARAD1D40678g | 11 | gnl\|TC-DB\|O75387 | 2.A.1.44 | Large neutral amino acids transporter small subunit 3 - *Homo sapiens* (Human). |
| ARAD1C44066g | 13 | gnl\|TC-DB\|Q09752 | 2.A.1.48 | Multidrug resistance protein fnx1 - *Schizosaccharomyces pombe* (Fission yeast). |
| ARAD1C21362g | 12 | gnl\|TC-DB\|Q5A7S4 | 2.A.1.58 | Putative uncharacterized protein - *Candida albicans* (Yeast). |
| ARAD1D00946g | 11 | gnl\|TC-DB\|Q5A7S4 | 2.A.1.58 | Putative uncharacterized protein - *Candida albicans* (Yeast). |
| ARAD1D31658g | 10 | gnl\|TC-DB\|Q5A7S4 | 2.A.1.58 | Putative uncharacterized protein - *Candida albicans* (Yeast). |
| ARAD1D03124g | 9 | gnl\|TC-DB\|Q467Y6 | 2.A.1.63 | Transport protein - Methanosarcina barkeri (strain Fusaro / DSM 804). |
| ARAD1C16346g | 12 | gnl\|TC-DB\|Q07W00 | 2.A.1.7 | Major facilitator superfamily MFS_1 - *Shewanella frigidimarina* (strain NCIMB 400). |
| ARAD1B04554g | 12 | gnl\|TC-DB\|Q07W00 | 2.A.1.7 | Major facilitator superfamily MFS_1 - *Shewanella frigidimarina* (strain NCIMB 400). |
| ARAD1C19250g | 10 | gnl\|TC-DB\|P22152 | 2.A.1.8 | Nitrate transporter (Nitrate permease) - *Emericella nidulans* (Aspergillus nidulans). |
| ARAD1C27632g | 9 | gnl\|TC-DB\|P22152 | 2.A.1.8 | Nitrate transporter (Nitrate permease) - *Emericella nidulans* (Aspergillus nidulans). |
| ARAD1C19206g | 8 | gnl\|TC-DB\|P22152 | 2.A.1.8 | Nitrate transporter (Nitrate permease) - *Emericella nidulans* (Aspergillus nidulans). |
| ARAD1D22352g | 10 | gnl\|TC-DB\|Q01MW8 | 2.A.1.9 | Probable inorganic phosphate transporter 1-4 - *Oryza sativa subsp. indica* |
| ARAD1A19360g | 9 | gnl\|TC-DB\|Q01MW8 | 2.A.1.9 | Probable inorganic phosphate transporter 1-4 - *Oryza sativa subsp. indica* |
| ARAD1D45606g | 8 | gnl\|TC-DB\|Q01MW8 | 2.A.1.9 | Probable inorganic phosphate transporter 1-4 - *Oryza sativa subsp. indica* |
| ARAD1A09416g | 8 | gnl\|TC-DB\|A2QYD7 | 2.A.16.4 | Plasma membrane free sulphite efflux pump Ssu1p - *Aspergillus niger*. |
| ARAD1D03740g | 11 | gnl\|TC-DB\|P32901 | 2.A.17.2 | Peptide transporter Ptr2 (peptide permease Ptr2) - *Saccharomyces cerevisiae* |
| ARAD1C43318g | 11 | gnl\|TC-DB\|P32901 | 2.A.17.2 | Peptide transporter Ptr2 (peptide permease Ptr2) - *Saccharomyces cerevisiae* |

| | | | | |
|---|---|---|---|---|
| ARAD1A19910g | 11 | gnl\|TC-DB\|Q6IT47 | 2.A.18.4 | Aromatic and neutral aliphatic amino acid permease - *Penicillium chrysogenum* |
| ARAD1D06270g | 9 | gnl\|TC-DB\|Q6IT47 | 2.A.18.4 | Aromatic and neutral aliphatic amino acid permease - *Penicillium chrysogenum* |
| ARAD1A14564g | 11 | gnl\|TC-DB\|Q6IT47 | 2.A.18.4 | Aromatic and neutral aliphatic amino acid permease - *Penicillium chrysogenum* |
| ARAD1D29084g | 11 | gnl\|TC-DB\|P38680 | 2.A.18.4 | N amino acid transport system protein (Methyltryptophan resistance) - *Neurospora crassa* . |
| ARAD1A06578g | 11 | gnl\|TC-DB\|P47082 | 2.A.18.5 | Hypothetical 65.3 |
| ARAD1C44528g | 11 | gnl\|TC-DB\|Q91XR7 | 2.A.18.6 | Amino acid transporter system N2 - *Rattus norvegicus* (Rat). |
| ARAD1B21538g | 10 | gnl\|TC-DB\|P40074 | 2.A.18.6 | Hypothetical 48.8 |
| ARAD1D35376g | 11 | gnl\|TC-DB\|P36062 | 2.A.18.7 | Hypothetical 75.5 |
| ARAD1B22440g | 11 | gnl\|TC-DB\|Q99385 | 2.A.19.2 | Vacuolar H+/Ca++ exchanger YDL128W - *Saccharomyces cerevisiae* (Baker's yeast). |
| ARAD1D46904g | 11 | gnl\|TC-DB\|Q6J4K2 | 2.A.19.4 | Sodium/potassium/calcium exchanger 6 - *Homo sapiens* |
| ARAD1D30162g | 13 | gnl\|TC-DB\|P42839 | 2.A.19.7 | Putative cation exchanger YNL321W - *Saccharomyces cerevisiae* (Baker's yeast). |
| ARAD1B10142g | 12 | gnl\|TC-DB\|O14091 | 2.A.2.6 | Hypothetical 61.7 |
| ARAD1C07238g | 12 | gnl\|TC-DB\|O14091 | 2.A.2.6 | Hypothetical 61.7 |
| ARAD1D06468g | 9 | gnl\|TC-DB\|P15710 | 2.A.20.2 | Phosphate-repressible phosphate permease - *Neurospora crassa.* |
| ARAD1B12386g | 11 | gnl\|TC-DB\|P33413 | 2.A.21.6 | Urea active transporter - *Saccharomyces cerevisiae* (baker's yeast). |
| ARAD1D16852g | 15 | gnl\|TC-DB\|P33413 | 2.A.21.6 | Urea active transporter - *Saccharomyces cerevisiae* (baker's yeast). |
| ARAD1D41294g | 3 | gnl\|TC-DB\|Q93N33 | 2.A.21.9 | Proline sensor PrlS - *Aeromonas hydrophila.* |
| ARAD1C09284g | 3 | gnl\|TC-DB\|Q93N33 | 2.A.21.9 | Proline sensor PrlS - *Aeromonas hydrophila.* |
| ARAD1B19668g | 3 | gnl\|TC-DB\|P18239 | 2.A.29.1 | ADP,ATP carrier protein 2 - *Saccharomyces cerevisiae* (Baker's yeast). |
| ARAD1D08272g | 3 | gnl\|TC-DB\|Q9H2D1 | 2.A.29.10 | Folate transporter/carrier - *Homo sapiens* (human). |
| ARAD1D13002g | 2 | gnl\|TC-DB\|Q01888 | 2.A.29.12 | Mitochondrial solute carrier protein homolog - *Bos taurus* |
| ARAD1B21252g | 4 | gnl\|TC-DB\|Q06497 | 2.A.29.17 | Similar to mitochondrial ADP/ATP carrier protein - *Saccharomyces cerevisiae* |
| ARAD1C04466g | 3 | gnl\|TC-DB\|Q06497 | 2.A.29.17 | Similar to mitochondrial ADP/ATP carrier protein - *Saccharomyces cerevisiae* |
| ARAD1B10318g | 3 | gnl\|TC-DB\|Q94AG6 | 2.A.29.18 | Putative mitochondrial carrier protein - *Arabidopsis thaliana* (Mouse-ear cress). |
| ARAD1D34716g | 2 | gnl\|TC-DB\|P38921 | 2.A.29.18 | Putative mitochondrial carrier protein PET8 - *Saccharomyces cerevisiae* (Baker's yeast). |
| ARAD1A09922g | 4 | gnl\|TC-DB\|P38921 | 2.A.29.18 | Putative mitochondrial carrier protein PET8 - *Saccharomyces cerevisiae* (Baker's yeast). |
| ARAD1D29282g | 2 | gnl\|TC-DB\|Q99297 | 2.A.29.2 | Putative mitochondrial carrier YOR222W - *Saccharomyces cerevisiae* (Baker's yeast). |
| ARAD1B01958g | 4 | gnl\|TC-DB\|P40556 | 2.A.29.27 | Putative mitochondrial carrier YIL006W - *Saccharomyces cerevisiae* (Baker's yeast). |
| ARAD1C33572g | 2 | gnl\|TC-DB\|P40035 | 2.A.29.4 | Mitochondrial phosphate carrier protein 2 - *Saccharomyces cerevisiae* |
| ARAD1C39270g | 6 | gnl\|TC-DB\|Q00319 | 2.A.29.6 | Peroxisomal membrane protein PMP47B - *Candida boidinii* (Yeast). |
| ARAD1A10912g | 3 | gnl\|TC-DB\|O43772 | 2.A.29.8 | Mitochondrial carnitine/acylcarnitine carrier protein - *Homo sapiens* (Human). |
| ARAD1D36080g | 11 | gnl\|TC-DB\|P25737 | 2.A.3.1 | Lysine-specific permease - *Escherichia coli* . |
| ARAD1D09482g | 12 | gnl\|TC-DB\|P04817 | 2.A.3.10 | Arginine permease - *Saccharomyces cerevisiae* (Baker's yeast). |
| ARAD1A00550g | 11 | gnl\|TC-DB\|P53388 | 2.A.3.10 | Dicarboxylic amino acid permease - *Saccharomyces cerevisiae* (Baker's yeast). |
| ARAD1A11946g | 12 | gnl\|TC-DB\|P43548 | 2.A.3.10 | General amino acid permease AGP3 - *Saccharomyces cerevisiae* (Baker's yeast). |
| ARAD1D17600g | 11 | gnl\|TC-DB\|P19145 | 2.A.3.10 | General amino-acid permease GAP1 - *Saccharomyces cerevisiae* (Baker's yeast). |
| ARAD1A19228g | 12 | gnl\|TC-DB\|P19145 | 2.A.3.10 | General amino-acid permease GAP1 - *Saccharomyces cerevisiae* (Baker's yeast). |
| ARAD1C04070g | 12 | gnl\|TC-DB\|P19145 | 2.A.3.10 | General amino-acid permease GAP1 - *Saccharomyces cerevisiae* (Baker's yeast). |
| ARAD1A02376g | 11 | gnl\|TC-DB\|P15380 | 2.A.3.10 | Proline-specific permease - *Saccharomyces cerevisiae* (Baker's yeast). |

| | | | | |
|---|---|---|---|---|
| ARAD1B00352g | 11 | gnl\|TC-DB\|P15380 | 2.A.3.10 | Proline-specific permease - *Saccharomyces cerevisiae* (Baker's yeast). |
| ARAD1D24222g | 11 | gnl\|TC-DB\|Q03770 | 2.A.3.10 | Putative amino-acid permease SSY1 - *Saccharomyces cerevisiae* (Baker's yeast). |
| ARAD1D20878g | 12 | gnl\|TC-DB\|Q9Y860 | 2.A.3.4 | GABA permease - *Emericella nidulans* (*Aspergillus nidulans*). |
| ARAD1D20900g | 10 | gnl\|TC-DB\|Q9Y860 | 2.A.3.4 | GABA permease - *Emericella nidulans* (*Aspergillus nidulans*). |
| ARAD1D49984g | 11 | gnl\|TC-DB\|Q9Y860 | 2.A.3.4 | GABA permease - *Emericella nidulans* (*Aspergillus nidulans*). |
| ARAD1D12584g | 12 | gnl\|TC-DB\|Q9Y860 | 2.A.3.4 | GABA permease - *Emericella nidulans* (*Aspergillus nidulans*). |
| ARAD1D04268g | 12 | gnl\|TC-DB\|P32837 | 2.A.3.4 | GABA-specific permease - *Saccharomyces cerevisiae* (Baker's yeast). |
| ARAD1D15884g | 12 | gnl\|TC-DB\|P32837 | 2.A.3.4 | GABA-specific permease - *Saccharomyces cerevisiae* (Baker's yeast). |
| ARAD1B20746g | 12 | gnl\|TC-DB\|P32837 | 2.A.3.4 | GABA-specific permease - *Saccharomyces cerevisiae* (Baker's yeast). |
| ARAD1D45210g | 12 | gnl\|TC-DB\|P32837 | 2.A.3.4 | GABA-specific permease - *Saccharomyces cerevisiae* (Baker's yeast). |
| ARAD1C11814g | 12 | gnl\|TC-DB\|P32837 | 2.A.3.4 | GABA-specific permease - *Saccharomyces cerevisiae* (Baker's yeast). |
| ARAD1D08074g | 12 | gnl\|TC-DB\|Q9UT18 | 2.A.3.4 | Uncharacterized amino-acid permease C9.10 |
| ARAD1A06600g | 10 | gnl\|TC-DB\|P82252 | 2.A.3.8 | B(0,+)-type amino acid transporter 1 - *Rattus norvegicus* (Rat). |
| ARAD1D28292g | 12 | gnl\|TC-DB\|P50276 | 2.A.3.8 | High affinity methionine permease - *Saccharomyces cerevisiae* (Baker's yeast). |
| ARAD1C23914g | 9 | gnl\|TC-DB\|O22526 | 2.A.30.6 | Cation-chloride co-transporter - *Nicotiana tabacum* (common tobacco). |
| ARAD1C19030g | 12 | gnl\|TC-DB\|P53838 | 2.A.31.3 | Boron efflux transporter - *Saccharomyces cerevisiae* (Baker's yeast). |
| ARAD1C09856g | 10 | gnl\|TC-DB\|Q04121 | 2.A.36.2 | Mitochondrial NA(+)/H(+) exchanger - *Saccharomyces cerevisiae* (Baker's yeast). |
| ARAD1C36938g | 10 | gnl\|TC-DB\|Q9P937 | 2.A.36.4 | Na+/H+ antiporter Cnh1 - *Candida albicans* (Yeast). |
| ARAD1D28578g | 10 | gnl\|TC-DB\|Q9P937 | 2.A.36.4 | Na+/H+ antiporter Cnh1 - *Candida albicans* (Yeast). |
| ARAD1D02706g | 12 | gnl\|TC-DB\|P40309 | 2.A.37.4 | Hypothetical 97.1 |
| ARAD1C44154g | 6 | gnl\|TC-DB\|Q6BYD8 | 2.A.38.2 | DhTRK1 protein - *Debaryomyces hansenii* (Yeast) (*Torulaspora hansenii*). |
| ARAD1B15598g | 6 | gnl\|TC-DB\|Q6BYD8 | 2.A.38.2 | DhTRK1 protein - *Debaryomyces hansenii* (Yeast) (*Torulaspora hansenii*). |
| ARAD1D48642g | 12 | gnl\|TC-DB\|Q708J7 | 2.A.39.2 | Putative purine-cytosine permease - *Candida albicans* (Yeast). |
| ARAD1B03256g | 12 | gnl\|TC-DB\|P53099 | 2.A.39.2 | Tpn1 64.5 |
| ARAD1A06402g | 12 | gnl\|TC-DB\|Q04895 | 2.A.39.3 | Allantoin permease (allantoin transport protein) - *Saccharomyces cerevisiae* |
| ARAD1C22902g | 12 | gnl\|TC-DB\|Q05998 | 2.A.39.4 | Thiamine transporter -*Saccharomyces cerevisiae* (baker's yeast). |
| ARAD1D24618g | 3 | gnl\|TC-DB\|O07084 | 2.A.4.1 | Cation transport protein YRDO - *Bacillus subtilis.* |
| ARAD1A12342g | 6 | gnl\|TC-DB\|P32798 | 2.A.4.2 | Cobalt uptake protein Cot1 - *Saccharomyces cerevisiae* (baker's yeast). |
| ARAD1C14410g | 6 | gnl\|TC-DB\|P53735 | 2.A.4.4 | Hypothetical 82.5 |
| ARAD1B10802g | 12 | gnl\|TC-DB\|Q03455 | 2.A.4.4 | Protein ZRG17 - *Saccharomyces cerevisiae* (Baker's yeast). |
| ARAD1A14586g | 5 | gnl\|TC-DB\|A4ZUV2 | 2.A.4.5 | Mn-specific cation diffusion facilitator transporter - *Populus trichocarpa* |
| ARAD1A08580g | 5 | gnl\|TC-DB\|A4ZUV2 | 2.A.4.5 | Mn-specific cation diffusion facilitator transporter - *Populus trichocarpa* |
| ARAD1D20966g | 12 | gnl\|TC-DB\|P48777 | 2.A.40.5 | Purine permease - *Emericella nidulans* (*aspergillus nidulans*). |
| ARAD1D46244g | 12 | gnl\|TC-DB\|Q874I3 | 2.A.41.2 | H+/nucleoside cotransporter - *Candida albicans* (Yeast). |
| ARAD1C07480g | 5 | gnl\|TC-DB\|O60931 | 2.A.43.1 | Cystinosin - *Homo sapiens* (Human). |
| ARAD1D24464g | 4 | gnl\|TC-DB\|Q60441 | 2.A.43.3 | Mannose-P-dolichol utilization defect 1 protein - *Cricetulus griseus* |
| ARAD1C43098g | 12 | gnl\|TC-DB\|P27514 | 2.A.47.2 | Uncharacterized transporter YNR013C - *Saccharomyces cerevisiae* (Baker's yeast). |
| ARAD1D30404g | 10 | gnl\|TC-DB\|A7LKG1 | 2.A.49.1 | CLC voltage-gated chloride channel - *Fusarium oxysporum f. sp. lycopersici*. |
| ARAD1D47938g | 9 | gnl\|TC-DB\|Q9GKE7 | 2.A.49.2 | Outwardly rectifying chloride channel - *Sus scrofa* (Pig). |

| | | | | |
|---|---|---|---|---|
| ARAD1B05742g | 9 | gnl\|TC-DB\|Q9GKE7 | 2.A.49.2 | Outwardly rectifying chloride channel - *Sus scrofa* (Pig). |
| ARAD1D26268g | 7 | gnl\|TC-DB\|P32804 | 2.A.5.1 | Zinc transporter ZRT1 Protein - *Saccharomyces cerevisiae* (Baker's yeast). |
| ARAD1D26290g | 7 | gnl\|TC-DB\|P32804 | 2.A.5.1 | Zinc transporter ZRT1 Protein - *Saccharomyces cerevisiae* (Baker's yeast). |
| ARAD1A06138g | 7 | gnl\|TC-DB\|P32804 | 2.A.5.1 | Zinc transporter ZRT1 Protein - *Saccharomyces cerevisiae* (Baker's yeast). |
| ARAD1A07678g | 7 | gnl\|TC-DB\|P32804 | 2.A.5.1 | Zinc transporter ZRT1 Protein - *Saccharomyces cerevisiae* (Baker's yeast). |
| ARAD1C28468g | 4 | gnl\|TC-DB\|P40544 | 2.A.5.4 | Zinc transporter YKE4 - *Saccharomyces cerevisiae* (Baker's yeast). |
| ARAD1C06380g | 7 | gnl\|TC-DB\|P0A8H3 | 2.A.5.5 | Zinc transporter zupT - *Escherichia coli* . |
| ARAD1C19338g | 11 | gnl\|TC-DB\|P53154 | 2.A.50.1 | Hypothetical 65.3 |
| ARAD1B18326g | 9 | gnl\|TC-DB\|P38359 | 2.A.53.1 | Sulfate transporter 1 (high-affinity) - *Saccharomyces cerevisiae* (baker's yeast). |
| ARAD1B08954g | 9 | gnl\|TC-DB\|Q9SAY1 | 2.A.53.1 | Sulfate transporter 1.1 |
| ARAD1B20284g | 11 | gnl\|TC-DB\|Q09764 | 2.A.53.7 | Hypothetical protein C24H6.11c in chromosome I - *Schizosaccharomyces pombe* |
| ARAD1D30580g | 4 | gnl\|TC-DB\|Q63965 | 2.A.54.1 | Tricarboxylate carrier (fragment) - *Rattus norvegicus* (rat). |
| ARAD1C27698g | 11 | gnl\|TC-DB\|P38778 | 2.A.55.1 | Transporter protein Smf1/Esp1 - *Saccharomyces cerevisiae* (baker's yeast). |
| ARAD1C06358g | 11 | gnl\|TC-DB\|P38925 | 2.A.55.1 | Transporter protein Smf1/Esp1 - *Saccharomyces cerevisiae* (baker's yeast). |
| ARAD1D09812g | 11 | gnl\|TC-DB\|P31381 | 2.A.57.3 | Nucleoside transporter Fun26 - *Saccharomyces cerevisiae* (baker's yeast). |
| ARAD1D45496g | 10 | gnl\|TC-DB\|P45946 | 2.A.59.1 | Hypothetical protein yqcL - *Bacillus subtilis* . |
| ARAD1D41008g | 6 | gnl\|TC-DB\|P04035 | 2.A.6.6 | 3-hydroxy-3-methylglutaryl-coenzyme A reductase - *Homo sapiens* (human). |
| ARAD1D13640g | 12 | gnl\|TC-DB\|Q12200 | 2.A.6.6 | Hypothetical 132.6 |
| ARAD1D41470g | 8 | gnl\|TC-DB\|P97260 | 2.A.6.6 | SREBP cleavage activating protein - *Cricetulus griseus* (Chinese hamster). |
| ARAD1D09042g | 12 | gnl\|TC-DB\|P38767 | 2.A.66.1 | Hypothetical 64.2 |
| ARAD1D22022g | 10 | gnl\|TC-DB\|P38767 | 2.A.66.1 | Hypothetical 64.2 |
| ARAD1D11110g | 10 | gnl\|TC-DB\|P38206 | 2.A.66.3 | Nuclear division RFT1 protein - *Saccharomyces cerevisiae* (Baker's yeast). |
| ARAD1A01166g | 15 | gnl\|TC-DB\|O14411 | 2.A.67.1 | OPT1P - *Candida albicans* (Yeast). |
| ARAD1B01254g | 13 | gnl\|TC-DB\|Q06593 | 2.A.67.1 | Hypothetical Protein HRD799 - *Saccharomyces cerevisiae* (Baker's yeast). |
| ARAD1B14564g | 12 | gnl\|TC-DB\|Q06593 | 2.A.67.1 | Hypothetical Protein HRD799 - *Saccharomyces cerevisiae* (Baker's yeast). |
| ARAD1B01540g | 11 | gnl\|TC-DB\|Q06593 | 2.A.67.1 | Hypothetical Protein HRD799 - *Saccharomyces cerevisiae* (Baker's yeast). |
| ARAD1C10934g | 14 | gnl\|TC-DB\|Q06593 | 2.A.67.1 | Hypothetical Protein HRD799 - *Saccharomyces cerevisiae* (Baker's yeast). |
| ARAD1D42988g | 12 | gnl\|TC-DB\|O14031 | 2.A.67.1 | Uncharacterized oligopeptide transporter C29B12.10c - *Schizosaccharomyces pombe* |
| ARAD1C13178g | 13 | gnl\|TC-DB\|Q54EF1 | 2.A.67.2 | Putative uncharacterized protein - *Dictyostelium discoideum* |
| ARAD1D28072g | 9 | gnl\|TC-DB\|Q00974 | 2.A.7.10 | UDP N-acetylglucosamine transporter - *Kluyveromyces lactis* (Yeast). |
| ARAD1C27478g | 10 | gnl\|TC-DB\|O64503 | 2.A.7.11 | Expressed protein - *Arabidopsis thaliana* (Mouse-ear cress). |
| ARAD1C40700g | 7 | gnl\|TC-DB\|O16658 | 2.A.7.12 | Putative uncharacterized protein - *Caenorhabditis elegans* . |
| ARAD1D08646g | 9 | gnl\|TC-DB\|Q96WN8 | 2.A.7.13 | Golgi GDP-mannose transporter - *Candida albicans* (Yeast). |
| ARAD1D04576g | 10 | gnl\|TC-DB\|Q9SFT8 | 2.A.7.24 | Putative integral membrane protein - *Arabidopsis thaliana* (Mouse-ear cress). |
| ARAD1A13882g | 8 | gnl\|TC-DB\|Q04083 | 2.A.7.24 | Thiamine-repressible mitochondrial transport protein THI74 - *Saccharomyces cerevisiae* |
| ARAD1B20196g | 10 | gnl\|TC-DB\|Q04083 | 2.A.7.24 | Thiamine-repressible mitochondrial transport protein THI74 - *Saccharomyces cerevisiae* |
| ARAD1B21626g | 8 | gnl\|TC-DB\|Q9LIR9 | 2.A.7.25 | Gb\|AAF34307.1 |
| ARAD1C42988g | 9 | gnl\|TC-DB\|Q7RWT8 | 2.A.7.25 | Putative uncharacterized protein - *Neurospora crassa* . |
| ARAD1B17006g | 8 | gnl\|TC-DB\|P22215 | 2.A.7.9 | SLY41 protein - *Saccharomyces cerevisiae* (Baker's yeast). |

| | | | | |
|---|---|---|---|---|
| ARAD1D06908g | 9 | gnl\|TC-DB\|Q10495 | 2.A.85.3 | Hypothetical 111.4 |
| ARAD1D23386g | 7 | gnl\|TC-DB\|Q10495 | 2.A.85.3 | Hypothetical 111.4 |
| ARAD1C21824g | 8 | gnl\|TC-DB\|Q10495 | 2.A.85.3 | Hypothetical 111.4 |
| ARAD1C39710g | 10 | gnl\|TC-DB\|Q10495 | 2.A.85.3 | Hypothetical 111.4 |
| ARAD1A17996g | 2 | gnl\|TC-DB\|Q25771 | 2.A.85.4 | Asparagine-rich antigen (clone 25c4) (fragment) - *Plasmodium falciparum.* |
| ARAD1A06820g | 3 | gnl\|TC-DB\|P47818 | 2.A.89.1 | Protein CCC1 - *Saccharomyces cerevisiae* (Baker's yeast). |
| ARAD1B01188g | 4 | gnl\|TC-DB\|P39952 | 2.A.9.1 | Cytochrome oxidase biogenesis protein Oxa1 - *Saccharomyces cerevisiae* (baker's yeast). |
| ARAD1C39754g | 10 | gnl\|TC-DB\|Q8BY89 | 2.A.92.1 | Choline transporter-like protein 2 - *Mus musculus* (Mouse). |
| ARAD1B15180g | 3 | gnl\|TC-DB\|B0WNM6 | 2.A.94.1 | Xenotropic and polytropic murine leukemia virus receptor xpr1 - *Culex quinquefasciatus* |
| ARAD1C31768g | 4 | gnl\|TC-DB\|B0WNM6 | 2.A.94.1 | Xenotropic and polytropic murine leukemia virus receptor xpr1 - *Culex quinquefasciatus* |
| ARAD1C23254g | 6 | gnl\|TC-DB\|B0WNM6 | 2.A.94.1 | Xenotropic and polytropic murine leukemia virus receptor xpr1 - *Culex quinquefasciatus* |
| ARAD1D46134g | 3 | gnl\|TC-DB\|B0WNM6 | 2.A.94.1 | Xenotropic and polytropic murine leukemia virus receptor xpr1 - *Culex quinquefasciatus* |
| ARAD1A09152g | 6 | gnl\|TC-DB\|P25613 | 2.A.96.1 | Uncharacterized protein YCR010C - *Saccharomyces cerevisiae* GN=YCR010C |
| ARAD1A17248g | 6 | gnl\|TC-DB\|P25613 | 2.A.96.1 | Uncharacterized protein YCR010C - *Saccharomyces cerevisiae* GN=YCR010C |
| ARAD1D43692g | 5 | gnl\|TC-DB\|P25613 | 2.A.96.1 | Uncharacterized protein YCR010C - *Saccharomyces cerevisiae* GN=YCR010C |
| ARAD1A01188g | 5 | gnl\|TC-DB\|P25613 | 2.A.96.1 | Uncharacterized protein YCR010C - *Saccharomyces cerevisiae* GN=YCR010C |
| ARAD1A01276g | 6 | gnl\|TC-DB\|P25613 | 2.A.96.1 | Uncharacterized protein YCR010C - *Saccharomyces cerevisiae* GN=YCR010C |
| ARAD1A15114g | 12 | gnl\|TC-DB\|P08183 | 3.A.1.201 | Multidrug resistance protein 1 (P-glycoprotein 1) - *Homo sapiens* (Human). |
| ARAD1C40040g | 2 | gnl\|TC-DB\|P33897 | 3.A.1.203 | Adrenoleukodystrophy protein (ALDP) - *Homo sapiens* (Human). |
| ARAD1C11440g | 3 | gnl\|TC-DB\|P33897 | 3.A.1.203 | Adrenoleukodystrophy protein (ALDP) - *Homo sapiens* (Human). |
| ARAD1C37202g | 7 | gnl\|TC-DB\|A8WEV1 | 3.A.1.204 | ABC transporter ABCG6 - *Leishmania donovani* . |
| ARAD1D38104g | 11 | gnl\|TC-DB\|Q9H221 | 3.A.1.204 | ATP-binding cassette sub-family G member 8 (Sterolin-2) - *Homo sapiens* (Human). |
| ARAD1A14146g | 6 | gnl\|TC-DB\|Q9C8K2 | 3.A.1.204 | ATP-dependent transmembrane transporter - *Arabidopsis thaliana* |
| ARAD1C43582g | 12 | gnl\|TC-DB\|P43071 | 3.A.1.205 | Multidrug resistance protein CDR1 - *Candida albicans* (Yeast). |
| ARAD1D07634g | 13 | gnl\|TC-DB\|P43071 | 3.A.1.205 | Multidrug resistance protein CDR1 - *Candida albicans* (Yeast). |
| ARAD1A12298g | 10 | gnl\|TC-DB\|P32568 | 3.A.1.205 | SNQ2 protein - *Saccharomyces cerevisiae* (Baker's yeast). |
| ARAD1D20724g | 12 | gnl\|TC-DB\|P32568 | 3.A.1.205 | SNQ2 protein - *Saccharomyces cerevisiae* (Baker's yeast). |
| ARAD1B20988g | 12 | gnl\|TC-DB\|P32568 | 3.A.1.205 | SNQ2 protein - *Saccharomyces cerevisiae* (Baker's yeast). |
| ARAD1D37796g | 12 | gnl\|TC-DB\|P12866 | 3.A.1.206 | Mating factor A secretion protein STE6 - *Saccharomyces cerevisiae* (Baker's yeast). |
| ARAD1C02970g | 16 | gnl\|TC-DB\|P32386 | 3.A.1.208 | ATP-dependent bile acid permease - *Saccharomyces cerevisiae* (Baker's yeast). |
| ARAD1D22132g | 16 | gnl\|TC-DB\|P39109 | 3.A.1.208 | Metal resistance protein YCF1 - *Saccharomyces cerevisiae* (Baker's yeast). |
| ARAD1D25124g | 14 | gnl\|TC-DB\|P39109 | 3.A.1.208 | Metal resistance protein YCF1 - *Saccharomyces cerevisiae* (Baker's yeast). |
| ARAD1A18106g | 10 | gnl\|TC-DB\|P53049 | 3.A.1.208 | Oligomycin resistance ATP-dependent permease YOR1 - *Saccharomyces cerevisiae* |
| ARAD1D05544g | 5 | gnl\|TC-DB\|P40416 | 3.A.1.210 | Mitochondrial transporter ATM1 precursor - Saccharomyces cerevisiae (Baker's yeast). |
| ARAD1B18150g | 14 | gnl\|TC-DB\|P78363 | 3.A.1.211 | Retinal-specific ATP-binding cassette transporter - *Homo sapiens* (Human). |
| ARAD1C13288g | 5 | gnl\|TC-DB\|P33310 | 3.A.1.212 | ATP-dependent permease MDL1 - *Saccharomyces cerevisiae* (Baker's yeast). |
| ARAD1A07766g | 4 | gnl\|TC-DB\|P46889 | 3.A.12.1 | Cell division protein ftsK - *Escherichia coli* . |
| ARAD1D37180g | 2 | gnl\|TC-DB\|P25694 | 3.A.16.1 | Cell division control protein 48 - *Saccharomyces cerevisiae* (Baker's yeast). |
| ARAD1C38610g | 4 | gnl\|TC-DB\|Q9BUN8 | 3.A.16.1 | Transitional endoplasmic reticulum ATPase (TER ATPase) - *Homo sapiens* (Human). |

| | | | | |
|---|---|---|---|---|
| ARAD1C16214g | 4 | gnl\|TC-DB\|Q9UKV3 | 3.A.18.1 | Apoptotic chromatin condensation inducer in the nucleus - *Homo sapiens* (Human). |
| ARAD1C11704g | 3 | gnl\|TC-DB\|Q8IYB3 | 3.A.18.1 | Eukaryotic initiation factor 4A-III - *Homo sapiens* (Human). |
| ARAD1A10296g | 4 | gnl\|TC-DB\|Q8IYB3 | 3.A.18.1 | Serine/arginine repetitive matrix protein 1 - *Homo sapiens* (Human). |
| ARAD1C03278g | 4 | gnl\|TC-DB\|P32842 | 3.A.2.2 | Vacuolar ATP synthase subunit b - *Saccharomyces cerevisiae* |
| ARAD1C30008g | 4 | gnl\|TC-DB\|P25515 | 3.A.2.2 | Vacuolar ATP synthase subunit b - *Saccharomyces cerevisiae* |
| ARAD1C38280g | 5 | gnl\|TC-DB\|P23968 | 3.A.2.2 | Vacuolar ATP synthase subunit b - *Saccharomyces cerevisiae* |
| ARAD1D18832g | 7 | gnl\|TC-DB\|P32563 | 3.A.2.2 | Vacuolar ATP synthase subunit b - *Saccharomyces cerevisiae* |
| ARAD1B14322g | 6 | gnl\|TC-DB\|P32563 | 3.A.2.2 | Vacuolar ATP synthase subunit b - *Saccharomyces cerevisiae* |
| ARAD1D35706g | 10 | gnl\|TC-DB\|P50993 | 3.A.3.1 | Sodium/potassium-transporting ATPase subunit alpha-2 - *Homo sapiens* |
| ARAD1B19404g | 7 | gnl\|TC-DB\|P39986 | 3.A.3.10 | Probable cation-transporting ATPase 1 - *Saccharomyces cerevisiae* (Baker's yeast). |
| ARAD1D44946g | 10 | gnl\|TC-DB\|Q54X63 | 3.A.3.15 | Cation-transporting ATPase - *Dictyostelium discoideum* AX4. |
| ARAD1D42218g | 8 | gnl\|TC-DB\|P13586 | 3.A.3.2 | Calcium-transporting ATPase 1 - *Saccharomyces cerevisiae* (baker's yeast). |
| ARAD1A06798g | 7 | gnl\|TC-DB\|P54678 | 3.A.3.2 | Probable calcium-transporting ATPase PAT1 - *Dictyostelium discoideum* (Slime mold). |
| ARAD1C10296g | 8 | gnl\|TC-DB\|P16615 | 3.A.3.2 | Sarcoplasmic/endoplasmic reticulum calcium ATPase 2 |
| ARAD1C39468g | 10 | gnl\|TC-DB\|P07038 | 3.A.3.3 | Plasma membrane ATPase (EC 3.6.3.6) (proton pump) - *Neurospora crassa* . |
| ARAD1C41800g | 9 | gnl\|TC-DB\|P07038 | 3.A.3.3 | Plasma membrane ATPase (EC 3.6.3.6) (proton pump) - *Neurospora crassa* . |
| ARAD1D15114g | 8 | gnl\|TC-DB\|Q9UVL6 | 3.A.3.5 | Copper resistance-associated P-type ATPase - *Candida albicans* (Yeast). |
| ARAD1D04444g | 8 | gnl\|TC-DB\|P38995 | 3.A.3.5 | Copper-transporting ATPase - *Saccharomyces cerevisiae* (Baker's yeast). |
| ARAD1D36564g | 7 | gnl\|TC-DB\|P39524 | 3.A.3.8 | Potential phospholipid-transporting ATPase 1 - *Saccharomyces cerevisiae* |
| ARAD1C23694g | 5 | gnl\|TC-DB\|P39524 | 3.A.3.8 | Potential phospholipid-transporting ATPase 1 - *Saccharomyces cerevisiae* |
| ARAD1C07084g | 10 | gnl\|TC-DB\|P32660 | 3.A.3.8 | Probable phospholipid-transporting ATPase DNF1 - *Saccharomyces cerevisiae* |
| ARAD1D11220g | 10 | gnl\|TC-DB\|P32660 | 3.A.3.8 | Probable phospholipid-transporting ATPase DNF1 - *Saccharomyces cerevisiae* |
| ARAD1D01364g | 7 | gnl\|TC-DB\|Q9XIE6 | 3.A.3.8 | Putative phospholipid-transporting ATPase 3 - *Arabidopsis thaliana* (Mouse-ear cress). |
| ARAD1D26796g | 10 | gnl\|TC-DB\|O13398 | 3.A.3.9 | P-type ATPase 2 - *Debaryomyces occidentalis* (Yeast) (*Schwanniomyces occidentalis* ). |
| ARAD1D33770g | 8 | gnl\|TC-DB\|P32915 | 3.A.5.8 | Signal recognition particle receptor alpha subunit homolog - *Saccharomyces cerevisiae* |
| ARAD1D17028g | 2 | gnl\|TC-DB\|P21825_I | 3.A.5.8 | Translocation protein (NPL1 protein) - *Saccharomyces cerevisiae* (Baker's yeast). |
| ARAD1A16566g | 3 | gnl\|TC-DB\|P14906 | 3.A.5.8 | Translocation protein SEC66 (HSS1 protein) - *Saccharomyces cerevisiae* (Baker's yeast). |
| ARAD1B03894g | 8 | gnl\|TC-DB\|P32915 | 3.A.5.8 | Translocation protein SEC72 (P23) - *Saccharomyces cerevisiae* (Baker's yeast). |
| ARAD1D01958g | 3 | gnl\|TC-DB\|Q9UGP8 | 3.A.5.9 | Signal recognition particle 68 kDa protein (SRP68) - *Homo sapiens* (Human). |
| ARAD1C41646g | 3 | gnl\|TC-DB\|P39515 | 3.A.8.1 | Mitochondrial import inner membrane translocase subunit TIM22 - *Saccharomyces cerevisiae* |
| ARAD1D14542g | 4 | gnl\|TC-DB\|P25710 | 3.D.1.6 | NADH:ubiquinone oxidoreductase 14kD subunit - *Neurospora crassa* . |
| ARAD1B03476g | 5 | gnl\|TC-DB\|P21592 | 3.D.4.8 | Cytochrome c oxidase polypeptide VII (EC 1.9.3.1) - *Saccharomyces cerevisiae* |
| ARAD1C15598g | 2 | gnl\|TC-DB\|P53266 | 3.D.4.8 | SHY1 protein - i (Baker's yeast). |
| ARAD1D36212g | 7 | gnl\|TC-DB\|O74631 | 3.E.1.5 | Protein FDD123 (CVHSP30/1) - *Coriolus versicolor* . |
| ARAD1C06138g | 7 | gnl\|TC-DB\|P32791 | 5.B.1.5 | Ferric reductase transmembrane component 1 precursor - *Saccharomyces cerevisiae* |
| ARAD1D37862g | 6 | gnl\|TC-DB\|P32791 | 5.B.1.5 | Ferric reductase transmembrane component 1 precursor - *Saccharomyces cerevisiae* |
| ARAD1D26136g | 6 | gnl\|TC-DB\|P32791 | 5.B.1.5 | Ferric reductase transmembrane component 1 precursor - *Saccharomyces cerevisiae* |
| ARAD1C28952g | 6 | gnl\|TC-DB\|P32791 | 5.B.1.5 | Ferric reductase transmembrane component 1 precursor - *Saccharomyces cerevisiae* |
| ARAD1D38060g | 6 | gnl\|TC-DB\|P32791 | 5.B.1.5 | Ferric reductase transmembrane component 1 precursor - *Saccharomyces cerevisiae* |

| | | | | |
|---|---|---|---|---|
| ARAD1D04136g | 6 | gnl\|TC-DB\|P32791 | 5.B.1.5 | Ferric reductase transmembrane component 1 precursor - *Saccharomyces cerevisiae* |
| ARAD1B03432g | 6 | gnl\|TC-DB\|P32791 | 5.B.1.5 | Ferric reductase transmembrane component 1 precursor - *Saccharomyces cerevisiae* |
| ARAD1D50996g | 6 | gnl\|TC-DB\|P32791 | 5.B.1.5 | Ferric reductase transmembrane component 1 precursor - *Saccharomyces cerevisiae* |
| ARAD1A00264g | 7 | gnl\|TC-DB\|Q12473 | 5.B.1.7 | Ferric reductase transmembrane component 6 - *Saccharomyces cerevisiae* |
| ARAD1A00286g | 7 | gnl\|TC-DB\|Q12473 | 5.B.1.7 | Ferric reductase transmembrane component 6 - *Saccharomyces cerevisiae* |
| ARAD1D40370g | 8 | gnl\|TC-DB\|Q12473 | 5.B.1.7 | Ferric reductase transmembrane component 6 - *Saccharomyces cerevisiae* |
| ARAD1D25784g | 6 | gnl\|TC-DB\|Q9WUE3 | 5.B.2.1 | 101F6 protein (Tumor suppressor region 10) - *Mus musculus* (Mouse). |
| ARAD1B20570g | 2 | gnl\|TC-DB\|P25656 | 8.A.27.1 | Cell division control protein 50 - *Saccharomyces cerevisiae* (Baker's yeast). |
| ARAD1A13288g | 7 | gnl\|TC-DB\|Q9P8U8 | 9.A.10.1 | High-affinity iron permease CaFTR2 - *Candida albicans* (Yeast). |
| ARAD1A02970g | 7 | gnl\|TC-DB\|Q9P8U8 | 9.A.10.1 | High-affinity iron permease CaFTR2 - *Candida albicans* (Yeast). |
| ARAD1A02992g | 7 | gnl\|TC-DB\|Q9P8U8 | 9.A.10.1 | High-affinity iron permease CaFTR2 - *Candida albicans* (Yeast). |
| ARAD1C27610g | 3 | gnl\|TC-DB\|P39685 | 9.A.14.1 | Nucleoporin GLE1 - *Saccharomyces cerevisiae* (Baker's yeast). |
| ARAD1C28028g | 2 | gnl\|TC-DB\|Q12445 | 9.A.14.1 | Nucleoporin GLE2 - *Saccharomyces cerevisiae* (Baker's yeast). |
| ARAD1C08932g | 3 | gnl\|TC-DB\|Q02455 | 9.A.14.1 | Protein MLP1 - *Saccharomyces cerevisiae* (Baker's yeast). |
| ARAD1C19316g | 7 | gnl\|TC-DB\|P53047 | 9.A.26.1 | RTA1 protein - *Saccharomyces cerevisiae* (Baker's yeast). |
| ARAD1A19184g | 7 | gnl\|TC-DB\|Q08417 | 9.A.26.1 | *S.cerevisiae* chromosome XV reading frame ORF YOR049c - *Saccharomyces cerevisiae* |
| ARAD1D27698g | 8 | gnl\|TC-DB\|Q08417 | 9.A.26.1 | *S.cerevisiae* chromosome XV reading frame ORF YOR049c - *Saccharomyces cerevisiae* |
| ARAD1D02684g | 7 | gnl\|TC-DB\|P40100 | 9.A.26.1 | Uncharacterized protein YER185W - *Saccharomyces cerevisiae* (Baker's yeast). |
| ARAD1C44088g | 7 | gnl\|TC-DB\|P40100 | 9.A.26.1 | Uncharacterized protein YER185W - *Saccharomyces cerevisiae* (Baker's yeast). |
| ARAD1D13090g | 4 | gnl\|TC-DB\|Q12207 | 9.A.27.1 | Non-classical export protein 2 - *Saccharomyces cerevisiae* (Baker's yeast). |
| ARAD1D20526g | 4 | gnl\|TC-DB\|Q3TWN3 | 9.A.40.3 | Metal transporter CNNM2 - *Mus musculus* (Mouse). |
| ARAD1D41734g | 5 | gnl\|TC-DB\|Q3TWN3 | 9.A.40.3 | Metal transporter CNNM2 - *Mus musculus* (Mouse). |
| ARAD1A06556g | 4 | gnl\|TC-DB\|Q13454 | 9.A.45.1 | Tumor suppressor candidate 3 - *Homo sapiens* GN=TUSC3 PE=2 SV=1 |
| ARAD1D32538g | 4 | gnl\|TC-DB\|P38224 | 9.A.46.1 | Factor-induced gene 1 protein - *Saccharomyces cerevisiae* (Baker's yeast). |
| ARAD1D27258g | 4 | gnl\|TC-DB\|P38224 | 9.A.46.1 | Factor-induced gene 1 protein - *Saccharomyces cerevisiae* (Baker's yeast). |
| ARAD1C21450g | 16 | gnl\|TC-DB\|P36051 | 9.A.6.1 | GPI-anchor biosynthetic protein MCD4 - *Saccharomyces cerevisiae* (Baker's yeast). |
| ARAD1C06490g | 10 | gnl\|TC-DB\|P36051 | 9.A.6.1 | GPI-anchor biosynthetic protein MCD4 - *Saccharomyces cerevisiae* (Baker's yeast). |
| ARAD1C05786g | 16 | gnl\|TC-DB\|P36051 | 9.A.6.1 | GPI-anchor biosynthetic protein MCD4 - *Saccharomyces cerevisiae* (Baker's yeast). |
| ARAD1D06204g | 2 | gnl\|TC-DB\|P38356 | 9.B.1.1 | Metal homeostasis protein BSD2 - *Saccharomyces cerevisiae* (Baker's yeast). |
| ARAD1C09966g | 2 | gnl\|TC-DB\|P14359 | 9.B.12.3 | Hypothetical 15.2 |
| ARAD1C00968g | 7 | gnl\|TC-DB\|Q12067_ | 9.B.19.1 | Metal homeostasis protein ATX2 - *Saccharomyces cerevisiae* (Bakers yeast). |
| ARAD1B19118g | 7 | gnl\|TC-DB\|Q63863 | 9.B.24.3 | NMDA receptor glutamate-binding subunit - *Rattus sp.* |
| ARAD1D19052g | 4 | gnl\|TC-DB\|Q10320 | 9.B.26.3 | Hypothetical 31.3 |
| ARAD1D36058g | 4 | gnl\|TC-DB\|P0C0H0 | 9.B.32.1 | Hyaluronan synthase - *Streptococcus pyogenes.* |
| ARAD1C38104g | 6 | gnl\|TC-DB\|P0C0H0 | 9.B.32.1 | Hyaluronan synthase - *Streptococcus pyogenes* . |
| ARAD1C37862g | 7 | gnl\|TC-DB\|Q54066 | 9.B.32.1 | Poly-beta-1,6-N-acetyl-D-glucosamine synthase ICAA - *Staphylococcus epidermidis.* |
| ARAD1C38126g | 6 | gnl\|TC-DB\|Q54066 | 9.B.32.1 | Poly-beta-1,6-N-acetyl-D-glucosamine synthase ICAA - *Staphylococcus epidermidis.* |
| ARAD1D48466g | 5 | gnl\|TC-DB\|Q8IUH5 | 9.B.37.1 | Palmitoyltransferase ZDHHC17 - *Homo sapiens* |
| ARAD1C41690g | 5 | gnl\|TC-DB\|Q8IUH5 | 9.B.37.1 | Palmitoyltransferase ZDHHC17 - *Homo sapiens* |

| | | | | |
|---|---|---|---|---|
| ARAD1C27368g | 4 | gnl\|TC-DB\|Q8R173 | 9.B.37.2 | Palmitoyltransferase ZDHHC3 - *Mus musculus* |
| ARAD1D07326g | 4 | gnl\|TC-DB\|Q8R173 | 9.B.37.2 | Palmitoyltransferase ZDHHC3 - *Mus musculus* |
| ARAD1B07040g | 4 | gnl\|TC-DB\|Q8R173 | 9.B.37.2 | Palmitoyltransferase ZDHHC3 - *Mus musculus* |
| ARAD1D07568g | 4 | gnl\|TC-DB\|P31379 | 9.B.7.2 | Uncharacterized membrane protein YAL018C - *Saccharomyces cerevisiae* (Baker's yeast). |
| ARAD1D17952g | 3 | gnl\|TC-DB\|P25560 | 9.B.82.1 | Protein RER1 involved in retention of membrane proteins - *Saccharomyces cerevisiae* |

## Table S10C Class of transporters amplified in *A. adeninivorans* compared to two other pre-CTG species

| ID-TCDB | ARAD | PIPA | YALI | ACRONYM | FAMILY NAME |
|---|---|---|---|---|---|
| 01.A.01.10 | 1 | none | none | VIC | Voltage-sensitive Na+Channel |
| 02.A.01.01 | 60 | 17 | 24 | SP | Sugar Porter |
| 02.A.01.08 | 3 | none | none | NPP | Nitrate/Nitrite Porter |
| 02.A.01.19 | 1 | none | none | AAHS | Aromatic Acid:$H^+$ Symporter |
| 02.A.01.15 | 1 | none | none | OCS | Organic Cation Transporter |
| 02.A.01.58 | 3 | 1 | 1 | NAG-T | N-Acetylglucosamine Transporter |
| 02.A.29.17 | 1 | none | none | MC | Peroxisomal ATP/ADP/AMP Antiporter, |
| 02.A.29.18 | 1 | none | none | MC | Mitochondrial S-adenosylmethionine Carrier, |
| 02.A.29.27 | 1 | none | none | MC | Mitochondrial $NAD^+$ Transporter, |
| 03.A.01.204 | 3 | none | 1 | ABCG | Eye Pigment Precursor Transporter |
| 03.A.12.01 | 1 | none | none | S-DNA-T | Septal DNA Translocator |
| 03.D.01.06. | 1 | none | none | NDH | Animal $H^+$-translocating NADH Dehydrogenase Complex |
| 09.A.40.03 | 2 | none | none | HCC | HlyC/CorC Family (HemolysinC/Co2+-resistance Protein) |
| 09.A.46.01 | 2 | none | 1 | LACC | Putative Low Affinity $Ca^{2+}$ Channel Family |

# S10D Sugar porters from ARAD & DEHA

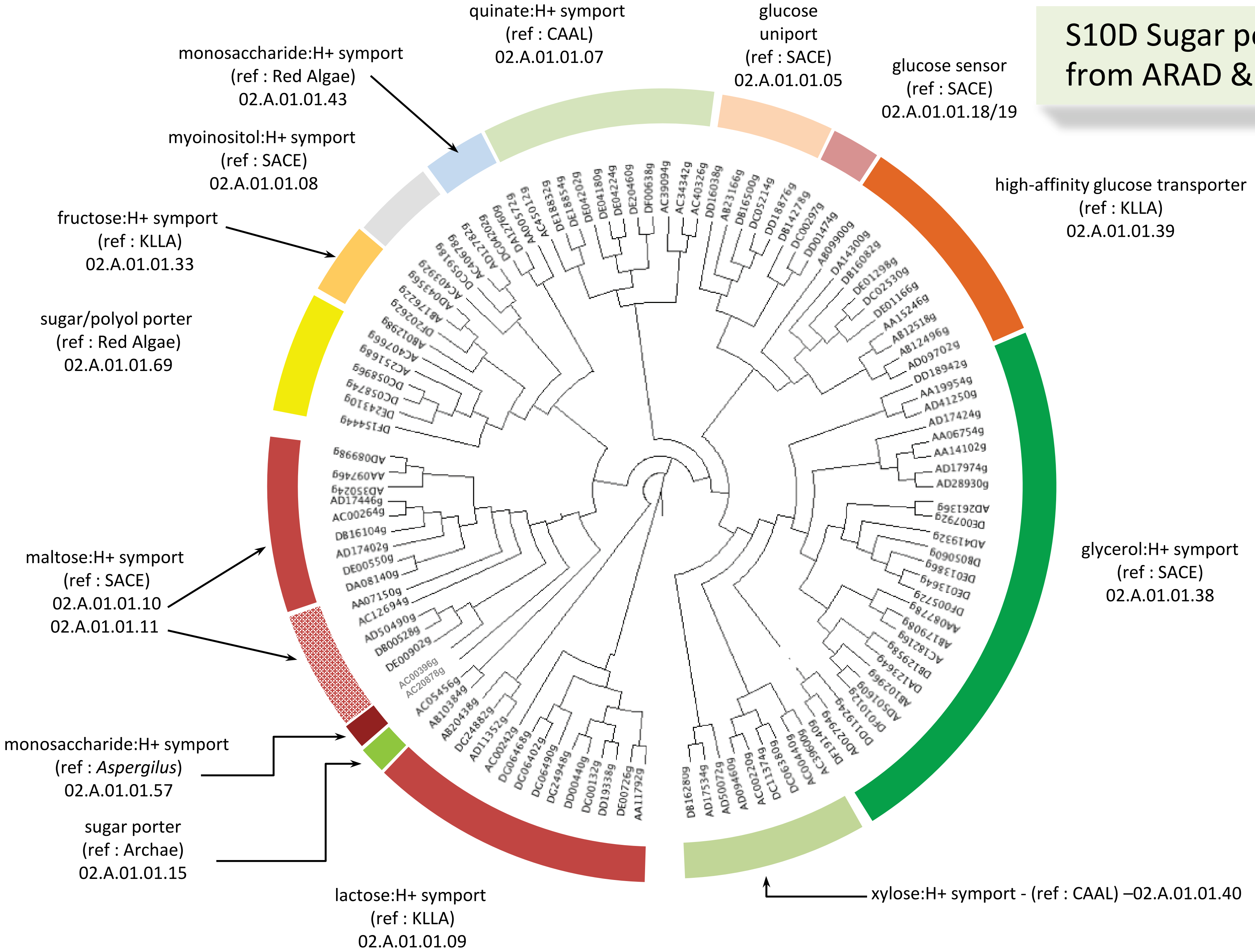

Supplement: Additional file 10 — Transporter distribution in hemiascomycete species. [file 1754-6834-7-66-S10.pdf]
