# Supplementary material for: The complete genome of Blastobotrys (Arxula) adeninivorans LS3 - a yeast of biotechnological interest
Source: Biotechnol Biofuels. 2014 Apr 24;7:66. doi: 10.1186/1754-6834-7-66 (PMC4022394; doi:10.1186/1754-6834-7-66)

Additional File 11. Tandem gene arrays in *A. adeninivorans.*

Table S11A List of tandem gene repeats in *A. adeninivorans.*

Tandem gene arrays (TGAs) were defined as genes of a given family separated by 0 to 10 intervening genes. After manual curation, 213 genes (including 12 pseudogenes) were assigned to 96 TGAs of one to six genes (see Table S11), most of the TGAs being in direct orientation (71.9%) as in other hemiascomycete yeasts. Compared to the whole set of CDSs, metabolic activities are over-represented among genes in TGAs (56% vs. 29%), as well as pseudogenes (5.6%, vs. 0.6%), (Table S11). Intron-containing genes on the contrary are under-represented in TGAs with seven genes only (3.3% vs. 10.6% for the whole gene set). A total of 62 tandem arrays without intervening gene were observed among TGAs. They involved 134 genes, 51 in direct repeats of up to 6 genes, 11 as inverted repeats.

TGA formation appears as a major mechanism for gene amplification of specific families of *A. adeninivorans*: all 8 members of the GL3C080 family, encoding a conserved NADPH oxidoreductase (Old Yellow Enzyme or Oye), occur in two uninterrupted tandems of 6 and 2 genes.

Figure S11B Examples of TGA duplication.

In the case of the two *A. adeninivorans* specific gene families GL3C4746 and GL3C4726, all members but one occur in direct pairs scattered on the same chromosome (GL3C4726) or on different chromosomes (GL3C4746). Blastn searches indicated that all tandems are highly conserved at the nucleotide level, both in coding and intergenic regions (Figure S11A1 and S11A2). It also suggested that single members in each family were likely part of an initial tandem from which the second member has been lost. Phylogenetic trees are compatible with a propagation of an ancestor tandem by successive replication, leading to independent differentiation of the two members of the pair in two well separated sub-families.

S11A1: GL3C4746

S11A2: GL3C4726

Figure S11C: Phylogeny of TGAs associated to GLC4705 inverted repeats.

A somewhat similar scenario may account for the formation of intermingled TGAs of members of the *A. adeninivorans* specific GL3C-4702, -4704, -4705, -4708 -4718 and -4726 families (main text and Figure 2). Eighteen out of the 25 members (including five pseudogenes) of the GL3C4705 family are arranged in a head-to-head orientation within seven blocks (numbered 1 to 7 as on Fig. 2) resulting from segmental duplications of an ancestral inverted repeat of two GL3C4705 members flanked by single copies of members of the GL3C4702, GL3C4708 and/or GL3C4704 families. Members of the GL3C4704 and GL3C4708 cluster together (see Fig S11B1 and S11B2) as expected if they derive from a unique initial capture event that was subsequently replicated along with the GL3C4705 pair. Such a trend is less obvious in the case of the members of the GL3C4702 families (Figure S11B3), which suggests that several capture events occurred in this case. Circled figures refer to associated GLC4705 inverted repeats on Figure 2.

S11C1: GL3C4704

S11C2: GL3C4708

S11C3: GL3C4702

Table S11A List of tandem gene repeats in *A. adeninivorans.*

Family	Members w/o pseudos	Gene	Pseudo	Position	Length	Type	Annotation
GL3C0002	56	ARAD1B12496g		1010029..1011603	1575	DR2	Plasma membrane glucose sensor
GL3C0002	56	ARAD1B12518g		1013670..1015286	1617	DR2	Plasma membrane glucose sensor
GL3C0002	56	ARAD1C00220g		9484..11154	1671	DR3	Glycerol proton symporter
GL3C0002	56	ARAD1C00242g		11589..13118	1530	DR3	Hexose transporter
GL3C0002	56	ARAD1C00264g		13900..15540	1641	DR3	Maltose permease
GL3C0002	56	ARAD1C00396g		24361..25938	1578	IR2 (1+X+1)	Hexose transporter
GL3C0002	56	ARAD1C00440g		29468..31243	1776	IR2 (1+X+1)	Plasma membrane glucose sensor
GL3C0002	56	ARAD1C40326g		3359592..3361145	1554	IR2 (1+X+1)	Galactose permease
GL3C0002	56	ARAD1C40392g		3364744..3366219	1476	IR2 (1+X+1)	Myo inositol transporter
GL3C0002	56	ARAD1C40678g		3389670..3391277	1608	DR2 (1+3X+1)	Myo inositol transporter
GL3C0002	56	ARAD1C40766g		3400238..3402127	1890	DR2 (1+3X+1)	Myo inositol transporter
GL3C0002	56	ARAD1D18062g		1479034..1481052	2019	IR4 (DR2 + DR2(1+3X+1))	High affinity maltose transporter
GL3C0002	56	ARAD1D18084g		1481646..1483184	1539	IR4 DR2 + DR2(1+3X+1)	Glycerol proton symporter
GL3C0002	56	ARAD1D18106g		1483233..1484864	1632	IR4 DR2 + DR2(1+3X+1)	Maltose permease
GL3C0002	56	ARAD1D18194g		1490126..1491718	1593	IR4 DR2 + DR2(1+3X+1)	Hexose transporter
GL3C0003	25	ARAD1A18612g		1546204..1546821	618	DR2 (1+10X+1)	Small GTPase
GL3C0003	25	ARAD1A18854g		1562290..1562937	648	DR2 (1+10X+1)	Small GTPase
GL3C0005	27	ARAD1A09570g		789415..790500	1086	DR2	Xylitol dehydrogenase
GL3C0005	27	ARAD1A09592g		790731..791765	1035	DR2	Xylitol dehydrogenase
GL3C0005	27	ARAD1D13794g		1120221..1121246	1026	IR2 (1+9X+1)	Conserved hypothetical protein, unknown function
GL3C0005	27	ARAD1D14014g		1140651..1141676	1026	IR2 (1+9X+1)	Conserved hypothetical protein, unknown function
GL3C0006	26	ARAD1C19206g		1573554..1575074	1521	DR2 (1+X+1)	Nitrate transporter
GL3C0006	26	ARAD1C19250g		1576032..1577546	1515	DR2 (1+X+1)	Nitrate transporter
GL3C0006	26	ARAD1C20174g		1671042..1672535	1494	DR2 (1+X+1)	High affinity nicotinic acid plasma membrane permease
GL3C0006	26	ARAD1C20218g		1674171..1675700	1530	DR2 (1+X+1)	Allantoin permease
GL3C0007	32	ARAD1D12672g		1027635..1029350	1716	DR2	Multidrug transporter of the MFS superfamily
GL3C0007	32	ARAD1D12694g		1030244..1031947	1704	DR2	Multidrug transporter of the MFS superfamily
GL3C0007	32	ARAD1D18524g		1517667..1519136	1470	DR2	Polyamine transport protein
GL3C0007	32	ARAD1D18546g		1519739..1521220	1482	DR2	Polyamine transport protein
GL3C0008	42	ARAD1B01562g		142849..143607	759	DR2 (1+8X+1)	2 4 dienoyl CoA reductase
GL3C0008	42	ARAD1B01848g		167100..167858	759	DR2 (1+8X+1)	2 4 dienoyl CoA reductase
GL3C0008	42	ARAD1C26686g		2226895..2227800	906	DR2	Acyl dihydroxyacetone phosphate reductase
GL3C0008	42	ARAD1C26708g		2228068..2228913	846	DR2	Acyl dihydroxyacetone phosphate reductase
GL3C0008	42	ARAD1C44506g		3718105..3718938	834	DR2 (1+8X+1)	Conserved hypothetical protein, unknown function
GL3C0008	42	ARAD1C44704g		3737316..3738074	759	DR2 (1+8X+1)	Conserved hypothetical protein, unknown function
GL3C0008	42	ARAD1D07590g		603358..604059	702	IR2 (1+8X+1)	2 4 dienoyl CoA reductase
GL3C0008	42	ARAD1D07788g		620279..621088	810	IR2 (1+8X+1)	2 4 dienoyl CoA reductase
GL3C0008	42	ARAD1D18942g		1556547..1557383	837	DR2 (1+X+1)	Putative short-chain dehydrogenase/reductase
GL3C0008	42	ARAD1D19030g		1559002..1559763	762	DR2 (1+X+1)	Putative short-chain dehydrogenase/reductase
GL3C0009	13	ARAD1B22814g		1869154..1870845	1692	DR2	Putative transporter of the Major Facilitator Superfamily MFS
GL3C0009	13	ARAD1B22836g		1871167..1872885	1719	DR2	Putative transporter of the Major Facilitator Superfamily MFS
GL3C0010	13	ARAD1C01474g		123867..124199	333	DR2	Ubiquitin conjugating enzyme E2
GL3C0010	13	ARAD1C01496g		124495..124938	444	DR2	Ubiquitin conjugating enzyme E2
GL3C0012	24	ARAD1B21824g		1801105..1802539	1435	IR2 (1+6X+1)	Aspartyl protease
GL3C0012	24	ARAD1B22000g		1813801..1815009	1209	IR2 (1+6X+1)	Aspartyl protease
GL3C0012	24	ARAD1C13772g		1130957..1132483	1527	DR4	SAP like aspartic protease, GPI-anchored
GL3C0012	24	ARAD1C13794g		1133509..1135170	1662	DR4	SAP like aspartic protease, GPI-anchored
GL3C0012	24	ARAD1C13816g		1136120..1137679	1560	DR4	SAP like aspartic protease, GPI-anchored
GL3C0012	24	ARAD1C13838g		1138784..1140442	1659	DR4	SAP like aspartic protease, GPI-anchored

GL3C0012	24	ARAD1D08514g		687271..688398	1128	DR2	GPI-anchored aspartic protease
GL3C0012	24	ARAD1D08536g		689394..692858	3465	DR2	GPI-anchored aspartic protease
GL3C0012	24	ARAD1D47014g		3929151..3930833	1683	DR2 (1+7X+1)	GPI anchored aspartyl protease
GL3C0012	24	ARAD1D47190g		3945714..3947150	1437	DR2 (1+7X+1)	GPI anchored aspartyl protease
GL3C0015	9	ARAD1D07040g		571441..573108	1668	DR2 (1+6X+1)	Subunit of the cytosolic chaperonin Cct ring complex
GL3C0015	9	ARAD1D07194g		581386..583056	1671	DR2 (1+6X+1)	Subunit of the cytosolic chaperonin Cct ring complex
GL3C0024	12	ARAD1A01694g		123435..123950	516	IR2 (1+7X+1)	Peptidyl prolyl cis trans isomerase
GL3C0024	12	ARAD1A01870g		133643..134353	711	IR2 (1+7X+1)	Peptidyl prolyl cis trans isomerase
GL3C0039	10	ARAD1A00264g		13504..15510	2007	DR2	Ferric reductase
GL3C0039	10	ARAD1A00286g		16931..18931	2001	DR2	Ferric reductase
GL3C0039	10	ARAD1D37862g		3170698..3172236	1539	IR2 (1+8X+1)	Ferric reductase
GL3C0039	10	ARAD1D38060g		3191392..3193434	2043	IR2 (1+8X+1)	Ferric reductase
GL3C0044	9	ARAD1D20878g		1718647..1720200	1554	DR2	Choline ethanolamine transporter
GL3C0044	9	ARAD1D20900g		1720521..1722086	1566	DR2	Choline ethanolamine transporter
GL3C0055	5	ARAD1B01584g	Yes	143859..144908	1050	DR2 (1+6+1)	putative hscarg dehydrogenase (relic)
GL3C0055	5	ARAD1B01672g	Yes	151204..152115	912	DR2 (1+6X+1)	methylglyoxal reductase (relic)
GL3C0063	5	ARAD1C17226g		1409413..1410969	1557	IR2 (1+4X+1)	histone acetyltransferase
GL3C0063	5	ARAD1C17380g	Yes	1423946..1424177	232	IR2 (1+4X+1)	histone acetyltransferase (relic)
GL3C0074	6	ARAD1C13750g		1128807..1130297	1491	DR2 (1+4X+1)	Similar to MFS transporter
GL3C0074	6	ARAD1C13860g		1141243..1142793	1551	DR2 (1+4X+1)	Similar to MFS transporter
GL3C0080	8	ARAD1D21714g		1781907..1782998	1092	DR2	NADPH oxidoreductase containing flavin mononucleotide FMN
GL3C0080	8	ARAD1D21736g		1783243..1784343	1101	DR2	NADPH oxidoreductase containing flavin mononucleotide FMN
GL3C0080	8	ARAD1D22374g	Yes	1840341..1841489	1149	DR6	NADPH oxidoreductase containing flavin mononucleotide FMN (relic)
GL3C0080	8	ARAD1D22396g		1841914..1843035	1122	DR6	NADPH oxidoreductase containing flavin mononucleotide FMN
GL3C0080	8	ARAD1D22418g		1843761..1844885	1125	DR6	NADPH oxidoreductase containing flavin mononucleotide FMN
GL3C0080	8	ARAD1D22440g		1845356..1846450	1095	DR6	NADPH oxidoreductase containing flavin mononucleotide FMN
GL3C0080	8	ARAD1D22462g		1846946..1848070	1125	DR6	NADPH oxidoreductase containing flavin mononucleotide FMN
GL3C0080	8	ARAD1D22484g		1848343..1849428	1086	DR6	NADPH oxidoreductase containing flavin mononucleotide FMN
GL3C0086	6	ARAD1D43758g		3632030..3633574	1545	DR2	N- formyltyrosine oxidase,sporulation- specific microsomal enzyme
GL3C0086	6	ARAD1D43780g		3634648..3636231	1584	DR2	N- formyltyrosine oxidase,sporulation- specific microsomal enzyme
GL3C0094	4	ARAD1C33308g		2777118..2777432	315	DR2 (1+10X+1)	Glutaredoxin thioltransferase
GL3C0094	4	ARAD1C33550g		2797163..2797511	349	DR2 (1+10X+1)	Glutaredoxin thioltransferase
GL3C0107	7	ARAD1A07194g		591376..593037	1662	DR2	Putative amidase
GL3C0107	7	ARAD1A07216g		593246..594907	1662	DR2	Putative amidase
GL3C0123	3	ARAD1A01452g		104941..106803	1863	IR2	Dihydrouridine synthase
GL3C0123	3	ARAD1A01474g		106911..107936	1026	IR2	Dihydrouridine synthase
GL3C0176	20	ARAD1A00462g		29097..31637	2541	IR2 (1+9X+1)	Conserved hypothetical protein, unknown function
GL3C0176	20	ARAD1A00682g		47090..48736	1647	IR2 (1+9X+1)	Conserved hypothetical protein, unknown function
GL3C0407	6	ARAD1B10340g		842152..843504	1353	DR2 (1+8X+1)	Hypothetical protein, unknown function
GL3C0407	6	ARAD1B10560g		858636..861053	2418	DR2 (1+8X+1)	Hypothetical protein, unknown function
GL3C0501	5	ARAD1C26334g		2201049..2201870	822	IR2	Enoyl-CoA hydratase/isomerase family protein
GL3C0501	5	ARAD1C26356g		2202271..2203098	828	IR2	Enoyl-CoA hydratase/isomerase family protein
GL3C0521	2	ARAD1B14212g		1149074..1149772	699	DR2 (1+X+1)	Putative histone acetylase

GL3C0521	2	ARAD1B14278g		1152399..1153916	1518	DR2 (1+X+1)	Putative histone acetylase
GL3C0522	7	ARAD1B09240g		761755..762774	1020	DR3	Putative aryl alcohol dehydrogenase
GL3C0522	7	ARAD1B09262g		763065..764081	1017	DR3	Putative aryl alcohol dehydrogenase
GL3C0522	7	ARAD1B09284g		764260..765342	1083	DR3	Putative aryl alcohol dehydrogenase
GL3C3548	3	ARAD1D04158g		340149..340724	576	DR2	Protein with eight cysteine-containing domain present in fungal extracellular membrane proteins
GL3C3548	3	ARAD1D04180g		341548..342282	735	DR2	Protein with eight cysteine-containing domain present in fungal extracellular membrane proteins
GL3C3859	3	ARAD1A05390g		437471..439111	1641	DR2 (1+6X+1)	Conserved hypothetical protein, unknown function
GL3C3859	3	ARAD1A05544g		448539..450179	1641	DR2 (1+6X+1)	Conserved hypothetical protein, unknown function
GL3C4063	2	ARAD1B03916g		324115..325497	1383	DR2	Alcohol acetyltransferase
GL3C4063	2	ARAD1B03938g		325616..327064	1449	DR2	Alcohol acetyltransferase
GL3C4088	8	ARAD1C00308g		17305..19323	2019	IR2 (1+4X+1)	Putative C6 transcription factor
GL3C4088	8	ARAD1C00418g		26384..28441	2058	IR2 (1+4X+1)	Putative C6 transcription factor
GL3C4410	2	ARAD1C38104g		3164581..3169670	5090	IR2	Chitin synthase III
GL3C4410	2	ARAD1C38126g		3170823..3176087	5265	IR2	Chitin synthase III
GL3C4702	25	ARAD1A12430g		1041785..1043857	2073	DR2	Multifunctional thiamine-phosphate pyrophosphorylase/synthase
GL3C4702	25	ARAD1A12452g		1044731..1046716	1986	DR2	Multifunctional thiamine-phosphate pyrophosphorylase
GL3C4702	25	ARAD1B03388g		279108..280328	1221	IR2 (1+8X+1)	Hypothetical protein, unknown function
GL3C4702	25	ARAD1B03586g		296108..298180	2073	IR2 (1+8X+1)	Hypothetical protein, unknown function
GL3C4702	25	ARAD1C13442g		1097971..1100043	2073	DR2	Hypothetical protein, unknown function
GL3C4702	25	ARAD1C13464g		1100892..1102964	2073	DR2	Hypothetical protein, unknown function
GL3C4702	25	ARAD1D12232g		986690..988477	1788	DR2(1+4X+1)	Hypothetical protein, unknown function
GL3C4702	25	ARAD1D12342g	Yes	999172..1001239	2068	DR2(1+4X+1)	Hypothetical protein, unknown function (relic)
GL3C4702	25	ARAD1D27764g	Yes	2297997..2298850	854	DR2 (1+X+1)	Hypothetical protein, unknown function (relic)
GL3C4702	25	ARAD1D27808g		2301977..2303764	1788	DR2 (1+X+1)	Hypothetical protein, unknown function
GL3C4704	22	ARAD1C18502g		1515164..1517233	2070	DR2	Hypothetical protein, unknown function
GL3C4704	22	ARAD1C18524g		1517616..1519688	2073	DR2	Hypothetical protein, unknown function
GL3C4704	22	ARAD1C19778g		1623913..1626642	2730	IR2 (1+5X+1)	Hypothetical protein, unknown function
GL3C4704	22	ARAD1C19910g		1637725..1640106	2382	IR2 (1+5X+1)	Hypothetical protein, unknown function
GL3C4705	20	ARAD1A11286g		941679..943868	2190	IR3 (IR2+X+1)	Hypothetical protein, unknown function
GL3C4705	20	ARAD1A11308g		944641..946608	1968	IR3 (IR2+X+1)	Hypothetical protein, unknown function
GL3C4705	20	ARAD1A11352g		950335..952269	1935	IR3 (IR2+X+1)	Hypothetical protein, unknown function
GL3C4705	20	ARAD1A13552g		1155647..1157578	1932	DR4 (IR2+3X+IR2)	Hypothetical protein, unknown function
GL3C4705	20	ARAD1A13574g		1158372..1160564	2193	DR4 (IR2+3X+IR2)	Hypothetical protein, unknown function
GL3C4705	20	ARAD1A13662g		1169536..1171347	1812	DR4 (IR2+3X+IR2)	Hypothetical protein, unknown function
GL3C4705	20	ARAD1A13684g		1171412..1173046	1635	DR4 (IR2+3X+IR2)	Hypothetical protein, unknown function
GL3C4705	20	ARAD1C07766g		631304..632938	1635	IR4 (IR2 + X+DR2)	Hypothetical protein, unknown function
GL3C4705	20	ARAD1C07788g		634017..636209	2193	IR4 (IR2 + X+DR2)	Hypothetical protein, unknown function
GL3C4705	20	ARAD1C07854g	Yes	642015..643918	1904	IR4 (IR2 + X+DR2)	Hypothetical protein, unknown function (relic)
GL3C4705	20	ARAD1C07876g		644415..646226	1812	IR4 (IR2 + X+DR2)	Hypothetical protein, unknown function
GL3C4705	20	ARAD1C22572g		1902254..1903888	1635	IR2	Hypothetical protein, unknown function
GL3C4705	20	ARAD1C22594g	Yes	1904238..1905938	1701	IR2	Hypothetical protein, unknown function (relic)
GL3C4705	20	ARAD1D04928g	Yes	393569..394015	447	DR2	Hypothetical protein, unknown function (relic)
GL3C4705	20	ARAD1D04950g		395096..396823	1728	DR2	Hypothetical protein, unknown function
GL3C4705	20	ARAD1D12210g		984454..986157	1704	IR3 (1+X+IR2)	Hypothetical protein, unknown function
GL3C4705	20	ARAD1D12254g		989601..991265	1665	IR3 (1+X+IR2)	Hypothetical protein, unknown function
GL3C4705	20	ARAD1D12276g	Yes	991597..993296	1700	IR3 (1+X+IR2)	Hypothetical protein, unknown function (relic)
GL3C4705	20	ARAD1D27786g		2299240..2301159	1920	DR2 (1+X+1)	Hypothetical protein, unknown function
GL3C4705	20	ARAD1D27830g		2304724..2306415	1692	DR2 (1+X+1)	Hypothetical protein, unknown function
GL3C4705	20	ARAD1D49192g		4129270..4131345	2076	IR3 (1+2X+IR2)	Hypothetical protein, unknown function
GL3C4705	20	ARAD1D49258g	Yes	4138188..4139104	917	IR3 (1+2X+IR2)	Hypothetical protein, unknown function (relic)

GL3C4705	20	ARAD1D49280g		4140220..4142154	1935	IR3 (1+2X+IR2)	Hypothetical protein, unknown function
GL3C4708	14	ARAD1A11022g		920145..921932	1788	DR2 (1+10X+1)	Hypothetical protein, unknown function
GL3C4708	14	ARAD1A11264g		939457..941148	1692	DR2 (1+10X+1)	Hypothetical protein, unknown function
GL3C4708	14	ARAD1C07810g		636771..638531	1761	IR2 (1+8X+1)	Hypothetical protein, unknown function
GL3C4708	14	ARAD1C08030g		656312..658300	1989	IR2 (1+8X+1)	Hypothetical protein, unknown function
GL3C4711	11	ARAD1D25366g		2102045..2103583	1539	DR3	F-box domain protein
GL3C4711	11	ARAD1D25388g	Yes	2104153..2105657	1505	DR3	F-box domain protein (relic)
GL3C4711	11	ARAD1D25432g		2106199..2107794	1596	DR3	F-box domain protein
GL3C4712	7	ARAD1D48422g		4067306..4069144	1839	DR2	Beta-glucosidase
GL3C4712	7	ARAD1D48444g		4069783..4071297	1515	DR2	Beta-glucosidase
GL3C4718	8	ARAD1C07700g		625965..627026	1062	DR2 (1+6X+1)	Hypothetical protein, unknown function
GL3C4718	8	ARAD1C07898g		646591..646977	387	DR2 (1+6X+1)	Hypothetical protein, unknown function
GL3C4725	6	ARAD1A01298g		93225..93788	564	IR3 (1+X+DR2)	Hypothetical protein, unknown function
GL3C4725	6	ARAD1A01342g		96081..96533	453	IR3 (1+X+DR2)	Hypothetical protein, unknown function
GL3C4725	6	ARAD1A01364g		97845..98480	636	IR3 (1+X+DR2)	Hypothetical protein, unknown function
GL3C4726	6	ARAD1C17292g	Yes	1414246..1416423	2178	DR2	Hypothetical protein, unknown function (relic)
GL3C4726	6	ARAD1C17314g		1416791..1419025	2235	DR2	Hypothetical protein, unknown function
GL3C4726	6	ARAD1C22308g		1878886..1881105	2220	DR2	Hypothetical protein, unknown function
GL3C4726	6	ARAD1C22330g		1881470..1883701	2232	DR2	Hypothetical protein, unknown function
GL3C4726	6	ARAD1C25762g		2156959..2159193	2235	DR2	Hypothetical protein, unknown function
GL3C4726	6	ARAD1C25784g		2159545..2161785	2241	DR2	Hypothetical protein, unknown function
GL3C4736	5	ARAD1D18304g		1500341..1501354	1014	IR3 (DR2+X+1)	Sterol deacetylase
GL3C4736	5	ARAD1D18326g		1501676..1502677	1002	IR3 (DR2+X+1)	Sterol deacetylase
GL3C4736	5	ARAD1D18370g		1504958..1505929	972	IR3 (DR2+X+1)	Sterol deacetylase
GL3C4738	4	ARAD1D46860g		3915311..3917119	1809	DR2	Hypothetical protein, unknown function
GL3C4738	4	ARAD1D46882g		3917714..3919423	1710	DR2	Hypothetical protein, unknown function
GL3C4746	5	ARAD1B18480g		1523422..1525737	2316	DR2	Hypothetical protein, unknown function
GL3C4746	5	ARAD1B18502g		1526336..1528672	2337	DR2	Hypothetical protein, unknown function
GL3C4746	5	ARAD1C21164g		1767445..1769802	2358	DR2	Hypothetical protein, unknown function
GL3C4746	5	ARAD1C21186g		1770393..1772729	2337	DR2	Hypothetical protein, unknown function
GL3C4765	3	ARAD1B19426g		1599403..1600500	1098	DR2	Putative GPI anchored dioxygenase
GL3C4765	3	ARAD1B19448g		1601067..1602170	1104	DR2	Putative GPI anchored dioxygenase
GL3C4766	4	ARAD1D46486g		3880691..3882097	1407	DR2	Hypothetical protein, unknown function
GL3C4766	4	ARAD1D46508g		3884442..3886001	1560	DR2	Hypothetical protein, unknown function
GL3C4767	4	ARAD1D01650g		135071..137149	2079	DR3	Putative C6 transcription factor
GL3C4767	4	ARAD1D01672g		137746..140199	2454	DR3	Putative C6 transcription factor
GL3C4767	4	ARAD1D01694g		140493..142379	1887	DR3	Putative C6 transcription factor
GL3C4808	2	ARAD1B05038g		419140..419874	735	DR2	Hypothetical protein, unknown function
GL3C4808	2	ARAD1B05060g		420354..421160	807	DR2	Hypothetical protein, unknown function
GL3C4812	2	ARAD1D51128g		4276992..4278545	1554	DR2	Putative cellulose-binding protein
GL3C4812	2	ARAD1D51150g		4278967..4280409	1443	DR2	Putative cellulose-binding protein
GL3C4932	3	ARAD1B06842g		550662..551336	675	DR2	Putative cutinase
GL3C4932	3	ARAD1B06864g		552289..552957	669	DR2	Putative cutinase
GL3C5307	2	ARAD1A09614g		792129..793007	879	DR2	Similarity to DTDP glucose 4 6 dehydratase
GL3C5307	2	ARAD1A09636g		793751..794629	879	DR2	Similarity to DTDP glucose 4 6 dehydratase
GL3C5672	1	ARAD1D47542g	Yes	3981064..3981841	778	DR2	Hypothetical protein, unknown function (relic)
GL3C5672	1	ARAD1D47564g		3983547..3988301	4755	DR2	Hypothetical protein, unknown function
GL3M4593	5	ARAD1C29480g		2450287..2451387	1101	DR2 (1+6X+1)	Catalytic subunit of protein phosphatase 2A
GL3M4593	5	ARAD1C29634g		2460259..2461323	1065	DR2 (1+6X+1)	Catalytic subunit of protein phosphatase 2A
GL3R0071	5	ARAD1D35662g		2964804..2966198	1395	DR2	Vacuolar carboxypeptidase Y proteinase C
GL3R0071	5	ARAD1D35684g		2966801..2968492	1692	DR2	Vacuolar carboxypeptidase Y proteinase C
GL3R0174	5	ARAD1A10274g		846495..847850	1356	IR2 (1+X+1)	Putative mannosidase GPI anchored membrane

						protein
GL3R0174	5	ARAD1A10318g	850638..851990	1353	IR2 (1+X+1)	Putative mannosidase GPI anchored membrane protein
GL3R0184	3	ARAD1A02970g	239701..240933	1233	DR2	High affinity iron permease
GL3R0184	3	ARAD1A02992g	241978..243129	1152	DR2	High affinity iron permease
GL3R0201	4	ARAD1D26268g	2165894..2167051	1158	DR2	High affinity zinc transporter
GL3R0201	4	ARAD1D26290g	2167529..2168662	1134	DR2	High affinity zinc transporter
GL3R0789	2	ARAD1D23936g	1989698..1990699	1002	DR2	Conserved hypothetical protein, unknown function
GL3R0789	2	ARAD1D23958g	1990888..1991952	1065	DR2	Conserved hypothetical protein, unknown function
GL3R0923	2	ARAD1C20944g	1740353..1741816	1464	DR2	Invertase
GL3R0923	2	ARAD1C20966g	1742117..1743643	1527	DR2	Invertase
GL3R1212	2	ARAD1D30954g	2549622..2550860	1239	IR2 (1+8X+1)	D serine dehydratase
GL3R1212	2	ARAD1D31152g	2573773..2575167	1395	IR2 (1+8X+1)	D serine dehydratase
GL3R1449	2	ARAD1D01760g	146185..146617	433	IR2 (1+2X+1)	Vacuolar H ATPase subunit G
GL3R1449	2	ARAD1D01826g	149671..150064	394	IR2 (1+2X+1)	Vacuolar H ATPase subunit G
GL3R1456	2	ARAD1D07436g	595366..595884	519	DR2	Zinc finger domain protein similar to Type I J proteins
GL3R1456	2	ARAD1D07458g	596240..597079	840	DR2	Zinc finger domain protein similar to Type I J proteins
GL3R1547	2	ARAD1A11814g	987118..990120	3003	DR2	Phenylcoumaran benzylic ether reductase (PCBER) like
GL3R1547	2	ARAD1A11836g	990868..993882	3015	DR2	Phenylcoumaran benzylic ether reductase (PCBER) like
GL3R3961	2	ARAD1D12892g	1050766..1052289	1524	DR2	Putative acid phosphatase
GL3R3961	2	ARAD1D12914g	1052667..1054247	1581	DR2	Putative acid phosphatase

IR = inverted repeat; DR = direct repeat
If repeats are interrupted by intervening genes, the number of intervening genes is indicated in parenthesis as nX (example: 1+2X+1 for a repeat interrupted by two intervening genes).

Figure S11B1 GL3C4746 family

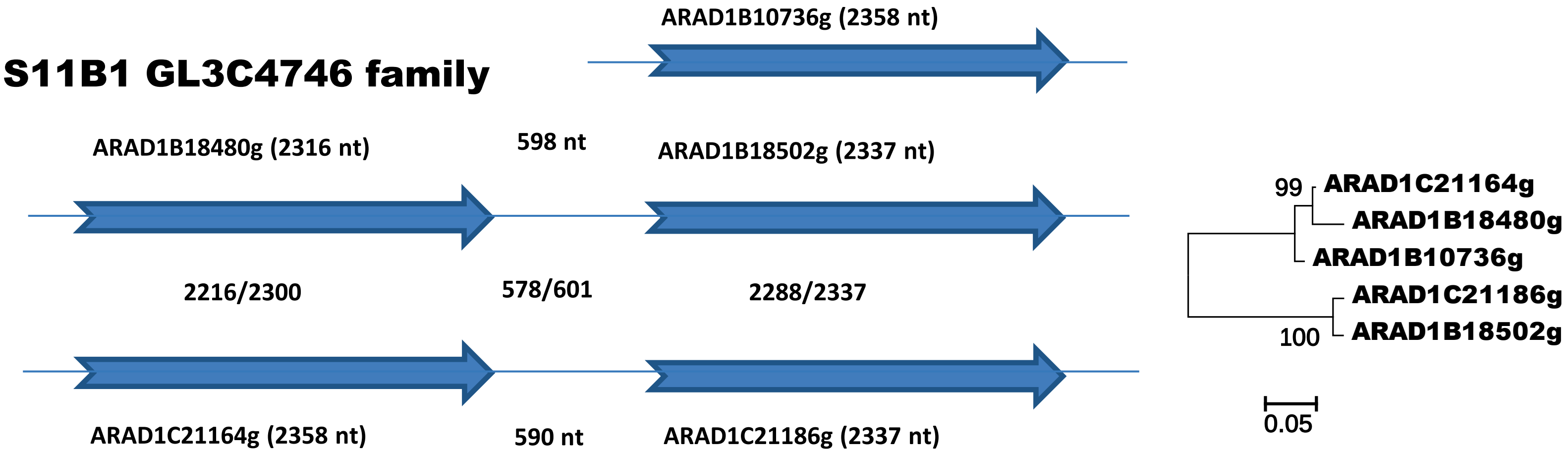

Figure S11B2 GL3C4726 family

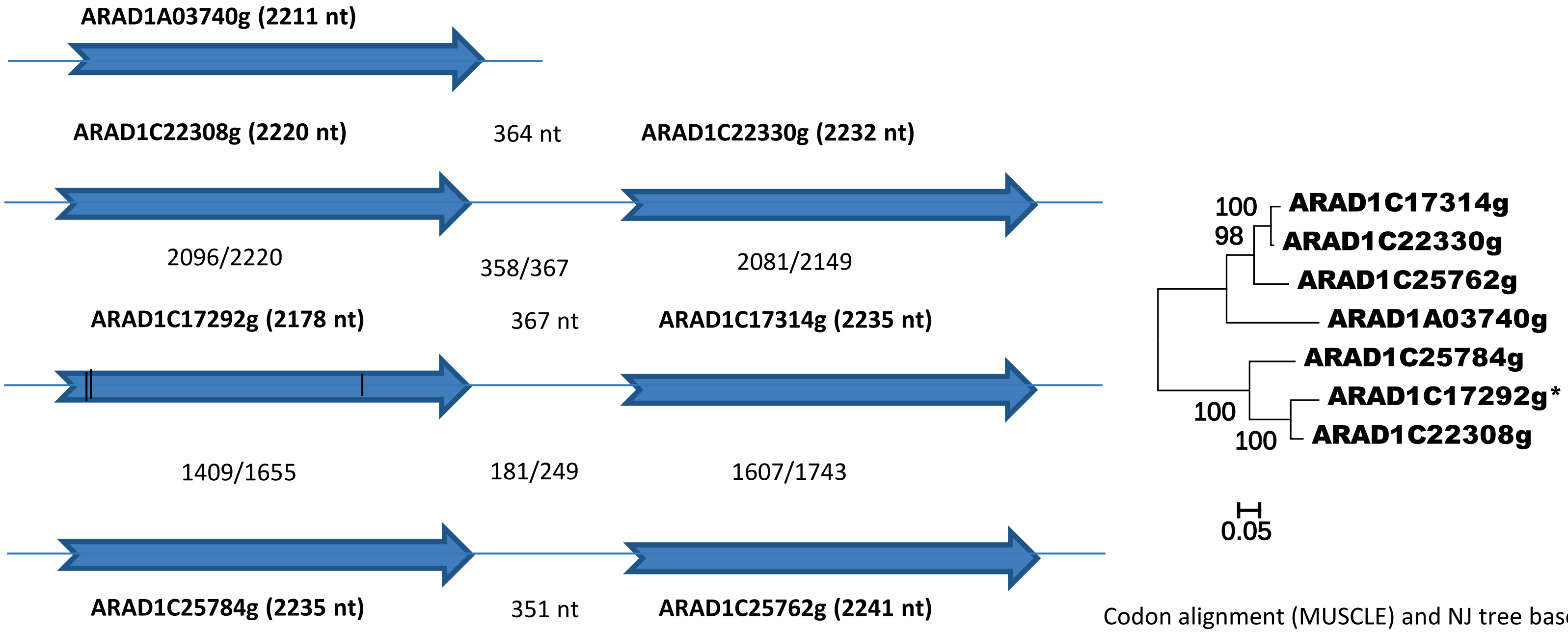

Nucleotide identity within gene and intergenes was computed based on BLASTn alignments

Codon alignment (MUSCLE) and NJ tree based on 1st and 2nd codon position. Pseudogene indicated by *.

Figure S11C1 GL3C4704 family

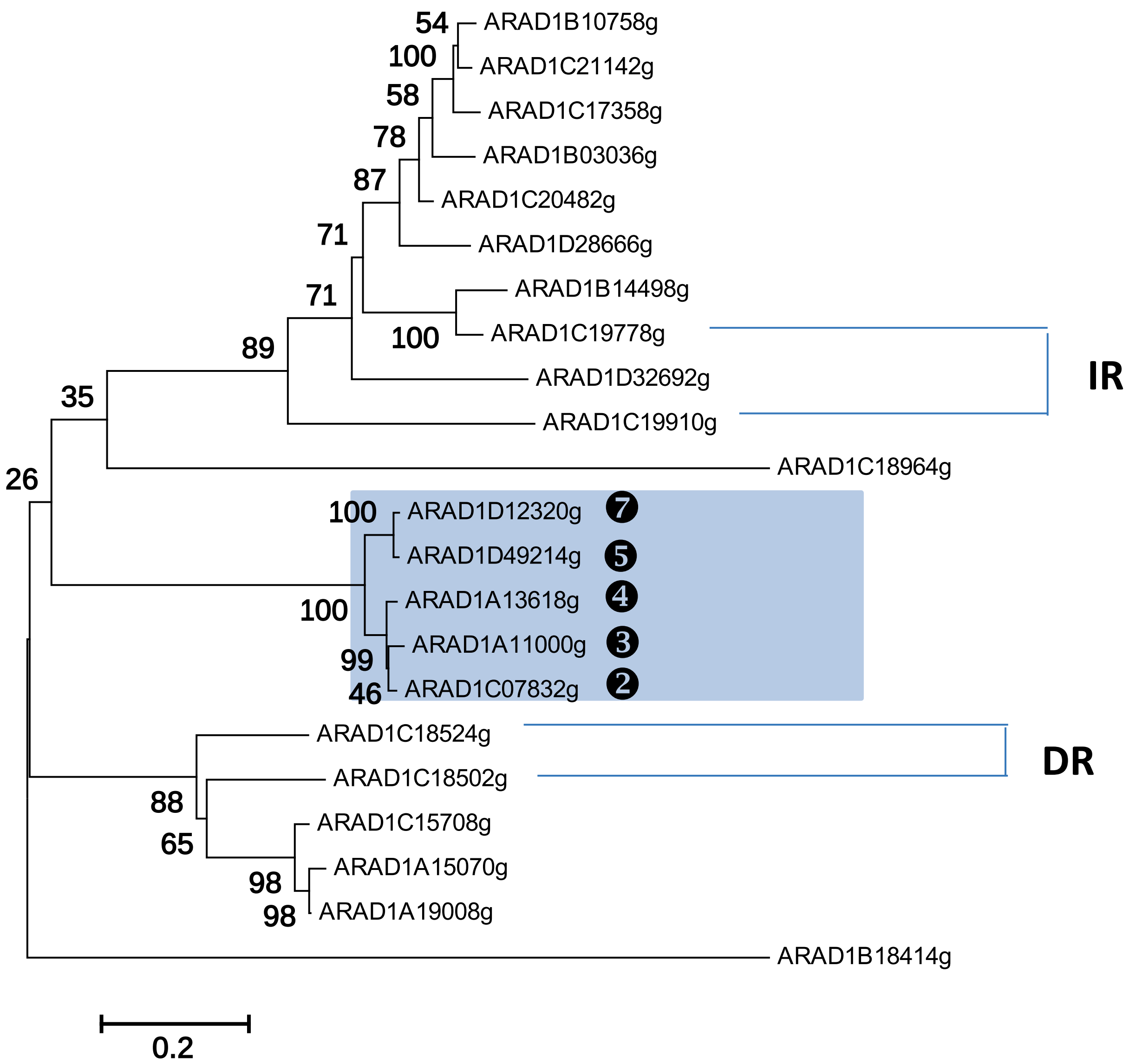

Figure S11C2 GL3C4708 family

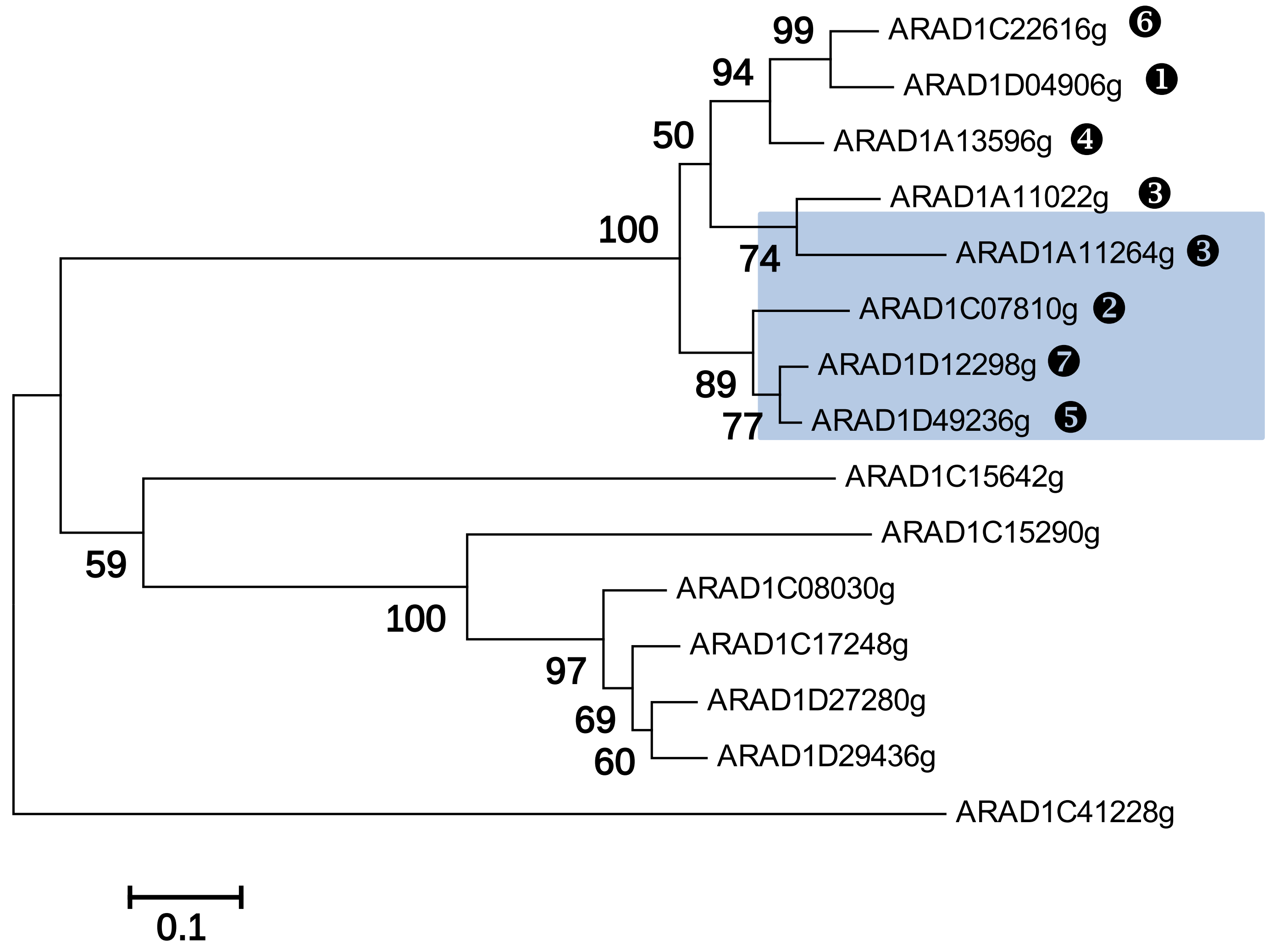

Figure S11C3 GL3C4702 family

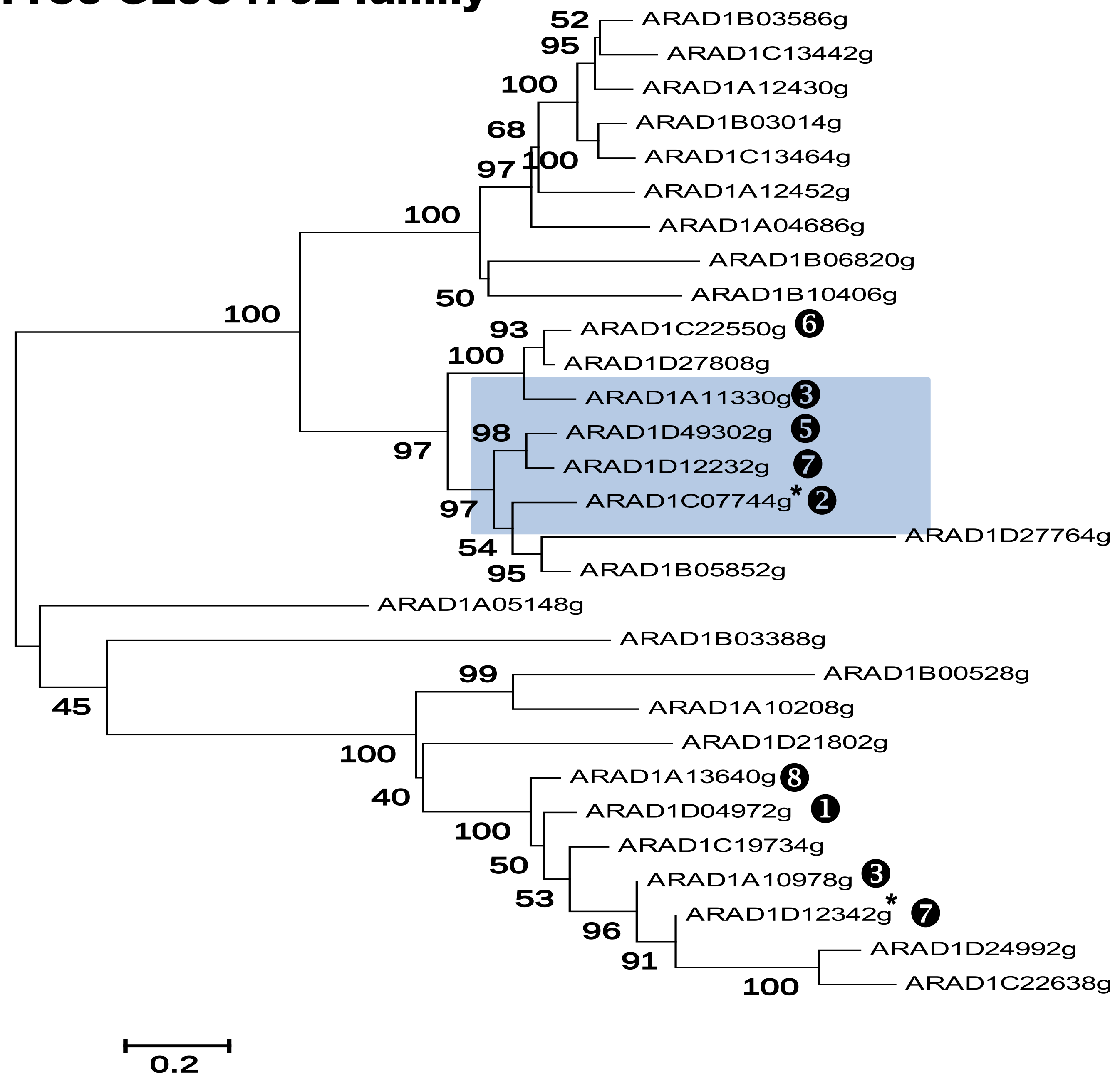

Supplement: Additional file 11 — Tandem gene arrays in A. adeninivorans. [file 1754-6834-7-66-S11.pdf]
