# Supplementary material for: The complete genome of Blastobotrys (Arxula) adeninivorans LS3 - a yeast of biotechnological interest
Source: Biotechnol Biofuels. 2014 Apr 24;7:66. doi: 10.1186/1754-6834-7-66 (PMC4022394; doi:10.1186/1754-6834-7-66)

Additional File 12. Mating type locus

Figure S12 Matalpha protein alignments.

Proteins were aligned using Multalin[17]. The Alpha box (DNA binding domain) is boxed in the *S. cerevisiae* sequence. UniProtKB references for the aligned proteins are: F2Z6B8 *Yarrowia lipolytica*, Q707Y7.1 *Ogataea angusta*, Q6BNT6 *Debaryomyces hansenii*, G8XZ51 *Millerozyma farinosa*, F1D944 *Candida orthopsilosis*, F2QXX3 *Komagataella pastoris*, C4Y9N3 *Clavispora lusitaniae*, G8ZQV3 *Torulaspora delbrueckii*, B2G3Y8 *Zygosaccharomyces rouxii*, P0CY06 *Saccharomyces cerevisiae*. The tree was built using ClustalW and visualized with NJ Plot; bootstrap values are indicated at nodes.

Figure S12 MTAL1 homologues

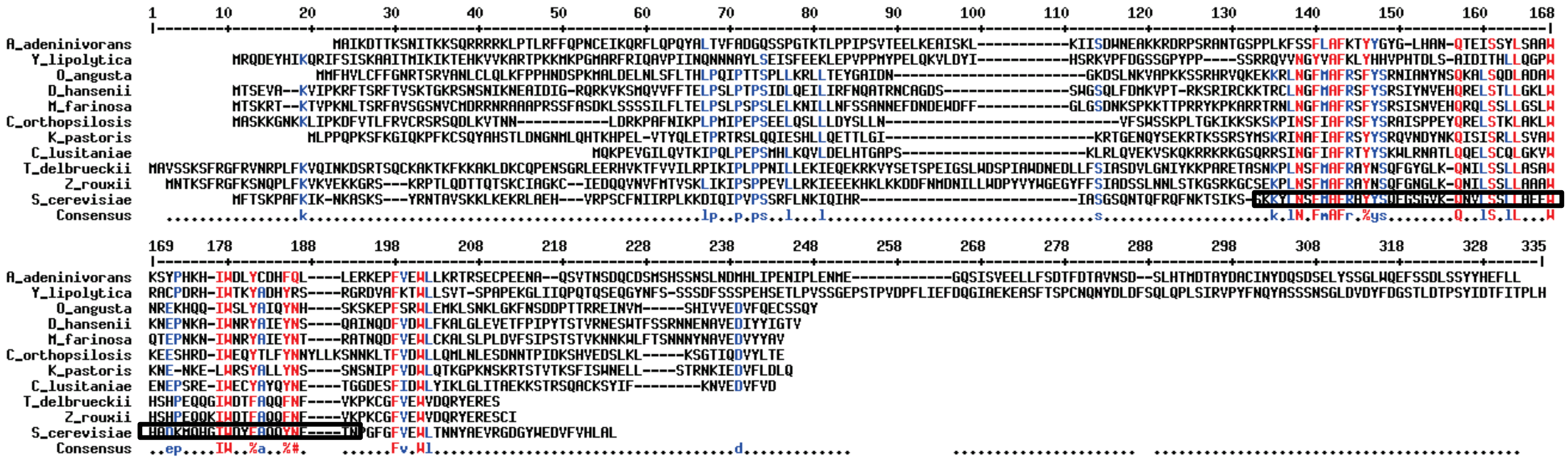

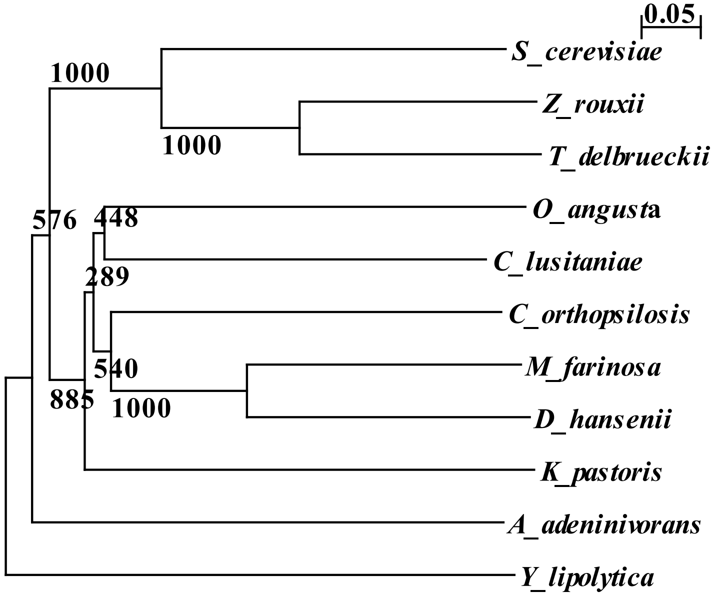

Supplement: Additional file 12 — Mating type locus. [file 1754-6834-7-66-S12.pdf]
