# Supplementary material for: The complete genome of Blastobotrys (Arxula) adeninivorans LS3 - a yeast of biotechnological interest
Source: Biotechnol Biofuels. 2014 Apr 24;7:66. doi: 10.1186/1754-6834-7-66 (PMC4022394; doi:10.1186/1754-6834-7-66)
Supplement: Additional file 13 — Homologues of genes involved in mating, meiosis and sporulation. [file 1754-6834-7-66-S13.pdf]

Additional File 13. List of homologues of genes involved in mating, meiosis and sporulation

We compiled a list of *S. cerevisiae* genes annotated as involved in mating, meioisis or sporulation in CGD, and completed it with genes previously assembled for a study of sex gene conservation in CTG yeasts[21]. Homologues of these genes were searched in the reannotated version of *D. hansenii* to facilitate comparison with CTG yeasts, in *K. pastoris, Y. lipolytica*, which are all sexual species, and *A. adeninivorans*. Only three genes are missing in *A. adeninivorans* compared to *Y. lipolytica*, namely homologues of *MER1* (meiosis-specific mRNA splicing), *NTD80* (Meiosis-specific transcription factor) and YJL160C (unknown function required for sporulation). Overall, the same gene set appears conserved in non *Saccharomycetaceae* and represents nearly 80% of the *S. cerevisiae* set. Genes involved in mating are particularly well conserved between *S. cerevisiae* and *A. adeninivorans* (48/53). Homologues of the MFα and MFa receptors are well conserved, as is the signaling cascade downstream of them and almost all genes involved in mating. Major exceptions concerned *FUS1* required for cell fusion in *S. cerevisiae*, which could not be detected in *Y. lipolytica* or in *A. adeninivorans* although this gene is conserved in CTG yeasts and in *K. pastoris*, and *KAR1* involved in karyogamy which however is also missing in *D. hansenii, P. pastoris* and *Y. lipolytica*.

On the contrary, meiosis- and sporulation-specific genes are often poorly conserved between *S. cerevisiae* and basal yeasts, and some appear to be missing. A few examples are given below.

Of the three proteins Spo11, Rec102 and Rec104 which form a complex required in *S. cerevisiae* for the initiation of recombination, only Spo11 appears conserved in non-*Saccharomycetaceae* yeasts. The Rec107-Mei4-Rec114 complex, also involved in double strand break formation and in the coordination of recombination initiation and first meiotic division, is apparently absent from non-*Saccharomycetaceae* yeasts. Sae3, which is a meiosis specific protein, forms a heterodimer with Mei5p and is proposed to be an assembly factor for Dmc1p involved in meiotic recombination: while Dmc1 homologues could be clearly identified, Sae3 and Mei5 encoding genes could not be identified in the four non-*Saccharomycetaceae* species. Gmc1 and Gmc2, both involved in meiotic progression and in synaptonemal complex assembly, could also not been identified in these species.

Several *S. cerevisiae* components such as Ady4, Sma1, Spo19, Spo20, Spo21, involved in prospore membrane assembly have apparently no clear counterpart in non *Saccharomycetaceae* species, while Spo1 was nicely conserved in all species. Osw1 and Osw2, required for the construction of the outer spore wall layers, are not found outside *Saccharomycetaceae*.

There are four MutL homologues in *S. cerevisiae* (Mlh1, Mlh2, Mlh3 and Pms1), three of which (Mlh1p-Mlh2p and Mlh1p-Mlh3p) form two heterodimers which play a major role in the meiotic cell cycle for the resolution of heteroduplexes and the repair of specific types of mismatches. Mlh2 seems to have been lost from non-*Saccharomycetaceae* yeasts. Note that the Pms1 which engages in a Mlh1-Pms1 heterodimer and plays a major role in mismatch repair but a limited role in meiotic crossing-over, is conserved in all species tested.

The Zip4 protein regulates crossover distribution by initiating synaptonemal complex formation together with Zip2, while Zip3 provides a link between recombination enzymes and synaptonemal complex proteins. The transverse filament protein Zip1 is required for normal levels of meiotic recombination and pairing between homologues. All are absent from all non-*Saccharomycetaceae* species tested. While Cdc55, Irr1, Mcd1, Mnd2, Rec8, Smc3 required for sister chromatid cohesion are all conserved, Rec8 is poorly conserved in *K. lactis* and *K. pastoris*, but not detectable in *D. hansenii, Y. lipolytica* and *A. adeninivorans* suggesting divergent evolution. Finally, Spo13, already undetectable in *K. lactis*, might be an "invention" of *Saccharomyces*.

S13 Genes involved in mating, meiosis and sporulation

Gene	S. cerevisiae	D. hansenii	P. pastoris GS115	Y. lipolytica	A. adeninivorans	GO SLIM	Comment
MFA1	YDR461W	DEHA2D10775g	No	No	No	conjugation	a factor
MFA2	YNL145W	No	No	No	No	conjugation	a factor
STE6	YKL209C	DEHA2F17226g	PAS_chr3_0858	YALI0E05973g	ARAD1D37796g	conjugation	a factor export
AXL1	YPR122W	DEHA2E02464g	PAS_chr2-2_0232	YALI0F25091g	ARAD1C18700g	conjugation	a factor maturation
RAM2	YKL019W	DEHA2F11022g	PAS_chr1-3_0192	YALI0B16126g	ARAD1D30690g	conjugation	a factor maturation
RCE1	YMR274C	DEHA2F07260g	PAS_chr4_0834	YALI0F07359g	ARAD1D17776g	conjugation	a factor maturation
STE14	YDR410C	DEHA2G05368g	PAS_chr3_0130	YALI0E22913g	ARAD1B23012g	conjugation	a factor maturation
STE23	YLR389C	DEHA2A05192g	PAS_chr1-4_0511	YALI0F25091g	ARAD1C18700g	conjugation	a factor maturation
STE24	YJR117W	DEHA2F06248g	PAS_chr2-2_0164	YALI0F11033g	ARAD1D28336g	conjugation	a factor maturation
STE3	YKL178C	DEHA2D04004g	PAS_chr3_0861	YALI0F11913g	ARAD1B23936g	conjugation	a factor receptor
MFα1	YPL187W	No	No	YALI0E16533g	No	conjugation	alpha factor
MFα2	YGL089C	DEHA2F19580g	PAS_chr2-1_0883*	YALI0E30415g	ARAD1C26928g	conjugation	alpha factor
KEX1	YGL203C	DEHA2F22352g	PAS_chr2-1_0174	YALI0B05170g	ARAD1C29150g	conjugation	alpha factor maturation
KEX2	YNL238W	DEHA2C10296g	PAS_chr2-1_0304	YALI0F13189g	ARAD1D46200g	conjugation	alpha factor maturation
STE13	YOR219C	DEHA2E17490g	PAS_chr2-2_0310	YALI0B02838g	ARAD1C38214g	conjugation	alpha factor maturation
STE2	YFL026W	DEHA2A10736g	PAS_chr4_0123	YALI0F03905g	ARAD1C29216g	conjugation	alpha factor receptor
DEP1	YAL013W	DEHA2C16192g	PAS_chr1-4_0492	YALI0F05896g	ARAD1C06710g	conjugation	mating efficiency
PRY3	YJL078C	DEHA2G01276g	PAS_chr3_0076	YALI0F21450g	ARAD1D08536g	conjugation	mating efficiency
HYM1	YKL189W	DEHA2D06974g	PAS_chr3_0313	YALI0D01199g	ARAD1C39930g	conjugation	mating efficiency
BAR1	YIL015W	DEHA2A03256g	PAS_chr4_0584	YALI0E10175g	ARAD1D23562g	conjugation	mating efficiency
BEM1	YBR200W	DEHA2E21670g	PAS_chr2-1_0548	YALI0F27643g	ARAD1C38654g	conjugation	morphogenesis
FIG1	YBR040W	DEHA2F15686g	PAS_chr1-4_0077	YALI0A14069g	ARAD1D27258g	conjugation	mating efficiency
FIG2	YCR089W	DEHA2G01276g	PAS_FragB_0067	YALI0C04136g	ARAD1D00638g	conjugation	cell fusion
FIG4	YNL325C	DEHA2E19800g	PAS_chr1-4_0456	YALI0E33099g	ARAD1A07876g	conjugation	mating efficiency
FUS1	YCL027W	DEHA2E13816g	PAS_chr4_0632	No	No	conjugation	morphogenesis
FUS2	YMR232W	DEHA2B05214g	No	YALI0F13673g	ARAD1C32560g	conjugation	karyogamy
HBT1	YDL223C	DEHA2B15334g	PAS_chr1-4_0378	YALI0F21450g	ARAD1C28820g	conjugation	mating efficiency
JEM1	YJL073W	DEHA2C09680g	PAS_chr2-2_0015	YALI0E17303g	ARAD1D24882g	conjugation	karyogamy
KAR1	YNL188W	No	No	No	No	conjugation	karyogamy
KAR2	YJL034W	DEHA2A01364g	PAS_chr2-1_0140	YALI0E13706g	ARAD1D06732g	conjugation	karyogamy

KAR3	YPR141C	DEHA2B02068g	PAS_chr4_0900	YALI0C12859g	ARAD1D29744g	conjugation	karyogamy
KAR4	YCL055W	DEHA2E22770g	PAS_chr4_0468	YALI0C17017g	ARAD1D47850g	conjugation	regulator
KAR5	YMR065W	DEHA2E21208g	**No**	YALI0D12947g	ARAD1D09152g	conjugation	karyogamy
KAR9	YPL269W	DEHA2D05016g	PAS_chr2-1_0869	YALI0E00792g	ARAD1C13354g	conjugation	karyogamy
KDX1	YKL161C	DEHA2E18348g	PAS_chr3_0895	YALI0B02816g	ARAD1C38236g	conjugation	MAP kinase signaling cascade
MDY2	YOL111C	DEHA2E11462g	PAS_chr2-1_0076	YALI0B17061g	ARAD1C37972g	conjugation	morphogenesis
MPS3	YJL019W	DEHA2G17842g	PAS_chr1-1_0400	YALI0B11770g	ARAD1A13860g	conjugation	karyogamy
OPY2	YPR075C	DEHA2F03982g	PAS_chr3_0637	YALI0A07601g	ARAD1D22726g	conjugation	MAP kinase signaling cascade
PRM1	YNL279W	DEHA2E13948g	PAS_chr1-1_0332	YALI0E08580g	ARAD1D05522g	conjugation	cell fusion
PRM10	YJL108C	DEHA2C03278g	PAS_chr1-3_0044	YALI0E24651g	ARAD1B20878g	conjugation	mating efficiency
PRM4	YPL156C	DEHA2E13508g	PAS_chr2-1_0585	YALI0F28127g	ARAD1C07502g	conjugation	mating efficiency
PRM6	YML047C	DEHA2F16126g	PAS_chr2-1_0566	YALI0E34199g	ARAD1C11704g	conjugation	mating efficiency
PTC1	YDL006W	DEHA2C02816g	PAS_chr4_0986	YALI0D22066g	ARAD1B20460g	conjugation	MAP kinase signaling cascade
RRI2	YOL117W	**No**	**No**	**No**	**No**	conjugation	mating efficiency
SCW10	YMR305C	DEHA2C12980g	PAS_chr1-3_0229	YALI0D20680g	ARAD1B07854g	conjugation	mating efficiency
SCW4	YGR279C	DEHA2C12980g	PAS_chr1-3_0229	YALI0D20680g	ARAD1B07854g	conjugation	cell fusion
STE11	YLR362W	DEHA2B05016g	PAS_chr3_0469	YALI0F13629g	ARAD1B23232g	conjugation	MAP kinase signaling cascade
STE12	YHR084W	DEHA2F25894g	PAS_chr4_0937	YALI0E16236g	ARAD1C10714g	conjugation	MAP kinase signaling cascade
STE18	YJR086W	DEHA2G24024g	PAS_chr4_0766	YALI0A06699g	ARAD1C15334g	conjugation	MAP kinase signaling cascade
STE20	YHL007C	DEHA2E22220g	PAS_c131_0004	YALI0F00572g	ARAD1C35046g	conjugation	MAP kinase signaling cascade
STE4	YOR212W	DEHA2C16368g	PAS_chr1-1_0201	YALI0E01364g	ARAD1C24838g	conjugation	MAP kinase signaling cascade
STE5	YDR103W	DEHA2G12826g	PAS_chr1-4_0438	YALI0F20680g	ARAD1A10494g	conjugation	MAP kinase signaling cascade
STE50	YCL032W	DEHA2D17490g	PAS_chr1-1_0348	YALI0E19228g	ARAD1D28622g	conjugation	MAP kinase signaling cascade
STE7	YDL159W	DEHA2F14498g	PAS_chr1-4_0008	YALI0B13178g	ARAD1C26026g	conjugation	MAP kinase signaling cascade
CDC55	YGL190C	DEHA2B04708g	PAS_chr2-1_0831	YALI0D14080g	ARAD1C16544g	meiotic cell cycle	chromatid cohesion
IRR1	YIL026C	DEHA2D04488g	PAS_chr3_0540	YALI0F19382g	ARAD1C02992g	meiotic cell cycle	chromatid cohesion
MCD1	YDL003W	DEHA2A14058g	PAS_chr4_0641	YALI0B08470g	ARAD1B16060g	meiotic cell cycle	chromatid cohesion
MND2	YIR025W	DEHA2B01342g	PAS_chr1-3_0304	YALI0F27819g	ARAD1C20064g	meiotic cell cycle	chromatid cohesion
REC8	YPR007C	DEHA2D07106g	PAS_chr3_1166	**No**	**No**	meiotic cell cycle	chromatid cohesion
SMC3	YJL074C	DEHA2G01606g	PAS_chr2-2_0306	YALI0A00616g	ARAD1D32186g	meiotic cell cycle	chromatid cohesion
SPO13	YHR014W	**No**	**No**	**No**	**No**	meiotic cell cycle	chromatid cohesion
HED1	YDR014W-A	**No**	**No**	**No**	**No**	meiotic cell cycle	Meiotic recombination
HFM1	YGL251C	DEHA2F07986g	PAS_chr1-1_0269	YALI0B13904g	ARAD1C39490g	meiotic cell cycle	Meiotic recombination

HOP2	YGL033W	DEHA2D12210g	No	No	No	meiotic cell cycle	Meiotic recombination
MEI4	YER044C-A	No	No	No	No	meiotic cell cycle	Meiotic recombination
MEI5	YPL121C	DEHA2G23298g	No	No	No	meiotic cell cycle	Meiotic recombination
MER1	YNL210W	DEHA2A11022g	PAS_chr3_0730	YALI0D05676g	No	meiotic cell cycle	Meiotic recombination
MLH2	YLR035C	No	No	No	No	meiotic cell cycle	Meiotic recombination
MLH3	YPL164C	DEHA2E10472g	PAS_chr3_0739	YALI0C07260g	ARAD1B17160g	meiotic cell cycle	Meiotic recombination
MSH4	YFL003C	DEHA2B13574g	PAS_chr1-4_0376	YALI0A09724g	ARAD1B17798g	meiotic cell cycle	Meiotic recombination
MUS81	YDR386W	DEHA2G05214g	PAS_chr2-1_0135	YALI0C14960g	ARAD1C39996g	meiotic cell cycle	Meiotic recombination
NDT80	YHR124W	DEHA2F21230g	PAS_chr2-2_0362	No	No	meiotic cell cycle	Meiotic recombination
REC102	YLR329W	No	No	No	No	meiotic cell cycle	Meiotic recombination
REC104	YHR157W	No	No	No	No	meiotic cell cycle	Meiotic recombination
REC107	YJR021C	No	No	No	No	meiotic cell cycle	Meiotic recombination
REC114	YMR133W	No	No	No	No	meiotic cell cycle	Meiotic recombination
SAE3	YHR079C-A	No	No	No	No	meiotic cell cycle	Meiotic recombination
GMC1	YDR506C	No	No	No	No	meiotic cell cycle	synaptonemal complex
GMC2	YLR445W	No	No	No	No	meiotic cell cycle	synaptonemal complex
HOP1	YIL072W	DEHA2D14740g	No	No	No	meiotic cell cycle	synaptonemal complex
RED1	YLR263W	No	No	No	No	meiotic cell cycle	synaptonemal complex
SPO11	YHL022C	DEHA2B13112g	No	YALI0F16335g	ARAD1C03784g	meiotic cell cycle	synaptonemal complex
SPO16	YHR153C	No	No	No	No	meiotic cell cycle	synaptonemal complex
ZIP1	YDR285W	No	No	No	No	meiotic cell cycle	synaptonemal complex
ZIP2	YGL249W	No	No	No	No	meiotic cell cycle	synaptonemal complex
ZIP3	YLR394W	No	No	No	No	meiotic cell cycle	synaptonemal complex
BNS1	YGR230W	No	No	YALI0B20559g	ARAD1D40480g	meiotic cell cycle	
CDC14	YFR028C	DEHA2F15356g	PAS_chr1-4_0081	YALI0E16038g	ARAD1D35002g	meiotic cell cycle	
CDC15	YAR019C	DEHA2C05060g	PAS_chr3_1061	YALI0F08165g	ARAD1B20350g	meiotic cell cycle	
CDC20	YGL116W	DEHA2E20966g	PAS_chr2-2_0495	YALI0C03377g	ARAD1C13024g	meiotic cell cycle	
CDC31	YOR257W	DEHA2F14784g	PAS_chr4_0388	YALI0A20518g	ARAD1C06886g	meiotic cell cycle	
CDC40	YDR364C	DEHA2G10142g	PAS_chr4_0942	YALI0D27346g	ARAD1C14630g	meiotic cell cycle	
CDC5	YMR001C	DEHA2F09966g	PAS_chr3_0243	YALI0D26015g	ARAD1B06974g	meiotic cell cycle	
CDC7	YDL017W	DEHA2C05588g	PAS_chr1-4_0192	YALI0F23287g	ARAD1D32560g	meiotic cell cycle	
CLB1	YGR108W	DEHA2A01760g	PAS_chr2-1_0102	YALI0B15180g	ARAD1B18942g	meiotic cell cycle	
CSM1	YCR086W	DEHA2A13486g	PAS_chr1-4_0004	No	No	meiotic cell cycle	

CSM2	YIL132C	No	No	No	No	meiotic cell cycle
CSM3	YMR048W	DEHA2F05038g	PAS_chr4_0244	YALI0E12287g	ARAD1D14498g	meiotic cell cycle
CSM4	YPL200W	No	No	No	No	meiotic cell cycle
DMC1	YER179W	DEHA2E16742g	PAS_chr3_0904	YALI0F15477g	ARAD1C10362g	meiotic cell cycle
DON1	YDR273W	DEHA2E07260g	PAS_chr2-2_0292	YALI0D00385g	ARAD1D07678g	meiotic cell cycle
ECM11	YDR446W	DEHA2G14498g	PAS_chr4_0241	No	No	meiotic cell cycle
EMI1	YDR512C	DEHA2C06512g	PAS_chr4_0869	YALI0B16962g	ARAD1B21208g	meiotic cell cycle
EMI2	YDR516C	DEHA2E06556g	PAS_chr4_0624	YALI0E15488g	ARAD1D19602g	meiotic cell cycle
EXO1	YOR033C	DEHA2E15444g	PAS_chr1-4_0633	YALI0E14014g	ARAD1C01540g	meiotic cell cycle
GLC7	YER133W	DEHA2E03674g	PAS_chr2-1_0635	YALI0A08077g	ARAD1A08338g	meiotic cell cycle
HOS4	YIL112W	DEHA2D02904g	PAS_chr4_0221	YALI0F00770g	ARAD1D35926g	meiotic cell cycle
HRR25	YPL204W	DEHA2C04290g	PAS_chr2-1_0219	YALI0F08305g	ARAD1D24046g	meiotic cell cycle
IDS2	YJL146W	No	No	No	No	meiotic cell cycle
IME1	YJR094C	No	No	No	No	meiotic cell cycle
IME2	YJL106W	DEHA2B00748g	PAS_chr1-3_0041	YALI0F03113g	ARAD1B20922g	meiotic cell cycle
IME4	YGL192W	DEHA2B04598g	PAS_chr2-1_0364	YALI0B03498g	ARAD1C32758g	meiotic cell cycle
LRS4	YDR439W	No	No	No	No	meiotic cell cycle
MAD2	YJL030W	DEHA2A10846g	PAS_chr4_0125	YALI0E10791g	ARAD1D06754g	meiotic cell cycle
MAM1	YER106W	No	No	No	No	meiotic cell cycle
MCK1	YNL307C	DEHA2E05060g	PAS_chr4_0812	YALI0D20966g	ARAD1B04290g	meiotic cell cycle
MEK1	YOR351C	No	No	No	No	meiotic cell cycle
MER3	YGL251C	DEHA2F07986g	PAS_chr1-1_0269	YALI0B13904g	ARAD1C39490g	meiotic cell cycle
MLH1	YMR167W	DEHA2G22022g	PAS_chr2-1_0165	YALI0C10032g	ARAD1B17160g	meiotic cell cycle
MMS4	YBR098W	DEHA2F15158g	PAS_chr2-1_0041	YALI0E33715g	ARAD1D48378g	meiotic cell cycle
MND1	YGL183C	DEHA2E04246g	No	No	No	meiotic cell cycle
MRE11	YMR224C	DEHA2F15818g	PAS_chr3_0851	YALI0B14553g	ARAD1D16258g	meiotic cell cycle
MSH2	YOL090W	DEHA2B15818g	PAS_chr1-4_0634	YALI0F26499g	ARAD1B17798g	meiotic cell cycle
MSH5	YDL154W	DEHA2B15818g	PAS_chr1-4_0634	YALI0B10197g	ARAD1B17798g	meiotic cell cycle
MSH6	YDR097C	DEHA2B13574g	PAS_chr2-1_0094	YALI0A09724g	ARAD1D37092g	meiotic cell cycle
MUM2	YBR057C	DEHA2G12298g	PAS_chr3_1241	YALI0A00369g	ARAD1A11176g	meiotic cell cycle
MUS81	YDR386W	DEHA2G05214g	PAS_chr2-1_0135	YALI0C14960g	ARAD1C39996g	meiotic cell cycle
NDJ1	YOL104C	No	No	No	No	meiotic cell cycle
NDT80	YHR124W	DEHA2A07282g	PAS_chr2-2_0362	YALI0B14773g	No	meiotic cell cycle

PDS5	YMR076C	DEHA2F19646g	PAS_chr3_0173	YALI0E12969g	ARAD1C18656g	meiotic cell cycle
PMS1	YNL082W	DEHA2A10868g	PAS_chr4_0124	YALI0E10769g	ARAD1D12122g	meiotic cell cycle
RAD1	YPL022W	DEHA2F08932g	PAS_chr2-2_0184	YALI0B22242g	ARAD1B12254g	meiotic cell cycle
RAD50	YNL250W	DEHA2D17314g	PAS_chr1-4_0513	YALI0D15246g	ARAD1D47388g	meiotic cell cycle
RAD51	YER095W	DEHA2C16698g	PAS_chr3_0904	YALI0F15477g	ARAD1C10362g	meiotic cell cycle
RAD52	YML032C	DEHA2G05148g	PAS_chr2-1_0153	YALI0F02431g	ARAD1B23606g	meiotic cell cycle
RAD55	YDR076W	**No**	PAS_chr1-4_0587	**No**	**No**	meiotic cell cycle
RAD57	YDR004W	DEHA2B13024g	PAS_chr1-3_0025	YALI0F10307g	ARAD1C29722g	meiotic cell cycle
RAM1	YDL090C	DEHA2G16918g	PAS_chr3_0636	YALI0D14762g	ARAD1A02112g	meiotic cell cycle
RDH54	YBR073W	DEHA2F06446g	PAS_FragB_0039	YALI0E24431g	ARAD1C24750g	meiotic cell cycle
RIM101	YHL027W	DEHA2D04796g	PAS_chr3_0625	YALI0B13640g	ARAD1C01914g	meiotic cell cycle
RIM11	YMR139W	DEHA2F08756g	PAS_chr1-4_0368	YALI0D20966g	ARAD1B04290g	meiotic cell cycle
RIM15	YFL033C	DEHA2F22572g	PAS_chr3_0148	YALI0F14707g	ARAD1C27148g	meiotic cell cycle
RIM4	YHL024W	DEHA2F03476g	PAS_chr1-3_0299	YALI0E16863g	ARAD1C37532g	meiotic cell cycle
RMD1	YDL001W	DEHA2F18150g	PAS_chr1-1_0444	YALI0B05390g	ARAD1B08360g	meiotic cell cycle
RMD6	YEL072W	DEHA2D13442g	**No**	**No**	**No**	meiotic cell cycle
RME1	YGR044C	DEHA2F19778g	PAS_chr1-1_0434	YALI0E17215g	ARAD1D33000g	meiotic cell cycle
SAE2	YGL175C	DEHA2C14212g	PAS_chr3_0435	YALI0C23727g	ARAD1D26114g	meiotic cell cycle
SET3	YKR029C	DEHA2B01386g	PAS_chr1-3_0037	YALI0F03069g	ARAD1B20944g	meiotic cell cycle
SGO1	YOR073W	DEHA2C06116g	PAS_chr3_0995	YALI0C07172g	ARAD1C17688g	meiotic cell cycle
SIN3	YOL004W	DEHA2F09900g	PAS_chr3_0245	YALI0D26315g	ARAD1B07018g	meiotic cell cycle
SLK19	YOR195W	DEHA2G18942g	PAS_chr2-1_0276	YALI0F02387g	ARAD1C09922g	meiotic cell cycle
Spmug66	**No**	DEHA2B08580g	**No**	YALI0B07073g	ARAD1C40227g**	meiotic cell cycle
SPO1	YNL012W	DEHA2F24420g	PAS_chr4_0039	YALI0E16060g	ARAD1D35024g	meiotic cell cycle
SPO12	YHR152W	**No**	**No**	YALI0B20559g**	ARAD1D40480g	meiotic cell cycle
SPO19	YPL130W	**No**	**No**	**No**	**No**	meiotic cell cycle
SPO21	YOL091W	**No**	**No**	**No**	**No**	meiotic cell cycle
SPO22	YIL073C	**No**	**No**	**No**	**No**	meiotic cell cycle
SPO23	YBR250W	**No**	**No**	**No**	**No**	meiotic cell cycle
SPO7	YAL009W	DEHA2C16104g	PAS_chr1-1_0248	YALI0D03091g	ARAD1C05764g	meiotic cell cycle
SSN8	YNL025C	DEHA2A09878g	PAS_chr2-2_0194	YALI0D04004g	ARAD1B21450g	meiotic cell cycle
SSP1	YHR184W	**No**	**No**	**No**	**No**	meiotic cell cycle
TOP2	YNL088W	DEHA2E01738g	PAS_chr3_0322	YALI0D23969g	ARAD1D22572g	meiotic cell cycle

TOS8	YGL096W	DEHA2D03454g	PAS_chr3_0590	YALI0E29271g	ARAD1C40612g	meiotic cell cycle	
TRS85	YDR108W	DEHA2E16830g	PAS_chr2-1_0774	YALI0E12177g	ARAD1D02508g	meiotic cell cycle	
UME1	YPL139C	DEHA2G16544g	PAS_chr2-1_0205	YALI0D26279g	ARAD1C17226g	meiotic cell cycle	
UME6	YDR207C	DEHA2E16236g	PAS_chr1-4_0401	YALI0A02497g	ARAD1D00638g	meiotic cell cycle	
WTM1	YOR230W	DEHA2F25234g	PAS_chr2-1_0205	YALI0D26279g	ARAD1C09592g	meiotic cell cycle	
WTM2	YOR229W	No	No	No	No	meiotic cell cycle	
YCS4	YLR272C	DEHA2F23386g	PAS_chr3_0265	YALI0F06402g	ARAD1C09438g	meiotic cell cycle	
YSC83	YHR017W	DEHA2A10670g	No	YALI0F28545g	ARAD1B07326g	meiotic cell cycle	
SPO14	YKR031C	DEHA2C02926g	PAS_chr3_0646	YALI0E18898g	ARAD1C40744g	sporulation	ascospore assembly
ADY3	YDL239C	DEHA2A12056g	PAS_chr4_0316	YALI0F30855g	ARAD1C09922g	sporulation	ascospore formation
ADY4	YLR227C	DEHA2E18084g	No	No	No	sporulation	ascospore formation
AMA1	YGR225W	DEHA2B14498g	PAS_chr1-4_0473	YALI0B17270g	ARAD1D40392g	sporulation	ascospore formation
CRR1	YLR213C	DEHA2D15532g	PAS_chr1-1_0293	YALI0B15510g	ARAD1D00396g	sporulation	ascospore formation
DIT1	YDR403W	No	No	No	No	sporulation	ascospore formation
DIT2	YDR402C	DEHA2E18612g	PAS_chr3_0957	YALI0B20702g	ARAD1D43758g	sporulation	ascospore formation
DTR1	YBR180W	DEHA2A10362g	PAS_chr2-2_0065	YALI0F03751g	ARAD1D36146g	sporulation	ascospore formation
GIP1	YBR045C	DEHA2D02904g	PAS_FragB_0067	YALI0B11770g	ARAD1C22704g	sporulation	ascospore formation
LOH1	YJL038C	No	No	No	No	sporulation	ascospore formation
MPC54	YOR177C	DEHA2C08778g	PAS_chr2-2_0307	YALI0F30855g	ARAD1C09922g	sporulation	ascospore formation
MUM3	YOR298W	DEHA2D17336g	PAS_chr4_0718	No	No	sporulation	ascospore formation
OSW1	YOR255W	No	No	No	No	sporulation	ascospore formation
OSW2	YLR054C	No	No	No	No	sporulation	ascospore formation
PFS1	YHR185C	DEHA2G21472g	No	No	No	sporulation	ascospore formation
SMA2	YML066C	DEHA2F06072g	PAS_chr4_0971	No	No	sporulation	ascospore formation
SMK1	YPR054W	DEHA2E18348g	PAS_chr3_0041	YALI0B02816g	ARAD1C38236g	sporulation	ascospore formation
SPO71	YDR104C	DEHA2G12804g	PAS_chr1-4_0435	YALI0D12540g	ARAD1C31592g	sporulation	ascospore formation
SPO73	YER046W	DEHA2A13332g	PAS_chr3_0550	YALI0C16841g	ARAD1D13552g	sporulation	ascospore formation
SPO74	YGL170C	No	No	No	No	sporulation	ascospore formation
SPO75	YLL005C	DEHA2G17292g	PAS_chr4_0560	YALI0D06864g	ARAD1C20702g	sporulation	ascospore formation
SPO77	YLR341W	No	No	No	No	sporulation	ascospore formation
SPR28	YDR218C	DEHA2C02266g	PAS_chr3_0397	YALI0E17765g	ARAD1D04620g	sporulation	ascospore formation
SPS1	YDR523C	DEHA2E15488g	PAS_chr1-1_0365	YALI0D19470g	ARAD1D47740g	sporulation	ascospore formation
SPS100	YHR139C	DEHA2G05918g	PAS_chr2-2_0481	YALI0F21450g	ARAD1D00638g	sporulation	ascospore formation

SPS2	YDR522C	DEHA2E15664g	PAS_c131_0003	YALI0D07480g	ARAD1D47718g	sporulation	ascospore formation
SSP2	YOR242C	DEHA2C12320g	PAS_chr3_1089	YALI0D06710g	ARAD1B21560g	sporulation	ascospore formation
SWM1	YDR260C	**No**	**No**	**No**	**No**	sporulation	ascospore formation
YSW1	YBR148W	DEHA2C08778g	PAS_chr1-4_0105	YALI0F02387g	ARAD1C09922g	sporulation	ascospore formation
CDA1	YLR307W	DEHA2C11836g	PAS_chr1-1_0103	YALI0F30833g	ARAD1C23012g	sporulation	
CDC10	YCR002C	DEHA2F17116g	PAS_chr4_0846	YALI0C21087g	ARAD1A01078g	sporulation	
CTS2	YDR371W	DEHA2G08866g	PAS_chr3_0107	YALI0F04532g	ARAD1B00396g	sporulation	
EMI5	YOL071W	DEHA2E13750g	PAS_chr2-1_0323	YALI0F11957g	ARAD1A15994g	sporulation	
FMP45	YDL222C	DEHA2F12386g	PAS_chr3_0287	YALI0F12331g	ARAD1D38478g	sporulation	
GAS3	YMR215W	DEHA2F06314g	PAS_chr3_0184	YALI0A03597g	ARAD1C11968g	sporulation	
GTS1	YGL181W	DEHA2E04136g	PAS_chr3_1109	YALI0B09603g	ARAD1D38236g	sporulation	
ISA1	YLL027W	DEHA2A06710g	PAS_chr1-1_0110	YALI0E19206g	ARAD1A14674g	sporulation	
ISC10	YER180C	**No**	**No**	**No**	**No**	sporulation	
MSO1	YNR049C	DEHA2F10692g	PAS_chr3_0529	YALI0C15532g	ARAD1D30228g	sporulation	
PRB1	YEL060C	DEHA2C13882g	PAS_chr1-1_0226	YALI0B16500g	ARAD1B09218g	sporulation	
RGR1	YLR071C	DEHA2E12958g	PAS_chr2-1_0800	YALI0D07194g	ARAD1A03058g	sporulation	
RMD8	YFR048W	DEHA2F22990g	PAS_chr1-4_0597	YALI0D01914g	ARAD1D46574g	sporulation	
RRT5	YFR032C	DEHA2B15246g	PAS_chr1-1_0218	YALI0F24717g	ARAD1C02904g	sporulation	
RSC1	YGR056W	DEHA2C01408g	PAS_chr2-2_0050	YALI0D20702g	ARAD1B07876g	sporulation	
SET1	YHR119W	DEHA2F20834g	PAS_chr2-2_0494	YALI0B14883g	ARAD1C16016g	sporulation	
SGA1	YIL099W	DEHA2A12254g	PAS_chr4_0579	YALI0E05203g	ARAD1D43296g	sporulation	
SHC1	YER096W	DEHA2A09768g	PAS_chr2-1_0261	YALI0F01925g	ARAD1A12474g	sporulation	
SLZ1	YNL196C	DEHA2D09966g	PAS_chr3_0814	YALI0D24563g	ARAD1C23144g	sporulation	
SMA1	YPL027W	**No**	**No**	**No**	**No**	sporulation	
SPO20	YMR017W	**No**	**No**	**No**	**No**	sporulation	
SPR1	YOR190W	DEHA2D08426g	PAS_chr2-1_0454	YALI0F05390g	ARAD1D09680g	sporulation	
SPR3	YGR059W	DEHA2G03058g	PAS_FragD_0011	YALI0D27148g	ARAD1D13882g	sporulation	
SPR6	YER115C	**No**	**No**	**No**	**No**	sporulation	
SPS18	YNL204C	DEHA2C02200g	PAS_chr3_0974	YALI0C02959g	ARAD1D04510g	sporulation	
SPS19	YNL202W	DEHA2C02178g	PAS_chr3_0975	YALI0C03003g	ARAD1D17534g	sporulation	
SPS4	YOR313C	DEHA2G06336g	**No**	YALI0F24167g	ARAD1D25850g	sporulation	
SSO1	YPL232W	DEHA2B09570g	PAS_chr1-4_0294	YALI0D25872g	ARAD1A16654g	sporulation	
SSO2	YMR183C	DEHA2B09570g	PAS_chr1-4_0294	YALI0D25872g	ARAD1A16654g	sporulation	

SUM1	YDR310C	No	No	No	No	sporulation
SUR7	YML052W	DEHA2F12386g	PAS_chr3_1058	YALI0F12331g	ARAD1D38478g	sporulation
SWS2	YNL081C	DEHA2A11704g	PAS_chr3_0520	YALI0B06578g	ARAD1D11968g	sporulation
TEP1	YNL128w	DEHA2B06116g	PAS_chr2-2_0351	YALI0C11033g	ARAD1D24728g	sporulation
VPS13	YLL040C	DEHA2D12430g	PAS_chr1-1_0211	YALI0F06791g	ARAD1C29854g	sporulation
YEL023C	YEL023c	No	PAS_chr2-1_0470	YALI0A02893g	ARAD1D50578g	sporulation
YFL012W	YFL012W	No	No	No	No	sporulation
YJL147C	YJL147C	No	No	No	No	sporulation
YJL160C	YJL160C	DEHA2B13442g	PAS_chr2-1_0887	YALI0B20306g	No	sporulation
YLR446W	YLR446W	DEHA2F24618g	PAS_chr1-4_0447	YALI0E20207g	ARAD1D41822g	sporulation
YNL194C	YNL194C	DEHA2F12386g	PAS_chr3_0287	YALI0F12331g	ARAD1D38478g	sporulation
YNL296W	YNL296W	No	No	No	No	sporulation
YOR268C	YOR268C	No	No	No	No	sporulation
YOR338W	YOR338W	DEHA2B05698g	PAS_chr3_0505	YALI0D12078g	ARAD1B17666g	sporulation
AFR1	YDR085C	DEHA2D03608g	No	No	No	signaling
BAG7	YOR134W	DEHA2G12122g	PAS_chr2-1_0117	YALI0D13684g	ARAD1D34584g	signaling
BCY1	YIL033C	DEHA2A01474g	PAS_chr3_0546	YALI0F04422g	ARAD1D15356g	signaling
BUD6	YLR319C	DEHA2C11022g	PAS_chr2-1_0372	YALI0E27918g	ARAD1D00858g	signaling
CDC24	YAL041W	DEHA2E11836g	PAS_chr1-4_0533	YALI0C14828g	ARAD1A02662g	signaling
CDC42	YLR229C	DEHA2G14168g	PAS_chr2-1_0112	YALI0B15752g	ARAD1C32340g	signaling
CMK2	YOL016C	DEHA2F09284g	PAS_chr1-4_0302	YALI0E27632g	ARAD1B13838g	signaling
CYR1	YJL005W	DEHA2C17490g	PAS_chr4_0356	YALI0B18759g	ARAD1C28182g	signaling
DIG2	YDR480W	DEHA2D15686g	PAS_chr4_0099	No	No	signaling
DSE1	YER124C	DEHA2D03696g	PAS_chr2-1_0355	YALI0D09823g	ARAD1B17490g	signaling
FAR1	YJL157C	DEHA2F13728g	PAS_chr1-3_0307	YALI0F20680g	ARAD1A10494g	signaling
FUS3	YBL016W	DEHA2E20086g	PAS_chr2-1_0872	YALI0E23496g	ARAD1C44286g	signaling
FYV5	YCL058C	No	No	No	No	signaling
GPA1	YHR005C	DEHA2D13618g	PAS_chr1-1_0096	YALI0E11627g	ARAD1D25520g	signaling
HOS2	YGL194C	DEHA2A08712g	PAS_chr2-2_0016	YALI0C06061g	ARAD1B08030g	signaling
NPR3	YHL023C	DEHA2E02046g	PAS_chr1-4_0385	YALI0A00682g	ARAD1A18194g	signaling
OPY1	YBR129C	DEHA2E22264g	PAS_chr3_0666	YALI0A14476g	ARAD1B02618g	signaling
OXR1	YPL196W	DEHA2G01320g	PAS_chr2-1_0031	YALI0E29865g	ARAD1C26774g	signaling
PEA2	YER149C	DEHA2G11792g	PAS_chr1-3_0291	No	No	signaling

RAS2	YNL098C	DEHA2F09438g	PAS_chr2-2_0347	YALI0E08756g	ARAD1B22924g	signaling
RIB5	YBR256C	DEHA2D13926g	PAS_chr4_0081	YALI0C01111g	ARAD1C34540g	signaling
RIM13	YMR154C	DEHA2G20988g	PAS_chr3_0635	YALI0D14740g	ARAD1A02090g	signaling
ROM2	YLR371W	DEHA2G05456g	PAS_chr2-1_0586	YALI0B20218g	ARAD1C21076g	signaling
RSR1	YGR152C	DEHA2C16544g	PAS_chr2-2_0347	YALI0F23177g	ARAD1D37928g	signaling
SCP160	YJL080C	DEHA2C03124g	PAS_chr1-3_0045	YALI0B21662g	ARAD1B21010g	signaling
SIF2	YBR103W	DEHA2F15598g	PAS_chr1-4_0201	YALI0F11165g	ARAD1B23892g	signaling
SNF1	YDR477W	DEHA2E15180g	PAS_chr2-1_0855	YALI0D02101g	ARAD1D01078g	signaling
SPA2	YLL021W	DEHA2E16588g	PAS_chr3_0816	YALI0F16665g	ARAD1A07238g	signaling
SST2	YLR452C	DEHA2E10164g	PAS_chr3_0840	YALI0D18018g	ARAD1A18480g	signaling
TOR1	YJR066W	DEHA2F23452g	PAS_chr2-1_0557	YALI0F07084g	ARAD1C30470g	signaling
TOR2	YKL203C	DEHA2F23452g	PAS_chr2-1_0557	YALI0F07084g	ARAD1C30470g	signaling
YSP1	YHR155W	DEHA2E14674g	PAS_chr2-1_0795	YALI0A09020g	ARAD1B18172g	signaling
YVH1	YIR026C	DEHA2F20460g	PAS_chr1-3_0193	YALI0B11572g	ARAD1D30734g	signaling
APC1	YNL172W	DEHA2D08932g	PAS_chr1-4_0020	YALI0D16885g	ARAD1D47960g	mitotic cell cycle
APC4	YDR118W	DEHA2B08316g	PAS_chr3_0477	YALI0D12705g	ARAD1A12012g	mitotic cell cycle
BIM1	YER016W	DEHA2D14278g	PAS_chr4_0999	YALI0A18227g	ARAD1C08734g	mitotic cell cycle
BNI1	YNL271C	DEHA2E14366g	PAS_chr4_0636	YALI0D10879g	ARAD1D09262g	mitotic cell cycle
CDC16	YKL022C	DEHA2C07062g	PAS_chr1-4_0197	YALI0B15378g	ARAD1C19624g	mitotic cell cycle
CDC23	YHR166C	DEHA2E02156g	PAS_chr3_1078	YALI0C10692g	ARAD1D49126g	mitotic cell cycle
CDC27	YBL084C	DEHA2F20614g	PAS_chr1-3_0148	YALI0A19712g	ARAD1B03102g	mitotic cell cycle
CDH1	YGL003C	DEHA2A06864g	PAS_chr2-2_0495	YALI0C18469g	ARAD1C14036g	mitotic cell cycle
CLB3	YDL155W	DEHA2G24134g	PAS_chr4_0604	YALI0B19206g	ARAD1D17908g	mitotic cell cycle
CLB5	YPR120C	**No**	**No**	**No**	**No**	mitotic cell cycle
SMC1	YFL008W	DEHA2F20020g	PAS_chr2-1_0082	YALI0E15620g	ARAD1D43648g	mitotic cell cycle
SMC2	YFR031C	DEHA2B15136g	PAS_chr4_0109	YALI0F24783g	ARAD1C02948g	mitotic cell cycle
SMC4	YLR086W	DEHA2B07920g	PAS_chr2-2_0416	YALI0C19129g	ARAD1B04840g	mitotic cell cycle
SWI5	YDR146C	DEHA2E03850g	PAS_chr1-3_0099	YALI0E16973g	ARAD1C37444g	mitotic cell cycle
TID3	YIL144W	DEHA2E09548g	PAS_chr1-4_0203	YALI0B11814g	ARAD1C22528g	mitotic cell cycle
XBP1	YIL101C	DEHA2E10714g	**No**	YALI0F16511g	ARAD1D46640g	mitotic cell cycle
DEF1	YKL054C	DEHA2D17732g	PAS_chr1-1_0470	YALI0C09933g	ARAD1A01518g	response to DNA damage
DIN7	YDR263C	DEHA2E15444g	PAS_chr1-4_0633	YALI0E14014g	ARAD1C01540g	response to DNA damage
DNL4	YOR005C	DEHA2G04224g	PAS_chr3_0732	YALI0D21384g	ARAD1C02090g	response to DNA damage

ELC1	YPL046C	DEHA2D13046g	No	YALI0A03575g	ARAD1C30866g	response to DNA damage
MSH1	YHR120W	DEHA2F20856g	PAS_chr2-2_0364	YALI0B10197g	ARAD1C16038g	response to DNA damage
POL3	YDL102W	DEHA2G22308g	PAS_chr2-1_0163	YALI0A08426g	ARAD1B21362g	response to DNA damage
RAD23	YEL037C	DEHA2B04180g	PAS_chr2-2_0445	YALI0D16401g	ARAD1A04510g	response to DNA damage
RAD54	YGL163C	DEHA2D05456g	PAS_chr3_0224	YALI0B07513g	ARAD1A16698g	response to DNA damage
RSC2	YLR357W	DEHA2C01408g	PAS_chr2-2_0050	YALI0D20702g	ARAD1B07876g	response to DNA damage
SIR4	YDR227W	DEHA2C08778g	PAS_FragB_0067	YALI0B11770g	ARAD1C09922g	response to DNA damage
SMC5	YOL034W	DEHA2A12606g	PAS_chr1-1_0245	YALI0E17193g	ARAD1A13508g	response to DNA damage
SMC6	YLR383W	DEHA2A05324g	PAS_chr1-4_0535	YALI0F02365g	ARAD1C09900g	response to DNA damage
SRS2	YJL092W	DEHA2B14410g	PAS_chr3_0219	YALI0E27269g	ARAD1B18128g	response to DNA damage
APC5	YOR249C	DEHA2C08668g	No	YALI0F05478g	ARAD1D19228g	chromatin organization
ARD1	YHR013C	DEHA2G04598g	PAS_chr1-3_0090	YALI0B05742g	ARAD1A17578g	chromatin organization
CAC2	YML102W	DEHA2F19074g	PAS_chr3_0248	YALI0B15015g	ARAD1B02090g	chromatin organization
CDC26	YFR036W	No	No	No	No	chromatin organization
MSI1	YBR195C	DEHA2F07920g	PAS_chr3_1191	YALI0B13750g	ARAD1C09086g	chromatin organization
RLF2	YPR018W	DEHA2E16060g	PAS_chr3_0814	YALI0F21637g	ARAD1B23562g	chromatin organization
SDS3	YIL084C	DEHA2A10450g	PAS_chr2-2_0472	YALI0F25663g	ARAD1D28204g	chromatin organization
SIR1	YKR101W	No	No	No	No	chromatin organization
ULS1	YOR191W	DEHA2F05676g	PAS_chr2-1_0447	YALI0F30261g	ARAD1B06094g	chromatin organization
ARE2	YNR019W	DEHA2F24222g	PAS_chr3_0467	YALI0F06578g	ARAD1B15510g	metabolism
ARN2	YHL047C	No	No	No	No	metabolism
CHO1	YER026C	DEHA2B15686g	PAS_chr1-1_0420	YALI0D08514g	ARAD1C15444g	metabolism
CLG1	YGL215W	DEHA2B03630g	PAS_chr4_0189	YALI0E07898g	ARAD1B01056g	metabolism
DAP1	YPL170W	DEHA2F14872g	PAS_chr4_0396	YALI0A20394g	ARAD1C06974g	metabolism
ENA2	YDR039C	DEHA2C02552g	PAS_chr1-1_0428	YALI0A01023g	ARAD1D26796g	metabolism
FBP1	YLR377C	DEHA2F01100g	PAS_chr3_0868	YALI0A15972g	ARAD1D31196g	metabolism
GLG1	YKR058W	DEHA2D15070g	PAS_chr4_0847	YALI0C21065g	ARAD1A01034g	metabolism
GNA1	YFL017C	DEHA2B05126g	PAS_chr4_0060	YALI0D20152g	ARAD1D45914g	metabolism
GTT1	YIR038C	DEHA2D16280g	PAS_chr2-2_0116	YALI0F25575g	ARAD1D41712g	metabolism
KGD1	YIL125W	DEHA2F17798g	PAS_chr2-1_0089	YALI0E33517g	ARAD1A11814g	metabolism
NEM1	YHR004C	DEHA2B02970g	PAS_chr2-1_0248	YALI0C02849g	ARAD1D07832g	metabolism
PCT1	YGR202C	DEHA2G21560g	PAS_chr2-2_0401	YALI0D18271g	ARAD1B14960g	metabolism
PDC1	YLR044C	DEHA2G18348g	PAS_chr3_0188	YALI0D10131g	ARAD1D26004g	metabolism

PGM2	YMR105C	DEHA2C05258g	PAS_chr1-4_0264	YALI0E02090g	ARAD1D37224g	metabolism
PMC1	YGL006W	DEHA2A09086g	PAS_chr1-1_0117	YALI0D04873g	ARAD1A06798g	metabolism
RKI1	YOR095C	DEHA2A09328g	PAS_chr4_0213	YALI0B06941g	ARAD1C12474g	metabolism
SUR4	YLR372W	DEHA2G05478g	PAS_chr3_0236	YALI0B20196g	ARAD1C21098g	metabolism
XKS1	YGR194C	DEHA2C06974g	PAS_chr1-1_0280	YALI0F10923g	ARAD1C08800g	metabolism
ACF2	YLR144C	DEHA2G00726g	PAS_chr1-1_0130	YALI0B17996g	ARAD1D27192g	other
ACT1	YFL039C	DEHA2D05412g	PAS_chr3_1169	YALI0D08272g	ARAD1A06666g	other
ADY2	YCR010C	DEHA2F17996g	PAS_chr1-1_0158	YALI0C23617g	ARAD1A09152g	other
ATG8	YBL078C	DEHA2D03960g	PAS_chr4_0704	YALI0E02662g	ARAD1C41976g	other
CCH1	YGR217W	DEHA2D14586g	PAS_chr2-1_0301	YALI0E15334g	ARAD1D08602g	other
CDC3	YLR314C	DEHA2D12540g	PAS_chr4_0548	YALI0D23595g	ARAD1D21428g	other
CHS1	YNL192W	DEHA2D03916g	PAS_chr1-1_0393	YALI0D25938g	ARAD1C33682g	other
CYB2	YML054C	DEHA2D05522g	PAS_chr4_0272	YALI0E21307g	ARAD1C32274g	other
ECM4	YKR076W	DEHA2C16566g	PAS_chr2-1_0315	YALI0B20416g	ARAD1C31680g	other
ESC8	YOL017W	DEHA2F23958g	**No**	**No**	**No**	other
GDI1	YER136W	DEHA2F25102g	PAS_chr3_0531	YALI0E33649g	ARAD1C04994g	other
HHO1	YPL127C	DEHA2C16478g	PAS_chr1-4_0282	YALI0B16280g	ARAD1B19515g	other
HO	YDL227C	**No**	**No**	**No**	**No**	other
HTZ1	YOL012C	DEHA2E05720g	PAS_chr4_0157	YALI0F02827g	ARAD1C32890g	other
HUL4	YJR036C	DEHA2F11704g	PAS_chr2-1_0469	YALI0D15444g	ARAD1C07260g	other
KRE9	YJL174W	DEHA2B10318g	PAS_chr3_0960	YALI0F17490g	ARAD1D45056g	other
LEE1	YPL054W	DEHA2D15928g	PAS_chr4_0104	YALI0B12540g	ARAD1D45232g	other
MPT5	YGL178W	DEHA2C14278g	PAS_chr3_0438	YALI0E10395g	ARAD1C41778g	other
NAM8	YHR086W	DEHA2F23276g	PAS_chr1-4_0223	YALI0D04917g	ARAD1A08470g	other
NAT1	YDL040C	DEHA2E11506g	PAS_chr1-1_0373	YALI0F12221g	ARAD1A15906g	other
NPT1	YOR209C	DEHA2G23870g	PAS_chr2-1_0221	YALI0B00220g	ARAD1C17842g	other
PFY1	YOR122C	DEHA2G14784g	PAS_chr3_0106	YALI0B07183g	ARAD1C39402g	other
PGI1	YBR196C	DEHA2F13156g	PAS_chr3_0456	YALI0F07711g	ARAD1C38940g	other
PIB1	YDR313C	DEHA2E08030g	PAS_chr1-1_0290	YALI0D15510g	ARAD1D31174g	other
PIN3	YPR154W	DEHA2B14476g	PAS_chr4_0500	YALI0C04114g	ARAD1D13046g	other
RFM1	YOR279C	**No**	**No**	**No**	**No**	other
RMD5	YDR255C	DEHA2D13024g	PAS_chr3_0209	YALI0A18788g	ARAD1C30844g	other
SCW11	YGL028C	DEHA2E22440g	PAS_chr2-1_0052	YALI0E21109g	ARAD1D24156g	other

SKT5	YBL061C	DEHA2A09768g	PAS_chr2-1_0261	YALI0F01925g	ARAD1A12474g	other
SNF4	YGL115W	DEHA2B07282g	PAS_chr2-1_0608	YALI0C03421g***	ARAD1C13068g	other
STU1	YBL034C	DEHA2F25872g	PAS_chr3_0913	YALI0D21912g	ARAD1C41008g	other
SYF2	YGR129W	DEHA2C03366g	PAS_chr4_0309	YALI0B03036g	ARAD1C04378g	other
TOS7	YOL019W	DEHA2G08932g	PAS_chr3_0919	YALI0E33627g	ARAD1A07766g	other
UBC11	YOR339C	**No**	**No**	**No**	**No**	other

*see Kuberl et al. 2011

**New gene

***Pseudo
