# Supplementary material for: The complete genome of Blastobotrys (Arxula) adeninivorans LS3 - a yeast of biotechnological interest
Source: Biotechnol Biofuels. 2014 Apr 24;7:66. doi: 10.1186/1754-6834-7-66 (PMC4022394; doi:10.1186/1754-6834-7-66)
Supplement: Additional file 14 — Mating pheromones. [file 1754-6834-7-66-S14.pdf]

Additional File 14. Mating pheromones

A potential candidate for MFα was identified in *A. adeninivorans* as the product of ARAD1C26928g, which contains eleven repeats of a 13 amino-acid peptide (IPKWEQM/LPGQPID) matching the sequence of other yeast MFα (Fig. S13A). Consensus processing sites for the signal peptidase and for Kex2p are present, but not for Ste13 since there are no X-Ala/X-Pro repeats. This suggests that MFα processing in *A. adeninivorans* is atypical: either there is only Kex2-like processing and 19 amino acid pheromones would be produced, partially divergent at their N-terminus, or another proteolytic machinery is involved in N-terminal trimming (Fig. S13B). We note that genes encoding Kex2, Kex1 and Ste13 genes exist in the sequenced strain.

The genes encoding the precursors of the other mating pheromone MFa are very short sized and poorly conserved, making them difficult to identify in genomes distant from *S. cerevisiae* without synteny data [22]. A possible candidate encoded by ARAD1D33583g was detected after a search for short proteins ending in CAAX required for prenylation and carboxylmethylation of Mfap (Fig. S13C). The 44 amino acid gene product is poorly conserved outside from its C-terminal CVIA motif, thus preventing prediction of the N-terminal processing sites [23]. All the machinery necessary for maturation and export of MFa is well conserved.

Figure S14A Precursors of MFalpha in hemiascomycetous yeasts.

Over lining indicates: green= signal peptide; grey= prodomain, blue= dibasic aminoacids (Kex2 site); yellow= Xala/Xpro repeats (Ste13 processing); red= mating pheromone alpha. Notice absence of Xala/Xpro repeats in *A. adeninivorans* pheromone precursor.

Figure S14B Alignment of alpha pheromones of representative species of hemiascomycetes.

Alignments were generated using Multalin and manually adjusted.

Figure S14C Alignment of *A. adeninivorans* putative precursor of pheromone a (Mfap).

Sequences are derived from: *S. cerevisiae* YDR461W (Sace_MFA1), *S. cerevisiae* YNL145W (Sace_MFA2), *C. glabrata* CAGL0C01919g (Cagl_MFA2), *Naumovozyma (Saccharomyces) castellii* Scas564.10d1 (Nacas_MFA), *Eremothecium (Ashbya) gossypii* ABL196Cp (Ergo_MFA2), *C. albicans* orf19.2164.1 (Caal_MFA1), *Y. lipolytica* YALI0C17028g (Yali_MFA2), and *A. adeninivorans* ARAD1D33583g. Note that loss of the conserved DN landmark prevents definition of the N-terminal processing site.

Sequences were aligned using ClustalX2.

Figure S14A

>YPL187wp *S. cerevisiae*
MRFPSIFTAVLFAASSALAAPVNTTEDETAQIPAEAVIGYLDLEGDFDVALPFSNSTNNGLLFINTSIAAKEEGVSLDKREAEAWH
WLQLKPGQPMYKREAEAEAWHWLQLKPGQPMYKREADAEAWHWLQLKPGQPMYKREADAEAWHWLQLKPGQPMY

>DEHA2F19580p *D. hansenii*
MKFSIFALTTLVSTISLAAAAPTPDSTETGKYVVLDYLVPDEAINNKVEITDDQQPLVVEESGKKYVLIVNATLAESVISKAGIDIE
GLEAAFAKSDDTASVSKRDANADAEAKFHWMTYRFFQPNLRKREANADAKFHWMTYRFFQPNLKKREANADAEAKFHWMTYR
FFQPNLKKREANAEANADAKFHWMTYRFFQPNLKKREANADAEAKFHWMTYRFFQPNL

>PAS_chr2-1_0883p *K. pastoris*
MKSLILNIISVTLAITSTAASAPVESIFANQPDSSLTDTNDGVGVGMSTIKEEDFGKHFVENQILDEAVIMSLKLRKGVNLFFLDDI
GLATELIGNKIAQIEAIDLSERLAQSWTNIRKNRLFGKREAEAEAEAEAFRWRNNEKNQPFGKREAEAEAEAEAEAEAEAFRW
RNNEKNQPFGKREAEAEAEAEAEAEAEAFRWRNNEKNQPFGKREAEAEAEAEAFRWRNNEKNQPFGKREADAEAEAEAEAFR
WRNNEKNQPFGKREAEAEAEAEAFRWRNNEKNQPFGKREAEAEAEAEAEAEAFRWRNNEKNQPFGKREAEAEAEAEAFRWRN
NEKNQPFGKREADAEAEAEAEAFRWRNNEKNQPFGKREASIDTGTDDGAYWSWRKNSVLERQ

>ARAD1C26928p *A. adeninivorans*
MHLKASFLLLIATAIVSTAAPISGDQQNVTEPDVDHQIPECQPRSPPIDRRDEKWEPDIPKWEQMPGQPIDKRDEKWKPDIPK
WEQLPGQPIDRRDVKWEPNIPKWEQMPGQPIDKRDEKWTPDIPKWEQMPGQPIDKRDVKWKPDIPKWEQMPGQPIDRRDV
KWTPDIPKWEQMPGQPIDRRDVKWTPDIPKWEQMPGQPIDKRDVKWEPNIPKWEQMPGQPIDKRDVKWKPDIPKWEQMP
GQPIDKRDVRWKPDIPKWEQMPGQPIDK

>YALI0E16533p *Y. lipolytica*
MKFSTIALAAVACLVSAAPAAPVGTGSHGPQSIPEEAIVGGLQGTENEIFVFFNDDESGKQGIAIIDAKKAQEAGFMDPQPDSEV
AAGNAKREASPEAWRWFWLPGYGEPNWKRDAMPADMDKEKREANPEAWRWFWLPGYGEPNWKRDAMPADMDKEKREAN
PEAWRWFWLPGYGEPNWKRDAMPADMDKEKREANPEAWRWFWLPGYGEPNW

Figure S14B

```
                        *
Sace   : -WH LQLKP-GQP-MY : 13
Klla   : -WS ITLRP-GQP-IF : 13
Klth   : -WR LSLSR-GQP-MY : 13
Sakl_1 : -WH LSFSK-GEP-MY : 13
Zyro   : --H IELDP-GQP-MF : 12
Arad   : IPK EQMPG--QP-ID : 13
Pipa   : -FR RNNEK-NQP-FG : 13
Sakl_2 : -WH LSFSF-GEP-LY : 13
Cagl   : -WH VKIRK-GQG-LF : 13
Piso_1 : KFH FKYNN-FDP-IT : 14
Piso_2 : KFH FTYSN-FDP-IT : 14
Deha   : KFH MTYRF-FQPNLR : 15
Yali   : -WR FWLPGYGEP-NW : 14
```

Figure S14C

```
                        *         20         *         40         *         60         *
Sace_MFA1    : ---------MQP--STATAAPKEKTSSEKKDN---YIIKG--VFWDP--------------------ACVIA : 36
Sace_MFA2    : ---------MQPITTASTQATQKDKSSEKKDN---YIIKG--LFWDP--------------------ACVIA : 38
Cagl_MFA2    : ---------MQP----TIEATQKDNTQEKRDN---YIVKG--FFWSP--------------------DCVIA : 34
Naca_MFA     : ---------MQP----TTQATHKDNSAEKQDN---YIVKG--LFWDP--------------------ACVIA : 34
Ergo_MFA2    : ---------MQL-----TNNTNKDESTENKDN---WIAKG--YMWTP--------------------QCVIV : 33
Caal_MFA1    : -------MAAQQQSKKTGSGQTKDKDAAAKNNAVRSVSTG--NCCST---------------------CSVM : 42
ARAD1D33583g : --------MYEPVAIEFAVSRPQSGVRVVPRR---VVMIAEMNLQGPG-----------------SISCVIA : 44
Yali_MFA2    : MTSWFDILQLAKQHKSHQSLAVDNNKNDKTKAPPLLPLQDNMAHYDPSWTHEQKVQEVKRRQSQSFRLCVIA : 72
Consensus               qp          kd      dn       g    w P                     CVI a
```
