# Supplementary material for: The complete genome of Blastobotrys (Arxula) adeninivorans LS3 - a yeast of biotechnological interest
Source: Biotechnol Biofuels. 2014 Apr 24;7:66. doi: 10.1186/1754-6834-7-66 (PMC4022394; doi:10.1186/1754-6834-7-66)
Supplement: Additional file 15 — Tannic acid degradation pathways in A. adeninivorans. [file 1754-6834-7-66-S15.pdf]

Additional File 15. Tannic acid and n-butanol degradation pathways in *A. adeninivorans*

Figure S15 Schematic overview of the tannic acid degradation pathway in A. adeninivorans..

The tannic acid degradation pathway includes extracellular (Atan1p, ATAN1 - ARAD1A06094g) and cell wall bound tannases (Atan2p, ATAN2 - ARAD1A19822g), gallate decarboxylase (Agdcp, AGDC - ARAD1C45804g) and catechol 1,2 dioxygenase (Acdop, ACDO - ARAD1D18458g) and enables the yeast to assimilate tannic acid and other hydroxylated derivatives of benzoic acid by non-oxidative decarboxylation. For three key genes expression patterns are shown as heat map indicating maximum expression 6 hours after induction with gallic acid.

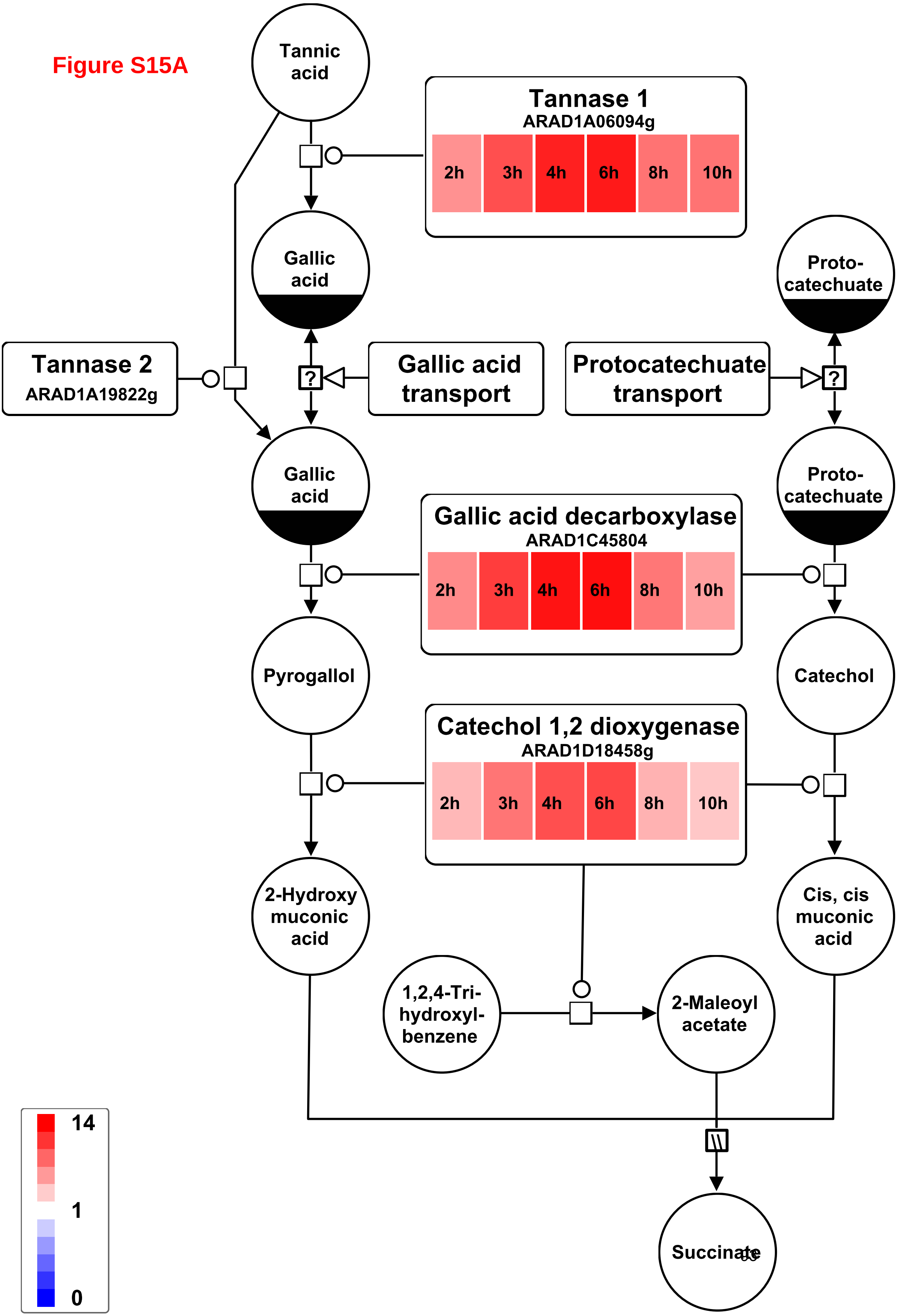
Figure S15A
Tannic acid
Tannase 1
ARAD1A06094g
2h
3h
4h
6h
8h
10h
Gallic acid
Proto-catechuate
Tannase 2
ARAD1A19822g
?
Gallic acid transport
Protocatechuate transport
?
Gallic acid
Proto-catechuate
Gallic acid decarboxylase
ARAD1C45804
2h
3h
4h
6h
8h
10h
Pyrogallol
Catechol
Catechol 1,2 dioxygenase
ARAD1D18458g
2h
3h
4h
6h
8h
10h
2-Hydroxy muconic acid
Cis, cis muconic acid
1,2,4-Tri-hydroxyl-benzene
2-Maleoyl acetate
Succinate
14
1
0
